# Synthesis of diversely substituted bis-pyrrolizidino/ thiopyrrolizidino oxindolo/acenaphthyleno curcuminoids *via* sequential azomethine ylide cycloaddition†

**DOI:** 10.1039/c8ra02725k

**Published:** 2018-05-23

**Authors:** Meenakshi Singh, Abhijit Hazra, Yogesh P. Bharitkar, Ritu Kalia, Ashutosh Sahoo, Sneha Saha, V. Ravichandiran, Shekhar Ghosh, Nirup B. Mondal

**Affiliations:** National Institute of Pharmaceutical Education and Research (NIPER) (IICB Campus), 4, Raja S. C. Mullick Road, Jadavpur Kolkata – 700 032 India; Department of Organic and Medicinal Chemistry, Indian Institute of Chemical Biology, Council of Scientific and Industrial Research 4, Raja S. C. Mullick Road, Jadavpur Kolkata – 700 032 India apuhazra@gmail.com yogeshbharitkar@gmail.com

## Abstract

Curcumin has been transformed to several diversely substituted bis-pyrrolizidino/thiopyrrolizidino oxindolo/acenaphthyleno curcuminoids *via* a sequential azomethine ylide cycloaddition reaction using isatins/acenaphthoquinone and proline/thioproline as the reagents. The products were separated *via* extensive chromatography and characterized by 1D/2D NMR and HRMS analysis.

## Abstract 2

Curcumin has been transformed to several diversely substituted bis-pyrrolizidino/thiopyrrolizidino oxindolo/acenaphthyleno curcuminoids *via* a sequential azomethine ylide cycloaddition reaction.

## Graphical abstract

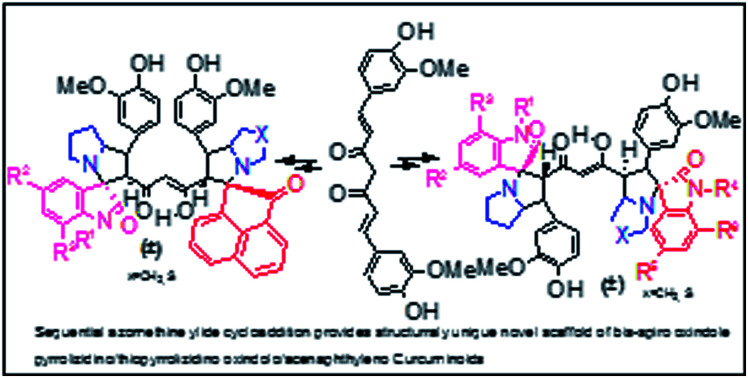

## Introduction

Incorporating diversity in the synthesis of combinatorial libraries of small molecules for biological screening is an emerging field.^1^ Rather than being directed toward a single biological target, diversified libraries can be used to identify new ligands for a variety of targets. It is hoped that the range of molecular architectures and potential bonding interactions present in a diversified library can provide interesting and specific biological activity across a range of targets. Although various chemical libraries are now available commercially, these remain focused primarily on so called ‘drug-like’ compounds.^2^ Because these libraries are concentrated in a relatively narrow region of chemical structure space, it seems unlikely that they will provide useful probes for all biological targets of interest.^3^ The crucial factor for achieving success in drug discovery is not the size of the library but its structural diversity.^4^ Several different strategies for library design have therefore been developed to target the biologically relevant regions of chemical structure space. DOS has provided powerful probes to investigate biological mechanisms and also served as a new driving force for advancing synthetic organic chemistry.

To provide cyclic and heterocyclic compounds with a high degree of structural complexity as well as skeletal and stereochemical diversity, dipolar cycloaddition reaction has emerged as a potential tool.^5^ Its ability to generate new stereocenters has allowed it to contribute very much to the development of stereo structure-activity relationships during screening campaigns. In particular, the sequential multicomponent reaction^6^ and sequential azomethine ylide (1,3 dipole) cycloaddition approach has emerged as one of the efficient strategies which can provide diverse spirooxindoles in an operationally simple procedure from readily available chemical reagents.^7^ Keeping the above facts in mind we started our journey of preparing diversely functionalized heterocycles *via* azomethine ylide cycloaddition using simple commercially available^8^ or synthetic dipolarophiles.^9^ We then extended it to a new dimension by employing dipolarophiles available from nature like andrographolide,^10^ withaferin A,^11^ curcumin^12^*etc.* Several compounds have been prepared and biological activity evaluation revealed some very promising increment in activity.^13,14^ In continuation of our molecular diversity programme, very recently we have synthesized various spirooxindolo super curcumin analogues^12^ to overcome the drawbacks related to the bioavailability of curcumin (less water solubility, easy metabolism and excretion) with comparable or better efficacy. An equally compelling motivation for their synthesis lies in their unique and formidable structure, the central feature being the biologically important curcumin and isatin (oxindole) units^12^ likewise done by various other group by synthsizing various diversified heterocyclic analogs of curcumin and their bioevaluation.^15–18^ We have so far succeeded in synthesizing a library of pyrrolizidino spirooxindolo curcumins, some with better and equal cytotoxic/antioxidant and antibacterial activity^19^ but with much more specificity and solubility compared to curcumin. These results of biological evaluation encouraged us to construct a better diversified library applying the sequential azomethine ylide cycloaddition strategy, coupling isatin, substituted isatins or acenapthoquinone with proline or thioproline as the amino acid component.

## Results and discussion

**Scheme 1:**
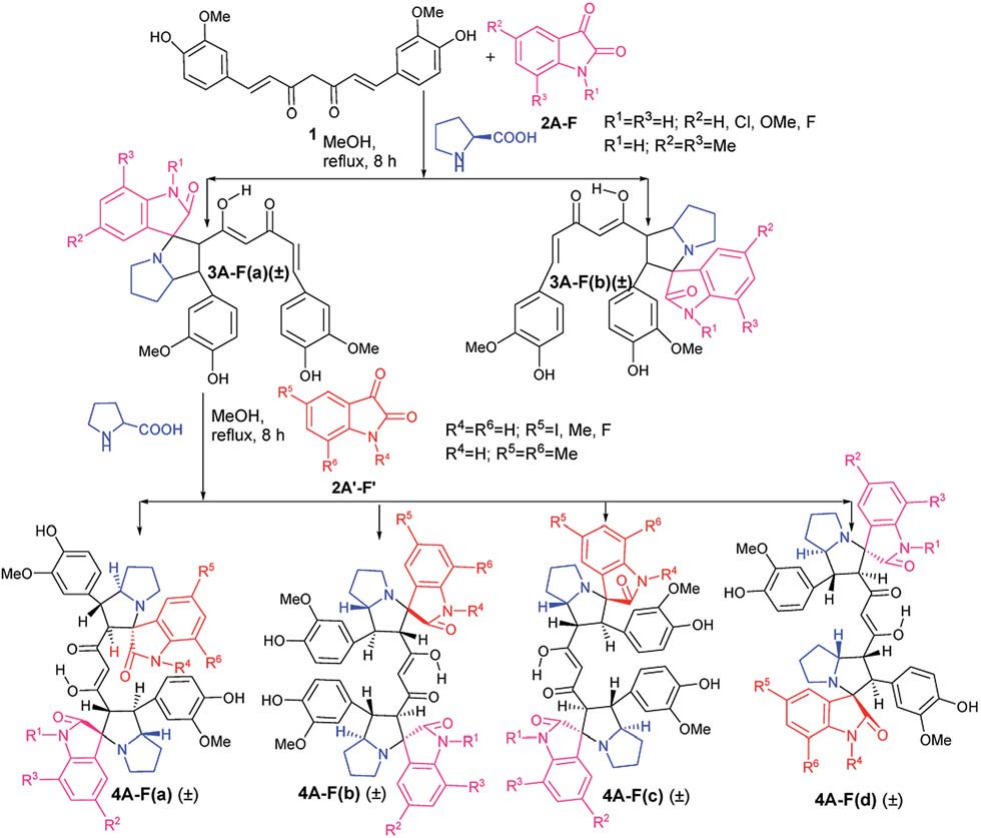
Synthesis of mixed bis-pyrrolizidino dispiro-oxindolo curcuminoids.

The first step in the two step sequential cycloaddition reaction was performed using curcumin (1), isatins (2A) and proline in 1 : 1 : 1 mole ratio in refluxing methanol *via in situ* generation of azomethine ylides. In approximately 7–8 h the reactions delivered the mono cycloaddition products (±)-3Aa (85%) and (±)-3Ab (5%) (Scheme 1). Major products (±)-3Aa were isolated and utilised in the second cycloaddition reaction with a differently substituted isatin and proline in equimolar ratio to produce four mixed pyrrolizidino spiro-oxindolo curcuminoids 4Aa–Ad with 80–85% total yield (Table 1).

**Table 1:** Yields of mixed bis-pyrrolizidino dispiro-oxindole 4(A-F)(a-d) derived from curcumin, isatins and proline

Entry^a^	R^1^	R^2^	R^3^	R^4^	R^5^	R^6^	Product	Yield^b^ (%)
1	H	H	H	H	I	H	4Aa	20
2	H	H	H	H	I	H	4Ab	23
3	H	H	H	H	I	H	4Ac	21
4	H	H	H	H	I	H	4Ad	24
5	H	H	H	H	Me	Me	4Ba	19
6	H	H	H	H	Me	Me	4Bb	22
7	H	H	H	H	Me	Me	4Bc	21
8	H	H	H	H	Me	Me	4Bd	23
9	H	Cl	H	H	Me	H	4Ca	17
10	H	Cl	H	H	Me	H	4Cb	20
11	H	Cl	H	H	Me	H	4Cc	20
12	H	Cl	H	H	Me	H	4Cd	23
13	H	OMe	H	H	F	H	4Da	18
14	H	OMe	H	H	F	H	4Db	22
15	H	OMe	H	H	F	H	4Dc	21
16	H	OMe	H	H	F	H	4Dd	24
17	H	F	H	H	Me	Me	4Ea	18
18	H	F	H	H	Me	Me	4Eb	22
19	H	F	H	H	Me	Me	4Ec	21
20	H	F	H	H	Me	Me	4Ed	23
21	H	Me	Me	H	F	H	4Fa	17
22	H	Me	Me	H	F	H	4Fb	20
23	H	Me	Me	H	F	H	4Fc	20
24	H	Me	Me	H	F	H	4Fd	22

a Unless otherwise noted, the reaction was performed with 1.76 mmol of 3A-F(a) (±), isatins and proline in 50.0 mL of MeOH under reflux for 8 h.

b Determined after isolation.

The products (4Aa–Ad) were characterized from detailed spectral studies. All gave the same pseudo molecular ion peaks at *m*/*z* 895 [M + H] + and 917 [M + Na] + in ESI-Q-TOF MS, indicating them to be isomeric. In the ^13^C NMR spectra, they displayed 45 carbon signals due to the absence of symmetry (present in the previously reported^12^ diastereomers) due to the difference in substitution pattern in the two oxindole rings (originating from isatin substitution).

However, as in the previous publication,^12^ the signals for the aromatic ring of curcumin remained virtually unaltered in the spectra of the products. The chemical shifts for the nuclei belonging to the α,β-unsaturated-diketone part of curcumin were of course distinctly perturbed, with C3/25 and C4/26 suffering profound alteration from downfield to upfield resonance positions.

**Fig. 1:**
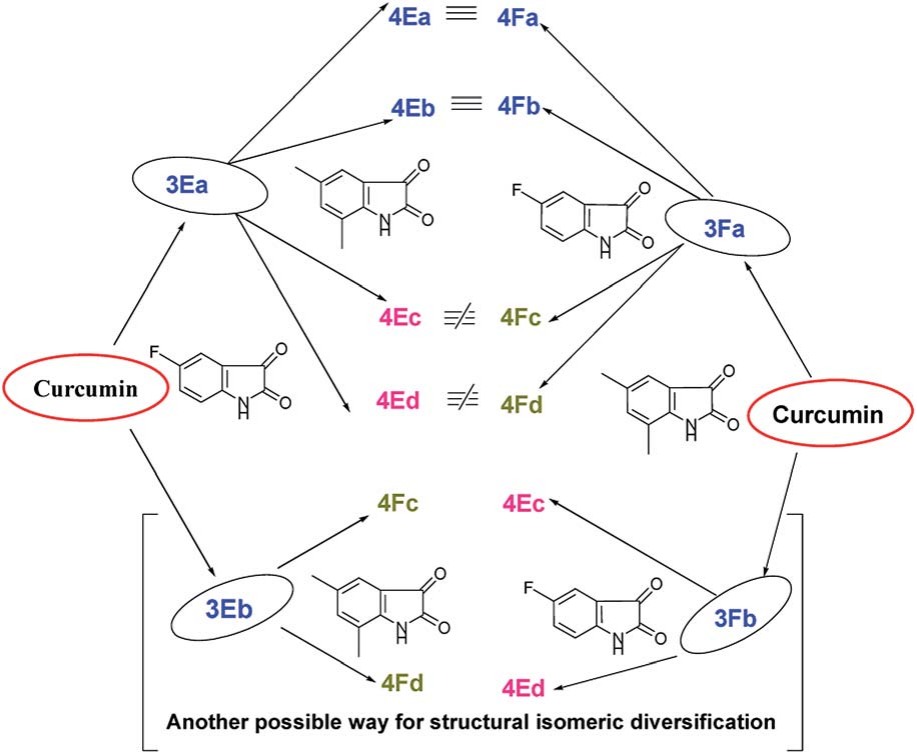
Possible mixed bis-pyrrolizidino dispiro-oxindolo curcuminoids *via* change in sequential addition of any two substituted isatins.

It is obvious from Table 1 that in the entries 17–20 and 21–24 only the sequence of addition of 5-fluoroisatin (2E) and 5,7-dimethylisatin (2F) has changed. This leads to the formation of (±)-4Ea–Ed and (±)-4Fa–Fd. From a simple analysis of the reaction profile and structure of the product it could be easily concluded that 4Ea, 4Eb are identical with 4Fa, 4Fb respectively whereas 4Ec, 4Ed are not identical with 4Fc and 4Fd due to different substitution present in oxindole ring. In fact this was also proved from the detailed NMR spectral comparison of all the products. Detailed possibilities in change of sequential addition with different isatins have been schematically represented using 5-fluoroisatin (2E) and 5,7-dimethylisatin (2F) as model example in Fig. 1.

Because of the possibility of formation of two other diastereomers (±)-4(A-F)c′ and (±)-4(A-F)d′ (Fig. 1), positional isomers of (±)-4(A-F)c and (±)-4(A-F)d due to different substitution in isatin ring, there is the extra advantage of more diversification. But it is necessary to separate (±)-3(A-F)a and 3(A-F)b/(±)-3(A-F)a′ and 3(A-F)b′ synthesized in the first step to avoid formation of complex mixture of compounds in one pot sequence reaction, difficult to separate.

**Scheme 2:**
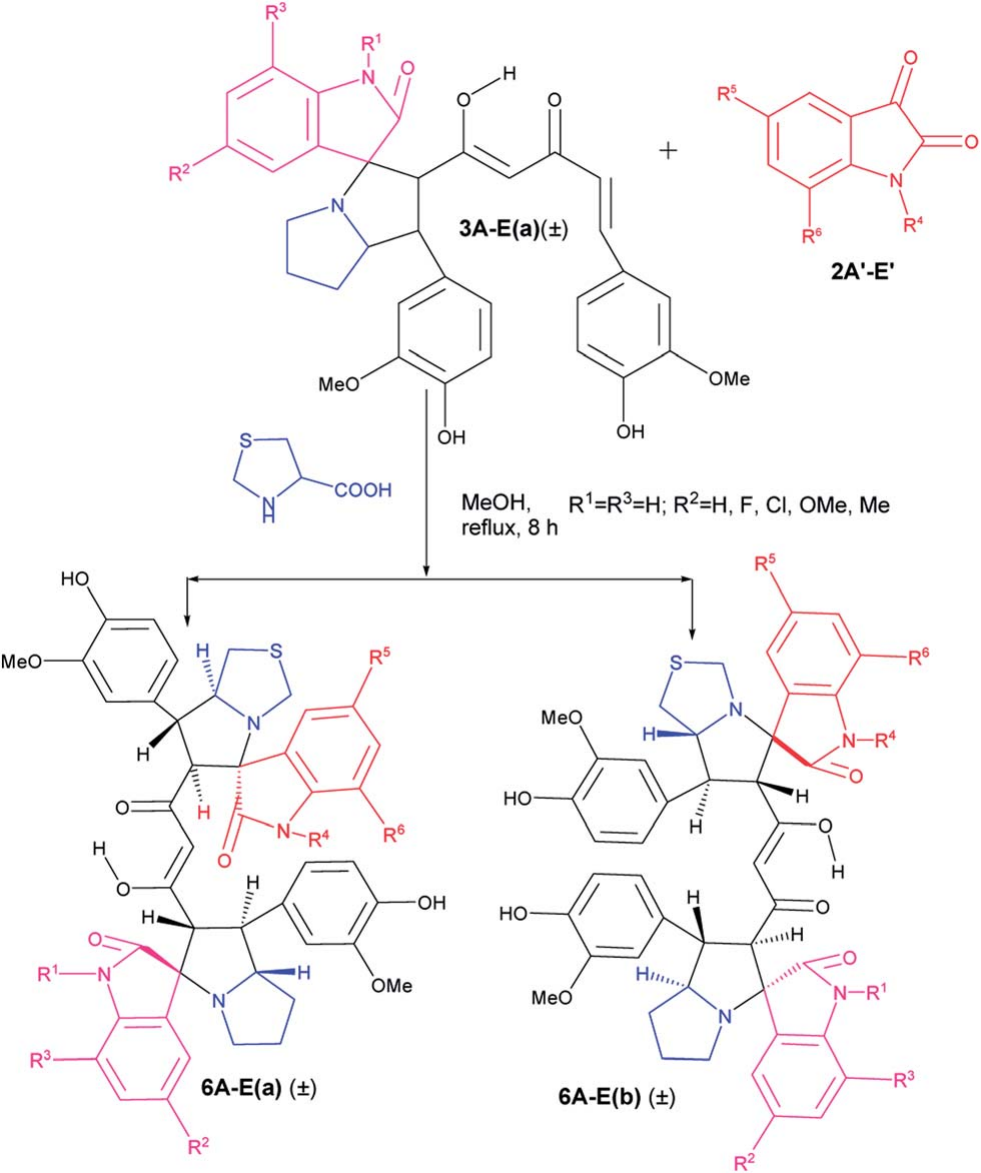
Synthesis of bis-thiopyrrolizidino dispiro-oxindolo curcuminoids.

Following the success in the synthesis of mixed bis-pyrrolizidino dispiro-oxindolo curcuminoids we wanted to synthesize mixed pyrrolizidino-thiopyrrolizidino dispiro-oxindolo curcuminoids (Scheme 2). But to confirm that the reaction indeed proceeds with thioproline (thiazolidine-4-carboxylic acid), we treated curcumin and isatins with thiazolidine-4-carboxylic acid in 1 : 2 : 2 mole ratio. In this case we obtained only two diastereomers (±)-5Aa and (±)-5Ab in around 70% yield in 15–16 h; both are symmetric in nature (confirmed from the 21 carbon peaks present in ^13^C NMR).

**Scheme 3:**
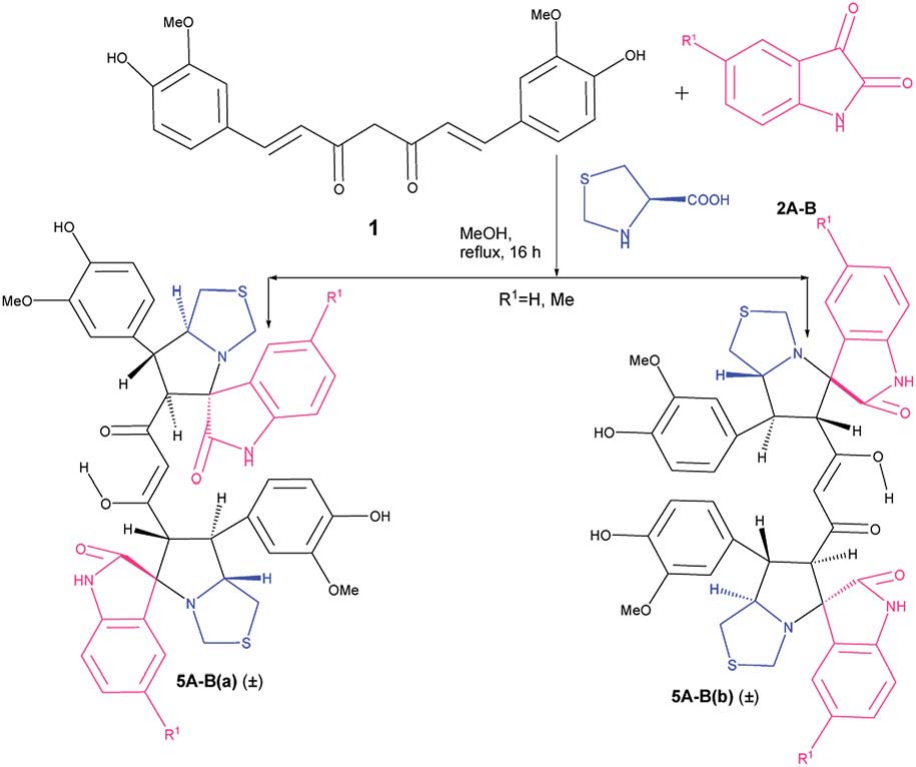
Synthesis of mixed pyrrolizidino-thiopyrrolizidino dispiro-oxindolo curcuminoids.

Similarly, when we used mono cycloaddition product (±)-3Aa with a differently substituted isatin and thiazolidine-4-carboxylic acid in 1 : 1 : 1 mole ratio, we could isolate only two diastereomers of mixed pyrrolizidino-thiopyrrolizidino dispiro-oxindolo curcuminoids after 8 h of reaction (Scheme 3, Table 2).

**Table 2:** Yields of mixed bis-thiopyrrolizidino dispiro-oxindole 6(A-E)(a-b) derived from curcumin, isatins, proline and thioproline

Entry^a^	R^1^	R^2^	R^3^	R^4^	R^5^	R^6^	Product	Yield^b^ (%)
1	H	H	H	H	OMe	H	6Aa	30
2	H	H	H	H	OMe	H	6Ab	35
3	H	F	H	H	I	H	6Ba	32
4	H	F	H	H	I	H	6Bb	37
5	H	Cl	H	H	I	H	6Ca	31
6	H	Cl	H	H	I	H	6Cb	35
7	H	OMe	H	H	Me	Me	6Da	32
8	H	OMe	H	H	Me	Me	6Db	36
9	H	Me	H	H	Me	Me	6Ea	31
10	H	Me	H	H	Me	Me	6Eb	36

a Unless otherwise noted, the reaction was performed with 1.76 mmol of 3A-E(a) (±), isatins and thioproline in 50.0 mL of MeOH under reflux for 8 h.

b Determined after isolation.

In case of the reactions where thioproline was used, the reaction rate and also the yield of the product were lower compared to those with proline perhaps due to lower solubility of thiazolidine-4-carboxylic acid (Table 2) in methanol.

As usual the products were characterized from detailed spectral studies. Both the products (±)-6Aa and (±)-6Ab gave the same peaks at *m*/*z* 817 [M + H]^+^/839 [M + Na]^+^ in the ESI-Q-TOF MS spectrum for the pseudomolecular ions, indicating them to be isomeric. Both showed 45 carbon signals in ^13^C NMR spectra indicating a break in symmetry due to the change in amino acid as well as for the difference in substitution in the isatin ring.

**Fig. 2:**
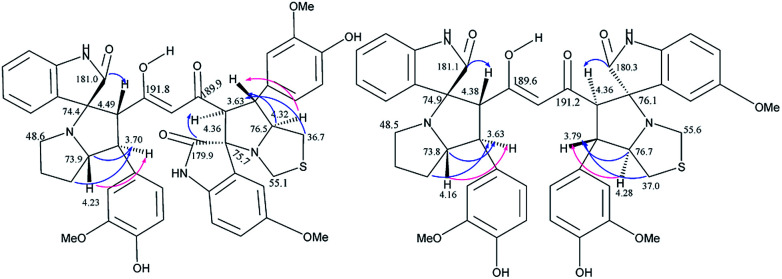
Important correlations of 6Aa and 6Ab [HMBC(
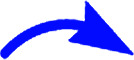
), COSY(
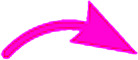
)].

The crucial evidence in support of the proposed structures came from the observed HMBC correlation in the 2D NMR spectrum of 6Aa/Ab between signals of C-10/32 (*i.e.*, the oxindole carbonyls, *δ* 181.0/179.9) and H-3/25 (*δ* 4.49/4.36) and signals of C-5/27 (*i.e.*, the point of attachment with proline, *δ* 73.9/76.5) and C-6/28 (*δ* 30.9/36.7) with H-4/26 (*δ* 3.70/3.63). Further, the COSY relationship between H-4/26 (*δ* 3.70/3.63) and H-5/27 (*δ* 4.23/4.32), coupled with medium to low NOESY cross peaks, strongly supports the mode of additions (Fig. 2).

It is worth mentioning here that the adjacent hydrogens on both side of the diketone functionality of the curcumin unit are anti to each other, whereas they were in syn orientation with the spirocyclic oxindole bond of the respective sides.

**Scheme 4:**
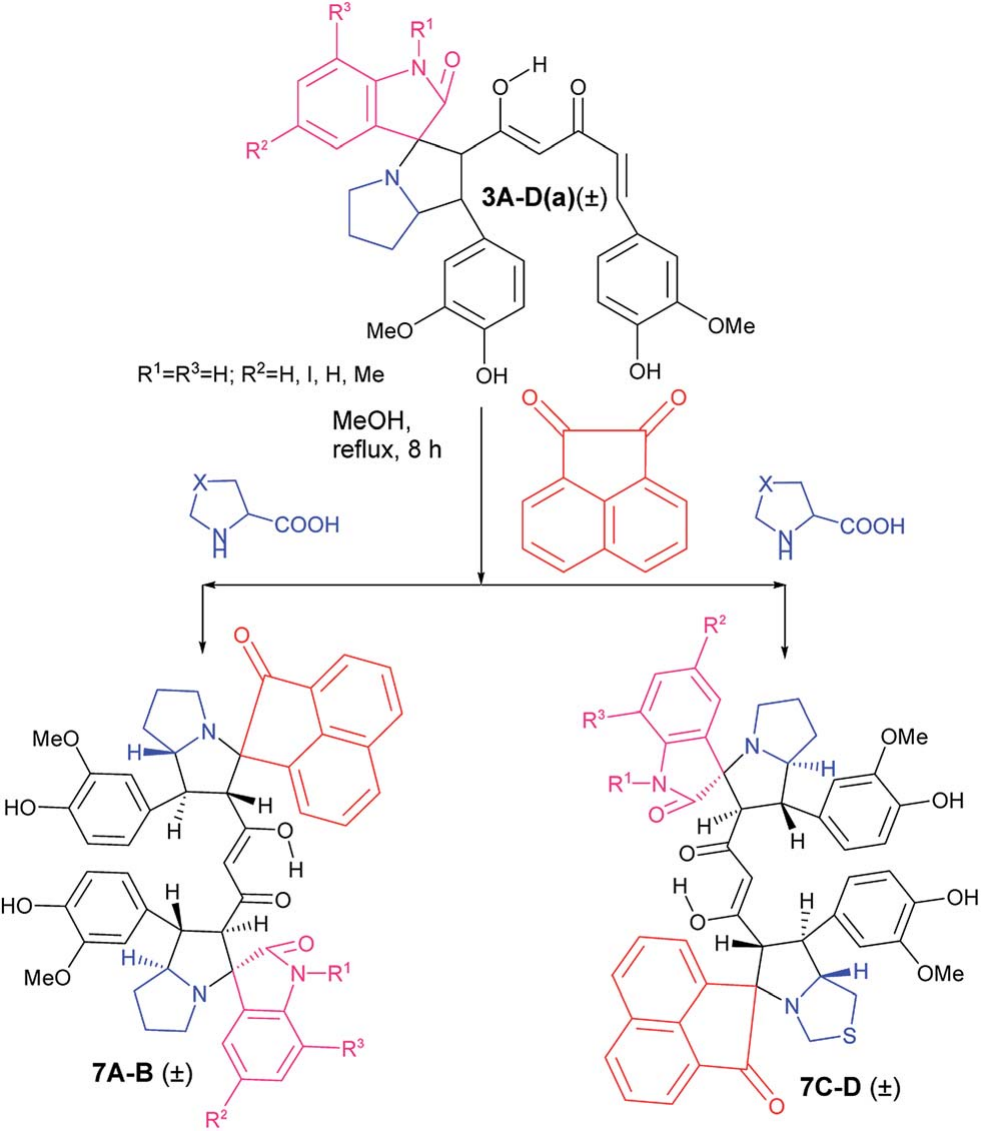
Synthesis of mixed bis-pyrrolizidino/-thiopyrrolizidino dispiro-oxindolo-acenapthylino curcuminoids.

The success achieved in the synthesis of mixed pyrrolizidino-thiopyrrolizidino dispiro-oxindolo curcuminoids prompted us for the synthesis of a new type of diversely substituted product using isatin, acenaphthoquinone and proline/thiproline in various combinations. We used mono cycloaddition product (±)-3Aa, acenaphthoquinone and proline/thioproline in 1 : 1 : 1 molar ratio and isolated only one isomer of mixed bis-pyrrolizidino-dispiro-oxindolo-acenaphthyleno curcuminoids/bis-pyrrolizidino-thiopyrrolizidino dispiro-oxindolo-acenaphthyleno curcuminoids (7A–D) after 8 h of reaction (Scheme 4) with moderate yield (45%, Table 3) although the formation of minor stereoisomers could not be ruled out.

**Table 3:** Yields of mixed bis-pyrrolizidino/-thiopyrrolizidino dispiro-oxindolo-acenaphthylino curcuminoids 7A–D

Entry^a^	R^1^	R^2^	R^3^	X	Product	Yield^b^ (%)
1	H	H	H	–CH_2_	7A	54
2	H	I	H	–CH_2_	7B	56
3	H	H	H	–S	7C	50
4	H	Me	H	–S	7D	49

a Unless otherwise noted, the reaction was performed with 1.76 mmol of 3A-D(a) (±), isatins and proline in 50.0 mL of MeOH under reflux for 8 h.

b Determined after isolation.

**Fig. 3:**
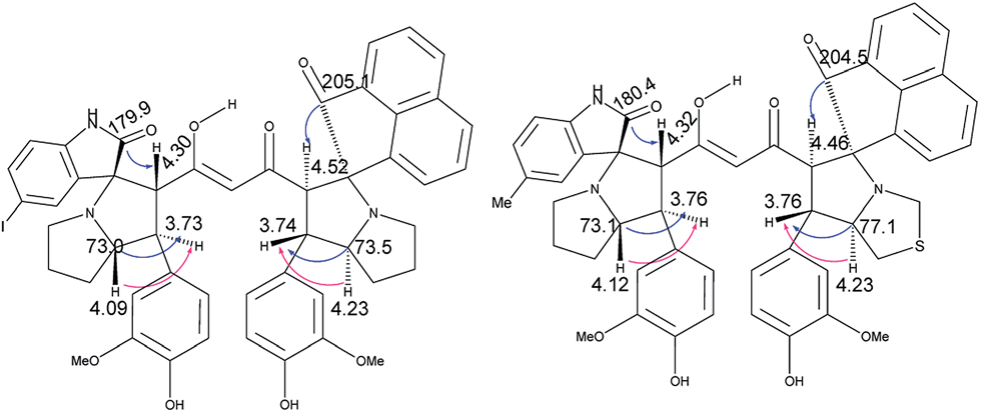
Important correlations of 7Bb and 7Db [HMBC(
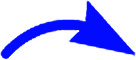
), COSY(
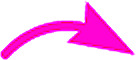
)].

The products were again characterized from detailed spectral studies. The product (±)-7B showed peak at *m*/*z* 930 ascribed to [M + H]^+^ in the ESI-Q-TOF MS spectrum. Crucial evidence in support of the proposed structure came from the observed HMBC correlation in the 2D NMR spectrum between signals of C-10/32, *i.e.*, the oxindole/acenaphthyleno carbonyls (*δ* 179.9/205.1) and H-3/25 (*δ* 4.30/4.52) and those of C-5/27 (*i.e.*, the point of attachment with proline, *δ* 73.0/73.5) with H-4/26 (*δ* 3.73/3.74). Further, the COSY relationship between H-4/26 (*δ* 3.73/3.74) and H-5/27 (*δ* 4.09/4.23) strongly supports the mode of additions (Fig. 3).

## Conclusion

In conclusion, curcumin has been successfully used in a two step sequential 1,3-dipolar azomethine ylide cycloaddition reaction to produce novel mixed bis-pyrrolizidino/-thiopyrrolizidino dispiro-oxindolo/acenaphthyleno curcuminoids. The structures of the products were determined by 1D/2D NMR analysis and MS. The unaltered pharmacophores of curcumin along with the newly generated diversely mixed spiro oxindolo pyrrolizidine moiety might add to the biological effectiveness of the scaffold with increased polarity and solubility.

## Experimental section

### General information

All the compounds evaluated in this work were synthesized in one-pot sequences. Melting points were determined in capillaries and are uncorrected. IR spectra were recorded as KBr pellets using a JASCO 410 FTIR spectrometer. The NMR spectra were recorded using a Bruker 600 DPX spectrometer operating at 600 MHz for ^1^H and 150 MHz for ^13^C in pyridine-*d*_5_ and the chemical shifts are reported in *δ* units. Mass spectra (positive mode) were obtained on a ESI-Q-TOF micro mass spectrometer in the electrospray ionization mode.

Curcumin was isolated from *Curcuma longa* in the usual way. Isatins, acenaphthoquinone and α-amino acids were purchased from Alfa-Aesar Company. All other solvents and chromatographic absorbents were procured from E. Merck (Germany) and SRL (India) Ltd. unless otherwise indicated. Thin layer chromatography was performed on pre-coated silica gel 60 F_254_ aluminum sheets (E. Merck, Germany) using the solvent system 5% MeOH in CHCl_3_ and spots were observed using UV irradiation and iodine. Compounds were separated using AKROS – “Automatic TLC Smart Flash” of Yamazen Corporation.

### Typical experimental procedure for synthesis of mono cycloaddition products (±)-3Aa

A mixture of 1 (13.6 mmol, 5 g), isatin (13.6 mmol, 2.00 g) and proline (13.6 mmol, 1.56 g) was taken in a round bottom flask, dissolved in 200 mL methanol, and heated to reflux for specified time period. After completion of the reaction as evident from TLC, the solvent was removed and the crude product was subjected to flash chromatography using increasing concentration of methanol in chloroform as eluant. The product was crystallized from chloroform–methanol mixture (85%, 6.56 g).

### Typical experimental procedure for synthesis of mixed cycloaddition products

A mixture of (±)-3Aa (1.76 mmol, 1 g), 5-iodoisatin (1.76 mmol, 481 mg) and proline (1.76 mmol, 205 mg) or thioproline (1.76 mmol, 235 mg) was taken in a round bottom flask, dissolved in 50 mL methanol, and heated to reflux for specified time period. The solvent was removed after completion of the reaction (as evident from TLC), and the crude product was subjected to flash chromatography using increasing concentration of methanol in chloroform as eluant to isolate mixed pyrrolizidino/pyrrilizidino-thiopyrrolizidino dispiro oxindolo curcuminoids. The product was crystallized from chloroform-methanol mixture.

### Spectral data of compound 4Aa (±)

Obtained as white solid; yield: 20% (314 mg); mp: 219–221 °C; *R*_f_ 0.49 (6% MeOH in CHCl_3_); UV absorption maximas (*λ*_max_ nm, methanol) 258, 213; IR (KBr, *ν*_max_ cm^−1^): 3376, 2960, 2869, 1722, 1612; ^1^H NMR (*Py-d*_5_): *δ* 11.51 (1H, s, –NH), 11.19 (1H, s, –NH), 7.97 (1H, s), 7.75 (1H, d, *J* = 7.8 Hz), 7.38 (2H, m), 7.29 (2H, m), 7.22 (2H, m), 7.13 (1H, d, *J* = 1.2 Hz), 6.99 (1H, t, *J* = 7.8 Hz), 6.96 (1H, d, *J* = 6.6 Hz), 6.87 (1H, d, *J* = 8.4 Hz), 6.75 (1H, dd, *J* = 1.8, 8.4 Hz), 5.80 (1H, s), 4.59 (1H, d, *J* = 12.6 Hz), 4.43 (1H, d, *J* = 12.6 Hz), 4.21 (2H, m), 3.82 (3H, s), 3.75 (1H, m), 3.66 (3H, s), 3.64 (1H, m), 2.74 (2H, m), 2.62 (2H, m), 1.87 (3H, m), 1.82 (2H, m), 1.70 (3H, m). ^13^C NMR (*Py-d*_5_): *δ* 191.8 (C=O), 190.8 (C=O), 181.1 (C=O), 180.2 (C=O), 149.6 (C), 149.2 (C), 147.7 (C), 147.6 (C), 143.9 (C), 143.6 (C), 138.9 (CH), 135.8 (CH), 131.9 (C), 130.9 (C), 129.7 (CH), 129.5 (C), 127.1 (C), 126.9 (CH), 121.7 (2 × CH), 121.0 (CH), 117.4 (CH), 117.1 (CH), 113.2 (CH), 111.7 (CH), 111.4 (CH), 110.6 (CH), 101.4 (CH), 84.7 (C), 74.49 (C), 74.46 (C), 74.42 (CH), 73.5 (CH), 65.2 (CH), 63.6 (CH), 56.3 (OMe), 56.1 (OMe), 52.8 (CH), 52.6 (CH), 48.6 (CH_2_), 48.3 (CH_2_), 31.13 (CH_2_), 31.06 (CH_2_), 28.1 (CH_2_), 27.8 (CH_2_). MS [ESI-MS, positive mode]: found *m*/*z* 895 [M + H]^+^. HRMS [ESI-MS, positive mode]: MF: C_45_H_43_N_4_O_8_I; found *m*/*z* 917.2042 [M + Na]^+^ [calcd. 917.2023].

### Spectral data of compound 4Ab (±)

Obtained as white solid; yield: 23% (362 mg); mp: 192–194 °C; *R*_f_ 0.43 (6% MeOH in CHCl_3_); UV absorption maximas (*λ*_max_ nm, methanol) 201; IR (KBr, *ν*_max_ cm^−1^): 3383, 2960, 2872, 1718, 1611. ^1^H NMR (*Py-d*_5_): *δ* 12.18 (1H, s, –NH), 11.81 (1H, s, –NH), 7.91 (1H, d, *J* = 1.8 Hz), 7.63 (1H, dd, *J* = 1.8, 8.4 Hz), 7.25 (2H, m), 7.19 (1H, m), 7.17 (2H, d, *J* = 7.8 Hz), 7.07 (1H, d, *J* = 1.8 Hz), 7.00 (2H, m), 6.92 (1H, d, *J* = 7.2 Hz), 6.85 (2H, m), 5.94 (1H, s), 4.42 (2H, t, *J* = 12.0 Hz), 4.20 (1H, m), 4.10 (1H, m), 3.85 (1H, m), 3.79 (3H, s), 3.70 (1H, m), 3.67 (3H, s), 2.69 (2H, m), 2.61 (2H, m), 1.86 (2H, m), 1.79 (1H, m), 1.69 (5H, m). ^13^C NMR (*Py-d*_5_): *δ* 191.6 (C=O), 189.7 (C=O), 181.2 (C=O), 180.5 (C=O), 149.2 (C), 149.1 (C), 147.52 (C), 147.50 (C), 143.9 (C), 143.5 (C), 138.8 (CH), 135.8 (CH), 131.4 (C), 131.2 (C), 129.8 (CH), 129.5 (C), 127.1 (CH), 126.6 (C), 121.8 (CH), 120.7 (2 × CH), 117.2 (CH), 117.0 (CH), 113.0 (CH), 112.2 (CH), 112.1 (CH), 110.6 (CH), 101.3 (CH), 84.7 (C), 74.9 (2 × C), 73.7 (CH), 73.6 (CH), 65.1 (CH), 64.3 (CH), 56.2 (OMe), 56.1 (OMe), 52.5 (CH), 51.8 (CH), 48.5 (CH_2_), 48.2 (CH_2_), 31.4 (CH_2_), 31.3 (CH_2_), 28.4 (CH_2_), 28.0 (CH_2_). MS [ESI-MS, positive mode]: found *m*/*z* 895 [M + H]^+^, 917 [M + Na]^+^. HRMS [ESI-MS, positive mode]: MF: C_45_H_43_N_4_O_8_I; found *m*/*z* 917.2034 [M + Na]^+^[calcd. 917.2023].

### Spectral data of compound 4Ac (±)

Obtained as white solid; yield: 21% (330 mg); mp: 175–177 °C; *R*_f_ 0.39 (6% MeOH in CHCl_3_); UV absorption maximas (*λ*_max_ nm, methanol) 283, 231; ^1^H NMR (*Py-d*_5_): *δ* 11.93 (1H, s, –NH), 11.54 (1H, s, –NH), 8.26 (1H, s), 7.60 (1H, m), 7.35 (1H, s), 7.28 (1H, t, *J* = 7.8 Hz), 7.23 (2H, m), 7.16 (1H, m), 7.05 (2H, m), 6.93 (1H, d, *J* = 8.4 Hz), 6.89 (2H, m), 6.54 (1H, d, *J* = 7.8 Hz), 6.05 (1H, s), 4.71 (1H, m), 4.62 (1H, d, *J* = 12 Hz), 4.33 (1H, m), 4.11 (1H, d, *J* = 12 Hz), 3.95 (1H, m), 3.71 (3H, s), 3.55 (3H, s), 3.23 (1H, m), 2.83 (2H, m), 1.96 (1H, m), 1.90 (2H, m), 1.74 (2H, m), 1.64 (2H, m), 1.40 (2H, m). ^13^C NMR (*Py-d*_5_): *δ* 196.3 (C=O), 194.5 (C=O), 181.2 (C=O), 179.4 (C=O), 149.3 (C), 148.3 (C), 147.7 (C), 147.5 (C), 144.6 (C), 144.1 (C), 138.7 (CH), 136.2 (CH), 131.4 (C), 131.0 (C), 129.9 (CH), 127.7 (CH), 126.9 (C), 126.8 (C), 124.1 (CH), 121.7 (CH), 121.4 (CH), 117.1 (CH), 116.5 (CH), 113.0 (CH), 112.6 (CH), 112.3 (CH), 110.7 (CH), 102.9 (CH), 84.3 (C), 76.5 (C), 75.1 (C), 73.8 (CH), 65.7 (CH), 64.6 (CH), 56.1 (OMe), 55.7 (OMe), 53.3 (CH), 52.7 (CH), 52.0 (CH), 51.5 (CH_2_), 48.5 (CH_2_), 31.3 (CH_2_), 29.9 (CH_2_), 28.2 (CH_2_), 26.8 (CH_2_). MS [ESI-MS, positive mode]: found *m*/*z* 895 [M + H]^+^, 917 [M + Na]^+^. HRMS [ESI-MS, positive mode]: MF: C_45_H_43_N_4_O_8_I; found *m*/*z* 917.2017 [M + Na]^+^ [calcd. 917.2023].

### Spectral data of compound 4Ad (±)

Obtained as white solid; yield: 24% (378 mg); mp: 218–220 °C; *R*_f_ 0.38 (6% MeOH in CHCl_3_); UV absorption maximas (*λ*_max_ nm, methanol) 262, 212; IR (KBr, *ν*_max_ cm^−1^): 3391, 2960, 1720, 1613; ^1^H NMR (*Py-d*_5_): *δ* 11.77 (1H, s, –NH), 11.51 (1H, s, –NH), 8.33 (1H, s), 7.67 (1H, d, *J* = 7.2 Hz), 7.60 (1H, d, *J* = 8.4 Hz), 7.36 (1H, s), 7.30 (1H, t, *J* = 7.8 Hz), 7.20 (1H, m), 7.15 (2H, m), 7.01 (1H, d, *J* = 7.8 Hz), 6.97 (1H, m), 6.92 (1H, m), 6.89 (1H, s), 6.54 (1H, d, *J* = 8.4 Hz), 5.97 (1H, s), 4.96 (1H, m), 4.67 (1H, q, *J* = 8.4 Hz), 4.59 (1H, d, *J* = 12.6 Hz), 4.37 (1H, m), 4.23 (1H, d, *J* = 12.0 Hz), 3.98 (1H, m), 3.70 (3H, s), 3.56 (3H, s), 3.15 (1H, m), 2.88 (1H, m), 2.74 (2H, m), 1.96 (1H, m), 1.89 (2H, m), 1.77 (1H, m), 1.67 (1H, m), 1.61 (1H, m), 1.51 (1H, m), 1.23 (1H, m). ^13^C NMR (*Py-d*_5_): *δ* 197.7 (C=O), 186.0 (C=O), 181.2 (C=O), 179.5 (C=O), 149.3 (C), 148.3 (C), 147.7 (C), 147.5 (C), 144.6 (C), 144.0 (C), 138.7 (CH), 136.0 (CH), 131.5 (C), 131.0 (C), 129.9 (CH), 128.0 (CH), 127.2 (C), 126.9 (C), 121.7 (CH), 121.4 (CH), 121.3 (CH), 117.1 (CH), 116.5 (CH), 113.3 (CH), 112.6 (CH), 112.4 (CH), 110.8 (CH), 102.9 (CH), 84.3 (C), 76.4 (C), 75.4 (C), 73.7 (CH), 65.6 (CH), 64.5 (CH), 56.1 (OMe), 55.8 (OMe), 53.8 (CH), 52.6 (CH), 51.5 (CH_2_), 51.1 (CH), 48.6 (CH_2_), 31.3 (CH_2_), 29.4 (CH_2_), 28.2 (CH_2_), 26.8 (CH_2_). MS [ESI-MS, positive mode]: found *m*/*z* 895 [M + H]^+^, 917 [M + Na]^+^. HRMS [ESI-MS, positive mode]: MF: C_45_H_43_N_4_O_8_I; found *m*/*z* 895.2209 [M + H]^+^[calcd. 895.2204].

### Spectral data of compound 4Ba (±)

Obtained as white solid; yield: 19% (266 mg); mp: 172–174 °C; *R*_f_ 0.50 (6% MeOH in CHCl_3_); UV absorption maximas (*λ*_max_ nm, methanol) 283, 231; ^1^H NMR (*Py-d*_5_): *δ* 10.94 (1H, s, –NH), 10.91 (1H, s, –NH), 7.31 (1H, d, *J* = 7.8 Hz), 7.27 (3H, m), 7.21 (1H, s), 7.18 (2H, m), 7.12 (1H, d, *J* = 7.8 Hz), 6.92 (2H, m), 6.88 (1H, d, *J* = 7.8 Hz), 6.77 (1H, s), 5.77 (1H, s), 4.50 (1H, d, *J* = 12.0 Hz), 4.40 (1H, d, *J* = 12.0 Hz), 4.31 (1H, m), 4.21 (1H, m), 3.82 (1H, m), 3.77 (3H, s), 3.74 (1H, m), 3.71 (3H, s), 2.80 (2H, m), 2.65 (1H, m), 2.60 (1H, m), 2.26 (3H, s), 2.22 (3H, s), 1.86 (4H, m), 1.78 (2H, m), 1.68 (2H, m). ^13^C NMR (*Py-d*_5_): *δ* 192.1 (C=O), 190.3 (C=O), 181.3 (C=O), 181.1 (C=O), 149.24 (C), 149.18 (C), 147.5 (C), 147.4 (C), 143.5 (C), 139.9 (C), 131.9 (CH), 131.8 (C), 131.5 (C), 130.6 (C), 129.7 (CH), 127.3 (CH), 126.6 (C), 126.2 (C), 125.5 (CH), 121.6 (CH), 121.13 (CH), 121.07 (CH), 119.4 (C), 117.2 (CH), 117.1 (CH), 112.2 (CH), 111.8 (CH), 110.6 (CH), 101.6 (CH), 74.9 (C), 74.5 (C), 73.7 (CH), 73.5 (CH), 65.1 (CH), 64.2 (CH), 56.2 (OMe), 56.1 (OMe), 52.53 (CH), 52.50 (CH), 48.6 (CH_2_), 48.4 (CH_2_), 31.2 (CH_2_), 30.9 (CH_2_), 28.1 (CH_2_), 27.6 (CH_2_), 21.2 (CH_3_), 17.2 (CH_3_). MS [ESI-MS, positive mode]: found *m*/*z* 797 [M + H]^+^. HRMS [ESI-MS, positive mode]: MF: C_47_H_48_N_4_O_8_; found *m*/*z* 819.3361 [M + Na]^+^ [calcd. 819.3370].

### Spectral data of compound 4Bb (±)

Obtained as white solid; yield: 22% (308 mg); mp: 188–190 °C; *R*_f_ 0.44 (6% MeOH in CHCl_3_); UV absorption maximas (*λ*_max_ nm, methanol) 296, 231; ^1^H NMR (*Py-d*_5_): *δ* 11.85 (1H, s, –NH), 11.73 (1H, s, –NH), 7.25 (2H, m), 7.21 (1H, m), 7.18 (2H, d, *J* = 7.8 Hz), 7.11 (2H, m), 7.03 (1H, d, *J* = 7.8 Hz), 6.97 (1H, d, *J* = 7.8 Hz), 6.91 (2H, m), 6.75 (1H, s), 6.00 (1H, s), 4.48 (1H, d, *J* = 12.0 Hz), 4.41 (1H, d, *J* = 12.0 Hz), 4.18 (2H, m), 3.87 (1H, m), 3.74 (3H, s), 3.71 (3H, s), 3.66 (1H, m), 2.80 (1H, m), 2.73 (1H, m), 2.66 (1H, m), 2.61 (1H, m), 2.31 (3H, s), 2.16 (3H, s), 1.90 (1H, m), 1.83 (3H, m), 1.71 (3H, m), 1.63 (1H, m) ^13^C NMR (*Py-d*_5_): *δ* 191.7 (C=O), 190.1 (C=O), 181.6 (C=O), 181.2 (C=O), 149.3 (C), 149.2 (C), 147.63 (C), 147.57 (C), 143.9 (C), 140.0 (C), 131.9 (CH), 131.7 (C), 131.5 (C), 130.7 (C), 129.8 (CH), 127.1 (CH), 126.7 (C), 126.4 (C), 125.3 (CH), 121.6 (CH), 120.9 (CH), 120.8 (CH), 119.4 (C), 117.1 (2 × CH), 112.2 (CH), 112.0 (CH), 110.5 (CH), 101.0 (CH), 75.1 (C), 74.8 (C), 73.8 (CH), 73.7 (CH), 64.7 (CH), 64.2 (CH), 56.12 (OMe), 56.09 (OMe), 52.4 (CH), 52.2 (CH), 48.6 (CH_2_), 48.3 (CH_2_), 31.5 (CH_2_), 31.2 (CH_2_), 28.3 (CH_2_), 27.9 (CH_2_), 21.4 (CH_3_), 17.3 (CH_3_). MS [ESI-MS, positive mode]: found *m*/*z* 797 [M + H]^+^. HRMS [ESI-MS, positive mode]: MF: C_47_H_48_N_4_O_8_; found *m*/*z* 819.3351 [M + Na]^+^ [calcd. 819.3370].

### Spectral data of compound 4Bc (±)

Obtained as white solid; yield: 21% (294 mg); mp: 169–171 °C; *R*_f_ 0.40 (6% MeOH in CHCl_3_); ^1^H NMR (DMSO-*d*_6_): *δ* 10.36 (1H, s, –NH), 9.93 (1H, s, –NH), 8.84 (1H, d, *J* = 4.2 Hz), 7.46 (1H, d, *J* = 7.2 Hz), 7.17 (1H, m), 7.02 (1H, s), 6.95 (1H, m), 6.74 (4H, m), 6.41 (1H, m), 6.26 (1H, s), 6.18 (1H, d, *J* = 6.6 Hz), 5.62 (1H, s), 4.24 (1H, m), 4.02 (1H, m), 3.78 (3H, s), 3.72 (2H, m), 3.56 (2H, m), 2.97 (1H, m), 2.42 (2H, m), 2.36 (1H, m), 2.25 (3H, s), 2.07 (3H, m), 1.96 (3H, s), 1.82 (2H, m), 1.68 (3H, m), 1.40 (1H, m), 1.15 (1H, m), 0.86 (1H, m). ^13^C NMR (DMSO-*d*_6_): *δ* 197.2 (C=O), 183.6 (C=O), 179.2 (C=O), 178.7 (C=O), 147.6 (C), 146.6 (C), 145.4 (C), 145.1 (C), 142.3 (C), 139.3 (C), 130.8 (CH), 130.3 (C), 129.9 (C), 129.1 (CH), 127.0 (CH), 126.5 (C), 126.3 (C), 125.2 (C), 124.6 (CH), 120.7 (CH), 119.9 (CH), 119.7 (CH), 118.2 (C), 115.6 (CH), 114.9 (CH), 111.8 (2 × CH), 109.8 (CH), 101.6 (CH), 74.8 (C), 73.6 (C), 72.5 (CH), 64.1 (CH), 62.3 (CH), 55.6 (OMe), 54.9 (OMe), 52.6 (CH), 50.4 (CH), 50.3 (CH_2_), 48.9 (CH), 47.4 (CH_2_), 30.1 (CH_2_), 28.0 (CH_2_), 27.2 (CH_2_), 25.6 (CH_2_), 20.7 (CH_3_), 16.2 (CH_3_). MS [ESI-MS, positive mode]: found *m*/*z* 797 [M + H]^+^. HRMS [ESI-MS, positive mode]: MF: C_47_H_48_N_4_O_8_; found *m*/*z* 819.3395 [M + Na]^+^ [calcd. 819.3370].

### Spectral data of compound 4Bd (±)

Obtained as white solid; yield: 23% (322 mg); mp: 170–172 °C; *R*_f_ 0.39 (6% MeOH in CHCl_3_); UV absorption maximas (*λ*_max_ nm, methanol) 283, 205; IR (KBr, *ν*_max_ cm^−1^): 3390, 2960, 1713, 1618; ^1^H NMR (*Py-d*_5_): *δ* 11.84 (1H, s, –NH), 11.17 (1H, s, –NH), 7.69 (1H, d, *J* = 7.2 Hz), 7.43 (1H, s), 7.38 (1H, s), 7.31 (1H, t, *J* = 7.2 Hz), 7.22 (1H, d, *J* = 7.2 Hz), 7.18 (1H, d, *J* = 1.2 Hz), 7.16 (1H, t, *J* = 7.8 Hz), 7.06 (1H, d, *J* = 7.8 Hz), 6.93 (3H, m), 6.81 (1H, s), 6.04 (1H, s), 4.99 (1H, m), 4.73 (1H, q, *J* = 7.8 Hz), 4.64 (1H, d, *J* = 6.6 Hz), 4.39 (1H, m), 4.25 (1H, d, *J* = 12 Hz), 3.99 (1H, dd, *J* = 9.6, 12 Hz), 3.75 (3H, s), 3.56 (3H, s), 3.23 (1H, m), 2.86 (1H, m), 2.79 (1H, t, *J* = 7.2 Hz), 2.73 (1H, m), 2.38 (3H, s), 2.01 (3H, s), 1.97 (1H, m), 1.90 (2H, m), 1.76 (2H, m), 1.65 (1H, m), 1.59 (1H, m), 1.30 (1H, m). ^13^C NMR (*Py-d*_5_): *δ* 198.0 (C=O), 186.2 (C=O), 181.4 (C=O), 180.6 (C=O), 149.3 (C), 148.3 (C), 147.6 (C), 147.3 (C), 143.9 (C), 141.0 (C), 131.8 (CH), 131.6 (2 × C), 130.9 (C), 130.0 (CH), 128.0 (CH), 127.9 (C), 126.9 (C), 125.4 (CH), 121.8 (CH), 121.7 (CH), 121.5 (CH), 119.4 (C), 117.1 (CH), 116.4 (CH), 113.2 (CH), 112.4 (CH), 110.9 (CH), 102.9 (CH), 76.5 (C), 75.4 (C), 73.8 (CH), 65.5 (CH), 64.4 (CH), 56.2 (OMe), 55.7 (OMe), 54.3 (CH), 52.6 (CH), 51.5 (CH_2_), 50.9 (CH), 48.6 (CH_2_), 31.3 (CH_2_), 29.4 (CH_2_), 28.2 (CH_2_), 26.8 (CH_2_), 21.4 (CH_3_), 17.1 (CH_3_). MS [ESI-MS, positive mode]: found *m*/*z* 797 [M + H]^+^. HRMS [ESI-MS, positive mode]: MF: C_47_H_48_N_4_O_8_; found *m*/*z* 819.3378 [M + Na]^+^ [calcd. 819.3370].

### Spectral data of compound 4Ca (±)

Obtained as white solid; yield: 17% (230 mg); mp: 183–185 °C; *R*_f_ 0.48 (6% MeOH in CHCl_3_); IR (KBr, *ν*_max_ cm^−1^): 3391, 2961, 2868, 1722, 1615; ^1^H NMR (*Py-d*_5_): *δ* 11.28 (1H, s, –NH), 10.88 (1H, s, –NH), 7.59 (1H, dd, *J* = 4.8, 6.6 Hz), 7.34 (1H, s), 7.24 (2H, m), 7.22 (1H, m), 7.18 (2H, m), 7.00 (3H, m), 6.85 (2H, m), 5.70 (1H, s), 4.52 (1H, d, *J* = 12.0 Hz), 4.37 (1H, d, *J* = 12.0 Hz), 4.29 (1H, m), 4.20 (1H, m), 3.81 (2H, m), 3.73 (3H, s), 3.70 (3H, s), 2.83 (1H, m), 2.77 (1H, m), 2.62 (2H, m), 2.16 (3H, s), 1.85 (4H, m), 1.78 (2H, m), 1.69 (2H, m). ^13^C NMR (*Py-d*_5_): *δ* 191.2 (C=O), 190.9 (C=O), 181.0 (C=O), 180.8 (C=O), 149.22 (C), 149.16 (C), 147.5 (C), 147.4 (C), 142.4 (C), 141.2 (C), 131.6 (C), 131.5 (C), 130.8 (C), 130.2 (CH), 129.7 (CH), 128.6 (C), 128.0 (CH), 127.5 (CH), 126.7 (C), 126.6 (C), 120.8 (2 × CH), 117.1 (2 × CH), 112.2 (CH), 111.8 (CH), 111.7 (CH), 110.4 (CH), 101.7 (CH), 74.7 (C), 74.5 (C), 73.7 (CH), 73.2 (CH), 65.1 (CH), 64.0 (CH), 56.1 (OMe), 56.0 (OMe), 52.2 (2 × CH), 48.54 (CH_2_), 48.51 (CH_2_), 31.0 (CH_2_), 30.6 (CH_2_), 27.9 (CH_2_), 27.7 (CH_2_), 21.2 (CH_3_). MS [ESI-MS, positive mode]: found *m*/*z* 817 [M + H]^+^. HRMS [ESI-MS, positive mode]: MF: C_46_H_45_N_4_O_8_Cl; found *m*/*z* 839.2817 [M + Na]^+^ [calcd. 839.2824].

### Spectral data of compound 4Cb (±)

Obtained as white solid; yield: 20% (271 mg); mp: 208–210 °C; *R*_f_ 0.43 (6% MeOH in CHCl_3_); IR (KBr, *ν*_max_ cm^−1^): 3382, 1711, 1613, 1516; ^1^H NMR (*Py-d*_5_): *δ* 12.14 (1H, s, –NH), 11.77 (1H, s, –NH), 7.59 (1H, m), 7.35 (1H, s), 7.20 (1H, m), 7.15 (2H, d, *J* = 8.4 Hz), 7.10 (1H, d, *J* = 1.8 Hz), 7.05 (1H, d, *J* = 1.2 Hz), 6.94 (1H, d, *J* = 7.8 Hz), 6.86 (1H, d, *J* = 7.8 Hz), 6.84 (1H, d, *J* = 1.2 Hz), 6.82 (1H, d, *J* = 7.8 Hz), 6.80 (1H, dd, *J* = 1.2, 8.4 Hz), 5.92 (1H, s), 4.39 (2H, d, *J* = 12.0 Hz), 4.20 (1H, m), 4.12 (1H, m), 3.92 (1H, m), 3.82 (1H, m), 3.66 (3H, s), 3.65 (3H, s), 2.82 (1H, m), 2.65 (3H, m), 2.11 (3H, s), 1.85 (5H, m), 1.71 (3H, m). ^13^C NMR (*Py-d*_5_): *δ* 191.2 (C=O), 189.8 (C=O), 181.2 (C=O), 180.9 (C=O), 149.0 (2 × C), 147.42 (C), 147.36 (C), 142.6 (C), 141.2 (C), 131.6 (C), 131.2 (C), 130.7 (C), 130.3 (CH), 129.9 (CH), 128.7 (C), 128.2 (CH), 127.5 (CH), 126.7 (C), 126.6 (C), 120.35 (CH), 120.31 (CH), 116.99 (CH), 116.97 (CH), 112.5 (CH), 112.3 (CH), 111.7 (CH), 110.3 (CH), 101.5 (CH), 75.25 (C), 75.22 (C), 73.7 (CH), 73.4 (CH), 65.3 (CH), 64.9 (CH), 56.0 (2 × OMe), 51.8 (CH), 51.6 (CH), 48.3 (CH_2_), 48.2 (CH_2_), 31.4 (2 × CH_2_), 28.4 (CH_2_), 28.3 (CH_2_), 21.3 (CH_3_). MS [ESI-MS, positive mode]: found *m*/*z* 817 [M + H]^+^, 839 [M + Na]^+^. HRMS [ESI-MS, positive mode]: MF: C_46_H_45_N_4_O_8_Cl; found *m*/*z* 839.2820 [M + Na]^+^ [calcd. 839.2824].

### Spectral data of compound 4Cc (±)

Obtained as white solid; yield: 20% (271 mg); mp: 174–176 °C; *R*_f_ 0.36 (6% MeOH in CHCl_3_); ^1^H NMR (*Py-d*_5_): *δ* 12.14 (1H, s, –NH), 11.20 (1H, s, –NH), 7.90 (1H, d, *J* = 1.8 Hz), 7.53 (1H, s), 7.39 (1H, dd, *J* = 1.8, 7.8 Hz), 7.30 (1H, m), 7.17 (1H, d, *J* = 7.8 Hz), 7.07 (1H, d, *J* = 8.4 Hz), 7.04 (1H, d, *J* = 8.4 Hz), 7.01 (1H, d, *J* = 8.4 Hz), 6.91 (1H, m), 6.86 (2H, m), 6.67 (1H, d, *J* = 7.8 Hz), 6.21 (1H, s), 5.04 (1H, m), 4.86 (1H, m), 4.66 (1H, d, *J* = 12.6 Hz), 4.31 (1H, m), 4.25 (1H, d, *J* = 12.0 Hz), 4.01 (1H, m), 3.68 (3H, s), 3.51 (3H, s), 3.34 (1H, m), 2.88 (1H, m), 2.82 (1H, m), 2.74 (1H, m), 2.34 (3H, s), 1.94 (3H, m), 1.88 (1H, m), 1.78 (4H, m). ^13^C NMR (*Py-d*_5_): *δ* 197.5 (C=O), 187.4 (C=O), 181.0 (C=O), 180.2 (C=O), 149.3 (C), 148.2 (C), 147.7 (C), 147.4 (C), 143.0 (C), 142.4 (C), 131.1 (C), 131.0 (C), 130.2 (CH), 130.0 (CH), 129.2 (C), 128.4 (C), 128.1 (CH), 127.7 (CH), 127.3 (C), 127.0 (C), 121.6 (CH), 121.2 (CH), 117.2 (CH), 116.4 (CH), 112.9 (CH), 112.4 (CH), 112.0 (CH), 110.2 (CH), 103.0 (CH), 76.6 (C), 75.2 (C), 74.0 (CH), 65.9 (CH), 64.6 (CH), 56.1 (OMe), 55.7 (OMe), 53.9 (CH), 52.4 (CH), 52.1 (CH), 51.5 (CH_2_), 48.5 (CH_2_), 31.3 (CH_2_), 29.1 (CH_2_), 28.4 (CH_2_), 27.0 (CH_2_), 21.5 (CH_3_). MS [ESI-MS, positive mode]: found *m*/*z* 817 [M + H]^+^, 839 [M + Na]^+^. HRMS [ESI-MS, positive mode]: MF: C_46_H_45_N_4_O_8_Cl; found *m*/*z* 839.2839 [M + Na]^+^ [calcd. 839.2824].

### Spectral data of compound 4Cd (±)

Obtained as white solid; yield: 23% (311 mg); mp: 172–174 °C; *R*_f_ 0.35 (6% MeOH in CHCl_3_); ^1^H NMR (*Py-d*_5_): *δ* 12.20 (1H, s, –NH), 11.24 (1H, s, –NH), 7.91 (1H, s), 7.56 (1H, m), 7.39 (1H, m), 7.31 (1H, s), 7.17 (1H, m), 7.07 (3H, m), 7.00 (1H, d, *J* = 7.2 Hz), 6.92 (1H, m), 6.86 (1H, m), 6.67 (1H, d, *J* = 7.8 Hz), 6.21 (1H, s), 4.87 (1H, m), 4.67 (1H, d, *J* = 12.6 Hz), 4.30 (2H, m), 4.03 (1H, m), 3.71 (1H, m), 3.68 (3H, s), 3.51 (3H, s), 2.87 (3H, m), 2.34 (3H, s), 2.23 (1H, s), 1.92 (5H, m), 1.78 (3H, m). ^13^C NMR (*Py-d*_5_): *δ* 198.4 (C=O), 185.7 (C=O), 181.0 (C=O), 180.1 (C=O), 149.2 (C), 148.2 (C), 147.7 (C), 147.4 (C), 142.9 (C), 142.4 (C), 131.3 (C), 130.9 (C), 130.1 (CH), 129.9 (CH), 129.1 (C), 128.4 (C), 128.1 (CH), 128.0 (CH), 127.6 (C), 126.8 (C), 121.4 (CH), 121.3 (CH), 117.1 (CH), 116.4 (CH), 113.2 (CH), 112.4 (CH), 111.9 (CH), 110.1 (CH), 103.0 (CH), 76.5 (C), 75.3 (C), 73.7 (CH), 65.5 (CH), 64.7 (CH), 56.0 (OMe), 55.7 (OMe), 54.0 (CH), 52.4 (CH), 51.5 (CH_2_), 50.9 (CH), 48.7 (CH_2_), 31.0 (CH_2_), 29.9 (CH_2_), 28.1 (CH_2_), 23.2 (CH_2_), 21.5 (CH_3_). MS [ESI-MS, positive mode]: found *m*/*z* 817 [M + H]^+^, 839 [M + Na]^+^. HRMS [ESI-MS, positive mode]: MF: C_46_H_45_N_4_O_8_Cl; found *m*/*z* 839.2812 [M + Na]^+^ [calcd. 839.2824].

### Spectral data of compound 4Da (±)

Obtained as white solid; yield: 18% (246 mg); mp: 238–240 °C; *R*_f_ 0.47 (6% MeOH in CHCl_3_); IR (KBr, *ν*_max_ cm^−1^): 3399, 2959, 2868, 1719, 1605; ^1^H NMR (*Py-d*_5_): *δ* 11.19 (1H, s, –NH), 10.89 (1H, s, –NH), 7.30 (1H, dd, *J* = 2.4 Hz), 7.28 (1H, d, *J* = 8.4 Hz), 7.25 (1H, d, *J* = 7.8 Hz), 7.22 (1H, d, *J* = 1.8 Hz), 7.20 (1H, d, *J* = 1.2 Hz), 7.14 (1H, d, *J* = 1.8 Hz), 7.08 (1H, dd, *J* = 1.8, 8.4 Hz), 7.00 (1H, td, *J* = 2.4, 8.4 Hz), 6.89 (3H, m), 6.85 (1H, q, *J* = 4.2), 5.75 (1H, s), 4.49 (2H, m), 4.20 (2H, m), 3.75 (3H, s), 3.72 (1H, m), 3.70 (3H, s), 3.69 (3H, s), 3.68 (1H, m), 2.82 (1H, m), 2.73 (1H, m), 2.63 (2H, m), 1.88 (2H, m), 1.78 (4H, m), 1.69 (2H, m). ^13^C NMR (*Py-d*_5_): *δ* 191.15 (C=O), 191.11 (C=O), 180.9 (C=O), 180.8 (C=O), 158.7 (C, ^1^*J*_C–F_ = 237.0 Hz), 155.4 (C), 149.3 (C), 149.2 (C), 147.6 (C), 147.5 (C), 139.8 (C), 137.1 (C), 131.5 (C), 131.3 (C), 128.6 (C), 127.9 (C), 121.2 (CH), 121.0 (CH), 117.2 (CH), 117.1 (CH), 116.1 (CH, ^2^*J*_C–F_ = 24.0 Hz), 115.3 (CH), 114.8 (CH, ^2^*J*_C–F_ = 24.0 Hz), 114.3 (CH), 111.9 (CH), 111.5 (CH), 111.1 (CH, ^3^*J*_C–F_ = 9.0 Hz), 110.9 (CH), 101.6 (CH), 74.9 (C), 74.8 (C), 73.8 (CH), 73.5 (CH), 64.6 (CH), 64.5 (CH), 56.2 (OMe), 56.1 (OMe), 56.0 (OMe), 52.6 (CH), 52.4 (CH), 48.4 (2 × CH_2_), 31.1 (CH_2_), 30.9 (CH_2_), 28.0 (CH_2_), 27.9 (CH_2_). MS [ESI-MS, positive mode]: found *m*/*z* 817 [M + H]^+^, 839 [M + Na]^+^. HRMS [ESI-MS, positive mode]: MF: C_46_H_45_N_4_O_9_F; found *m*/*z* 839.3084 [M + Na]^+^ [calcd. 839.3068].

### Spectral data of compound 4Db (±)

Obtained as white solid; yield: 22% (300 mg); mp: 178–180 °C; *R*_f_ 0.43 (6% MeOH in CHCl_3_); IR (KBr, *ν*_max_ cm^−1^): 3385, 2961, 2870, 1721, 1605; ^1^H NMR (*Py-d*_5_): *δ* 11.91 (1H, s, –NH), 11.74 (1H, s, –NH), 7.33 (1H, dd, *J* = 2.4, 8.4 Hz), 7.18 (2H, m), 7.14 (1H, d, *J* = 7.8 Hz), 7.11 (2H, dd, *J* = 1.8, 7.8 Hz), 7.01 (1H, td, *J* = 2.4, 9.0 Hz), 6.96 (1H, d, *J* = 8.4 Hz), 6.87 (3H, m), 6.83 (1H, dd, *J* = 2.4, 8.4 Hz), 5.96 (1H, s), 4.44 (2H, m), 4.13 (2H, m), 3.84 (1H, m), 3.76 (1H, m), 3.73 (3H, s), 3.71 (3H, s), 3.63 (3H, s), 2.76 (1H, m), 2.65 (3H, m), 1.86 (2H, m), 1.78 (2H, m), 1.69 (4H, m). ^13^C NMR (*Py-d*_5_): *δ* 191.4 (C=O), 189.5 (C=O), 181.14 (C=O), 181.08 (C=O), 158.7 (C, ^1^*J*_C–F_ = 237 Hz), 155.4 (C), 149.1 (2 × C), 147.42 (C), 147.40 (C), 139.9 (C), 137.1 (C), 131.5 (C), 131.2 (C), 128.4 (C, ^3^*J*_C–F_ = 7.5 Hz), 128.0 (C), 120.7 (CH), 120.6 (CH), 117.1 (CH), 117.0 (CH), 116.2 (CH, ^2^*J*_C–F_ = 24.0 Hz), 115.4 (CH), 114.9 (CH, ^2^*J*_C–F_ = 25.5 Hz), 114.0 (CH), 112.2 (CH), 112.1 (CH), 111.2 (CH, ^3^*J*_C–F_ = 9 Hz), 110.9 (CH), 101.5 (CH), 75.4 (C), 75.3 (C), 73.9 (CH), 73.6 (CH), 65.0 (CH), 64.5 (CH), 56.08 (OMe), 56.06 (OMe), 55.9 (OMe), 51.9 (CH), 51.8 (CH), 48.2 (2 × CH_2_), 31.6 (CH_2_), 31.5 (CH_2_), 28.41 (CH_2_), 28.40 (CH_2_). MS [ESI-MS, positive mode]: found *m*/*z* 817 [M + H]^+^, 839 [M + Na]^+^. HRMS [ESI-MS, positive mode]: MF: C_46_H_45_N_4_O_9_F; found *m*/*z* 839.3062 [M + Na]^+^ [calcd. 839.3068].

### Spectral data of compound 4Dc (±)

Obtained as white solid; yield: 21% (286 mg); mp: 196–198 °C; *R*_f_ 0.37 (6% MeOH in CHCl_3_); ^1^H NMR (*Py-d*_5_): *δ* 11.81 (1H, s, –NH), 11.39 (1H, s, –NH), 7.66 (1H, dd, *J* = 2.4, 8.4 Hz), 7.40 (1H, d, *J* = 1.8 Hz), 7.34 (1H, s), 7.15 (1H, d, *J* = 7.8 Hz), 7.09 (1H, d, *J* = 9.0 Hz), 7.03 (1H, m), 7.00 (1H, dd, *J* = 2.4, 8.4 Hz), 6.92 (3H, m), 6.86 (1H, d, *J* = 7.8 Hz), 6.64 (1H, q, *J* = 4.2 Hz), 6.11 (1H, s), 4.97 (1H, m), 4.77 (1H, m), 4.66 (1H, d, *J* = 12.6 Hz), 4.31 (1H, m), 4.19 (1H, d, *J* = 12.0 Hz), 3.97 (1H, m), 3.71 (3H, s), 3.68 (3H, s), 3.53 (3H, s), 3.25 (1H, m), 2.90 (1H, m), 2.83 (1H, m), 2.74 (1H, m), 1.93 (1H, m), 1.86 (3H, m), 1.72 (4H, m). ^13^C NMR (*Py-d*_5_): *δ* 195.7 (C=O), 189.0 (C=O), 181.2 (C=O), 180.1 (C=O), 158.9 (C, ^1^*J*_C–F_ = 235.5 Hz), 155.6 (C), 149.2 (C), 148.3 (C), 147.64 (C), 147.56 (C), 140.9 (C), 137.5 (C), 131.5 (C), 129.9 (C, ^3^*J*_C–F_ = 9.4 Hz), 128.4 (C), 126.9 (C), 121.8 (CH), 121.2 (CH), 117.1 (CH), 116.5 (CH), 116.1 (CH, ^2^*J*_C–F_ = 22.5 Hz), 115.7 (CH), 115.2 (CH, ^2^*J*_C–F_ = 24.0 Hz), 113.8 (CH), 112.7 (CH), 112.4 (CH), 110.9 (CH, ^3^*J*_C–F_ = 7.5 Hz), 110.8 (CH), 102.7 (CH), 76.8 (C), 75.4 (C), 73.9 (CH), 65.8 (CH), 64.7 (CH), 56.1 (OMe), 56.0 (OMe), 55.7 (OMe), 53.4 (CH), 52.5 (CH), 52.1 (CH), 51.5 (CH_2_), 48.5 (CH_2_), 31.4 (CH_2_), 28.9 (CH_2_), 28.3 (CH_2_), 26.9 (CH_2_). MS [ESI-MS, positive mode]: found *m*/*z* 817 [M + H]^+^, 839 [M + Na]^+^. HRMS [ESI-MS, positive mode]: MF: C_46_H_45_N_4_O_9_F; found *m*/*z* 839.3059 [M + Na]^+^ [calcd. 839.3068].

### Spectral data of compound 4Dd (±)

Obtained as white solid; yield: 24% (327 mg); mp: 189–191 °C; *R*_f_ 0.36 (6% MeOH in CHCl_3_); ^1^H NMR (*Py-d*_5_): *δ* 11.66 (1H, s, –NH), 11.37 (1H, s, –NH), 7.72 (1H, d, *J* = 8.4 Hz), 7.45 (1H, s), 7.37 (1H, s), 7.18 (1H, d, *J* = 8.4 Hz), 7.10 (1H, d, *J* = 7.8 Hz), 7.02 (2H, m), 6.95 (3H, m), 6.92 (1H, m), 6.66 (1H, q, *J* = 3.6 Hz), 5.97 (1H, s), 4.93 (1H, t, *J* = 10.2 Hz), 4.71 (1H, m), 4.60 (1H, d, *J* = 11.4 Hz), 4.41 (1H, m), 4.25 (1H, d, *J* = 12.0 Hz), 4.01 (1H, m), 3.80 (3H, s), 3.69 (3H, s), 3.52 (3H, s), 3.20 (1H, m), 2.97 (1H, m), 2.78 (2H, m), 1.91 (4H, m), 1.77 (2H, m), 1.65 (1H, m), 1.60 (1H, m). ^13^C NMR (*Py-d*_5_): *δ* 194.0 (C=O), 187.2 (C=O), 181.3 (C=O), 180.1 (C=O), 158.9 (C, ^1^*J*_C–F_ = 235.5 Hz), 155.6 (C), 149.3 (C), 148.3 (C), 147.6 (C), 147.5 (C), 140.9 (C), 137.5 (C), 131.7 (C), 130.0 (C), 128.5 (C), 127.4 (C), 121.8 (CH), 121.6 (CH), 117.0 (CH), 116.4 (CH), 116.0 (CH, ^2^*J*_C–F_ = 24.0 Hz), 115.8 (CH), 115.3 (CH, ^2^*J*_C–F_ = 24.0 Hz), 114.3 (CH), 113.1 (CH), 112.3 (CH), 110.9 (CH), 110.8 (CH), 102.8 (CH), 76.8 (C), 75.7 (C), 73.6 (CH), 65.8 (CH), 65.0 (CH), 56.3 (OMe), 56.1 (OMe), 55.8 (OMe), 53.9 (CH), 52.7 (CH), 51.45 (CH), 51.41 (CH_2_), 48.6 (CH_2_), 31.2 (CH_2_), 29.3 (CH_2_), 28.1 (CH_2_), 26.8 (CH_2_). MS [ESI-MS, positive mode]: found *m*/*z* 817 [M + H]^+^, 839 [M + Na]^+^. HRMS [ESI-MS, positive mode]: MF: C_46_H_45_N_4_O_9_F; found *m*/*z* 839.3059 [M + Na]^+^ [calcd. 839.3068].

### Spectral data of compound 4Ea (±)

Obtained as white solid; yield: 18% (250 mg); mp: 180–182 °C; *R*_f_ 0.47 (6% MeOH in CHCl_3_); IR (KBr, *ν*_max_ cm^−1^): 3397, 2959, 2868, 1720, 1615; ^1^H NMR (*Py-d*_5_): *δ* 11.02 (1H, s, –NH), 10.96 (1H, s, –NH), 7.32 (1H, dd, *J* = 2.4, 8.4 Hz), 7.28 (2H, m), 7.23 (1H, d, *J* = 1.8 Hz), 7.21 (1H, m), 7.17 (1H, d, *J* = 1.2 Hz), 7.09 (1H, dd, *J* = 1.2, 7.8 Hz), 6.97 (2H, m), 6.81 (1H, m), 6.77 (1H, s), 5.74 (1H, s), 4.52 (1H, d, *J* = 12.6 Hz), 4.38 (1H, d, *J* = 12.0 Hz), 4.33 (1H, m), 4.17 (1H, m), 3.79 (2H, m), 3.75 (3H, s), 3.70 (3H, s), 2.82 (1H, m), 2.76 (1H, m), 2.63 (2H, m), 2.24 (3H, s), 2.20 (3H, s), 1.82 (6H, m), 1.69 (2H, m). ^13^C NMR (*Py-d*_5_): *δ* 192.4 (C=O), 190.2 (C=O), 181.2 (C=O), 181.0 (C=O), 158.7 (C, ^1^*J*_C–F_ = 235.5 Hz), 149.22 (C), 149.17 (C), 147.5 (C), 147.4 (C), 139.8 (C), 139.6 (C), 131.9 (CH), 131.7 (C), 131.5 (C), 130.6 (C), 128.3 (C), 126.1 (C), 125.4 (CH), 120.91 (CH), 120.87 (CH), 119.3 (C), 117.2 (CH), 117.1 (CH), 116.1 (CH, ^2^*J*_C–F_ = 22.5 Hz), 115.1 (CH, ^2^*J*_C–F_ = 24.0 Hz), 112.4 (CH), 111.6 (CH), 111.1 (CH), 101.6 (CH), 74.9 (C), 74.8 (C), 73.8 (CH), 73.3 (CH), 65.6 (CH), 63.6 (CH), 56.1 (OMe), 56.0 (OMe), 52.4 (CH), 52.3 (CH), 48.5 (CH_2_), 48.4 (CH_2_), 31.1 (CH_2_), 30.7 (CH_2_), 28.0 (CH_2_), 27.7 (CH_2_), 21.2 (CH_3_), 17.1 (CH_3_). MS [ESI-MS, positive mode]: found *m*/*z* 815 [M + H]^+^, 837 [M + Na]^+^. HRMS [ESI-MS, positive mode]: MF: C_47_H_47_N_4_O_8_F; found *m*/*z* 837.3240 [M + Na]^+^ [calcd. 837.3276].

### Spectral data of compound 4Eb (±)

Obtained as white solid; yield: 22% (305 mg); mp: 182–184 °C; *R*_f_ 0.43 (6% MeOH in CHCl_3_); IR (KBr, *ν*_max_ cm^−1^): 3403, 3208, 2964, 2871, 1713, 1605; ^1^H NMR (*Py-d*_5_): *δ* 12.03 (1H, s, –NH), 11.73 (1H, s, –NH), 7.28 (1H, d, *J* = 7.8 Hz), 7.17 (2H, m), 7.13 (2H, m), 7.08 (1H, s), 6.98 (1H, m), 6.82 (3H, m), 6.69 (1H, s), 5.93 (1H, s), 4.38 (2H, m), 4.20 (1H, m), 4.13 (1H, m), 3.92 (1H, m), 3.78 (1H, m), 3.67 (3H, s), 3.64 (3H, s), 2.84 (1H, m), 2.68 (3H, m), 2.28 (3H, s), 2.12 (3H, s), 1.92 (1H, m), 1.84 (4H, m), 1.72 (3H, m). ^13^C NMR (*Py-d*_5_): *δ* 190.7 (C=O), 190.3 (C=O), 181.5 (C=O), 181.3 (C=O), 158.6 (C, ^1^*J*_C–F_ = 237.0 Hz), 149.0 (2 × C), 147.44 (C), 147.38 (C), 139.9 (2 × C), 131.9 (CH), 131.6 (C), 131.3 (C), 130.6 (C), 128.3 (C), 126.2 (C), 125.5 (CH), 120.3 (CH), 120.2 (CH), 119.4 (C), 117.0 (CH), 116.9 (CH), 116.2 (CH, ^2^*J*_C–F_ = 24.0 Hz), 115.1 (CH, ^2^*J*_C–F_ = 24.0 Hz), 112.6 (CH), 112.3 (CH), 111.0 (CH, ^3^*J*_C–F_ = 7.5 Hz), 101.4 (CH), 75.4 (2 × C), 73.6 (CH), 73.4 (CH), 65.2 (CH), 64.9 (CH), 56.04 (OMe), 55.98 (OMe), 51.8 (CH), 51.5 (CH), 48.33 (CH_2_), 48.29 (CH_2_), 31.5 (CH_2_), 31.3 (CH_2_), 28.4 (CH_2_), 28.2 (CH_2_), 21.3 (CH_3_), 17.2 (CH_3_). MS [ESI-MS, positive mode]: found *m*/*z* 815 [M + H]^+^, 837 [M + Na]^+^. HRMS [ESI-MS, positive mode]: MF: C_47_H_47_N_4_O_8_F; found *m*/*z* 837.3305 [M + Na]^+^ [calcd. 837.3276].

### Spectral data of compound 4Ec (±)

Obtained as white solid; yield: 21% (292 mg); mp: 172–174 °C; *R*_f_ 0.38 (6% MeOH in CHCl_3_); ^1^H NMR (*Py-d*_5_): *δ* 12.01 (1H, s, –NH), 11.15 (1H, s, –NH), 7.66 (1H, dd, *J* = 2.4, 8.4 Hz), 7.37 (1H, s), 7.31 (1H, d, *J* = 1.8 Hz), 7.17 (1H, m), 7.13 (2H, m), 7.01 (1H, m), 6.92 (2H, m), 6.85 (1H, m), 6.79 (1H, s), 6.15 (1H, s), 4.99 (1H, dd, *J* = 9.6, 12.0 Hz), 4.80 (1H, q, *J* = 8.4 Hz), 4.66 (1H, d, *J* = 12.0 Hz), 4.31 (1H, m), 4.23 (1H, d, *J* = 12.0 Hz), 4.00 (1H, dd, *J* = 9.6, 12.0 Hz), 3.70 (3H, s), 3.52 (3H, s), 3.21 (1H, m), 2.83 (2H, m), 2.74 (1H, m), 2.33 (3H, s), 1.99 (3H, s), 1.91 (3H, m), 1.83 (2H, m), 1.76 (2H, m), 1.69 (1H, m). ^13^C NMR (*Py-d*_5_): *δ* 196.8 (C=O), 188.2 (C=O), 181.3 (C=O), 180.4 (C=O), 158.9 (C, ^1^*J*_C–F_ = 237.0 Hz), 149.3 (C), 148.2 (C), 147.7 (C), 147.4 (C), 141.0 (C), 140.2 (C), 131.8 (CH), 131.2 (C), 130.8 (C), 128.8 (C, ^3^*J*_C–F_ = 7.5 Hz), 127.8 (C), 127.5 (C), 125.3 (CH), 121.7 (CH), 121.2 (CH), 119.3 (C), 117.1 (CH), 116.4 (CH), 116.3 (CH, ^2^*J*_C–F_ = 24.0 Hz), 115.4 (CH, ^2^*J*_C–F_ = 24.0 Hz), 113.0 (CH), 112.3 (CH), 111.2 (CH, ^3^*J*_C–F_ = 7.5 Hz), 102.8 (CH), 76.6 (C), 75.4 (C), 73.9 (CH), 65.6 (CH), 64.7 (CH), 56.1 (OMe), 55.6 (OMe), 54.0 (CH), 52.3 (CH), 51.7 (CH), 51.5 (CH_2_), 48.4 (CH_2_), 31.3 (CH_2_), 29.0 (CH_2_), 28.3 (CH_2_), 26.9 (CH_2_), 21.4 (CH_3_), 17.0 (CH_3_). MS [ESI-MS, positive mode]: found *m*/*z* 815 [M + H]^+^, 837 [M + Na]^+^. HRMS [ESI-MS, positive mode]: MF: C_47_H_47_N_4_O_8_F; found *m*/*z* 837.3284 [M + Na]^+^ [calcd. 837.3276].

### Spectral data of compound 4Ed (±)

Obtained as white solid; yield: 23% (319 mg); mp: 170–172 °C; *R*_f_ 0.37 (6% MeOH in CHCl_3_); ^1^H NMR (*Py-d*_5_): *δ* 11.87 (1H, s, –NH), 11.14 (1H, s, –NH), 7.74 (1H, dd, *J* = 2.4, 8.4 Hz), 7.40 (1H, s), 7.31 (1H, d, *J* = 1.8 Hz), 7.27 (1H, m), 7.18 (1H, m), 7.12 (2H, m), 6.95 (2H, m), 6.91 (1H, d, *J* = 8.4 Hz), 6.80 (1H, s), 6.04 (1H, s), 5.05 (1H, dd, *J* = 9.6, 12.0 Hz), 4.68 (1H, m), 4.61 (1H, d, *J* = 12.0 Hz), 4.37 (1H, m), 4.25 (1H, d, *J* = 12.0 Hz), 4.00 (1H, m), 3.66 (3H, s), 3.54 (3H, s), 3.29 (1H, m), 2.77 (2H, m), 2.38 (3H, s), 1.99 (3H, s), 1.93 (2H, m), 1.76 (3H, m), 1.64 (2H, m), 1.55 (2H, m). ^13^C NMR (*Py-d*_5_): *δ* 198.0 (C=O), 186.2 (C=O), 181.2 (C=O), 180.4 (C=O), 158.9 (C, ^1^*J*_C–F_ = 237.0 Hz), 149.3 (C), 148.3 (C), 147.7 (C), 147.4 (C), 141.0 (C), 140.1 (C), 131.8 (CH), 131.2 (C), 130.8 (C), 128.8 (C, ^3^*J*_C–F_ = 7.5 Hz), 127.8 (C), 127.3 (C), 125.4 (CH), 121.7 (CH), 121.3 (CH), 119.3 (C), 117.14 (CH), 117.08 (CH), 116.3 (CH, ^2^*J*_C–F_ = 24.0 Hz), 115.6 (CH, ^2^*J*_C–F_ = 24.0 Hz), 113.1 (CH), 112.3 (CH), 111.3 (CH, ^3^*J*_C–F_ = 7.5 Hz), 102.9 (CH), 76.5 (C), 75.7 (C), 73.7 (CH), 65.5 (CH), 64.7 (CH), 56.1 (OMe), 55.7 (OMe), 54.2 (CH), 52.4 (CH), 51.5 (CH_2_), 50.8 (CH), 48.5 (CH_2_), 31.2 (CH_2_), 29.9 (CH_2_), 28.3 (CH_2_), 26.8 (CH_2_), 21.4 (CH_3_), 17.0 (CH_3_). MS [ESI-MS, positive mode]: found *m*/*z* 815 [M + H]^+^, 837 [M + Na]^+^. HRMS [ESI-MS, positive mode]: MF: C_47_H_47_N_4_O_8_F; found *m*/*z* 837.3264 [M + Na]^+^ [calcd. 837.3276].

### Spectral data of compound 4Fa (±)

Obtained as white solid; yield: 17% (232 mg); mp: 182–184 °C; *R*_f_ 0.47 (6% MeOH in CHCl_3_); IR (KBr, *ν*_max_ cm^−1^): 3385, 2959, 2868, 1719, 1607; ^1^H NMR (*Py-d*_5_): *δ* 10.96 (2H, s, –NH), 7.32 (1H, dd, *J* = 2.4, 8.4 Hz), 7.27 (2H, m), 7.23 (1H, d, *J* = 1.8 Hz), 7.22 (1H, m), 7.16 (1H, d, *J* = 1.8 Hz), 7.09 (1H, dd, *J* = 1.8, 7.8 Hz), 6.96 (2H, m), 6.80 (1H, m), 6.77 (1H, s), 5.74 (1H, s), 4.52 (1H, d, *J* = 12.6 Hz), 4.38 (1H, d, *J* = 12.0 Hz), 4.33 (1H, m), 4.16 (1H, m), 3.79 (2H, m), 3.75 (3H, s), 3.70 (3H, s), 2.80 (1H, m), 2.76 (1H, m), 2.62 (2H, m), 2.24 (3H, s), 2.20 (3H, s), 1.82 (6H, m), 1.69 (2H, m). ^13^C NMR (*Py-d*_5_): *δ* 192.3 (C=O), 190.2 (–C=O), 181.2 (C=O), 181.0 (C=O), 158.7 (C, ^1^*J*_C–F_ = 240.0 Hz), 149.21 (C), 149.16 (C), 147.5 (C), 147.4 (C), 139.8 (C), 139.6 (C), 131.9 (CH), 131.6 (C), 131.5 (C), 130.6 (C), 128.3 (C, ^3^*J*_C–F_ = 7.5 Hz), 126.1 (C), 125.4 (CH), 120.91 (CH), 120.87 (CH), 119.3 (C), 117.2 (CH), 117.1 (CH), 116.1 (CH, ^2^*J*_C–F_ = 22.5 Hz), 115.1 (CH, ^2^*J*_C–F_ = 24.0 Hz), 112.4 (CH), 111.6 (CH), 111.1 (CH), 101.7 (-CH), 74.9 (C), 74.8 (C), 73.8 (CH), 73.3 (CH), 65.6 (CH), 63.8 (CH), 56.1 (OMe), 56.0 (OMe), 52.4 (CH), 52.3 (CH), 48.5 (CH_2_), 48.4 (CH_2_), 31.1 (CH_2_), 30.7 (CH_2_), 28.0 (CH_2_), 27.7 (CH_2_), 21.2 (CH_3_), 17.1 (CH_3_). MS [ESI-MS, positive mode]: found *m*/*z* 815 [M + H]^+^, 837 [M + Na]^+^. HRMS [ESI-MS, positive mode]: MF: C_47_H_47_N_4_O_8_F; found *m*/*z* 815.3458 [M + H]^+^ [calcd. 815.3456].

### Spectral data of compound 4Fb (±)

Obtained as white solid; yield: 20% (273 mg); mp: 184–186 °C; *R*_f_ 0.43 (6% MeOH in CHCl_3_); IR (KBr, *ν*_max_ cm^−1^): 3402, 2958, 2869, 1714, 1613, 1518; ^1^H NMR (*Py-d*_5_): *δ* 12.03 (1H, s, –NH), 11.73 (1H, s, –NH), 7.28 (1H, dd, *J* = 2.4, 7.8 Hz), 7.19 (1H, s), 7.16 (1H, d, *J* = 8.4 Hz), 7.13 (2H, m), 7.08 (1H, d, *J* = 1.8 Hz), 6.98 (1H, td, *J* = 2.4, 9.0 Hz), 6.82 (3H, m), 6.69 (1H, s), 5.92 (1H, s), 4.38 (2H, m), 4.20 (1H, m), 4.13 (1H, m), 3.92 (1H, m), 3.78 (1H, m), 3.67 (3H, s), 3.64 (3H, s), 2.85 (1H, m), 2.67 (3H, m), 2.28 (3H, s), 2.12 (3H, s), 1.92 (1H, m), 1.85 (2H, m), 1.81 (2H, m), 1.71 (3H, m). ^13^C NMR (*Py-d*_5_): *δ* 190.6 (C=O), 190.3 (C=O), 181.5 (C=O), 181.3 (C=O), 158.6 (C, ^1^*J*_C–F_ = 237.0 Hz), 149.0 (2 × C), 147.44 (C), 147.38 (C), 139.9 (2 × C), 131.9 (CH), 131.6 (C), 131.3 (C), 130.6 (C), 128.4 (C, ^3^*J*_C–F_ = 7.5 Hz), 126.2 (C), 125.5 (CH), 120.3 (CH), 120.2 (CH), 119.4 (C), 117.0 (CH), 116.9 (CH), 116.2 (CH, ^2^*J*_C–F_ = 24.0 Hz), 115.1 (CH, ^2^*J*_C–F_ = 24.0 Hz), 112.6 (CH), 112.3 (CH), 111.0 (CH, ^3^*J*_C–F_ = 7.5 Hz), 101.4 (CH), 75.44 (C), 75.41 (C), 73.6 (CH), 73.4 (CH), 65.3 (CH), 64.9 (CH), 56.03 (OMe), 55.98 (OMe), 51.8 (CH), 51.5 (CH), 48.33 (CH_2_), 48.29 (CH_2_), 31.5 (CH_2_), 31.3 (CH_2_), 28.4 (CH_2_), 28.2 (CH_2_), 21.3 (CH_3_), 17.2 (CH_3_). MS [ESI-MS, positive mode]: found *m*/*z* 815 [M + H]^+^, 837 [M + Na]^+^. HRMS [ESI-MS, positive mode]: MF: C_47_H_47_N_4_O_8_F; found *m*/*z* 815.3456 [M + H]^+^ [calcd. 815.3456].

### Spectral data of compound 4Fc (±)

Obtained as white solid; yield: 20% (273 mg); mp: 173–175 °C; *R*_f_ 0.37 (6% MeOH in CHCl_3_); ^1^H NMR (*Py-d*_5_): *δ* 11.77 (1H, s, –NH), 11.38 (1H, s, –NH), 7.63 (1H, m), 7.44 (1H, s), 7.34 (1H, s), 7.16 (1H, d, *J* = 8.4 Hz), 7.12 (1H, m), 7.00 (1H, td, *J* = 2.4, 9.0 Hz), 6.91 (2H, m), 6.86 (2H, m), 6.64 (1H, q, *J* = 4.2 Hz), 6.10 (1H, s), 4.99 (1H, m), 4.74 (1H, m), 4.65 (1H, d, *J* = 12.0 Hz), 4.33 (1H, m), 4.14 (1H, d, *J* = 12.0 Hz), 4.05 (1H, m), 3.67 (3H, s), 3.51 (3H, s), 3.22 (1H, m), 2.95 (1H, m), 2.83 (1H, m), 2.75 (1H, m), 2.39 (3H, s), 2.28 (3H, s), 1.95 (3H, m), 1.78 (3H, m), 1.69 (2H, m). ^13^C NMR (*Py-d*_5_): *δ* 195.7 (C=O), 189.0 (C=O), 181.6 (C=O), 180.1 (C=O), 158.9 (C, ^1^*J*_C–F_ = 235.5 Hz), 149.2 (C), 148.3 (C), 147.61 (C), 147.58 (C), 140.9 (C), 140.3 (C), 131.9 (CH), 131.6 (C), 130.8 (C), 129.8 (C, ^3^*J*_C–F_ = 7.4 Hz), 126.8 (C), 126.7 (C), 125.7 (CH), 121.7 (CH), 121.2 (CH), 119.5 (C), 117.1 (CH), 116.4 (CH), 116.1 (CH, ^2^*J*_C–F_ = 22.5 Hz), 115.2 (CH, ^2^*J*_C–F_ = 24.0 Hz), 112.7 (CH), 112.4 (CH), 110.9 (CH, ^3^*J*_C–F_ = 7.5 Hz), 102.8 (CH), 76.8 (C), 75.2 (C), 73.8 (CH), 65.9 (CH), 64.6 (CH), 56.1 (OMe), 55.7 (OMe), 53.2 (CH), 52.6 (CH), 52.0 (CH), 51.5 (CH_2_), 48.6 (CH_2_), 31.3 (CH_2_), 29.0 (CH_2_), 28.2 (CH_2_), 26.9 (CH_2_), 21.3 (CH_3_), 17.4 (CH_3_). MS [ESI-MS, positive mode]: found *m*/*z* 815 [M + H]^+^, 837 [M + Na]^+^. HRMS [ESI-MS, positive mode]: MF: C_47_H_47_N_4_O_8_F; found *m*/*z* 815.3462 [M + H]^+^ [calcd. 815.3456].

### Spectral data of compound 4Fd (±)

Obtained as white solid; yield: 22% (300 mg); mp: 186–188 °C; *R*_f_ 0.36 (6% MeOH in CHCl_3_); ^1^H NMR (*Py-d*_5_): *δ* 11.73 (1H, s, –NH), 11.37 (1H, s, –NH), 7.66 (1H, d, *J* = 7.8 Hz), 7.54 (1H, s), 7.41 (1H, s), 7.18 (2H, s), 7.01 (1H, td, *J* = 3.0, 9.0 Hz), 6.95 (1H, dd, *J* = 1.8, 8.4 Hz), 6.90 (3H, m), 6.68 (1H, q, *J* = 4.2 Hz), 6.00 (1H, s), 4.91 (1H, m), 4.71 (1H, m), 4.64 (1H, d, *J* = 12.0 Hz), 4.40 (1H, m), 4.29 (1H, d, *J* = 12.0 Hz), 4.12 (1H, m), 3.70 (3H, s), 3.49 (3H, s), 3.18 (1H, m), 2.97 (1H, m), 2.76 (2H, m), 2.37 (3H, s), 2.34 (3H, s), 2.25 (1H, m), 1.96 (3H, m), 1.79 (1H, m), 1.73 (1H, m), 1.63 (1H, m), 1.58 (1H, m). ^13^C NMR (*Py-d*_5_): *δ* 195.2 (C=O), 190.8 (C=O), 185.8 (C=O), 180.3 (C=O), 158.9 (C, ^1^*J*_C–F_ = 235.5 Hz), 149.2 (C), 148.2 (C), 147.45 (C), 147.37 (C), 140.9 (C), 140.2 (C), 131.9 (CH), 130.7 (C), 130.2 (C), 129.6 (C), 127.75 (C), 127.0 (C), 126.1 (CH), 121.6 (CH), 121.4 (CH), 119.5 (C), 117.0 (CH), 116.3 (CH), 116.0 (CH, ^2^*J*_C–F_ = 24.0 Hz), 115.9 (CH, ^2^*J*_C–F_ = 25.5 Hz), 113.3 (CH), 112.5 (CH), 110.8 (CH, ^3^*J*_C–F_ = 7.5 Hz), 102.85 (CH), 76.8 (C), 75.7 (C), 73.7 (CH), 65.1 (CH), 64.7 (CH), 56.1 (OMe), 55.7 (OMe), 54.4 (CH), 52.6 (CH), 51.3 (CH, CH_2_), 48.7 (CH_2_), 31.3 (CH_2_), 29.2 (CH_2_), 28.2 (CH_2_), 26.8 (CH_2_), 21.4 (CH_3_), 17.4 (CH_3_). MS [ESI-MS, positive mode]: found *m*/*z* 815 [M + H]^+^, 837 [M + Na]^+^. HRMS [ESI-MS, positive mode]: MF: C_47_H_47_N_4_O_8_F; found *m*/*z* 815.3452 [M + H]^+^ [calcd. 815.3456].

### Spectral data of compound 5Aa (±)

Obtained as white solid; yield: 30% (655 mg); mp: 230–232 °C; *R*_f_ 0.65 (5% MeOH in CHCl_3_); UV absorption maximas (*λ*_max_ nm, methanol) 297, 229; IR (KBr, *ν*_max_ cm^−1^): 3336, 2927, 1725, 1615, 1517; ^1^H NMR (*Py-d*_5_): *δ* 11.26 (2H, s, –NH), 7.64 (2H, d, *J* = 7.8 Hz), 7.31 (2H, m), 7.25 (2H, dd, *J* = 0.6, 7.8 Hz), 7.22 (2H, m), 6.99 (4H, m), 6.93 (2H, d, *J* = 7.8 Hz), 5.74 (1H, s), 4.36 (2H, d, *J* = 12.6 Hz), 4.33 (2H, m), 3.92 (2H, d, *J* = 10.2 Hz), 3.76 (6H, s), 3.65 (4H, m), 3.11 (2H, dd, *J* = 2.4, 11.4 Hz), 3.00 (2H, m). ^13^C NMR (*Py-d*_5_): *δ* 190.5 (C=O), 180.0 (C=O), 149.5 (C), 148.0 (C), 143.8 (C), 130.5 (C), 130.3 (CH), 128.5 (CH), 124.5 (C), 121.7 (CH), 121.5 (CH), 117.3 (CH), 111.7 (CH), 110.5 (CH), 101.4 (CH), 76.7 (CH), 75.1 (C), 62.6 (CH), 56.2 (OMe), 54.9 (CH_2_), 51.5 (CH), 36.7 (CH_2_). MS [ESI-MS, positive mode]: found *m*/*z* 827 [M + Na]^+^. HRMS [ESI-MS, positive mode]: MF: C_43_H_40_N_4_O_8_S_2_; found *m*/*z* 827.2180 [M + Na]^+^ [calcd. 827.2185].

### Spectral data of compound 5Ab (±)

Obtained as white solid; yield: 35% (765 mg); mp: 242–244 °C; *R*_f_ 0.55 (5% MeOH in CHCl_3_); UV absorption maximas (*λ*_max_ nm, methanol) 304, 228; IR (KBr, *ν*_max_ cm^−1^): 3407, 2928, 1718, 1617, 1518; ^1^H NMR (*Py-d*_5_): *δ* 11.94 (2H, s, –NH), 7.43 (4H, m), 7.30 (2H, d, *J* = 1.8 Hz), 7.20 (1H, d, *J* = 1.8 Hz), 7.18 (3H, t, *J* = 7.8 Hz), 6.94 (4H, m), 5.98 (1H, s), 4.36 (2H, d, *J* = 13.2 Hz), 4.32 (2H, t, *J* = 7.8 Hz), 3.92 (2H, d, *J* = 10.8 Hz), 3.86 (6H, s), 3.63 (4H, m), 3.15 (2H, d, *J* = 11.4 Hz), 3.00 (2H, dd, *J* = 6.6, 11.4 Hz). ^13^C NMR (*Py-d*_5_): *δ* 190.5 (C=O), 180.3 (C=O), 148.2 (2 × C), 144.0 (C), 130.3 (CH), 130.2 (C), 128.4 (CH), 123.5 (C), 121.8 (CH), 121.6 (CH), 117.5 (CH), 111.7 (CH), 110.5 (CH), 101.1 (CH), 76.9 (CH), 75.5 (C), 61.6 (CH), 56.3 (OMe), 55.9 (CH_2_), 51.9 (CH), 36.9 (CH_2_). MS [ESI-MS, positive mode]: found *m*/*z* 827 [M + Na] ^+^. HRMS [ESI-MS, positive mode]: MF: C_43_H_40_N_4_O_8_S_2_; found *m*/*z* 827.2167 [M + Na]^+^ [calcd. 827.2185].

### Spectral data of compound 5Ba (±)

Obtained as white solid; yield: 31% (700 mg); mp: 216–218 °C; *R*_f_ 0.60 (5% MeOH in CHCl_3_); IR (KBr, *ν*_max_ cm^−1^): 3379, 2923, 1718, 1602, 1521; ^1^H NMR (*Py-d*_5_): *δ* 10.96 (2H, s, –NH), 7.52 (2H, s), 7.27 (2H, d, *J* = 8.4 Hz), 7.22 (2H, m), 7.00 (2H, dd, *J* = 1.8, 8.4 Hz), 6.94 (2H, d, *J* = 8.4 Hz), 6.84 (2H, d, *J* = 7.8 Hz), 5.66 (1H, s), 4.36 (2H, m), 4.30 (2H, d, *J* = 12.0 Hz), 3.88 (2H, d, *J* = 10.2 Hz), 3.78 (2H, m), 3.73 (6H, s), 3.60 (2H, d, *J* = 9.6 Hz), 3.10 (2H, dd, *J* = 2.4, 10.8 Hz), 2.98 (2H, m), 2.12 (6H, s). ^13^C NMR (*Py-d*_5_): *δ* 190.2 (C=O), 180.2 (C=O), 149.3 (C), 147.7 (C), 141.1 (C), 130.9 (C), 130.724 (C), 130.716 (CH), 129.1 (CH), 124.6 (C), 121.2 (CH), 117.3 (CH), 112.2 (CH), 110.3 (CH), 101.6 (CH), 76.4 (CH), 75.2 (C), 62.9 (CH), 56.2 (OMe), 54.6 (CH_2_), 50.9 (CH), 36.7 (CH_2_), 21.3 (CH_3_). MS [ESI-MS, positive mode]: found *m*/*z* 855 [M + Na]^+^. HRMS [ESI-MS, positive mode]: MF: C_45_H_44_N_4_O_8_S_2_; found *m*/*z* 855.2495 [M + Na]^+^ [calcd. 855.2498].

### Spectral data of compound 5Bb (±)

Obtained as white solid; yield: 35% (791 mg); mp: 260–262 °C; *R*_f_ 0.53 (5% MeOH in CHCl_3_); IR (KBr, *ν*_max_ cm^−1^): 3388, 2921, 1708, 1617, 1516; ^1^H NMR (*Py-d*_5_): *δ* 11.93 (2H, s, –NH), 7.55 (2H, s), 7.17 (2H, d, *J* = 7.8 Hz), 7.11 (2H, d, *J* = 1.8 Hz), 6.93 (2H, d, *J* = 7.8 Hz), 6.88 (2H, dd, *J* = 1.2, 7.8 Hz), 6.79 (2H, d, *J* = 7.8 Hz), 5.88 (1H, s), 4.30 (2H, m), 4.27 (2H, d, *J* = 12.0 Hz), 3.95 (2H, d, *J* = 10.2 Hz), 3.86 (2H, m), 3.70 (2H, d, *J* = 10.2 Hz), 3.67 (6H, s), 3.15 (2H, dd, *J* = 1.8, 11.4 Hz), 3.02 (2H, m), 2.08 (6H, s). ^13^C NMR (*Py-d*_5_): *δ* 189.9 (C=O), 180.3 (C=O), 149.2 (C), 147.8 (C), 141.4 (C), 130.8 (CH), 130.7 (C), 130.5 (C), 129.2 (CH), 124.6 (C), 120.8 (CH), 117.2 (CH), 112.4 (CH), 110.0 (CH), 101.3 (CH), 76.7 (CH), 75.9 (C), 63.0 (CH), 56.0 (OMe), 55.1 (CH_2_), 50.8 (CH), 37.0 (CH_2_), 21.3 (CH_3_). MS [ESI-MS, positive mode]: found *m*/*z* 855 [M + Na]^+^. HRMS [ESI-MS, positive mode]: MF: C_45_H_44_N_4_O_8_S_2_; found *m*/*z* 855.2498 [M + Na]^+^ [calcd. 855.2498].

### Spectral data of compound 6Aa (±)

Color: Obtained as white solid; Yield: 30% (431 mg); Mp: 172–174 °C; *R*_f_ 0.60 (5% MeOH in CHCl_3_); UV absorption maximas (*λ*_max_ nm, methanol) 258, 207; IR (KBr, *ν*_max_ cm^−1^): 3290, 2926, 1720, 1608, 1520; ^1^H NMR (*Py-d*_5_): *δ* 11.13 (2H, s, –NH), 7.61 (1H, s), 7.32 (2H, d, *J* = 8.4 Hz), 7.26 (1H, d, *J* = 8.4 Hz), 7.18 (2H, m), 7.15 (1H, m), 7.13 (1H, s), 6.94 (4H, m), 6.81 (1H, m), 5.77 (1H, s), 4.49 (1H, d, *J* = 12.0 Hz), 4.36 (1H, d, *J* = 12.6 Hz), 4.32 (1H, m), 4.23 (1H, m), 3.96 (1H, d, *J* = 9.6 Hz), 3.81 (3H, s), 3.73 (3H, s), 3.70 (1H, m), 3.68 (3H, s), 3.63 (2H, m), 3.08 (1H, d, *J* = 10.2 Hz), 2.98 (1H, m), 2.76 (1H, m), 2.61 (1H, m), 1.89 (1H, m), 1.79 (2H, m), 1.67 (1H, m). ^13^C NMR (*Py-d*_5_): *δ* 191.8 (C=O), 189.9 (C=O), 181.0 (C=O), 179.9 (C=O), 155.2 (C), 149.4 (C), 149.3 (C), 147.9 (C), 147.6 (C), 143.8 (C), 137.2 (C), 131.8 (C), 130.2 (C), 129.7 (CH), 126.94 (C), 126.89 (CH), 125.9 (C), 121.8 (CH), 121.6 (CH), 121.1 (CH), 117.3 (CH), 117.2 (CH), 116.4 (CH), 115.0 (CH), 111.8 (CH), 111.5 (CH), 110.8 (CH), 110.6 (CH), 101.6 (CH), 76.5 (CH), 75.7 (C), 74.4 (C), 73.9 (CH), 64.0 (CH), 63.0 (CH), 56.19 (OMe), 56.16 (OMe), 56.12 (OMe), 55.1 (CH_2_), 52.7 (CH), 51.6 (CH), 48.6 (CH_2_), 36.7 (CH_2_), 30.9 (CH_2_), 27.6 (CH_2_). MS [ESI-MS, positive mode]: found *m*/*z* 817 [M + H]^+^, 839 [M + Na]^+^. HRMS [ESI-MS, positive mode]: MF: C_45_H_44_N_4_O_9_S; found *m*/*z* 839.2722 [M + Na]^+^ [calcd. 839.2727].

### Spectral data of compound 6Ab (±)

Obtained as white solid; yield: 35% (503 mg); mp: 176–178 °C; *R*_f_ 0.55 (5% MeOH in CHCl_3_); UV absorption maximas (*λ*_max_ nm, methanol) 256, 201; ^1^H NMR (*Py-d*_5_): *δ* 11.91 (1H, s, –NH), 11.78 (1H, s, –NH), 7.49 (1H, d, *J* = 2.4 Hz), 7.22 (2H, m), 7.19 (1H, m), 7.16 (2H, dd, *J* = 1.8, 7.8 Hz), 7.13 (1H, d, *J* = 7.2 Hz), 7.01 (1H, dd, *J* = 1.8, 7.8 Hz), 6.94 (4H, m), 6.87 (1H, dd, *J* = 2.4, 8.4 Hz), 5.95 (1H, s), 4.38 (2H, m), 4.28 (1H, m), 4.16 (1H, m), 3.97 (1H, d, *J* = 10.8 Hz), 3.79 (1H, m), 3.76 (3H, s), 3.72 (3H, s), 3.68 (1H, m), 3.63 (3H, s, 1H, m), 3.13 (1H, dd, *J* = 1.8, 11.4 Hz), 3.00 (1H, m), 2.69 (1H, m), 2.60 (1H, m), 1.85 (1H, m), 1.68 (3H, m). ^13^C NMR (*Py-d*_5_): *δ* 191.2 (C=O), 189.6 (C=O), 181.1 (C=O), 180.3 (C=O), 155.1 (C), 149.3 (C), 149.2 (C), 147.9 (C), 147.6 (C), 143.8 (C), 137.3 (C), 131.3 (C), 130.6 (C), 129.7 (CH), 127.1 (CH), 126.6 (C), 125.8 (C), 121.7 (CH), 121.3 (CH), 120.9 (CH), 117.2 (CH), 117.1 (CH), 115.8 (CH), 115.3 (CH), 112.1 (CH), 111.9 (CH), 110.7 (CH), 110.6 (CH), 101.2 (CH), 76.7 (CH), 76.1 (C), 74.9 (C), 73.8 (CH), 64.1 (CH), 62.7 (CH), 56.15 (OMe), 56.11 (OMe), 55.90 (OMe), 55.6 (CH_2_), 52.3 (CH), 51.2 (CH), 48.5 (CH_2_), 37.0 (CH_2_), 31.3 (CH_2_), 28.0 (CH_2_). MS [ESI-MS, positive mode]: found *m*/*z* 817 [M + H]^+^, 839 [M + Na]^+^. HRMS [ESI-MS, positive mode]: MF: C_45_H_44_N_4_O_9_S; found *m*/*z* 839.2728 [M + Na]^+^ [calcd. 839.2727].

### Spectral data of compound 6Ba (±)

Obtained as white solid; yield: 32% (508 mg); mp: 212–214 °C; *R*_f_ 0.56 (5% MeOH in CHCl_3_); IR (KBr, *ν*_max_ cm^−1^): 3511, 3209, 2924, 1713, 1606, 1515; ^1^H NMR (*Py-d*_5_): *δ* 11.73 (1H, s, –NH), 11.29 (1H, s, –NH), 8.38 (1H, d, *J* = 1.2 Hz), 7.84 (1H, d, *J* = 7.8 Hz), 7.42 (1H, d, *J* = 1.2 Hz), 7.37 (1H, d, *J* = 7.8 Hz), 7.28 (3H, m), 7.08 (1H, d, *J* = 1.8 Hz), 7.01 (1H, m), 6.85 (2H, m), 6.60 (1H, dd, *J* = 1.8, 7.8 Hz), 5.79 (1H, s), 4.64 (1H, d, *J* = 12.0 Hz), 4.32 (1H, d, *J* = 12.6 Hz), 4.26 (1H, m), 4.17 (1H, m), 3.94 (1H, d, *J* = 10.8 Hz), 3.88 (3H, s), 3.80 (1H, m), 3.69 (1H, m), 3.62 (3H, s), 3.48 (1H, m), 3.04 (1H, m), 2.95 (1H, m), 2.66 (2H, m), 1.86 (3H, m), 1.66 (1H, m). ^13^C NMR (*Py-d*_5_): *δ* 193.3 (C=O), 188.9 (C=O), 180.8 (C=O), 179.3 (C=O), 158.7 (C, ^1^*J*_C–F_ = 239.1 Hz), 149.6 (C), 149.5 (C), 148.1 (C), 147.8 (C), 143.8 (C), 140.1 (C), 139.6 (CH), 137.1 (CH), 132.0 (C), 129.6 (C), 129.4 (C), 127.1 (C), 122.4 (CH), 120.8 (CH), 117.7 (CH), 117.2 (CH), 115.9 (CH, ^2^*J*_C–F_ = 22.5 Hz), 114.3 (CH, ^2^*J*_C–F_ = 24 Hz), 112.9 (CH), 112.0 (CH), 111.0 (CH, ^3^*J*_C–F_ = 6.45 Hz), 110.7 (CH), 101.0 (CH), 84.4 (C), 76.6 (CH), 75.5 (C), 74.9 (CH), 74.6 (C), 64.1 (CH), 62.6 (CH), 56.5 (OMe), 56.0 (OMe), 55.7 (CH_2_), 52.5 (CH), 51.7 (CH), 48.2 (CH_2_), 36.9 (CH_2_), 31.1 (CH_2_), 28.0 (CH_2_). MS [ESI-MS, positive mode]: found *m*/*z* 953 [M + Na]^+^. HRMS [ESI-MS, positive mode]: MF: C_44_H_40_N_4_O_8_IFS; found *m*/*z* 953.1488 [M + Na]^+^ [calcd. 953.1493].

### Spectral data of compound 6Bb (±)

Obtained as white solid; yield: 37% (587 mg); mp: 220–222 °C; *R*_f_ 0.48 (5% MeOH in CHCl_3_); ^1^H NMR (*Py-d*_5_): *δ* 12.25 (1H, s, –NH), 11.88 (1H, s, –NH), 8.31 (1H, s), 7.59 (1H, d, *J* = 7.8 Hz), 7.30 (1H, d, *J* = 7.8 Hz), 7.14 (1H, d, *J* = 7.8 Hz), 7.09 (1H, d, *J* = 8.4 Hz), 7.06 (1H, s), 7.02 (1H, s), 6.96 (1H, m), 6.81 (2H, m), 6.76 (2H, d, *J* = 7.8 Hz), 5.82 (1H, s), 4.32 (1H, d, *J* = 12.0 Hz), 4.25 (1H, m), 4.21 (1H, d, *J* = 12.0 Hz), 4.14 (1H, m), 3.93 (1H, d, *J* = 10.8 Hz), 3.83 (1H, m), 3.73 (3H, s), 3.71 (1H, m), 3.65 (1H, m), 3.63 (3H, s), 3.12 (1H, d, *J* = 12.0 Hz), 2.99 (1H, m), 2.63 (2H, m), 1.84 (1H, m), 1.76 (1H, m), 1.67 (2H, m). ^13^C NMR (*Py-d*_5_): *δ* 190.4 (C=O), 189.2 (C=O), 181.0 (C=O), 179.6 (C=O), 158.6 (C, ^1^*J*_C–F_ = 237.0 Hz), 149.1 (C), 149.0 (C), 147.8 (C), 147.4 (C), 143.5 (C), 139.8 (C), 139.1 (CH), 137.4 (CH), 131.1 (C), 130.1 (C), 128.3 (C), 127.0 (C), 120.7 (CH), 120.3 (CH), 117.2 (CH), 117.1 (CH), 116.2 (CH, ^2^*J*_C–F_ = 24.0 Hz), 115.0 (CH, ^2^*J*_C–F_ = 24 Hz), 112.6 (CH), 112.5 (2 × CH), 111.2 (CH, ^3^*J*_C–F_ = 7.5 Hz), 101.7 (CH), 84.5 (C), 76.4 (CH), 76.0 (C), 75.3 (C), 73.6 (CH), 64.8 (CH), 63.5 (CH), 56.1 (OMe), 56.0 (OMe), 55.5 (CH_2_), 51.9 (CH), 51.0 (CH), 48.2 (CH_2_), 37.1 (CH_2_), 31.4 (CH_2_), 28.3 (CH_2_). MS [ESI-MS, positive mode]: found *m*/*z* 953 [M + Na]^+^. HRMS [ESI-MS, positive mode]: MF: C_44_H_40_N_4_O_8_IFS; found *m*/*z* 953.1487 [M + Na]^+^ [calcd. 953.1493].

### Spectral data of compound 6Ca (±)

Obtained as white solid; yield: 31% (455 mg); mp: 180–182 °C; *R*_f_ 0.54 (5% MeOH in CHCl_3_); IR (KBr, *ν*_max_ cm^−1^): 3514, 3200, 1713, 1610, 1515; ^1^H NMR (*Py-d*_5_): *δ* 11.61 (1H, s, –NH), 11.39 (1H, s, –NH), 8.29 (1H, s), 7.76 (1H, d, *J* = 7.8 Hz), 7.57 (1H, m), 7.34 (2H, m), 7.28 (2H, m), 7.18 (1H, d, *J* = 7.8 Hz), 7.13 (1H, s), 6.88 (1H, d, *J* = 7.8 Hz), 6.80 (1H, d, *J* = 7.8 Hz), 6.77 (1H, d, *J* = 8.4 Hz), 5.74 (1H, s), 4.57 (1H, d, *J* = 12.0 Hz), 4.38 (1H, m), 4.27 (1H, m), 4.20 (1H, m), 3.92 (1H, d, *J* = 10.8 Hz), 3.82 (1H, m), 3.80 (3H, s), 3.67 (3H, s), 3.64 (1H, m), 3.57 (1H, m), 3.08 (1H, d, *J* = 11.4 Hz), 2.96 (1H, m), 2.71 (1H, m), 2.61 (1H, m), 1.82 (3H, m), 1.66 (1H, m). ^13^C NMR (*Py-d*_5_): *δ* 192.8 (C=O), 189.0 (C=O), 180.5 (C=O), 179.3 (C=O), 149.9 (C), 149.5 (C), 148.0 (C), 147.7 (C), 143.7 (C), 142.6 (C), 139.4 (CH), 137.0 (CH), 131.9 (C), 130.0 (CH), 129.8 (C), 129.3 (C), 127.2 (C), 126.9 (CH), 126.6 (C), 121.9 (CH), 120.9 (CH), 117.5 (CH), 117.3 (CH), 112.8 (CH), 112.0 (CH), 111.8 (CH), 111.2 (CH), 101.1 (CH), 84.5 (C), 76.7 (CH), 75.2 (2 × C), 74.4 (CH), 64.6 (CH), 62.4 (CH), 56.3 (OMe), 56.1 (OMe), 55.3 (CH_2_), 52.4 (CH), 51.4 (CH), 48.2 (CH_2_), 36.9 (CH_2_), 31.0 (CH_2_), 27.9 (CH_2_). MS [ESI-MS, positive mode]: found *m*/*z* 947 [M + H] ^+^. HRMS [ESI-MS, positive mode]: MF: C_44_H_40_ClIN_4_O_8_S; found *m*/*z* 947.1375 [M + H]^+^ [calcd. 947.1378].

### Spectral data of compound 6Cb (±)

Obtained as white solid; yield: 35% (549 mg); mp: 230–232 °C; *R*_f_ 0.46 (5% MeOH in CHCl_3_); ^1^H NMR (*Py-d*_5_): *δ* 12.23 (1H, s, –NH), 12.06 (1H, s, –NH), 8.30 (1H, s), 7.59 (1H, m), 7.55 (1H, dd, *J* = 1.8, 8.4 Hz), 7.17 (1H, dd, *J* = 1.8, 8.4 Hz), 7.10 (2H, dd, *J* = 3.0, 8.4 Hz), 7.02 (2H, m), 6.79 (1H, d, *J* = 8.4 Hz), 6.75 (2H, m), 6.68 (1H, d, *J* = 7.8 Hz), 5.80 (1H, s), 4.28 (2H, d, *J* = 12.0 Hz), 4.18 (1H, m), 4.14 (1H, m), 3.92 (1H, d, *J* = 10.8 Hz), 3.82 (2H, t, *J* = 10.8 Hz), 3.68 (3H, s), 3.64 (3H, s), 3.62 (1H, m), 3.10 (1H, m), 2.99 (1H, m), 2.67 (1H, m), 2.60 (1H, m), 1.85 (1H, m), 1.79 (1H, m), 1.70 (2H, m). ^13^C NMR (*Py-d*_5_): *δ* 189.8 (2 × C=O), 180.8 (C=O), 179.5 (C=O), 149.0 (C), 148.9 (C), 147.7 (C), 147.3 (C), 143.5 (C), 142.4 (C), 139.1 (CH), 137.4 (CH), 131.1 (C), 130.1 (C), 129.9 (CH), 128.5 (C), 127.6 (CH), 127.0 (C), 126.7 (C), 120.5 (CH), 120.1 (CH), 117.1 (CH), 117.0 (CH), 112.8 (CH), 112.6 (CH), 112.5 (CH), 111.8 (CH), 101.7 (CH), 84.5 (C), 76.3 (CH), 76.1 (C), 75.3 (C), 73.3 (CH), 65.5 (CH), 63.6 (CH), 56.13 (OMe), 56.06 (OMe), 55.5 (CH_2_), 51.7 (CH), 50.9 (CH), 48.2 (CH_2_), 37.1 (CH_2_), 31.3 (CH_2_), 28.4 (CH_2_). MS [ESI-MS, positive mode]: found *m*/*z* 947 [M + H] ^+^. HRMS [ESI-MS, positive mode]: MF: C_44_H_40_ClIN_4_O_8_S; found *m*/*z* 969.1193 [M + Na]+[calcd. 969.1198].

### Spectral data of compound 6Da (±)

Obtained as white solid; yield: 32% (452 mg); mp: 202–204 °C; *R*_f_ 0.55 (5% MeOH in CHCl_3_); IR (KBr, *ν*_max_ cm^−1^): 3411, 2930, 1721, 1604, 1518; ^1^H NMR (*Py-d*_5_): *δ* 11.26 (1H, s, –NH), 10.63 (1H, s, –NH), 7.43 (1H, s), 7.28 (2H, t, *J* = 8.4 Hz), 7.23 (2H, m), 7.10 (1H, d, *J* = 2.4 Hz), 7.06 (1H, dd, *J* = 1.2, 7.8 Hz), 7.01 (1H, dd, *J* = 1.2, 7.8 Hz), 6.86 (1H, d, *J* = 7.8 Hz), 6.81 (1H, dd, *J* = 2.4, 8.4 Hz), 6.75 (1H, s), 5.72 (1H, s), 4.38 (3H, m), 4.28 (1H, m), 3.95 (1H, m), 3.81 (1H, m), 3.75 (3H, s), 3.74 (3H, s), 3.71 (2H, m), 3.64 (3H, s), 3.17 (1H, m), 3.00 (1H, m), 2.88 (1H, m), 2.62 (1H, m), 2.23 (3H, s), 2.15 (3H, s), 1.88 (1H, m), 1.79 (1H, m), 1.70 (2H, m). ^13^C NMR (*Py-d*_5_): *δ* 194.0 (C=O), 187.9 (C=O), 180.9 (C=O), 180.4 (C=O), 155.4 (C), 149.4 (C), 149.1 (C), 147.7 (C), 147.5 (C), 140.0 (C), 136.8 (C), 132.3 (CH), 132.0 (C), 130.6 (C), 130.5 (C), 127.8 (C), 126.5 (CH), 124.2 (C), 121.3 (CH), 121.0 (CH), 119.0 (C), 117.3 (CH), 117.1 (CH), 115.0 (CH), 114.5 (CH), 112.4 (CH), 111.7 (CH), 110.8 (CH), 101.9 (CH), 77.1 (CH), 75.5 (C), 74.8 (C), 72.9 (CH), 66.1 (CH), 61.5 (CH), 56.13 (2 × OMe), 56.09 (OMe), 55.2 (CH_2_), 52.5 (CH), 51.1 (CH), 48.5 (CH_2_), 36.7 (CH_2_), 30.6 (CH_2_), 27.5 (CH_2_), 21.1 (CH_3_), 17.2 (CH_3_). MS [ESI-MS, positive mode]: found *m*/*z* 845 [M + H]^+^. HRMS [ESI-MS, positive mode]: MF: C_47_H_48_N_4_O_9_S; found *m*/*z* 845.3238 [M + H]^+^ [calcd. 845.3220].

### Spectral data of compound 6Db (±)

Obtained as white solid; yield: 36% (508 mg); mp: 182–184 °C; *R*_f_ 0.47 (5% MeOH in CHCl_3_); ^1^H NMR (*Py-d*_5_): *δ* 11.83 (1H, s, –NH), 11.79 (1H, s, –NH), 7.47 (1H, s), 7.18 (1H, dd, *J* = 1.2, 7.8 Hz), 7.12 (2H, m), 7.07 (2H, m), 6.84 (3H, m), 6.77 (1H, dd, *J* = 2.4, 8.4 Hz), 6.69 (1H, s), 5.87 (1H, d, *J* = 1.2 Hz), 4.33 (2H, m), 4.27 (1H, d, *J* = 12.6 Hz), 4.19 (1H, m), 3.98 (1H, d, *J* = 10.2 Hz), 3.90 (1H, m), 3.80 (1H, t, *J* = 10.2 Hz), 3.75 (1H, d, *J* = 10.2 Hz), 3.68 (3H, s), 3.64 (3H, s), 3.54 (3H, s), 3.17 (1H, m), 3.04 (1H, m), 2.81 (1H, m), 2.64 (1H, m), 2.26 (3H, s), 2.10 (3H, s), 1.87 (1H, m), 1.79 (1H, m), 1.69 (2H, m). ^13^C NMR (*Py-d*_5_): *δ* 191.0 (C=O), 189.3 (C=O), 181.2 (C=O), 180.6 (C=O), 155.4 (C), 149.1 (C), 149.0 (C), 147.7 (C), 147.4 (C), 140.0 (C), 137.1 (C), 132.3 (CH), 131.6 (C), 130.7 (C), 130.4 (C), 127.9 (C), 126.5 (CH), 124.3 (C), 120.7 (CH), 120.3 (CH), 119.1 (C), 117.2 (CH), 116.9 (CH), 115.3 (CH), 114.1 (CH), 112.6 (CH), 112.4 (CH), 110.7 (CH), 101.4 (CH), 76.6 (CH), 76.1 (C), 75.5 (C), 73.4 (CH), 65.2 (CH), 63.3 (CH), 56.1 (OMe), 56.0 (OMe), 55.8 (OMe), 55.0 (CH_2_), 51.8 (CH), 50.7 (CH), 48.4 (CH_2_), 36.9 (CH_2_), 31.3 (CH_2_), 28.1 (CH_2_), 21.3 (CH_3_), 17.2 (CH_3_). MS [ESI-MS, positive mode]: found *m*/*z* 845 [M + H]^+^. HRMS [ESI-MS, positive mode]: MF: C_47_H_48_N_4_O_9_S; found *m*/*z* 845.3209 [M + H]^+^ [calcd. 845.3220].

### Spectral data of compound 6Ea (±)

Obtained as white solid; yield: 31% (441 mg); mp: 172–174 °C; *R*_f_ 0.51 (5% MeOH in CHCl_3_); ^1^H NMR (*Py-d*_5_): *δ* 11.16 (1H, s, –NH), 10.69 (1H, s, –NH), 7.46 (1H, s), 7.30 (2H, m), 7.28 (1H, s), 7.25 (2H, d, *J* = 9.6 Hz), 7.08 (1H, d, *J* = 7.8 Hz), 7.03 (1H, d, *J* = 7.8 Hz), 6.95 (1H, d, *J* = 7.8 Hz), 6.83 (1H, d, *J* = 7.8 Hz), 6.73 (1H, s), 5.73 (1H, s), 4.37 (3H, m), 3.95 (1H, d, *J* = 10.2 Hz), 3.87 (1H, m), 3.81 (1H, m), 3.76 (3H, s), 3.73 (3H, s), 3.71 (1H, m), 3.16 (1H, m), 3.01 (1H, m), 2.86 (1H, m), 2.63 (1H, m), 2.24 (3H, s), 2.15 (6H, s), 2.01 (1H, m), 1.90 (1H, m), 1.82 (2H, m), 1.69 (1H, m). ^13^C NMR (*Py-d*_5_): *δ* 192.6 (C=O), 187.8 (C=O), 180.5 (C=O), 179.9 (C=O), 148.7 (C), 148.5 (C), 147.1 (C), 146.8 (C), 140.4 (C), 139.3 (C), 131.7 (CH), 131.4 (C), 130.2 (3 × C), 129.9 (C), 129.6 (CH), 127.5 (CH), 126.0 (C), 125.9 (CH), 120.5 (CH), 120.3 (CH), 118.4 (C), 116.7 (CH), 116.5 (CH), 111.8 (CH), 111.4 (CH), 109.7 (CH), 101.2 (CH), 76.2 (CH), 75.0 (C), 74.1 (C), 72.6 (CH), 65.0 (CH), 61.4 (CH), 55.5 (2 × OMe), 54.4 (CH_2_), 51.7 (CH), 50.5 (CH), 48.0 (CH_2_), 36.1 (CH_2_), 30.2 (CH_2_), 27.0 (CH_2_), 20.7 (CH_3_), 20.6 (CH_3_), 16.6 (CH_3_). MS [ESI-MS, positive mode]: found *m*/*z* 829 [M + H]^+^. HRMS [ESI-MS, positive mode]: MF: C_47_H_49_N_4_O_8_S; found *m*/*z* 829.3268 [M + H]^+^ [calcd. 829.3271].

### Spectral data of compound 6Eb (±)

Obtained as white solid; yield: 36% (512 mg); mp: 169–171 °C; *R*_f_ 0.45 (5% MeOH in CHCl_3_); IR (KBr, *ν*_max_ cm^−1^): 3404, 2926, 1712, 1605, 1517, 1485; ^1^H NMR (*Py-d*_5_): *δ* 7.41 (1H, s), 7.17 (2H, m), 7.09 (3H, m), 6.90 (1H, d, *J* = 7.8 Hz), 6.82 (2H, m), 6.78 (1H, d, *J* = 7.8 Hz), 6.64 (1H, s), 5.85 (1H, s), 4.27 (3H, m), 4.16 (1H, m), 3.93 (1H, d, *J* = 9.6 Hz), 3.83 (2H, q, *J* = 9.6, 21 Hz), 3.70 (1H, d, *J* = 9.6 Hz), 3.63 (3H, s), 3.58 (3H, s), 3.13 (1H, m), 2.99 (1H, m), 2.78 (1H, m), 2.59 (1H, m), 2.21 (3H, s), 2.05 (3H, s), 2.00 (3H, s), 1.84 (1H, m), 1.75 (2H, m), 1.64 (1H, m). ^13^C NMR (*Py-d*_5_): *δ* 190.6 (C=O), 188.8 (C=O), 180.8 (C=O), 180.1 (C=O), 148.6 (C), 148.4 (C), 147.2 (C), 146.8 (C), 140.7 (C), 139.5 (C), 131.8 (CH), 131.1 (C), 130.2 (2 × C), 130.1 (C), 129.8 (C), 129.7 (CH), 127.5 (CH), 126.0 (C), 125.9 (CH), 120.1 (CH), 119.7 (CH), 118.5 (C), 116.6 (CH), 116.4 (CH), 111.9 (CH), 111.7 (CH), 109.7 (CH), 100.7 (CH), 76.1 (CH), 75.5 (C), 74.7 (C), 73.0 (CH), 64.5 (CH), 62.5 (CH), 55.5 (OMe), 55.4 (OMe), 54.5 (CH_2_), 51.1 (CH), 50.2 (CH), 47.9 (CH_2_), 36.3 (CH_2_), 30.7 (CH_2_), 27.5 (CH_2_), 20.8 (CH_3_), 20.7 (CH_3_), 16.7 (CH_3_). MS [ESI-MS, positive mode]: found *m*/*z* 829 [M + H] ^+^. HRMS [ESI-MS, positive mode]: MF: C_47_H_49_N_4_O_8_S; found *m*/*z* 829.3268 [M + H]^+^ [calcd. 829.3271].

### Spectral data of compound 7A (±)

Obtained as white solid; yield: 54% (636 mg); mp: 192–194 °C; *R*_f_ 0.52 (5% MeOH in CHCl_3_); UV absorption maximas (*λ*_max_ nm, methanol) 314, 228; IR (KBr, *ν*_max_ cm^−1^): 3511, 2960, 1720, 1607, 1515; ^1^H NMR (*Py-d*_5_): *δ* 11.82 (1H, s, –NH), 7.87 (2H, m), 7.84 (2H, m), 7.76 (1H, t, *J* = 7.2 Hz), 7.69 (1H, d, *J* = 7.2 Hz), 7.41 (1H, t, *J* = 7.2 Hz), 7.37 (1H, d, *J* = 1.2 Hz), 7.30 (1H, t, *J* = 7.8 Hz), 7.16 (3H, m), 7.02 (1H, d, *J* = 7.8 Hz), 6.79 (1H, dd, *J* = 1.2, 8.4 Hz), 6.75 (1H, d, *J* = 8.4 Hz), 6.70 (1H, s), 6.03 (1H, s), 4.96 (1H, m), 4.61 (2H, m), 4.45 (1H, d, *J* = 11.4 Hz), 4.38 (1H, m), 4.01 (1H, dd, *J* = 9.6, 12 Hz), 3.71 (3H, s), 3.36 (3H, s), 3.14 (1H, m), 2.90 (1H, m), 2.74 (1H, m), 2.54 (1H, t, *J* = 7.2 Hz), 1.98 (1H, m), 1.90 (2H, m), 1.78 (1H, m), 1.70 (1H, m), 1.64 (1H, m), 1.50 (1H, m), 1.31 (1H, m). ^13^C NMR (*Py-d*_5_): *δ* 204.6 (C=O), 197.4 (C=O), 186.5 (C=O), 181.2 (C=O), 149.3 (C), 148.1 (C), 147.7 (C), 147.2 (C), 144.0 (C), 143.2 (C), 138.1 (C), 133.2 (C), 131.7 (CH), 131.5 (C), 131.3 (C), 129.9 (CH), 128.83 (CH), 128.79 (CH), 128.0 (CH), 127.5 (C), 126.9 (C), 125.9 (CH), 123.7 (CH), 121.9 (CH), 121.8 (CH), 121.7 (CH), 121.4 (CH), 117.1 (CH), 116.3 (CH), 113.2 (CH), 112.3 (CH), 110.8 (CH), 103.0 (CH), 79.3 (C), 75.3 (C), 73.7 (CH), 66.0 (CH), 64.6 (CH), 56.1 (OMe), 55.6 (OMe), 54.8 (CH), 52.6 (CH), 51.8 (CH_2_), 50.7 (CH), 48.6 (CH_2_), 31.3 (CH_2_), 29.5 (CH_2_), 28.2 (CH_2_), 26.7 (CH_2_). MS [ESI-MS, positive mode]: found *m*/*z* 804 [M + H]^+^, 826 [M + Na]^+^. HRMS [ESI-MS, positive mode]: MF: C_49_H_45_N_3_O_8_; found *m*/*z* 826.3113 [M + Na]^+^ [calcd. 826.3104].

### Spectral data of compound 7B (±)

Obtained as yellowish white solid; yield: 56% (629 mg); mp: 163–165 °C; *R*_f_ 0.50 (5% MeOH in CHCl_3_); ^1^H NMR (*Py-d*_5_): *δ* 12.17 (1H, s, –NH), 8.06 (2H, d, *J* = 7.2 Hz), 7.88 (1H, d, *J* = 8.4 Hz), 7.84 (1H, s), 7.67 (2H, m), 7.62 (1H, t, *J* = 7.2 Hz), 7.47 (1H, d, *J* = 7.2 Hz), 7.34 (1H, d, *J* = 7.8 Hz), 7.30 (1H, s), 7.19 (1H, d, *J* = 8.4 Hz), 7.15 (1H, d, *J* = 8.4 Hz), 7.03 (1H, s), 6.91 (1H, d, *J* = 8.4 Hz), 6.83 (1H, d, *J* = 7.8 Hz), 5.81 (1H, s), 4.52 (1H, d, *J* = 11.4 Hz), 4.30 (1H, d, *J* = 12.0 Hz), 4.23 (1H, m), 4.09 (1H, m), 3.88 (3H, s), 3.74 (2H, m), 3.66 (3H, s), 2.62 (2H, m), 2.52 (1H, m), 2.35 (1H, m), 1.84 (2H, m), 1.70 (5H, m), 1.58 (1H, m). ^13^C NMR (*Py-d*_5_): *δ* 205.1 (C=O), 191.4 (C=O), 189.4 (C=O), 179.9 (C=O), 148.8 (C), 148.6 (C), 147.1 (C), 147.0 (C), 142.9 (C), 142.2 (C), 138.2 (CH), 135.0 (CH), 132.1 (C), 131.8 (CH), 130.75 (C), 130.67 (C), 130.45 (C), 128.9 (C), 128.2 (CH), 128.1 (CH), 125.3 (CH), 123.6 (C), 122.9 (CH), 121.6 (CH), 120.4 (CH), 120.3 (CH), 116.7 (CH), 116.4 (CH), 112.4 (CH), 111.5 (CH), 111.1 (CH), 100.7 (CH), 84.2 (C), 77.5 (C), 74.1 (C), 73.5 (CH), 73.0 (CH), 64.5 (CH), 62.9 (CH), 55.7 (OMe), 55.4 (OMe), 52.9 (CH), 51.4 (CH), 48.2 (CH_2_), 47.7 (CH_2_), 30.7 (CH_2_), 30.4 (CH_2_), 27.7 (CH_2_), 27.0 (CH_2_). MS [ESI-MS, positive mode]: found *m*/*z* 930 [M + H]^+^. HRMS [ESI-MS, positive mode]: MF: C_49_H_45_IN_3_O_8_; found *m*/*z* 930.2252 [M + H]^+^ [calcd. 930.2251].

### Spectral data of compound 7C (±)

Obtained as white solid; yield: 50% (607 mg); mp: 204–206 °C; *R*_f_ 0.49 (5% MeOH in CHCl_3_); ^1^H NMR (*Py-d*_5_): *δ* 11.78 (1H, s, –NH), 8.08 (1H, dd, *J* = 2.4, 6.6 Hz), 8.05 (1H, dd, *J* = 2.4, 8.4 Hz), 7.83 (1H, dd, *J* = 2.4, 8.4 Hz), 7.74 (1H, m), 7.62 (2H, m), 7.47 (1H, dd, *J* = 1.8, 7.8 Hz), 7.41 (1H, dd, *J* = 1.8, 7.8 Hz), 7.31 (1H, s), 7.26 (1H, d, *J* = 8.4 Hz), 7.18 (2H, m), 7.12 (1H, d, *J* = 7.8 Hz), 6.96 (1H, d, *J* = 7.8 Hz), 6.91 (2H, m), 5.86 (1H, s), 4.46 (1H, m), 4.32 (2H, m), 4.11 (1H, m), 3.87 (3H, s), 3.82 (3H, s), 3.76 (2H, m), 3.49 (1H, m), 3.24 (1H, d, *J* = 10.8 Hz), 3.20 (1H, d, *J* = 11.4 Hz), 3.02 (1H, m), 2.57 (2H, m), 1.86 (1H, m), 1.71 (2H, m), 1.64 (1H, m). ^13^C NMR (*Py-d*_5_): *δ* 205.0 (C=O), 190.9 (C=O), 190.5 (C=O), 180.9 (C=O), 149.8 (C), 149.6 (C), 148.3 (C), 147.9 (C), 143.9 (C), 143.4 (C), 133.2 (C), 132.51 (CH), 132.47 (C), 131.2 (C), 131.0 (C), 130.3 (C), 129.7 (CH), 128.7 (CH), 128.6 (CH), 126.8 (CH), 126.4 (C), 126.2 (CH), 125.1 (CH), 122.4 (CH), 121.9 (CH), 121.7 (CH), 121.5 (CH), 117.5 (CH), 117.3 (CH), 111.9 (CH), 111.3 (CH), 110.6 (CH), 100.9 (CH), 78.7 (C), 77.4 (CH), 74.4 (C), 73.8 (CH), 63.8 (CH), 61.2 (CH), 56.3 (OMe), 56.2 (OMe), 55.9 (CH_2_), 53.0 (CH), 52.4 (CH), 48.4 (CH_2_), 37.2 (CH_2_), 31.3 (CH_2_), 27.9 (CH_2_). MS [ESI-MS, positive mode]: found *m*/*z* 822 [M + H]^+^, 844 [M + Na]^+^. HRMS [ESI-MS, positive mode]: MF: C_48_H_43_N_3_O_8_S; found *m*/*z* 844.2670 [M + Na]^+^ [calcd. 844.2669].

### Spectral data of compound 7D (±)

Obtained as yellowish white solid; yield: 49% (588 mg); mp: 160–162 °C; *R*_f_ 0.48 (5% MeOH in CHCl_3_); UV absorption maximas (*λ*_max_ nm, methanol) 284, 254, 215; ^1^H NMR (*Py-d*_5_): *δ* 8.05 (2H, m), 7.87 (1H, d, *J* = 8.4 Hz), 7.76 (1H, d, *J* = 8.4 Hz), 7.65 (1H, t, *J* = 7.8 Hz), 7.60 (1H, d, *J* = 7.2 Hz), 7.39 (1H, d, *J* = 7.8 Hz), 7.31 (1H, d, *J* = 1.2 Hz), 7.27 (1H, d, *J* = 8.4 Hz), 7.20 (1H, m), 7.15 (1H, s), 7.07 (2H, m), 7.01 (1H, d, *J* = 7.8 Hz), 6.96 (1H, dd, *J* = 1.2, 7.8 Hz), 5.88 (1H, s), 4.46 (1H, d, *J* = 12.6 Hz), 4.33 (2H, m), 4.12 (1H, m), 3.88 (3H, s), 3.76 (2H, m), 3.73 (3H, s), 3.70 (1H, m), 3.24 (1H, d, *J* = 10.8 Hz), 3.12 (1H, d, *J* = 11.4 Hz), 3.00 (1H, m), 2.72 (1H, m), 2.63 (1H, m), 2.13 (3H, s), 1.87 (1H, m), 1.77 (2H, m), 1.68 (1H, m). ^13^C NMR (*Py-d*_5_): *δ* 204.5 (C=O), 192.0 (C=O), 188.1 (C=O), 180.4 (C=O), 149.0 (C), 148.7 (C), 147.6 (C), 147.1 (C), 142.8 (C), 140.8 (C), 132.8 (C), 131.9 (CH), 131.8 (C), 130.8 (C), 130.5 (C), 130.3 (C), 129.8 (CH, C), 128.1 (CH), 128.0 (CH), 127.3 (CH), 126.1 (C), 125.5 (CH), 124.3 (CH), 121.8 (CH), 120.9 (CH), 120.6 (CH), 116.8 (CH), 116.5 (CH), 111.4 (CH), 110.8 (CH), 109.9 (CH), 100.4 (CH), 78.2 (C), 77.1 (CH), 74.1 (C), 73.1 (CH), 64.1 (CH), 60.5 (CH), 55.6 (OMe), 55.5 (OMe), 55.2 (CH_2_), 51.8 (CH), 51.6 (CH), 47.7 (CH_2_), 36.8 (CH_2_), 30.8 (CH_2_), 27.7 (CH_2_), 20.8 (CH_3_). MS [ESI-MS, positive mode]: found *m*/*z* 836 [M + H]^+^. HRMS [ESI-MS, positive mode]: MF: C_49_H_46_N_3_O_8_S; found *m*/*z* 836.3009 [M + H]^+^ [calcd. 836.3006].

## Conflicts of interest

There are no conflicts of interest to declare.

## Supplementary Material

RA-008-C8RA02725K-s001
