# Supplementary material for: Synthesis of diversely substituted bis-pyrrolizidino/ thiopyrrolizidino oxindolo/acenaphthyleno curcuminoids via sequential azomethine ylide cycloaddition
Source: RSC Adv. 2018 May 23;8(34):18938–51. doi: 10.1039/c8ra02725k (PMC9080697; doi:10.1039/c8ra02725k)
Supplement: RA-008-C8RA02725K-s001 [file RA-008-C8RA02725K-s001.pdf]

Electronic Supplementary Material (ESI) for RSC Advances.
This journal is © The Royal Society of Chemistry 2018

Synthesis of diversely substituted bis-pyrrolizidino/thiopyrrolizidino oxindolo/acenaphthyleno curcuminoids via sequential azomethine ylide cycloaddition

Meenakshi Singh[a], Abhijit Hazra,[a]* Yogesh P. Bharitkar,[a]* Ritu Kalia[a], Ashutosh Sahoo[b], Sneha Saha[a], Ravichandiran V[a], Shekhar Ghosh[b], Nirup B. Mondal[b]

[a]National Institute of Pharmaceutical Education and Research (NIPER), Indian Institute of Chemical Biology, 4, Raja S. C. Mullick Road, Jadavpur, Kolkata - 700 032, India

[b]Department of Organic and Medicinal Chemistry, Indian Institute of Chemical Biology, Council of Scientific and Industrial Research, 4, Raja S. C. Mullick Road, Jadavpur, Kolkata - 700 032, India

E-mail: apuhazra@gmail.com/ yogeshbharitkar@gmail.com

Phone: +9133-2499-5900; Fax: +9133-2429-2422

Supplymentary information and Documents:

General Experimental Information:	2-3
Spectral data of compounds **4Aa-4Fd**:	3-18
Spectral data of compounds **5Aa-5Bb**:	18-20
Spectral data of compounds **6Aa-6Eb**:	20-26
Spectral data of compounds **7A-7D**:	26-29
Spectral copy of compounds **4Aa-4Fd**:	30-101
Spectral copy of compounds **5Aa-5Bb**:	102-113
Spectral copy of compounds **6Aa-6Eb**:	114-143
Spectral copy of compounds **7A-7D**:	144-155
2D NMR figure, **6Aa, 6Ab, 7B** and **7D**:	156-156
2D NMR spectra, **6Aa, 6Ab, 7B** and **7D**:	157-173

Experimental Section:

General Information:

Chemistry:

All the compounds evaluated in this work were synthesized in two step sequences. Melting points were determined in capillaries and are uncorrected. IR spectra were recorded as KBr pellets using a JASCO 410 FTIR spectrometer. The NMR spectra were recorded using a Bruker 600 DPX spectrometer operating at 600 MHz for ^{1}H and 150 MHz for ^{13}C in *Pyridine-d_5* with TMS as internal standard and the chemical shifts are reported in δ units. Mass spectra (positive mode) were obtained on a LC-ESI-Q-TOF micro mass spectrometer in the electrospray ionization mode. Curcumin was isolated from *Curcuma longa* in the usual way. Isatins, acenaphthoquinone and α-amino acids were purchased from Alfa-Aesar Company. All other solvents and chromatographic absorbents were procured from E. Merck (Germany) and SRL (India) Ltd. unless otherwise indicated. Thin layer chromatography was performed on pre-coated silica gel 60 F_{254} aluminum sheets (E. Merck, Germany) using the solvent system 5% MeOH in $CHCl_3$ and spots were developed using UV irradiation, Iodine and Liebermann-Burchard reagent. Compounds were separated using AKROS –"Automatic TLC Smart Flash" of Yamazen Corporation.

Typical experimental procedure for synthesis of mono cycloaddition products 3Aa (±):

A mixture of **1** (13.6 mmol, 5 g), isatin (13.6 mmol, 2.00 g) and proline (13.6 mmol, 1.56 g) was taken in a round bottom flask, dissolved in 50 mL methanol, and heated to reflux for 8 h. After completion of the reaction as evident from TLC, the solvent was removed and the crude product was subjected to column chromatography using gradual increasing methanol in chloroform as eluant using flash chromatography. The product was crystallized from chloroform-methanol mixture (85%, 6.56 g).

Typical experimental procedure for synthesis of mixed cycloaddition products:

A mixture of **3Aa (±)** (1.76 mmol, 1 g), 5-iodoisatin (1.76 mmol, 481 mg) and proline (1.76 mmol, 205 mg) or thioproline (1.76 mmol, 235 mg) was taken in a round bottom flask, dissolved in 50 mL methanol, and heated to reflux for 8 h. The solvent was removed after completion of the reaction, evident from TLC, and the crude product was subjected to column chromatography using gradual increasing methanol in chloroform as eluant using flash chromatography to isolate diastereomeric mixed pyrrolizidino/pyrrilizidino-thiopyrrolizidino

dispiro oxindolo curcuminoids. The product was crystallized from chloroform-methanol mixture.

Compound 4Aa (±):

Color: Obtained as white solid; **Yield**: 20% (314 mg); **Mp**: 219-221 ºC; $\mathbf{R_f}$ 0.49 (6 % MeOH in $CHCl_3$);

IR (KBr, ν_{max} Cm^{-1}): 3376, 2960, 2869, 1722, 1612;

^{1}H NMR (*Py-d$_5$*): δ 11.51 (1H, s, -NH), 11.19 (1H, s, -NH), 7.97 (1H, s), 7.75 (1H, d, *J*=7.8 Hz), 7.38 (2H, m), 7.29 (2H, m), 7.22 (2H, m), 7.13 (1H, d, *J*=1.2 Hz), 6.99 (1H, t, *J*=7.8 Hz), 6.96 (1H, d, *J*=6.6 Hz), 6.87 (1H, d, *J*=8.4 Hz), 6.75 (1H, dd, *J*=1.8, 8.4 Hz), 5.80 (1H, s), 4.59 (1H, d, *J*=12.6 Hz), 4.43 (1H, d, *J*=12.6 Hz), 4.21 (2H, m), 3.82 (3H, s), 3.75 (1H, m), 3.66 (3H, s), 3.64 (1H, m), 2.74 (2H, m), 2.62 (2H, m), 1.87 (3H, m), 1.82 (2H, m), 1.70 (3H, m).

^{13}C NMR (*Py-d$_5$*): δ 191.8 (-C=O), 190.8 (-C=O), 181.1 (-C=O), 180.2 (-C=O), 149.6 (-C), 149.2 (-C), 147.7 (-C), 147.6 (-C), 143.9 (-C), 143.6 (-C), 138.9 (-CH), 135.8 (-CH), 131.9 (-C), 130.9 (-C), 129.7 (-CH), 129.5 (-C), 127.1 (-C), 126.9 (-CH), 121.7 (2×-CH), 121.0 (-CH), 117.4 (-CH), 117.1 (-CH), 113.2 (-CH), 111.7 (-CH), 111.4 (-CH), 110.6 (-CH), 101.4 (-CH), 84.7 (-C), 74.49 (-C), 74.46 (-C), 74.42 (-CH), 73.5 (-CH), 65.2 (-CH), 63.6 (-CH), 56.3 (-OMe), 56.1 (-OMe), 52.8 (-CH), 52.6 (-CH), 48.6 (-CH_2), 48.3 (-CH_2), 31.13 (-CH_2), 31.06 (-CH_2), 28.1 (-CH_2), 27.8 (-CH_2).

MS [ESI-MS, positive mode]: found *m/z* 895 [M+H]$^{+}$.

HRMS [ESI-MS, positive mode]: MF: $C_{45}H_{43}N_4O_8I$; found m/z 917.2042 [M+Na]$^{+}$ [calcd. 917.2023].

Compound 4Ab (±):

Color: Obtained as white solid; **Yield**: 23% (362 mg); **Mp**: 192-194 ºC; $\mathbf{R_f}$ 0.43 (6 % MeOH in $CHCl_3$);

IR (KBr, ν_{max} Cm^{-1}): 3383, 2960, 2872, 1718, 1611.

^{1}H NMR (*Py-d$_5$*): δ 12.18 (1H, s, -NH), 11.81 (1H, s, -NH), 7.91 (1H, d, *J*=1.8 Hz), 7.63 (1H, dd, *J*=1.8, 8.4 Hz), 7.25 (2H, m), 7.19 (1H, m), 7.17 (2H, d, *J*=7.8 Hz), 7.07 (1H, d, *J*=1.8 Hz), 7.00 (2H, m), 6.92 (1H, d, *J*=7.2 Hz), 6.85 (2H, m), 5.94 (1H, s), 4.42 (2H, t, *J*=12.0 Hz), 4.20 (1H, m), 4.10 (1H, m), 3.85 (1H, m), 3.79 (3H, s), 3.70 (1H, m), 3.67 (3H, s), 2.69 (2H, m), 2.61 (2H, m), 1.86 (2H, m), 1.79 (1H, m), 1.69 (5H, m).

^{13}C NMR (*Py-d$_5$*): δ 191.6 (-C=O), 189.7 (-C=O), 181.2 (-C=O), 180.5 (-C=O), 149.2 (-C), 149.1 (-C), 147.52 (-C), 147.50 (-C), 143.9 (-C), 143.5 (-C), 138.8 (-CH), 135.8 (-CH), 131.4 (-C), 131.2 (-C), 129.8 (-CH), 129.5 (-C), 127.1 (-CH), 126.6 (-C), 121.8 (-CH), 120.7 (2×-CH), 117.2 (-CH), 117.0 (-CH), 113.0 (-CH), 112.2 (-CH), 112.1 (-CH), 110.6 (-CH), 101.3 (-CH), 84.7 (-C), 74.9 (2×-C), 73.7 (-CH), 73.6 (-CH), 65.1 (-CH), 64.3 (-CH), 56.2 (-OMe), 56.1 (-OMe), 52.5 (-CH), 51.8 (-CH), 48.5 (-CH_2), 48.2 (-CH_2), 31.4 (-CH_2), 31.3 (-CH_2), 28.4 (-CH_2), 28.0 (-CH_2).

MS [ESI-MS, positive mode]: found *m/z* 895 $[M+H]^+$, 917 $[M+Na]^+$.
HRMS [ESI-MS, positive mode]: MF: C45H43N_4O_8I; found m/z 917.2034 $[M+Na]^+$ [calcd. 917.2023].

<u>Compound 4Ac (±):</u>
Color: Obtained as white solid; **Yield**: 21% (330 mg); **Mp**: 175-177 ºC; **R$_f$** 0.39 (6 % MeOH in $CHCl_3$);
^{1}H NMR (*Py-d$_5$*): δ 11.93 (1H, s, -NH), 11.54 (1H, s, -NH), 8.26 (1H, s), 7.60 (1H, m), 7.35 (1H, s), 7.28 (1H, t, *J*=7.8 Hz), 7.23 (2H, m), 7.16 (1H, m), 7.05 (2H, m), 6.93 (1H, d, *J*=8.4 Hz), 6.89 (2H, m), 6.54 (1H, d, *J*=7.8 Hz), 6.05 (1H, s), 4.71 (1H, m), 4.62 (1H, d, *J*=12 Hz), 4.33 (1H, m), 4.11 (1H, d, *J*=12 Hz), 3.95 (1H, m), 3.71 (3H, s), 3.55 (3H, s), 3.23 (1H, m), 2.83 (2H, m), 1.96 (1H, m), 1.90 (2H, m), 1.74 (2H, m), 1.64 (2H, m), 1.40 (2H, m).

^{13}C NMR (*Py-d$_5$*): δ 196.3 (-C=O), 194.5 (-C=O), 181.2 (-C=O), 179.4 (-C=O), 149.3 (-C), 148.3 (-C), 147.7 (-C), 147.5 (-C), 144.6 (-C), 144.1 (-C), 138.7 (-CH), 136.2 (-CH), 131.4 (-C), 131.0 (-C), 129.9 (-CH), 127.7 (-CH), 126.9 (-C), 126.8 (-C), 124.1 (-CH), 121.7 (-CH), 121.4 (-CH), 117.1 (-CH), 116.5 (-CH), 113.0 (-CH), 112.6 (-CH), 112.3 (-CH), 110.7 (-CH), 102.9 (-CH), 84.3 (-C), 76.5 (-C), 75.1 (-C), 73.8 (-CH), 65.7 (-CH), 64.6 (-CH), 56.1 (-OMe), 55.7 (-OMe), 53.3 (-CH), 52.7 (-CH), 52.0 (-CH), 51.5 (-CH_2), 48.5 (-CH_2), 31.3 (-CH_2), 29.9 (-CH_2), 28.2 (-CH_2), 26.8 (-CH_2).

MS [ESI-MS, positive mode]: found *m/z* 895 $[M+H]^+$, 917 $[M+Na]^+$.
HRMS [ESI-MS, positive mode]: MF: $C_{45}H_{43}N_4O_8I$; found m/z 917.2017 $[M+Na]^+$ [calcd. 917.2023].

<u>Compound 4Ad (±):</u>
Color: Obtained as white solid; **Yield**: 24% (378 mg); **Mp**: 218-220 ºC; **R$_f$** 0.38 (6 % MeOH in $CHCl_3$);

IR (KBr, ν_{max} Cm^{-1}): 3391, 2960, 1720, 1613;

^{1}H NMR (*Py-d$_5$*): δ 11.77 (1H, s, -NH), 11.51 (1H, s, -NH), 8.33 (1H, s), 7.67 (1H, d, *J*=7.2 Hz), 7.60 (1H, d, *J*=8.4 Hz), 7.36 (1H, s), 7.30 (1H, t, *J*=7.8 Hz), 7.20 (1H, m), 7.15 (2H, m), 7.01 (1H, d, *J*=7.8 Hz), 6.97 (1H, m), 6.92 (1H, m), 6.89 (1H, s), 6.54 (1H, d, *J*=8.4 Hz), 5.97 (1H, s), 4.96 (1H, m), 4.67 (1H, q, *J*=8.4 Hz), 4.59 (1H, d, *J*=12.6 Hz), 4.37 (1H, m), 4.23 (1H, d, *J*=12.0 Hz), 3.98 (1H, m), 3.70 (3H, s), 3.56 (3H, s), 3.15 (1H, m), 2.88 (1H, m), 2.74 (2H, m), 1.96 (1H, m), 1.89 (2H, m), 1.77 (1H, m), 1.67 (1H, m), 1.61 (1H, m), 1.51 (1H, m), 1.23 (1H, m).

^{13}C NMR (*Py-d$_5$*): δ 197.7 (-C=O), 186.0 (-C=O), 181.2 (-C=O), 179.5 (-C=O), 149.3 (-C), 148.3 (-C), 147.7 (-C), 147.5 (-C), 144.6 (-C), 144.0 (-C), 138.7 (-CH), 136.0 (-CH), 131.5 (-C), 131.0 (-C), 129.9 (-CH), 128.0 (-CH), 127.2 (-C), 126.9 (-C), 121.7 (-CH), 121.4 (-CH), 121.3 (-CH), 117.1 (-CH), 116.5 (-CH), 113.3 (-CH), 112.6 (-CH), 112.4 (-CH), 110.8 (-CH), 102.9 (-CH), 84.3 (-C), 76.4 (-C), 75.4 (-C), 73.7 (-CH), 65.6 (-CH), 64.5 (-CH), 56.1 (-OMe), 55.8 (-OMe), 53.8 (-CH), 52.6 (-CH), 51.5 (-CH$_2$), 51.1 (-CH), 48.6 (-CH$_2$), 31.3 (-CH$_2$), 29.4 (-CH$_2$), 28.2 (-CH$_2$), 26.8 (-CH$_2$).

MS [ESI-MS, positive mode]: found *m/z* 895 [M+H]$^+$, 917 [M+Na]$^+$.
HRMS [ESI-MS, positive mode]: MF: $C_{45}H_{43}N_4O_8I$; found m/z 895.2209 [M+H]$^+$ [calcd. 895.2204].

Compound 4Ba (±):
Color: Obtained as white solid; **Yield**: 19% (266 mg); **Mp**: 172-174 ºC; **R$_f$** 0.50 (6 % MeOH in $CHCl_3$);
^{1}H NMR (*Py-d$_5$*): δ 10.94 (1H, s, -NH), 10.91 (1H, s, -NH), 7.31 (1H, d, *J*=7.8 Hz), 7.27 (3H, m), 7.21 (1H, s), 7.18 (2H, m), 7.12 (1H, d, *J*=7.8 Hz), 6.92 (2H, m), 6.88 (1H, d, *J*=7.8 Hz), 6.77 (1H, s), 5.77 (1H, s), 4.50 (1H, d, *J*=12.0 Hz), 4.40 (1H, d, *J*=12.0 Hz), 4.31 (1H, m), 4.21 (1H, m), 3.82 (1H, m), 3.77 (3H, s), 3.74 (1H, m), 3.71 (3H, s), 2.80 (2H, m), 2.65 (1H, m), 2.60 (1H, m), 2.26 (3H, s), 2.22 (3H, s), 1.86 (4H, m), 1.78 (2H, m), 1.68 (2H, m).

^{13}C NMR (*Py-d$_5$*): δ 192.1 (-C=O), 190.3 (-C=O), 181.3 (-C=O), 181.1 (-C=O), 149.24 (-C), 149.18 (-C), 147.5 (-C), 147.4 (-C), 143.5 (-C), 139.9 (-C), 131.9 (-CH), 131.8 (-C), 131.5 (-C), 130.6 (-C), 129.7 (-CH), 127.3 (-CH), 126.6 (-C), 126.2 (-C), 125.5 (-CH), 121.6 (-CH), 121.13 (-CH), 121.07 (-CH), 119.4 (-C), 117.2 (-CH), 117.1 (-CH), 112.2 (-CH), 111.8 (-CH), 110.6 (-CH), 101.6 (-CH), 74.9 (-C), 74.5 (-C), 73.7 (-CH), 73.5 (-CH), 65.1 (-CH),

64.2 (-CH), 56.2 (-OMe), 56.1 (-OMe), 52.53 (-CH), 52.50 (-CH), 48.6 ($-CH_2$), 48.4 ($-CH_2$), 31.2 ($-CH_2$), 30.9 ($-CH_2$), 28.1 ($-CH_2$), 27.6 ($-CH_2$), 21.2 ($-CH_3$), 17.2 ($-CH_3$).

MS [ESI-MS, positive mode]: found *m/z* 797 $[M+H]^+$.
HRMS [ESI-MS, positive mode]: MF: $C_{47}H_{48}N_4O_8$; found m/z 819.3361 $[M+Na]^+$ [calcd. 819.3370].

Compound 4Bb (±):
Color: Obtained as white solid; **Yield**: 22% (308 mg); **Mp**: 188-190 ºC; R_f 0.44 (6 % MeOH in $CHCl_3$);
^{1}**H NMR (*Py-d$_5$*):** δ 11.85 (1H, s, -NH), 11.73 (1H, s, -NH), 7.25 (2H, m), 7.21 (1H, m), 7.18 (2H, d, *J*=7.8 Hz), 7.11 (2H, m), 7.03 (1H, d, *J*=7.8 Hz), 6.97 (1H, d, *J*=7.8 Hz), 6.91 (2H, m), 6.75 (1H, s), 6.00 (1H, s), 4.48 (1H, d, *J*=12.0 Hz), 4.41 (1H, d, *J*=12.0 Hz), 4.18 (2H, m), 3.87 (1H, m), 3.74 (3H, s), 3.71 (3H, s), 3.66 (1H, m), 2.80 (1H, m), 2.73 (1H, m), 2.66 (1H, m), 2.61 (1H, m), 2.31 (3H, s), 2.16 (3H, s), 1.90 (1H, m), 1.83 (3H, m), 1.71 (3H, m), 1.63 (1H, m).

^{13}**C NMR (*Py-d$_5$*):** δ 191.7 (-C=O), 190.1 (-C=O), 181.6 (-C=O), 181.2 (-C=O), 149.3 (-C), 149.2 (-C), 147.63 (-C), 147.57 (-C), 143.9 (-C), 140.0 (-C), 131.9 (-CH), 131.7 (-C), 131.5 (-C), 130.7 (-C), 129.8 (-CH), 127.1 (-CH), 126.7 (-C), 126.4 (-C), 125.3 (-CH), 121.6 (-CH), 120.9 (-CH), 120.8 (-CH), 119.4 (-C), 117.1 (2×-CH), 112.2 (-CH), 112.0 (-CH), 110.5 (-CH), 101.0 (-CH), 75.1 (-C), 74.8 (-C), 73.8 (-CH), 73.7 (-CH), 64.7 (-CH), 64.2 (-CH), 56.12 (-OMe), 56.09 (-OMe), 52.4 (-CH), 52.2 (-CH), 48.6 ($-CH_2$), 48.3 ($-CH_2$), 31.5 ($-CH_2$), 31.2 ($-CH_2$), 28.3 ($-CH_2$), 27.9 ($-CH_2$), 21.4 ($-CH_3$), 17.3 ($-CH_3$).

MS [ESI-MS, positive mode]: found *m/z* 797 $[M+H]^+$.
HRMS [ESI-MS, positive mode]: MF: $C_{47}H_{48}N_4O_8$; found m/z 819.3351 $[M+Na]^+$ [calcd. 819.3370].

Compound 4Bc (±):
Color: Obtained as white solid; **Yield**: 21% (294 mg); **Mp**: 169-171 ºC; R_f 0.40 (6 % MeOH in $CHCl_3$);
^{1}**H NMR (*DMSO-d$_6$*):** δ 10.36 (1H, s, -NH), 9.93 (1H, s, -NH), 8.84 (1H, d, *J*=4.2 Hz), 7.46 (1H, d, *J*= 7.2 Hz), 7.17 (1H, m), 7.02 (1H, s), 6.95 (1H, m), 6.74 (4H, m), 6.41 (1H, m), 6.26 (1H, s), 6.18 (1H, d, *J*= 6.6 Hz), 5.62 (1H, s), 4.24 (1H, m), 4.02 (1H, m), 3.78 (3H, s), 3.72

(2H, m), 3.56 (2H, m), 2.97 (1H, m), 2.42 (2H, m), 2.36 (1H, m), 2.25 (3H, s), 2.07 (3H, m), 1.96 (3H, s), 1.82 (2H, m), 1.68 (3H, m), 1.40 (1H, m), 1.15 (1H, m), 0.86 (1H, m).

^{13}C NMR (*DMSO-d$_6$*): δ 197.2 (-C=O), 183.6 (-C=O), 179.2 (-C=O), 178.7 (-C=O), 147.6 (-C), 146.6 (-C), 145.4 (-C), 145.1 (-C), 142.3 (-C), 139.3 (-C), 130.8 (-CH), 130.3 (-C), 129.9 (-C), 129.1 (-CH), 127.0 (-CH), 126.5 (-C), 126.3 (-C), 125.2 (-C), 124.6 (-CH), 120.7 (-CH), 119.9 (-CH), 119.7 (-CH), 118.2 (-C), 115.6 (-CH), 114.9 (-CH), 111.8 (2×-CH), 109.8 (-CH), 101.6 (-CH), 74.8 (-C), 73.6 (-C), 72.5 (-CH), 64.1 (-CH), 62.3 (-CH), 55.6 (-OMe), 54.9 (-OMe), 52.6 (-CH), 50.4 (-CH), 50.3 (-CH$_2$), 48.9 (-CH), 47.4 (-CH$_2$), 30.1 (-CH$_2$), 28.0 (-CH$_2$), 27.2 (-CH$_2$), 25.6 (-CH$_2$), 20.7 (-CH$_3$), 16.2 (-CH$_3$).

MS [ESI-MS, positive mode]: found *m/z* 797 [M+H] $^+$.
HRMS [ESI-MS, positive mode]: MF: $C_{47}H_{48}N_4O_8$; found m/z 819.3395 [M+Na] $^+$ [calcd. 819.3370].

<u>Compound 4Bd (±):</u>
Color: Obtained as white solid; **Yield**: 23% (322 mg); **Mp**: 170-172 ºC; **R$_f$** 0.39 (6 % MeOH in $CHCl_3$);
IR (KBr, ν_{max} Cm^{-1}): 3390, 2960, 1713, 1618;

^{1}H NMR (*Py-d$_5$*): δ 11.84 (1H, s, -NH), 11.17 (1H, s, -NH), 7.69 (1H, d, *J*=7.2 Hz), 7.43 (1H, s), 7.38 (1H, s), 7.31 (1H, t, *J*=7.2 Hz), 7.22 (1H, d, *J*=7.2 Hz), 7.18 (1H, d, *J*=1.2 Hz), 7.16 (1H, t, *J*=7.8 Hz), 7.06 (1H, d, *J*=7.8 Hz), 6.93 (3H, m), 6.81 (1H, s), 6.04 (1H, s), 4.99 (1H, m), 4.73 (1H, q, *J*=7.8 Hz), 4.64 (1H, d, *J*=6.6 Hz), 4.39 (1H, m), 4.25 (1H, d, *J*=12 Hz), 3.99 (1H, dd, *J*=9.6, 12 Hz), 3.75 (3H, s), 3.56 (3H, s), 3.23 (1H, m), 2.86 (1H, m), 2.79 (1H, t, *J*=7.2 Hz), 2.73 (1H, m), 2.38 (3H, s), 2.01 (3H, s), 1.97 (1H, m), 1.90 (2H, m), 1.76 (2H, m), 1.65 (1H, m), 1.59 (1H, m), 1.30 (1H, m).

^{13}C NMR (*Py-d$_5$*): δ 198.0 (-C=O), 186.2 (-C=O), 181.4 (-C=O), 180.6 (-C=O), 149.3 (-C), 148.3 (-C), 147.6 (-C), 147.3 (-C), 143.9 (-C), 141.0 (-C), 131.8 (-CH), 131.6 (2×-C), 130.9 (-C), 130.0 (-CH), 128.0 (-CH), 127.9 (-C), 126.9 (-C), 125.4 (-CH), 121.8 (-CH), 121.7 (-CH), 121.5 (-CH), 119.4 (-C), 117.1 (-CH), 116.4 (-CH), 113.2 (-CH), 112.4 (-CH), 110.9 (-CH), 102.9 (-CH), 76.5 (-C), 75.4 (-C), 73.8 (-CH), 65.5 (-CH), 64.4 (-CH), 56.2 (-OMe), 55.7 (-OMe), 54.3 (-CH), 52.6 (-CH), 51.5 (-CH$_2$), 50.9 (-CH), 48.6 (-CH$_2$), 31.3 (-CH$_2$), 29.4 (-CH$_2$), 28.2 (-CH$_2$), 26.8 (-CH$_2$), 21.4 (-CH$_3$), 17.1 (-CH$_3$).

MS [ESI-MS, positive mode]: found *m/z* 797 [M+H] $^+$.

HRMS [ESI-MS, positive mode]: MF: $C_{47}H_{48}N_4O_8$; found m/z 819.3378 $[M+Na]^+$ [calcd. 819.3370].

Compound 4Ca (±):

Color: Obtained as white solid; **Yield**: 17% (230 mg); **Mp**: 183-185 ℃; $\mathbf{R_f}$ 0.48 (6 % MeOH in $CHCl_3$);

IR (KBr, ν_{max} Cm^{-1}): 3391, 2961, 2868, 1722, 1615;

^{1}H NMR (*Py-d$_5$*): δ 11.28 (1H, s, -NH), 10.88 (1H, s, -NH), 7.59 (1H, dd, *J*=4.8, 6.6 Hz), 7.34 (1H, s), 7.24 (2H, m), 7.22 (1H, m), 7.18 (2H, m), 7.00 (3H, m), 6.85 (2H, m), 5.70 (1H, s), 4.52 (1H, d, *J*=12.0 Hz), 4.37 (1H, d, *J*=12.0 Hz), 4.29 (1H, m), 4.20 (1H, m), 3.81 (2H, m), 3.73 (3H, s), 3.70 (3H, s), 2.83 (1H, m), 2.77 (1H, m), 2.62 (2H, m), 2.16 (3H, s), 1.85 (4H, m), 1.78 (2H, m), 1.69 (2H, m).

^{13}C NMR (*Py-d$_5$*): δ 191.2 (-C=O), 190.9 (-C=O), 181.0 (-C=O), 180.8 (-C=O), 149.22 (-C), 149.16 (-C), 147.5 (-C), 147.4 (-C), 142.4 (-C), 141.2 (-C), 131.6 (-C), 131.5 (-C), 130.8 (-C), 130.2 (-CH), 129.7 (-CH), 128.6 (-C), 128.0 (-CH), 127.5 (-CH), 126.7 (-C), 126.6 (-C), 120.8 (2×-CH), 117.1 (2×-CH), 112.2 (-CH), 111.8 (-CH), 111.7 (-CH), 110.4 (-CH), 101.7 (-CH), 74.7 (-C), 74.5 (-C), 73.7 (-CH), 73.2 (-CH), 65.1 (-CH), 64.0 (-CH), 56.1 (-OMe), 56.0 (-OMe), 52.2 (2×-CH), 48.54 (-CH_2), 48.51 (-CH_2), 31.0 (-CH_2), 30.6 (-CH_2), 27.9 (-CH_2), 27.7 (-CH_2), 21.2 (-CH_3).

MS [ESI-MS, positive mode]: found *m/z* 817 $[M+H]^+$.

HRMS [ESI-MS, positive mode]: MF: $C_{46}H_{45}N_4O_8Cl$; found m/z 839.2817 $[M+Na]^+$ [calcd. 839.2824].

Compound 4Cb (±):

Color: Obtained as white solid; **Yield**: 20% (271 mg); **Mp**: 208-210 ℃; $\mathbf{R_f}$ 0.43 (6 % MeOH in $CHCl_3$);

IR (KBr, ν_{max} Cm^{-1}): 3382, 1711, 1613, 1516;

^{1}H NMR (*Py-d$_5$*): δ 12.14 (1H, s, -NH), 11.77 (1H, s, -NH), 7.59 (1H, m), 7.35 (1H, s), 7.20 (1H, m), 7.15 (2H, d, *J*=8.4 Hz), 7.10 (1H, d, *J*=1.8 Hz), 7.05 (1H, d, *J*=1.2 Hz), 6.94 (1H, d, *J*=7.8 Hz), 6.86 (1H, d, *J*=7.8 Hz), 6.84 (1H, d, *J*=1.2 Hz), 6.82 (1H, d, *J*=7.8 Hz), 6.80 (1H, dd, *J*=1.2, 8.4 Hz), 5.92 (1H, s), 4.39 (2H, d, *J*=12.0 Hz), 4.20 (1H, m), 4.12 (1H, m), 3.92 (1H, m), 3.82 (1H, m), 3.66 (3H, s), 3.65 (3H, s), 2.82 (1H, m), 2.65 (3H, m), 2.11 (3H, s), 1.85 (5H, m), 1.71 (3H, m).

^{13}C NMR (*Py-d$_5$*): δ 191.2 (-C=O), 189.8 (-C=O), 181.2 (-C=O), 180.9 (-C=O), 149.0 (2×-C), 147.42 (-C), 147.36 (-C), 142.6 (-C), 141.2 (-C), 131.6 (-C), 131.2 (-C), 130.7 (-C), 130.3 (-CH), 129.9 (-CH), 128.7 (-C), 128.2 (-CH), 127.5 (-CH), 126.7 (-C), 126.6 (-C), 120.35 (-CH), 120.31 (-CH), 116.99 (-CH), 116.97 (-CH), 112.5 (-CH), 112.3 (-CH), 111.7 (-CH), 110.3 (-CH), 101.5 (-CH), 75.25 (-C), 75.22 (-C), 73.7 (-CH), 73.4 (-CH), 65.3 (-CH), 64.9 (-CH), 56.0 (2×-OMe), 51.8 (-CH), 51.6 (-CH), 48.3 (-CH_2), 48.2 (-CH_2), 31.4 (2×-CH_2), 28.4 (-CH_2), 28.3 (-CH_2), 21.3 (-CH_3).

MS [ESI-MS, positive mode]: found *m/z* 817 [M+H]$^+$, 839 [M+Na]$^+$.
HRMS [ESI-MS, positive mode]: MF: $C_{46}H_{45}N_4O_8Cl$; found m/z 839.2820 [M+Na]$^+$ [calcd. 839.2824].

Compound 4Cc (±):

Color: Obtained as white solid; **Yield**: 20% (271 mg); **Mp**: 174-176 ºC; **R_f** 0.36 (6 % MeOH in $CHCl_3$);
^{1}H NMR (*Py-d$_5$*): δ 12.14 (1H, s, -NH), 11.20 (1H, s, -NH), 7.90 (1H, d, *J*=1.8 Hz), 7.53 (1H, s), 7.39 (1H, dd, *J*=1.8, 7.8 Hz), 7.30 (1H, m), 7.17 (1H, d, *J*=7.8 Hz), 7.07 (1H, d, *J*=8.4 Hz), 7.04 (1H, d, *J*=8.4 Hz), 7.01 (1H, d, *J*=8.4 Hz), 6.91 (1H, m), 6.86 (2H, m), 6.67 (1H, d, *J*=7.8 Hz), 6.21 (1H, s), 5.04 (1H, m), 4.86 (1H, m), 4.66 (1H, d, *J*=12.6 Hz), 4.31 (1H, m), 4.25 (1H, d, *J*=12.0 Hz), 4.01 (1H, m), 3.68 (3H, s), 3.51 (3H, s), 3.34 (1H, m), 2.88 (1H, m), 2.82 (1H, m), 2.74 (1H, m), 2.34 (3H, s), 1.94 (3H, m), 1.88 (1H, m), 1.78 (4H, m).

^{13}C NMR (*Py-d$_5$*): δ 197.5 (-C=O), 187.4 (-C=O), 181.0 (-C=O), 180.2 (-C=O), 149.3 (-C), 148.2 (-C), 147.7 (-C), 147.4 (-C), 143.0 (-C), 142.4 (-C), 131.1 (-C), 131.0 (-C), 130.2 (-CH), 130.0 (-CH), 129.2 (-C), 128.4 (-C), 128.1 (-CH), 127.7 (-CH), 127.3 (-C), 127.0 (-C), 121.6 (-CH), 121.2 (-CH), 117.2 (-CH), 116.4 (-CH), 112.9 (-CH), 112.4 (-CH), 112.0 (-CH), 110.2 (-CH), 103.0 (-CH), 76.6 (-C), 75.2 (-C), 74.0 (-CH), 65.9 (-CH), 64.6 (-CH), 56.1 (-OMe), 55.7 (-OMe), 53.9 (-CH), 52.4 (-CH), 52.1 (-CH), 51.5 (-CH_2), 48.5 (-CH_2), 31.3 (-CH_2), 29.1 (-CH_2), 28.4 (-CH_2), 27.0 (-CH_2), 21.5 (-CH_3).

MS [ESI-MS, positive mode]: found *m/z* 817 [M+H]$^+$, 839 [M+Na]$^+$.
HRMS [ESI-MS, positive mode]: MF: $C_{46}H_{45}N_4O_8Cl$; found m/z 839.2839 [M+Na]$^+$ [calcd. 839.2824].

Compound 4Cd (±):

Color: Obtained as white solid; **Yield**: 23% (311 mg); **Mp**: 172-174 ºC; $\mathbf{R_f}$ 0.35 (6 % MeOH in $CHCl_3$);

^{1}H NMR (*Py-d$_5$*): δ 12.20 (1H, s, -NH), 11.24 (1H, s, -NH), 7.91 (1H, s), 7.56 (1H, m), 7.39 (1H, m), 7.31 (1H, s), 7.17 (1H, m), 7.07 (3H, m), 7.00 (1H, d, *J*=7.2 Hz), 6.92 (1H, m), 6.86 (1H, m), 6.67 (1H, d, *J*=7.8 Hz), 6.21 (1H, s), 4.87 (1H, m), 4.67 (1H, d, *J*=12.6 Hz), 4.30 (2H, m), 4.03 (1H, m), 3.71 (1H, m), 3.68 (3H, s), 3.51 (3H, s), 2.87 (3H, m), 2.34 (3H, s), 2.23 (1H, s), 1.92 (5H, m), 1.78 (3H, m).

^{13}C NMR (*Py-d$_5$*): δ 198.4 (-C=O), 185.7 (-C=O), 181.0 (-C=O), 180.1 (-C=O), 149.2 (-C), 148.2 (-C), 147.7 (-C), 147.4 (-C), 142.9 (-C), 142.4 (-C), 131.3 (-C), 130.9 (-C), 130.1 (-CH), 129.9 (-CH), 129.1 (-C), 128.4 (-C), 128.1 (-CH), 128.0 (-CH), 127.6 (-C), 126.8 (-C), 121.4 (-CH), 121.3 (-CH), 117.1 (-CH), 116.4 (-CH), 113.2 (-CH), 112.4 (-CH), 111.9 (-CH), 110.1 (-CH), 103.0 (-CH), 76.5 (-C), 75.3 (-C), 73.7 (-CH), 65.5 (-CH), 64.7 (-CH), 56.0 (-OMe), 55.7 (-OMe), 54.0 (-CH), 52.4 (-CH), 51.5 (-CH_2), 50.9 (-CH), 48.7 (-CH_2), 31.0 (-CH_2), 29.9 (-CH_2), 28.1 (-CH_2), 23.2 (-CH_2), 21.5 (-CH_3).

MS [ESI-MS, positive mode]: found *m/z* 817 $[M+H]^+$, 839 $[M+Na]^+$.
HRMS [ESI-MS, positive mode]: MF: $C_{46}H_{45}N_4O_8Cl$; found m/z 839.2812 $[M+Na]^+$ [calcd. 839.2824].

<u>**Compound 4Da (±):**</u>

Color: Obtained as white solid; **Yield**: 18% (246 mg); **Mp**: 238-240 ºC; $\mathbf{R_f}$ 0.47 (6 % MeOH in $CHCl_3$);
IR (KBr, ν_{max} Cm^{-1}): 3399, 2959, 2868, 1719, 1605;

^{1}H NMR (*Py-d$_5$*): δ 11.19 (1H, s, -NH), 10.89 (1H, s, -NH), 7.30 (1H, dd, *J*=2.4 Hz), 7.28 (1H, d, *J*=8.4 Hz), 7.25 (1H, d, *J*=7.8 Hz), 7.22 (1H, d, *J*=1.8 Hz), 7.20 (1H, d, *J*=1.2 Hz), 7.14 (1H, d, *J*=1.8 Hz), 7.08 (1H, dd, *J*=1.8, 8.4 Hz), 7.00 (1H, td, *J*=2.4, 8.4 Hz), 6.89 (3H, m), 6.85 (1H, q, *J*=4.2), 5.75 (1H, s), 4.49 (2H, m), 4.20 (2H, m), 3.75 (3H, s), 3.72 (1H, m), 3.70 (3H, s), 3.69 (3H, s), 3.68 (1H, m), 2.82 (1H, m), 2.73 (1H, m), 2.63 (2H, m), 1.88 (2H, m), 1.78 (4H, m), 1.69 (2H, m).

^{13}C NMR (*Py-d$_5$*): δ 191.15 (-C=O), 191.11 (-C=O), 180.9 (-C=O), 180.8 (-C=O), 158.7 (-C, $^1J_{C-F}$=237.0 Hz), 155.4 (-C), 149.3 (-C), 149.2 (-C), 147.6 (-C), 147.5 (-C), 139.8 (-C), 137.1 (-C), 131.5 (-C), 131.3 (-C), 128.6 (-C), 127.9 (-C), 121.2 (-CH), 121.0 (-CH), 117.2 (-CH), 117.1 (-CH), 116.1 (-CH, $^2J_{C-F}$=24.0 Hz), 115.3 (-CH), 114.8 (-CH, $^2J_{C-F}$=24.0 Hz), 114.3 (-

CH), 111.9 (-CH), 111.5 (-CH), 111.1 (-CH, ${}^{3}J_{C\text{-}F}$=9.0 Hz), 110.9 (-CH), 101.6 (-CH), 74.9 (-C), 74.8 (-C), 73.8 (-CH), 73.5 (-CH), 64.6 (-CH), 64.5 (-CH), 56.2 (-OMe), 56.1 (-OMe), 56.0 (-OMe), 52.6 (-CH), 52.4 (-CH), 48.4 (2×-CH_2), 31.1 (-CH_2), 30.9 (-CH_2), 28.0 (-CH_2), 27.9 (-CH_2).

MS [ESI-MS, positive mode]: found *m/z* 817 $[M+H]^+$, 839 $[M+Na]^+$.
HRMS [ESI-MS, positive mode]: MF: $C_{46}H_{45}N_4O_9F$; found m/z 839.3084 $[M+Na]^+$ [calcd. 839.3068].

Compound 4Db (±):
Color: Obtained as white solid; **Yield**: 22% (300 mg); **Mp**: 178-180 ºC; $\mathbf{R_f}$ 0.43 (6 % MeOH in $CHCl_3$);
IR (KBr, ν_{max} Cm^{-1}): 3385, 2961, 2870, 1721, 1605;

^{1}H NMR (*Py-d$_5$*): δ 11.91 (1H, s, -NH), 11.74 (1H, s, -NH), 7.33 (1H, dd, *J*=2.4, 8.4 Hz), 7.18 (2H, m), 7.14 (1H, d, *J*=7.8 Hz), 7.11 (2H, dd, *J*=1.8, 7.8 Hz), 7.01 (1H, td, *J*=2.4, 9.0 Hz), 6.96 (1H, d, *J*=8.4 Hz), 6.87 (3H, m), 6.83 (1H, dd, *J*=2.4, 8.4 Hz), 5.96 (1H, s), 4.44 (2H, m), 4.13 (2H, m), 3.84 (1H, m), 3.76 (1H, m), 3.73 (3H, s), 3.71 (3H, s), 3.63 (3H, s), 2.76 (1H, m), 2.65 (3H, m), 1.86 (2H, m), 1.78 (2H, m), 1.69 (4H, m).

^{13}C NMR (*Py-d$_5$*): δ 191.4 (-C=O), 189.5 (-C=O), 181.14 (-C=O), 181.08 (-C=O), 158.7 (-C, ${}^{1}J_{C\text{-}F}$=237 Hz), 155.4 (-C), 149.1 (2×-C), 147.42 (-C), 147.40 (-C), 139.9 (-C), 137.1 (-C), 131.5 (-C), 131.2 (-C), 128.4 (-C, ${}^{3}J_{C\text{-}F}$=7.5 Hz), 128.0 (-C), 120.7 (-CH), 120.6 (-CH), 117.1 (-CH), 117.0 (-CH), 116.2 (-CH, ${}^{2}J_{C\text{-}F}$=24.0 Hz), 115.4 (-CH), 114.9 (-CH, ${}^{2}J_{C\text{-}F}$=25.5 Hz), 114.0 (-CH), 112.2 (-CH), 112.1 (-CH), 111.2 (-CH, ${}^{3}J_{C\text{-}F}$=9 Hz), 110.9 (-CH), 101.5 (-CH), 75.4 (-C), 75.3 (-C), 73.9 (-CH), 73.6 (-CH), 65.0 (-CH), 64.5 (-CH), 56.08 (-OMe), 56.06 (-OMe), 55.9 (-OMe), 51.9 (-CH), 51.8 (-CH), 48.2 (2×-CH_2), 31.6 (-CH_2), 31.5 (-CH_2), 28.41 (-CH_2), 28.40 (-CH_2).

MS [ESI-MS, positive mode]: found *m/z* 817 $[M+H]^+$, 839 $[M+Na]^+$.
HRMS [ESI-MS, positive mode]: MF: $C_{46}H_{45}N_4O_9F$; found m/z 839.3062 $[M+Na]^+$ [calcd. 839.3068].

Compound 4Dc (±):
Color: Obtained as white solid; **Yield**: 21% (286 mg); **Mp**: 196-198 ºC; $\mathbf{R_f}$ 0.37 (6 % MeOH in $CHCl_3$);

^{1}H NMR (*Py-d$_5$*): δ 11.81 (1H, s, -NH), 11.39 (1H, s, -NH), 7.66 (1H, dd, *J*=2.4, 8.4 Hz), 7.40 (1H, d, *J*=1.8 Hz), 7.34 (1H, s), 7.15 (1H, d, *J*=7.8 Hz), 7.09 (1H, d, *J*=9.0 Hz), 7.03 (1H, m), 7.00 (1H, dd, *J*=2.4, 8.4 Hz), 6.92 (3H, m), 6.86 (1H, d, *J*=7.8 Hz), 6.64 (1H, q, *J*=4.2 Hz), 6.11 (1H, s), 4.97 (1H, m), 4.77 (1H, m), 4.66 (1H, d, *J*=12.6 Hz), 4.31 (1H, m), 4.19 (1H, d, *J*=12.0 Hz), 3.97 (1H, m), 3.71 (3H, s), 3.68 (3H, s), 3.53 (3H, s), 3.25 (1H, m), 2.90 (1H, m), 2.83 (1H, m), 2.74 (1H, m), 1.93 (1H, m), 1.86 (3H, m), 1.72 (4H, m).

^{13}C NMR (*Py-d$_5$*): δ 195.7 (-C=O), 189.0 (-C=O), 181.2 (-C=O), 180.1 (-C=O), 158.9 (-C, $^1J_{C-F}$=235.5 Hz), 155.6 (-C), 149.2 (-C), 148.3 (-C), 147.64 (-C), 147.56 (-C), 140.9 (-C), 137.5 (-C), 131.5 (-C), 129.9 (-C, $^3J_{C-F}$=9.4 Hz), 128.4 (-C), 126.9 (-C), 121.8 (-CH), 121.2 (-CH), 117.1 (-CH), 116.5 (-CH), 116.1 (-CH, $^2J_{C-F}$=22.5 Hz), 115.7 (-CH), 115.2 (-CH, $^2J_{C-F}$=24.0 Hz), 113.8 (-CH), 112.7 (-CH), 112.4 (-CH), 110.9 (-CH, $^3J_{C-F}$=7.5 Hz), 110.8 (-CH), 102.7 (-CH), 76.8 (-C), 75.4 (-C), 73.9 (-CH), 65.8 (-CH), 64.7 (-CH), 56.1 (-OMe), 56.0 (-OMe), 55.7 (-OMe), 53.4 (-CH), 52.5 (-CH), 52.1 (-CH), 51.5 (-CH_2), 48.5 (-CH_2), 31.4 (-CH_2), 28.9 (-CH_2), 28.3 (-CH_2), 26.9 (-CH_2).

MS [ESI-MS, positive mode]: found *m/z* 817 [M+H]$^+$, 839 [M+Na]$^+$.
HRMS [ESI-MS, positive mode]: MF: $C_{46}H_{45}N_4O_9F$; found m/z 839.3059 [M+Na]$^+$ [calcd. 839.3068].

Compound 4Dd (±):
Color: Obtained as white solid; **Yield**: 24% (327 mg); **Mp**: 189-191 ºC; **R$_f$** 0.36 (6 % MeOH in $CHCl_3$);
^{1}H NMR (*Py-d$_5$*): δ 11.66 (1H, s, -NH), 11.37 (1H, s, -NH), 7.72 (1H, d, *J*=8.4 Hz), 7.45 (1H, s), 7.37 (1H, s), 7.18 (1H, d, *J*=8.4 Hz), 7.10 (1H, d, *J*=7.8 Hz), 7.02 (2H, m), 6.95 (3H, m), 6.92 (1H, m), 6.66 (1H, q, *J*=3.6 Hz), 5.97 (1H, s), 4.93 (1H, t, *J*=10.2 Hz), 4.71 (1H, m), 4.60 (1H, d, *J*=11.4 Hz), 4.41 (1H, m), 4.25 (1H, d, *J*=12.0 Hz), 4.01 (1H, m), 3.80 (3H, s), 3.69 (3H, s), 3.52 (3H, s), 3.20 (1H, m), 2.97 (1H, m), 2.78 (2H, m), 1.91 (4H, m), 1.77 (2H, m), 1.65 (1H, m), 1.60 (1H, m).

^{13}C NMR (*Py-d$_5$*): δ 194.0 (-C=O), 187.2 (-C=O), 181.3 (-C=O), 180.1 (-C=O), 158.9 (-C, $^1J_{C-F}$=235.5 Hz), 155.6 (-C), 149.3 (-C), 148.3 (-C), 147.6 (-C), 147.5 (-C), 140.9 (-C), 137.5 (-C), 131.7 (-C), 130.0 (-C), 128.5 (-C), 127.4 (-C), 121.8 (-CH), 121.6 (-CH), 117.0 (-CH), 116.4 (-CH), 116.0 (-CH, $^2J_{C-F}$=24.0 Hz), 115.8 (-CH), 115.3 (-CH, $^2J_{C-F}$=24.0 Hz), 114.3 (-CH), 113.1 (-CH), 112.3 (-CH), 110.9 (-CH), 110.8 (-CH), 102.8 (-CH), 76.8 (-C), 75.7 (-C), 73.6 (-CH), 65.8 (-CH), 65.0 (-CH), 56.3 (-OMe), 56.1 (-OMe), 55.8 (-OMe), 53.9 (-CH),

52.7 (-CH), 51.45 (-CH), 51.41 (-CH_2), 48.6 (-CH_2), 31.2 (-CH_2), 29.3 (-CH_2), 28.1 (-CH_2), 26.8 (-CH_2).

MS [ESI-MS, positive mode]: found *m/z* 817 $[M+H]^+$, 839 $[M+Na]^+$.
HRMS [ESI-MS, positive mode]: MF: $C_{46}H_{45}N_4O_9F$; found m/z 839.3059 $[M+Na]^+$ [calcd. 839.3068].

<u>**Compound 4Ea (±):**</u>
Color: Obtained as white solid; **Yield**: 18% (250 mg); **Mp**: 180-182 ºC; $\mathbf{R_f}$ 0.47 (6 % MeOH in $CHCl_3$);
IR (KBr, ν_{max} Cm^{-1}): 3397, 2959, 2868, 1720, 1615;

^{1}H NMR (*Py-d$_5$*): δ 11.02 (1H, s, -NH), 10.96 (1H, s, -NH), 7.32 (1H, dd, *J*=2.4, 8.4 Hz), 7.28 (2H, m), 7.23 (1H, d, *J*=1.8 Hz), 7.21 (1H, m), 7.17 (1H, d, *J*=1.2 Hz), 7.09 (1H, dd, *J*=1.2, 7.8 Hz), 6.97 (2H, m), 6.81 (1H, m), 6.77 (1H, s), 5.74 (1H, s), 4.52 (1H, d, *J*=12.6 Hz), 4.38 (1H, d, *J*=12.0 Hz), 4.33 (1H, m), 4.17 (1H, m), 3.79 (2H, m), 3.75 (3H, s), 3.70 (3H, s), 2.82 (1H, m), 2.76 (1H, m), 2.63 (2H, m), 2.24 (3H, s), 2.20 (3H, s), 1.82 (6H, m), 1.69 (2H, m).

^{13}C NMR (*Py-d$_5$*): δ 192.4 (-C=O), 190.2 (-C=O), 181.2 (-C=O), 181.0 (-C=O), 158.7 (-C, $^1J_{C-F}$=235.5 Hz), 149.22 (-C), 149.17 (-C), 147.5 (-C), 147.4 (-C), 139.8 (-C), 139.6 (-C), 131.9 (-CH), 131.7 (-C), 131.5 (-C), 130.6 (-C), 128.3 (-C), 126.1 (-C), 125.4 (-CH), 120.91 (-CH), 120.87 (-CH), 119.3 (-C), 117.2 (-CH), 117.1 (-CH), 116.1 (-CH, $^2J_{C-F}$=22.5 Hz), 115.1 (-CH, $^2J_{C-F}$=24.0 Hz), 112.4 (-CH), 111.6 (-CH), 111.1 (-CH), 101.6 (-CH), 74.9 (-C), 74.8 (-C), 73.8 (-CH), 73.3 (-CH), 65.6 (-CH), 63.6 (-CH), 56.1 (-OMe), 56.0 (-OMe), 52.4 (-CH), 52.3 (-CH), 48.5 (-CH_2), 48.4 (-CH_2), 31.1 (-CH_2), 30.7 (-CH_2), 28.0 (-CH_2), 27.7 (-CH_2), 21.2 (-CH_3), 17.1 (-CH_3).

MS [ESI-MS, positive mode]: found *m/z* 815 $[M+H]^+$, 837 $[M+Na]^+$.
HRMS [ESI-MS, positive mode]: MF: $C_{47}H_{47}N_4O_8F$; found m/z 837.3240 $[M+Na]^+$ [calcd. 837.3276].

<u>**Compound 4Eb (±):**</u>
Color: Obtained as white solid; **Yield**: 22% (305 mg); **Mp**: 182-184 ºC; $\mathbf{R_f}$ 0.43 (6 % MeOH in $CHCl_3$);
IR (KBr, ν_{max} Cm^{-1}): 3403, 3208, 2964, 2871, 1713, 1605;

^{1}H NMR (*Py-d$_5$*): δ 12.03 (1H, s, -NH), 11.73 (1H, s, -NH), 7.28 (1H, d, *J*=7.8 Hz), 7.17 (2H, m), 7.13 (2H, m), 7.08 (1H, s), 6.98 (1H, m), 6.82 (3H, m), 6.69 (1H, s), 5.93 (1H, s), 4.38 (2H, m), 4.20 (1H, m), 4.13 (1H, m), 3.92 (1H, m), 3.78 (1H, m), 3.67 (3H, s), 3.64 (3H, s), 2.84 (1H, m), 2.68 (3H, m), 2.28 (3H, s), 2.12 (3H, s), 1.92 (1H, m), 1.84 (4H, m), 1.72 (3H, m).

^{13}C NMR (*Py-d$_5$*): δ 190.7 (-C=O), 190.3 (-C=O), 181.5 (-C=O), 181.3 (-C=O), 158.6 (-C, $^1J_{C-F}$=237.0 Hz), 149.0 (2×-C), 147.44 (-C), 147.38 (-C), 139.9 (2×-C), 131.9 (-CH), 131.6 (-C), 131.3 (-C), 130.6 (-C), 128.3 (-C), 126.2 (-C), 125.5 (-CH), 120.3 (-CH), 120.2 (-CH), 119.4 (-C), 117.0 (-CH), 116.9 (-CH), 116.2 (-CH, $^2J_{C-F}$=24.0 Hz), 115.1 (-CH, $^2J_{C-F}$=24.0 Hz), 112.6 (-CH), 112.3 (-CH), 111.0 (-CH, $^3J_{C-F}$=7.5 Hz), 101.4 (-CH), 75.4 (2×-C), 73.6 (-CH), 73.4 (-CH), 65.2 (-CH), 64.9 (-CH), 56.04 (-OMe), 55.98 (-OMe), 51.8 (-CH), 51.5 (-CH), 48.33 (-CH_2), 48.29 (-CH_2), 31.5 (-CH_2), 31.3 (-CH_2), 28.4 (-CH_2), 28.2 (-CH_2), 21.3 (-CH_3), 17.2 (-CH_3).

MS [ESI-MS, positive mode]: found *m/z* 815 [M+H]$^+$, 837 [M+Na]$^+$.
HRMS [ESI-MS, positive mode]: MF: $C_{47}H_{47}N_4O_8F$; found m/z 837.3305 [M+Na]$^+$ [calcd. 837.3276].

<u>Compound 4Ec (±):</u>
Color: Obtained as white solid; **Yield**: 21% (292 mg); **Mp**: 172-174 ºC; **R_f** 0.38 (6 % MeOH in $CHCl_3$);
^{1}H NMR (*Py-d$_5$*): δ 12.01 (1H, s, -NH), 11.15 (1H, s, -NH), 7.66 (1H, dd, *J*=2.4, 8.4 Hz), 7.37 (1H, s), 7.31 (1H, d, *J*=1.8 Hz), 7.17 (1H, m), 7.13 (2H, m), 7.01 (1H, m), 6.92 (2H, m), 6.85 (1H, m), 6.79 (1H, s), 6.15 (1H, s), 4.99 (1H, dd, *J*=9.6, 12.0 Hz), 4.80 (1H, q, *J*=8.4 Hz), 4.66 (1H, d, J=12.0 Hz), 4.31 (1H, m), 4.23 (1H, d, *J*=12.0 Hz), 4.00 (1H, dd, *J*=9.6, 12.0 Hz), 3.70 (3H, s), 3.52 (3H, s), 3.21 (1H, m), 2.83 (2H, m), 2.74 (1H, m), 2.33 (3H, s), 1.99 (3H, s), 1.91 (3H, m), 1.83 (2H, m), 1.76 (2H, m), 1.69 (1H, m).

^{13}C NMR (*Py-d$_5$*): δ 196.8 (-C=O), 188.2 (-C=O), 181.3 (-C=O), 180.4 (-C=O), 158.9 (-C, $^1J_{C-F}$=237.0 Hz), 149.3 (-C), 148.2 (-C), 147.7 (-C), 147.4 (-C), 141.0 (-C), 140.2 (-C), 131.8 (-CH), 131.2 (-C), 130.8 (-C), 128.8 (-C, $^3J_{C-F}$=7.5 Hz), 127.8 (-C), 127.5 (-C), 125.3 (-CH), 121.7 (-CH), 121.2 (-CH), 119.3 (-C), 117.1 (-CH), 116.4 (-CH), 116.3 (-CH, $^2J_{C-F}$=24.0 Hz), 115.4 (-CH, $^2J_{C-F}$=24.0 Hz), 113.0 (-CH), 112.3 (-CH), 111.2 (-CH, $^3J_{C-F}$=7.5 Hz), 102.8 (-CH), 76.6 (-C), 75.4 (-C), 73.9 (-CH), 65.6 (-CH), 64.7 (-CH), 56.1 (-OMe), 55.6 (-OMe),

54.0 (-CH), 52.3 (-CH), 51.7 (-CH), 51.5 (-CH_2), 48.4 (-CH_2), 31.3 (-CH_2), 29.0 (-CH_2), 28.3 (-CH_2), 26.9 (-CH_2), 21.4 (-CH_3), 17.0 (-CH_3).

MS [ESI-MS, positive mode]: found *m/z* 815 $[M+H]^+$, 837 $[M+Na]^+$.
HRMS [ESI-MS, positive mode]: MF: $C_{47}H_{47}N_4O_8F$; found m/z 837.3284 $[M+Na]^+$ [calcd. 837.3276].

Compound 4Ed (±):
Color: Obtained as white solid; **Yield**: 23% (319 mg); **Mp**: 170-172 ºC; $\mathbf{R_f}$ 0.37 (6 % MeOH in $CHCl_3$);
^{1}H NMR (*Py-d₅*): δ 11.87 (1H, s, -NH), 11.14 (1H, s, -NH), 7.74 (1H, dd, *J*=2.4, 8.4 Hz), 7.40 (1H, s), 7.31 (1H, d, *J*=1.8 Hz), 7.27 (1H, m), 7.18 (1H, m), 7.12 (2H, m), 6.95 (2H, m), 6.91 (1H, d, *J*=8.4 Hz), 6.80 (1H, s), 6.04 (1H, s), 5.05 (1H, dd, *J*=9.6, 12.0 Hz), 4.68 (1H, m), 4.61 (1H, d, *J*=12.0 Hz), 4.37 (1H, m), 4.25 (1H, d, *J*=12.0 Hz), 4.00 (1H, m), 3.66 (3H, s), 3.54 (3H, s), 3.29 (1H, m), 2.77 (2H, m), 2.38 (3H, s), 1.99 (3H, s), 1.93 (2H, m), 1.76 (3H, m), 1.64 (2H, m), 1.55 (2H, m).

^{13}C NMR (*Py-d₅*): δ 198.0 (-C=O), 186.2 (-C=O), 181.2 (-C=O), 180.4 (-C=O), 158.9 (-C, $^1J_{C-F}$=237.0 Hz), 149.3 (-C), 148.3 (-C), 147.7 (-C), 147.4 (-C), 141.0 (-C), 140.1 (-C), 131.8 (-CH), 131.2 (-C), 130.8 (-C), 128.8 (-C, $^3J_{C-F}$=7.5 Hz), 127.8 (-C), 127.3 (-C), 125.4 (-CH), 121.7 (-CH), 121.3 (-CH), 119.3 (-C), 117.14 (-CH), 117.08 (-CH), 116.3 (-CH, $^2J_{C-F}$=24.0 Hz), 115.6 (-CH, $^2J_{C-F}$=24.0 Hz), 113.1 (-CH), 112.3 (-CH), 111.3 (-CH, $^3J_{C-F}$=7.5 Hz), 102.9 (-CH), 76.5 (-C), 75.7 (-C), 73.7 (-CH), 65.5 (-CH), 64.7 (-CH), 56.1 (-OMe), 55.7 (-OMe), 54.2 (-CH), 52.4 (-CH), 51.5 (-CH_2), 50.8 (-CH), 48.5 (-CH_2), 31.2 (-CH_2), 29.9 (-CH_2), 28.3 (-CH_2), 26.8 (-CH_2), 21.4 (-CH_3), 17.0 (-CH_3).

MS [ESI-MS, positive mode]: found *m/z* 815 $[M+H]^+$, 837 $[M+Na]^+$.
HRMS [ESI-MS, positive mode]: MF: $C_{47}H_{47}N_4O_8F$; found m/z 837.3264 $[M+Na]^+$ [calcd. 837.3276].

Compound 4Fa (±):
Color: Obtained as white solid; **Yield**: 17% (232 mg); **Mp**: 182-184 ºC; $\mathbf{R_f}$ 0.47 (6 % MeOH in $CHCl_3$);
IR (KBr, ν_{max} Cm^{-1}): 3385, 2959, 2868, 1719, 1607;

^{1}H NMR (*Py-d₅*): δ 10.96 (2H, s, -NH), 7.32 (1H, dd, *J*=2.4, 8.4 Hz), 7.27 (2H, m), 7.23 (1H, d, *J*=1.8 Hz), 7.22 (1H, m), 7.16 (1H, d, *J*=1.8 Hz), 7.09 (1H, dd, *J*=1.8, 7.8 Hz), 6.96 (2H,

m), 6.80 (1H, m), 6.77 (1H, s), 5.74 (1H, s), 4.52 (1H, d, *J*=12.6 Hz), 4.38 (1H, d, *J*=12.0 Hz), 4.33 (1H, m), 4.16 (1H, m), 3.79 (2H, m), 3.75 (3H, s), 3.70 (3H, s), 2.80 (1H, m), 2.76 (1H, m), 2.62 (2H, m), 2.24 (3H, s), 2.20 (3H, s), 1.82 (6H, m), 1.69 (2H, m).

^{13}C NMR (*Py-d$_5$*): δ 192.3 (-C=O), 190.2 (-C=O), 181.2 (-C=O), 181.0 (-C=O), 158.7 (-C, $^{1}J_{C\text{-}F}$=240.0 Hz), 149.21 (-C), 149.16 (-C), 147.5 (-C), 147.4 (-C), 139.8 (-C), 139.6 (-C), 131.9 (-CH), 131.6 (-C), 131.5 (-C), 130.6 (-C), 128.3 (-C, $^{3}J_{C\text{-}F}$=7.5 Hz), 126.1 (-C), 125.4 (-CH), 120.91 (-CH), 120.87 (-CH), 119.3 (-C), 117.2 (-CH), 117.1 (-CH), 116.1 (-CH, $^{2}J_{C\text{-}F}$=22.5 Hz), 115.1 (-CH, $^{2}J_{C\text{-}F}$=24.0 Hz), 112.4 (-CH), 111.6 (-CH), 111.1 (-CH), 101.7 (-CH), 74.9 (-C), 74.8 (-C), 73.8 (-CH), 73.3 (-CH), 65.6 (-CH), 63.8 (-CH), 56.1 (-OMe), 56.0 (-OMe), 52.4 (-CH), 52.3 (-CH), 48.5 (-CH_2), 48.4 (-CH_2), 31.1 (-CH_2), 30.7 (-CH_2), 28.0 (-CH_2), 27.7 (-CH_2), 21.2 (-CH_3), 17.1 (-CH_3).

MS [ESI-MS, positive mode]: found *m/z* 815 [M+H]$^+$, 837 [M+Na]$^+$.
HRMS [ESI-MS, positive mode]: MF: $C_{47}H_{47}N_4O_8F$; found m/z 815.3458 [M+H]$^+$ [calcd. 815.3456].

Compound 4Fb (±):
Color: Obtained as white solid; **Yield**: 20% (273 mg); **Mp**: 184-186 ºC; **R_f** 0.43 (6 % MeOH in $CHCl_3$);
IR (KBr, ν_{max} Cm^{-1}): 3402, 2958, 2869, 1714, 1613, 1518;

^{1}H NMR (*Py-d$_5$*): δ 12.03 (1H, s, -NH), 11.73 (1H, s, -NH), 7.28 (1H, dd, *J*=2.4, 7.8 Hz), 7.19 (1H, s), 7.16 (1H, d, *J*=8.4 Hz), 7.13 (2H, m), 7.08 (1H, d, *J*=1.8 Hz), 6.98 (1H, td, *J*=2.4, 9.0 Hz), 6.82 (3H, m), 6.69 (1H, s), 5.92 (1H, s), 4.38 (2H, m), 4.20 (1H, m), 4.13 (1H, m), 3.92 (1H, m), 3.78 (1H, m), 3.67 (3H, s), 3.64 (3H, s), 2.85 (1H, m), 2.67 (3H, m), 2.28 (3H, s), 2.12 (3H, s), 1.92 (1H, m), 1.85 (2H, m), 1.81 (2H, m), 1.71 (3H, m).

^{13}C NMR (*Py-d$_5$*): δ 190.6 (-C=O), 190.3 (-C=O), 181.5 (-C=O), 181.3 (-C=O), 158.6 (-C, $^{1}J_{C\text{-}F}$=237.0 Hz), 149.0 (2×-C), 147.44 (-C), 147.38 (-C), 139.9 (2×-C), 131.9 (-CH), 131.6 (-C), 131.3 (-C), 130.6 (-C), 128.4 (-C, $^{3}J_{C\text{-}F}$=7.5 Hz), 126.2 (-C), 125.5 (-CH), 120.3 (-CH), 120.2 (-CH), 119.4 (-C), 117.0 (-CH), 116.9 (-CH), 116.2 (-CH, $^{2}J_{C\text{-}F}$=24.0 Hz), 115.1 (-CH, $^{2}J_{C\text{-}F}$=24.0 Hz), 112.6 (-CH), 112.3 (-CH), 111.0 (-CH, $^{3}J_{C\text{-}F}$=7.5 Hz), 101.4 (-CH), 75.44 (-C), 75.41 (-C), 73.6 (-CH), 73.4 (-CH), 65.3 (-CH), 64.9 (-CH), 56.03 (-OMe), 55.98 (-OMe), 51.8 (-CH), 51.5 (-CH), 48.33 (-CH_2), 48.29 (-CH_2), 31.5 (-CH_2), 31.3 (-CH_2), 28.4 (-CH_2), 28.2 (-CH_2), 21.3 (-CH_3), 17.2 (-CH_3).

MS [ESI-MS, positive mode]: found *m/z* 815 $[M+H]^+$, 837 $[M+Na]^+$.

HRMS [ESI-MS, positive mode]: MF: $C_{47}H_{47}N_4O_8F$; found m/z 815.3456 $[M+H]^+$ [calcd. 815.3456].

Compound 4Fc (±):

Color: Obtained as white solid; **Yield**: 20% (273 mg); **Mp**: 173-175 ºC; $\mathbf{R_f}$ 0.37 (6 % MeOH in $CHCl_3$);

^{1}H NMR (*Py-d$_5$*): δ 11.77 (1H, s, -NH), 11.38 (1H, s, -NH), 7.63 (1H, m), 7.44 (1H, s), 7.34 (1H, s), 7.16 (1H, d, *J*=8.4 Hz), 7.12 (1H, m), 7.00 (1H, td, *J*=2.4, 9.0 Hz), 6.91 (2H, m), 6.86 (2H, m), 6.64 (1H, q, *J*=4.2 Hz), 6.10 (1H, s), 4.99 (1H, m), 4.74 (1H, m), 4.65 (1H, d, *J*=12.0 Hz), 4.33 (1H, m), 4.14 (1H, d, *J*=12.0 Hz), 4.05 (1H, m), 3.67 (3H, s), 3.51 (3H, s), 3.22 (1H, m), 2.95 (1H, m), 2.83 (1H, m), 2.75 (1H, m), 2.39 (3H, s), 2.28 (3H, s), 1.95 (3H, m), 1.78 (3H, m), 1.69 (2H, m).

^{13}C NMR (*Py-d$_5$*): δ 195.7 (-C=O), 189.0 (-C=O), 181.6 (-C=O), 180.1 (-C=O), 158.9 (-C, $^1J_{C-F}$=235.5 Hz), 149.2 (-C), 148.3 (-C), 147.61 (-C), 147.58 (-C), 140.9 (-C), 140.3 (-C), 131.9 (-CH), 131.6 (-C), 130.8 (-C), 129.8 (-C, $^3J_{C-F}$=7.4 Hz), 126.8 (-C), 126.7 (-C), 125.7 (-CH), 121.7 (-CH), 121.2 (-CH), 119.5 (-C), 117.1 (-CH), 116.4 (-CH), 116.1 (-CH, $^2J_{C-F}$=22.5 Hz), 115.2 (-CH, $^2J_{C-F}$=24.0 Hz), 112.7 (-CH), 112.4 (-CH), 110.9 (-CH, $^3J_{C-F}$=7.5 Hz), 102.8 (-CH), 76.8 (-C), 75.2 (-C), 73.8 (-CH), 65.9 (-CH), 64.6 (-CH), 56.1 (-OMe), 55.7 (-OMe), 53.2 (-CH), 52.6 (-CH), 52.0 (-CH), 51.5 ($-CH_2$), 48.6 ($-CH_2$), 31.3 ($-CH_2$), 29.0 ($-CH_2$), 28.2 ($-CH_2$), 26.9 ($-CH_2$), 21.3 ($-CH_3$), 17.4 ($-CH_3$).

MS [ESI-MS, positive mode]: found *m/z* 815 $[M+H]^+$, 837 $[M+Na]^+$.

HRMS [ESI-MS, positive mode]: MF: $C_{47}H_{47}N_4O_8F$; found m/z 815.3462 $[M+H]^+$ [calcd. 815.3456].

Compound 4Fd (±):

Color: Obtained as white solid; **Yield**: 22% (300 mg); **Mp**: 186-188 ºC; $\mathbf{R_f}$ 0.36 (6 % MeOH in $CHCl_3$);

^{1}H NMR (*Py-d$_5$*): δ 11.73 (1H, s, -NH), 11.37 (1H, s, -NH), 7.66 (1H, d, *J*=7.8 Hz), 7.54 (1H, s), 7.41 (1H, s), 7.18 (2H, s), 7.01 (1H, td, *J*=3.0, 9.0 Hz), 6.95 (1H, dd, *J*=1.8, 8.4 Hz), 6.90 (3H, m), 6.68 (1H, q, *J*=4.2 Hz), 6.00 (1H, s), 4.91 (1H, m), 4.71 (1H, m), 4.64 (1H, d, *J*=12.0 Hz), 4.40 (1H, m), 4.29 (1H, d, *J*=12.0 Hz), 4.12 (1H, m), 3.70 (3H, s), 3.49 (3H, s), 3.18 (1H, m), 2.97 (1H, m), 2.76 (2H, m), 2.37 (3H, s), 2.34 (3H, s), 2.25 (1H, m), 1.96 (3H, m), 1.79 (1H, m), 1.73 (1H, m), 1.63 (1H, m), 1.58 (1H, m).

^{13}C NMR (*Py-d$_5$*): δ 195.2 (-C=O), 190.8 (-C=O), 185.8 (-C=O), 180.3 (-C=O), 158.9 (-C, $^{1}J_{C-F}$=235.5 Hz), 149.2 (-C), 148.2 (-C), 147.45 (-C), 147.37 (-C), 140.9 (-C), 140.2 (-C), 131.9 (-CH), 130.7 (-C), 130.2 (-C), 129.6 (-C), 127.75 (-C), 127.0 (-C), 126.1 (-CH), 121.6 (-CH), 121.4 (-CH), 119.5 (-C), 117.0 (-CH), 116.3 (-CH), 116.0 (-CH, $^{2}J_{C-F}$=24.0 Hz), 115.9 (-CH, $^{2}J_{C-F}$=25.5 Hz), 113.3 (-CH), 112.5 (-CH), 110.8 (-CH, $^{3}J_{C-F}$=7.5 Hz), 102.85 (-CH), 76.8 (-C), 75.7 (-C), 73.7 (-CH), 65.1 (-CH), 64.7 (-CH), 56.1 (-OMe), 55.7 (-OMe), 54.4 (-CH), 52.6 (-CH), 51.3 (-CH, CH_2), 48.7 (-CH_2), 31.3 (-CH_2), 29.2 (-CH_2), 28.2 (-CH_2), 26.8 (-CH_2), 21.4 (-CH_3), 17.4 (-CH_3).

MS [ESI-MS, positive mode]: found *m/z* 815 $[M+H]^+$, 837 $[M+Na]^+$.
HRMS [ESI-MS, positive mode]: MF: $C_{47}H_{47}N_4O_8F$; found m/z 815.3452 $[M+H]^+$ [calcd. 815.3456].

Compound 5Aa (±):
Color: Obtained as white solid; **Yield**: 30% (655 mg); **Mp**: 230-232 ºC; **R$_f$** 0.65 (5 % MeOH in $CHCl_3$);
IR (KBr, ν$_{max}$ Cm^{-1}): 3336, 2927, 1725, 1615, 1517;

^{1}H NMR (*Py-d$_5$*): δ 11.26 (2H, s, -NH), 7.64 (2H, d, *J*=7.8 Hz), 7.31 (2H, m), 7.25 (2H, dd, *J*=0.6, 7.8 Hz), 7.22 (2H, m), 6.99 (4H, m), 6.93 (2H, d, *J*=7.8 Hz), 5.74 (1H, s), 4.36 (2H, d, *J*=12.6 Hz), 4.33 (2H, m), 3.92 (2H, d, *J*=10.2 Hz), 3.76 (6H, s), 3.65 (4H, m), 3.11 (2H, dd, *J*=2.4, 11.4 Hz), 3.00 (2H, m).

^{13}C NMR (*Py-d$_5$*): δ 190.5 (-C=O), 180.0 (-C=O), 149.5 (-C), 148.0 (-C), 143.8 (-C), 130.5 (-C), 130.3 (-CH), 128.5 (-CH), 124.5 (-C), 121.7 (-CH), 121.5 (-CH), 117.3 (-CH), 111.7 (-CH), 110.5 (-CH), 101.4 (-CH), 76.7 (-CH), 75.1 (-C), 62.6 (-CH), 56.2 (-OMe), 54.9 (-CH_2), 51.5 (-CH), 36.7 (-CH_2).

MS [ESI-MS, positive mode]: found *m/z* 827 $[M+Na]^+$.
HRMS [ESI-MS, positive mode]: MF: $C_{43}H_{40}N_4O_8S_2$; found m/z 827.2180 $[M+Na]^+$ [calcd. 827.2185].

Compound 5Ab (±):
Color: Obtained as white solid; **Yield**: 35% (765 mg); **Mp**: 242-244 ºC; **R$_f$** 0.55 (5 % MeOH in $CHCl_3$);
IR (KBr, ν$_{max}$ Cm^{-1}): 3407, 2928, 1718, 1617, 1518;

^{1}H NMR (*Py-d$_5$*): δ 11.94 (2H, s, -NH), 7.43 (4H, m), 7.30 (2H, d, *J*=1.8 Hz), 7.20 (1H, d, *J*=1.8 Hz), 7.18 (3H, t, *J*=7.8 Hz), 6.94 (4H, m), 5.98 (1H, s), 4.36 (2H, d, *J*=13.2 Hz), 4.32 (2H, t, *J*=7.8 Hz), 3.92 (2H, d, *J*=10.8 Hz), 3.86 (6H, s), 3.63 (4H, m), 3.15 (2H, d, *J*=11.4 Hz), 3.00 (2H, dd, *J*=6.6, 11.4 Hz).

^{13}C NMR (*Py-d$_5$*): δ 190.5 (-C=O), 180.3 (-C=O), 148.2 (2×-C), 144.0 (-C), 130.3 (-CH), 130.2 (-C), 128.4 (-CH), 123.5 (-C), 121.8 (-CH), 121.6 (-CH), 117.5 (-CH), 111.7 (-CH), 110.5 (-CH), 101.1 (-CH), 76.9 (-CH), 75.5 (-C), 61.6 (-CH), 56.3 (-OMe), 55.9 (-CH_2), 51.9 (-CH), 36.9 (-CH_2).

MS [ESI-MS, positive mode]: found *m/z* 827 [M+Na] $^+$.
HRMS [ESI-MS, positive mode]: MF: $C_{43}H_{40}N_4O_8S_2$; found m/z 827.2167 [M+Na] $^+$ [calcd. 827.2185].

<u>Compound 5Ba (±):</u>
Color: Obtained as white solid; **Yield**: 31% (700 mg); **Mp**: 216-218 ºC; **R$_f$** 0.60 (5 % MeOH in $CHCl_3$);
IR (KBr, ν_{max} Cm^{-1}): 3379, 2923, 1718, 1602, 1521;

^{1}H NMR (*Py-d$_5$*): δ 10.96 (2H, s, -NH), 7.52 (2H, s), 7.27 (2H, d, *J*=8.4 Hz), 7.22 (2H, m), 7.00 (2H, dd, *J*=1.8, 8.4 Hz), 6.94 (2H, d, *J*=8.4 Hz), 6.84 (2H, d, *J*=7.8 Hz), 5.66 (1H, s), 4.36 (2H, m), 4.30 (2H, d, *J*=12.0 Hz), 3.88 (2H, d, *J*=10.2 Hz), 3.78 (2H, m), 3.73 (6H, s), 3.60 (2H, d, *J*=9.6 Hz), 3.10 (2H, dd, J=2.4, 10.8 Hz), 2.98 (2H, m), 2.12 (6H, s).

^{13}C NMR (*Py-d$_5$*): δ 190.2 (-C=O), 180.2 (-C=O), 149.3 (-C), 147.7 (-C), 141.1 (-C), 130.9 (-C), 130.724 (-C), 130.716 (-CH), 129.1 (-CH), 124.6 (-C), 121.2 (-CH), 117.3 (-CH), 112.2 (-CH), 110.3 (-CH), 101.6 (-CH), 76.4 (-CH), 75.2 (-C), 62.9 (-CH), 56.2 (-OMe), 54.6 (-CH_2), 50.9 (-CH), 36.7 (-CH_2), 21.3 (-CH_3).

MS [ESI-MS, positive mode]: found *m/z* 855 [M+Na] $^+$.
HRMS [ESI-MS, positive mode]: MF: $C_{45}H_{44}N_4O_8S_2$; found m/z 855.2495 [M+Na] $^+$ [calcd. 855.2498].

<u>Compound 5Bb (±):</u>
Color: Obtained as white solid; **Yield**: 35% (791 mg); **Mp**: 260-262 ºC; **R$_f$** 0.53 (5 % MeOH in $CHCl_3$);
IR (KBr, ν_{max} Cm^{-1}): 3388, 2921, 1708, 1617, 1516;

^{1}H NMR (*Py-d$_5$*): δ 11.93 (2H, s, -NH), 7.55 (2H, s), 7.17 (2H, d, *J*=7.8 Hz), 7.11 (2H, d, *J*=1.8 Hz), 6.93 (2H, d, *J*=7.8 Hz), 6.88 (2H, dd, *J*=1.2, 7.8 Hz), 6.79 (2H, d, *J*=7.8 Hz), 5.88 (1H, s), 4.30 (2H, m), 4.27 (2H, d, *J*=12.0 Hz), 3.95 (2H, d, *J*=10.2 Hz), 3.86 (2H, m), 3.70 (2H, d, *J*=10.2 Hz), 3.67 (6H, s), 3.15 (2H, dd, *J*=1.8, 11.4 Hz), 3.02 (2H, m), 2.08 (6H, s).

^{13}C NMR (*Py-d$_5$*): δ 189.9 (-C=O), 180.3 (-C=O), 149.2 (-C), 147.8 (-C), 141.4 (-C), 130.8 (-CH), 130.7 (-C), 130.5 (-C), 129.2 (-CH), 124.6 (-C), 120.8 (-CH), 117.2 (-CH), 112.4 (-CH), 110.0 (-CH), 101.3 (-CH), 76.7 (-CH), 75.9 (-C), 63.0 (-CH), 56.0 (-OMe), 55.1 (-CH_2), 50.8 (-CH), 37.0 (-CH_2), 21.3 (-CH_3).

MS [ESI-MS, positive mode]: found *m/z* 855 [M+Na]$^+$.
HRMS [ESI-MS, positive mode]: MF: $C_{45}H_{44}N_4O_8S_2$; found m/z 855.2498 [M+Na]$^+$ [calcd. 855.2498].

__**Compound 6Aa (±):**__
Color: Obtained as white solid; **Yield**: 30% (431 mg); **Mp**: 172-174 ºC; **R_f** 0.60 (5 % MeOH in $CHCl_3$);
IR (KBr, $ν_{max}$ Cm^{-1}): 3290, 2926, 1720, 1608, 1520;

^{1}H NMR (*Py-d$_5$*): δ 11.13 (2H, s, -NH), 7.61 (1H, s), 7.32 (2H, d, *J*=8.4 Hz), 7.26 (1H, d, *J*=8.4 Hz), 7.18 (2H, m), 7.15 (1H, m), 7.13 (1H, s), 6.94 (4H, m), 6.81 (1H, m), 5.77 (1H, s), 4.49 (1H, d, *J*=12.0 Hz), 4.36 (1H, d, *J*=12.6 Hz), 4.32 (1H, m), 4.23 (1H, m), 3.96 (1H, d, *J*=9.6 Hz), 3.81 (3H, s), 3.73 (3H, s), 3.70 (1H, m), 3.68 (3H, s), 3.63 (2H, m), 3.08 (1H, d, *J*=10.2 Hz), 2.98 (1H, m), 2.76 (1H, m), 2.61 (1H, m), 1.89 (1H, m), 1.79 (2H, m), 1.67 (1H, m).

^{13}C NMR (*Py-d$_5$*): δ 191.8 (-C=O), 189.9 (-C=O), 181.0 (-C=O), 179.9 (-C=O), 155.2 (-C), 149.4 (-C), 149.3 (-C), 147.9 (-C), 147.6 (-C), 143.8 (-C), 137.2 (-C), 131.8 (-C), 130.2 (-C), 129.7 (-CH), 126.94 (-C), 126.89 (-CH), 125.9 (-C), 121.8 (-CH), 121.6 (-CH), 121.1 (-CH), 117.3 (-CH), 117.2 (-CH), 116.4 (-CH), 115.0 (-CH), 111.8 (-CH), 111.5 (-CH), 110.8 (-CH), 110.6 (-CH), 101.6 (-CH), 76.5 (-CH), 75.7 (-C), 74.4 (-C), 73.9 (-CH), 64.0 (-CH), 63.0 (-CH), 56.19 (-OMe), 56.16 (-OMe), 56.12 (-OMe), 55.1 (-CH_2), 52.7 (-CH), 51.6 (-CH), 48.6 (-CH_2), 36.7 (-CH_2), 30.9 (-CH_2), 27.6 (-CH_2).

MS [ESI-MS, positive mode]: found *m/z* 817 [M+H]$^+$, 839 [M+Na]$^+$.
HRMS [ESI-MS, positive mode]: MF: $C_{45}H_{44}N_4O_9S$; found m/z 839.2722 [M+Na]$^+$ [calcd. 839.2727].

Compound 6Ab (±):

Color: Obtained as white solid; **Yield**: 35% (503 mg); **Mp**: 176-178 ºC; $\mathbf{R_f}$ 0.55 (5 % MeOH in $CHCl_3$);

^{1}H NMR (*Py-d$_5$*): δ 11.91 (1H, s, -NH), 11.78 (1H, s, -NH), 7.49 (1H, d, *J*=2.4 Hz), 7.22 (2H, m), 7.19 (1H, m), 7.16 (2H, dd, *J*=1.8, 7.8 Hz), 7.13 (1H, d, *J*=7.2 Hz), 7.01 (1H, dd, *J*=1.8, 7.8 Hz), 6.94 (4H, m), 6.87 (1H, dd, *J*=2.4, 8.4 Hz), 5.95 (1H, s), 4.38 (2H, m), 4.28 (1H, m), 4.16 (1H, m), 3.97 (1H, d, J=10.8 Hz), 3.79 (1H, m), 3.76 (3H, s), 3.72 (3H, s), 3.68 (1H, m), 3.63 (3H, s, 1H, m), 3.13 (1H, dd, *J*=1.8, 11.4 Hz), 3.00 (1H, m), 2.69 (1H, m), 2.60 (1H, m), 1.85 (1H, m), 1.68 (3H, m).

^{13}C NMR (*Py-d$_5$*): δ 191.2 (-C=O), 189.6 (-C=O), 181.1 (-C=O), 180.3 (-C=O), 155.1 (-C), 149.3 (-C), 149.2 (-C), 147.9 (-C), 147.6 (-C), 143.8 (-C), 137.3 (-C), 131.3 (-C), 130.6 (-C), 129.7 (-CH), 127.1 (-CH), 126.6 (-C), 125.8 (-C), 121.7 (-CH), 121.3 (-CH), 120.9 (-CH), 117.2 (-CH), 117.1 (-CH), 115.8 (-CH), 115.3 (-CH), 112.1 (-CH), 111.9 (-CH), 110.7 (-CH), 110.6 (-CH), 101.2 (-CH), 76.7 (-CH), 76.1 (-C), 74.9 (-C), 73.8 (-CH), 64.1 (-CH), 62.7 (-CH), 56.15 (-OMe), 56.11 (-OMe), 55.90 (-OMe), 55.6 (-CH_2), 52.3 (-CH), 51.2 (-CH), 48.5 (-CH_2), 37.0 (-CH_2), 31.3 (-CH_2), 28.0 (-CH_2).

MS [ESI-MS, positive mode]: found *m/z* 817 $[M+H]^+$, 839 $[M+Na]^+$.

HRMS [ESI-MS, positive mode]: MF: $C_{45}H_{44}N_4O_9S$; found m/z 839.2728 $[M+Na]^+$ [calcd. 839.2727].

Compound 6Ba (±):

Color: Obtained as white solid; **Yield**: 32% (508 mg); **Mp**: 212-214 ºC; $\mathbf{R_f}$ 0.56 (5 % MeOH in $CHCl_3$);

IR (KBr, ν_{max} Cm^{-1}): 3511, 3209, 2924, 1713, 1606, 1515;

^{1}H NMR (*Py-d$_5$*): δ 11.73 (1H, s, -NH), 11.29 (1H, s, -NH), 8.38 (1H, d, *J*=1.2 Hz), 7.84 (1H, d, *J*=7.8 Hz), 7.42 (1H, d, *J*=1.2 Hz), 7.37 (1H, d, *J*=7.8 Hz), 7.28 (3H, m), 7.08 (1H, d, *J*=1.8 Hz), 7.01 (1H, m), 6.85 (2H, m), 6.60 (1H, dd, *J*=1.8, 7.8 Hz), 5.79 (1H, s), 4.64 (1H, d, *J*=12.0 Hz), 4.32 (1H, d, *J*=12.6 Hz), 4.26 (1H, m), 4.17 (1H, m), 3.94 (1H, d, *J*=10.8 Hz), 3.88 (3H, s), 3.80 (1H, m), 3.69 (1H, m), 3.62 (3H, s), 3.48 (1H, m), 3.04 (1H, m), 2.95 (1H, m), 2.66 (2H, m), 1.86 (3H, m), 1.66 (1H, m).

^{13}C NMR (*Py-d$_5$*): δ 193.3 (-C=O), 188.9 (-C=O), 180.8 (-C=O), 179.3 (-C=O), 158.7 (-C, $^1J_{C-F}$=239.1 Hz), 149.6 (-C), 149.5 (-C), 148.1 (-C), 147.8 (-C), 143.8 (-C), 140.1 (-C), 139.6

(-CH), 137.1 (-CH), 132.0 (-C), 129.6 (-C), 129.4 (-C), 127.1 (-C), 122.4 (-CH), 120.8 (-CH), 117.7 (-CH), 117.2 (-CH), 115.9 (-CH, $^{2}J_{C-F}$=22.5 Hz), 114.3 (-CH, $^{2}J_{C-F}$=24 Hz), 112.9 (-CH), 112.0 (-CH), 111.0 (-CH, $^{3}J_{C-F}$=6.45 Hz), 110.7 (-CH), 101.0 (-CH), 84.4 (-C), 76.6 (-CH), 75.5 (-C), 74.9 (-CH), 74.6 (-C), 64.1 (-CH), 62.6 (-CH), 56.5 (-OMe), 56.0 (-OMe), 55.7 (-CH_2), 52.5 (-CH), 51.7 (-CH), 48.2 (-CH_2), 36.9 (-CH_2), 31.1 (-CH_2), 28.0 (-CH_2).

MS [ESI-MS, positive mode]: found *m/z* 953 $[M+Na]^{+}$.
HRMS [ESI-MS, positive mode]: MF: $C_{44}H_{40}N_4O_8IFS$; found m/z 953.1488 $[M+Na]^{+}$ [calcd. 953.1493].

Compound 6Bb (±):
Color: Obtained as white solid; **Yield**: 37% (587 mg); **Mp**: 220-222 ºC; $\mathbf{R_f}$ 0.48 (5 % MeOH in $CHCl_3$);
^{1}H NMR (*Py-d$_5$*): δ 12.25 (1H, s, -NH), 11.88 (1H, s, -NH), 8.31 (1H, s), 7.59 (1H, d, *J*=7.8 Hz), 7.30 (1H, d, *J*=7.8 Hz), 7.14 (1H, d, *J*=7.8 Hz), 7.09 (1H, d, *J*=8.4 Hz), 7.06 (1H, s), 7.02 (1H, s), 6.96 (1H, m), 6.81 (2H, m), 6.76 (2H, d, *J*=7.8 Hz), 5.82 (1H, s), 4.32 (1H, d, *J*=12.0 Hz), 4.25 (1H, m), 4.21 (1H, d, *J*=12.0 Hz), 4.14 (1H, m), 3.93 (1H, d, *J*=10.8 Hz), 3.83 (1H, m), 3.73 (3H, s), 3.71 (1H, m), 3.65 (1H, m), 3.63 (3H, s), 3.12 (1H, d, *J*=12.0 Hz), 2.99 (1H, m), 2.63 (2H, m), 1.84 (1H, m), 1.76 (1H, m), 1.67 (2H, m).

^{13}C NMR (*Py-d$_5$*): δ 190.4 (-C=O), 189.2 (-C=O), 181.0 (-C=O), 179.6 (-C=O), 158.6 (-C, $^{1}J_{C-F}$=237.0 Hz), 149.1 (-C), 149.0 (-C), 147.8 (-C), 147.4 (-C), 143.5 (-C), 139.8 (-C), 139.1 (-CH), 137.4 (-CH), 131.1 (-C), 130.1 (-C), 128.3 (-C), 127.0 (-C), 120.7 (-CH), 120.3 (-CH), 117.2 (-CH), 117.1 (-CH), 116.2 (-CH, $^{2}J_{C-F}$=24.0 Hz), 115.0 (-CH, $^{2}J_{C-F}$=24 Hz), 112.6 (-CH), 112.5 (2×-CH), 111.2 (-CH, $^{3}J_{C-F}$=7.5 Hz), 101.7 (-CH), 84.5 (-C), 76.4 (-CH), 76.0 (-C), 75.3 (-C), 73.6 (-CH), 64.8 (-CH), 63.5 (-CH), 56.1 (-OMe), 56.0 (-OMe), 55.5 (-CH_2), 51.9 (-CH), 51.0 (-CH), 48.2 (-CH_2), 37.1 (-CH_2), 31.4 (-CH_2), 28.3 (-CH_2).

MS [ESI-MS, positive mode]: found *m/z* 953 $[M+Na]^{+}$.
HRMS [ESI-MS, positive mode]: MF: $C_{44}H_{40}N_4O_8IFS$; found m/z 953.1487 $[M+Na]^{+}$ [calcd. 953.1493].

Compound 6Ca (±):
Color: Obtained as white solid; **Yield**: 31% (455 mg); **Mp**: 180-182 ºC; $\mathbf{R_f}$ 0.54 (5 % MeOH in $CHCl_3$);
IR (KBr, ν_{max} Cm^{-1}): 3514, 3200, 1713, 1610, 1515;

^{1}H NMR (*Py-d$_5$*): δ 11.61 (1H, s, -NH), 11.39 (1H, s, -NH), 8.29 (1H, s), 7.76 (1H, d, *J*=7.8 Hz), 7.57 (1H, m), 7.34 (2H, m), 7.28 (2H, m), 7.18 (1H, d, *J*=7.8 Hz), 7.13 (1H, s), 6.88 (1H, d, *J*=7.8 Hz), 6.80 (1H, d, *J*=7.8 Hz), 6.77 (1H, d, *J*=8.4 Hz), 5.74 (1H, s), 4.57 (1H, d, *J*=12.0 Hz), 4.38 (1H, m), 4.27 (1H, m), 4.20 (1H, m), 3.92 (1H, d, *J*=10.8 Hz), 3.82 (1H, m), 3.80 (3H, s), 3.67 (3H, s), 3.64 (1H, m), 3.57 (1H, m), 3.08 (1H, d, *J*=11.4 Hz), 2.96 (1H, m), 2.71 (1H, m), 2.61 (1H, m), 1.82 (3H, m), 1.66 (1H, m).

^{13}C NMR (*Py-d$_5$*): δ 192.8 (-C=O), 189.0 (-C=O), 180.5 (-C=O), 179.3 (-C=O), 149.9 (-C), 149.5 (-C), 148.0 (-C), 147.7 (-C), 143.7 (-C), 142.6 (-C), 139.4 (-CH), 137.0 (-CH), 131.9 (-C), 130.0 (-CH), 129.8 (-C), 129.3 (-C), 127.2 (-C), 126.9 (-CH), 126.6 (-C), 121.9 (-CH), 120.9 (-CH), 117.5 (-CH), 117.3 (-CH), 112.8 (-CH), 112.0 (-CH), 111.8 (-CH), 111.2 (-CH), 101.1 (-CH), 84.5 (-C), 76.7 (-CH), 75.2 (2×-C), 74.4 (-CH), 64.6 (-CH), 62.4 (-CH), 56.3 (-OMe), 56.1 (-OMe), 55.3 (-CH_2), 52.4 (-CH), 51.4 (-CH), 48.2 (-CH_2), 36.9 (-CH_2), 31.0 (-CH_2), 27.9 (-CH_2).

MS [ESI-MS, positive mode]: found *m/z* 947 $[M+H]^+$.
HRMS [ESI-MS, positive mode]: MF: $C_{44}H_{40}ClIN_4O_8S$; found m/z 947.1375 $[M+H]^+$ [calcd. 947.1378].

Compound 6Cb (±):
Color: Obtained as white solid; **Yield**: 35% (549 mg); **Mp**: 230-232 ºC; **R_f** 0.46 (5 % MeOH in $CHCl_3$);
^{1}H NMR (*Py-d$_5$*): δ 12.23 (1H, s, -NH), 12.06 (1H, s, -NH), 8.30 (1H, s), 7.59 (1H, m), 7.55 (1H, dd, *J*=1.8, 8.4 Hz), 7.17 (1H, dd, *J*=1.8, 8.4 Hz), 7.10 (2H, dd, *J*=3.0, 8.4 Hz), 7.02 (2H, m), 6.79 (1H, d, *J*=8.4 Hz), 6.75 (2H, m), 6.68 (1H, d, *J*=7.8 Hz), 5.80 (1H, s), 4.28 (2H, d, *J*=12.0 Hz), 4.18 (1H, m), 4.14 (1H, m), 3.92 (1H, d, *J*=10.8 Hz), 3.82 (2H, t, *J*=10.8 Hz), 3.68 (3H, s), 3.64 (3H, s), 3.62 (1H, m), 3.10 (1H, m), 2.99 (1H, m), 2.67 (1H, m), 2.60 (1H, m), 1.85 (1H, m), 1.79 (1H, m), 1.70 (2H, m).

^{13}C NMR (*Py-d$_5$*): δ 189.8 (2×-C=O), 180.8 (-C=O), 179.5 (-C=O), 149.0 (-C), 148.9 (-C), 147.7 (-C), 147.3 (-C), 143.5 (-C), 142.4 (-C), 139.1 (-CH), 137.4 (-CH), 131.1 (-C), 130.1 (-C), 129.9 (-CH), 128.5 (-C), 127.6 (-CH), 127.0 (-C), 126.7 (-C), 120.5 (-CH), 120.1 (-CH), 117.1 (-CH), 117.0 (-CH), 112.8 (-CH), 112.6 (-CH), 112.5 (-CH), 111.8 (-CH), 101.7 (-CH), 84.5 (-C), 76.3 (-CH), 76.1 (-C), 75.3 (-C), 73.3 (-CH), 65.5 (-CH), 63.6 (-CH), 56.13 (-OMe), 56.06 (-OMe), 55.5 (-CH_2), 51.7 (-CH), 50.9 (-CH), 48.2 (-CH_2), 37.1 (-CH_2), 31.3 (-CH_2), 28.4 (-CH_2).

MS [ESI-MS, positive mode]: found *m/z* 947 $[M+H]^+$.

HRMS [ESI-MS, positive mode]: MF: $C_{44}H_{40}ClIN_4O_8S$; found m/z 969.1193 $[M+Na]^+$ [calcd. 969.1198].

Compound 6Da (±):

Color: Obtained as white solid; **Yield**: 32% (452 mg); **Mp**: 202-204 ºC; $\mathbf{R_f}$ 0.55 (5 % MeOH in $CHCl_3$);

IR (KBr, ν_{max} Cm^{-1}): 3411, 2930, 1721, 1604, 1518;

^{1}H NMR (*Py-d$_5$*): δ 11.26 (1H, s, -NH), 10.63 (1H, s, -NH), 7.43 (1H, s), 7.28 (2H, t, *J*=8.4 Hz), 7.23 (2H, m), 7.10 (1H, d, *J*=2.4 Hz), 7.06 (1H, dd, *J*=1.2, 7.8 Hz), 7.01 (1H, dd, *J*=1.2, 7.8 Hz), 6.86 (1H, d, *J*=7.8 Hz), 6.81 (1H, dd, *J*=2.4, 8.4 Hz), 6.75 (1H, s), 5.72 (1H, s), 4.38 (3H, m), 4.28 (1H, m), 3.95 (1H, m), 3.81 (1H, m), 3.75 (3H, s), 3.74 (3H, s), 3.71 (2H, m), 3.64 (3H, s), 3.17 (1H, m), 3.00 (1H, m), 2.88 (1H, m), 2.62 (1H, m), 2.23 (3H, s), 2.15 (3H, s), 1.88 (1H, m), 1.79 (1H, m), 1.70 (2H, m).

^{13}C NMR (*Py-d$_5$*): δ 194.0 (-C=O), 187.9 (-C=O), 180.9 (-C=O), 180.4 (-C=O), 155.4 (-C), 149.4 (-C), 149.1 (-C), 147.7 (-C), 147.5 (-C), 140.0 (-C), 136.8 (-C), 132.3 (-CH), 132.0 (-C), 130.6 (-C), 130.5 (-C), 127.8 (-C), 126.5 (-CH), 124.2 (-C), 121.3 (-CH), 121.0 (-CH), 119.0 (-C), 117.3 (-CH), 117.1 (-CH), 115.0 (-CH), 114.5 (-CH), 112.4 (-CH), 111.7 (-CH), 110.8 (-CH), 101.9 (-CH), 77.1 (-CH), 75.5 (-C), 74.8 (-C), 72.9 (-CH), 66.1 (-CH), 61.5 (-CH), 56.13 (2×-OMe), 56.09 (-OMe), 55.2 ($-CH_2$), 52.5 (-CH), 51.1 (-CH), 48.5 ($-CH_2$), 36.7 ($-CH_2$), 30.6 ($-CH_2$), 27.5 ($-CH_2$), 21.1 ($-CH_3$), 17.2 ($-CH_3$).

MS [ESI-MS, positive mode]: found *m/z* 845 $[M+H]^+$.

HRMS [ESI-MS, positive mode]: MF: $C_{47}H_{48}N_4O_9S$; found m/z 845.3238 $[M+H]^+$ [calcd. 845.3220].

Compound 6Db (±):

Color: Obtained as white solid; **Yield**: 36% (508 mg); **Mp**: 182-184 ºC; $\mathbf{R_f}$ 0.47 (5 % MeOH in $CHCl_3$);

^{1}H NMR (*Py-d$_5$*): δ 11.83 (1H, s, -NH), 11.79 (1H, s, -NH), 7.47 (1H, s), 7.18 (1H, dd, *J*=1.2, 7.8 Hz), 7.12 (2H, m), 7.07 (2H, m), 6.84 (3H, m), 6.77 (1H, dd, *J*=2.4, 8.4 Hz), 6.69 (1H, s), 5.87 (1H, d, *J*=1.2 Hz), 4.33 (2H, m), 4.27 (1H, d, *J*=12.6 Hz), 4.19 (1H, m), 3.98 (1H, d, *J*=10.2 Hz), 3.90 (1H, m), 3.80 (1H, t, *J*=10.2 Hz), 3.75 (1H, d, *J*=10.2 Hz), 3.68 (3H,

s), 3.64 (3H, s), 3.54 (3H, s), 3.17 (1H, m), 3.04 (1H, m), 2.81 (1H, m), 2.64 (1H, m), 2.26 (3H, s), 2.10 (3H, s), 1.87 (1H, m), 1.79 (1H, m), 1.69 (2H, m).

^{13}C NMR (*Py-d$_5$*): δ 191.0 (-C=O), 189.3 (-C=O), 181.2 (-C=O), 180.6 (-C=O), 155.4 (-C), 149.1 (-C), 149.0 (-C), 147.7 (-C), 147.4 (-C), 140.0 (-C), 137.1 (-C), 132.3 (-CH), 131.6 (-C), 130.7 (-C), 130.4 (-C), 127.9 (-C), 126.5 (-CH), 124.3 (-C), 120.7 (-CH), 120.3 (-CH), 119.1 (-C), 117.2 (-CH), 116.9 (-CH), 115.3 (-CH), 114.1 (-CH), 112.6 (-CH), 112.4 (-CH), 110.7 (-CH), 101.4 (-CH), 76.6 (-CH), 76.1 (-C), 75.5 (-C), 73.4 (-CH), 65.2 (-CH), 63.3 (-CH), 56.1 (-OMe), 56.0 (-OMe), 55.8 (-OMe), 55.0 (-CH_2), 51.8 (-CH), 50.7 (-CH), 48.4 (-CH_2), 36.9 (-CH_2), 31.3 (-CH_2), 28.1 (-CH_2), 21.3 (-CH_3), 17.2 (-CH_3).

MS [ESI-MS, positive mode]: found *m/z* 845 $[M+H]^+$.
HRMS [ESI-MS, positive mode]: MF: $C_{47}H_{48}N_4O_9S$; found m/z 845.3209 $[M+H]^+$ [calcd. 845.3220].

<u>Compound 6Ea (±):</u>
Color: Obtained as white solid; **Yield**: 31% (441 mg); **Mp**: 172-174 ºC; **R_f** 0.51 (5 % MeOH in $CHCl_3$);
^{1}H NMR (*Py-d$_5$*): δ 11.16 (1H, s, -NH), 10.69 (1H, s, -NH), 7.46 (1H, s), 7.30 (2H, m), 7.28 (1H, s), 7.25 (2H, d, *J*= 9.6 Hz), 7.08 (1H, d, *J*= 7.8 Hz), 7.03 (1H, d, *J*=7.8 Hz), 6.95 (1H, d, *J*= 7.8 Hz), 6.83 (1H, d, *J*=7.8 Hz), 6.73 (1H, s), 5.73 (1H, s), 4.37 (3H, m), 3.95 (1H, d, *J*=10.2 Hz), 3.87 (1H, m), 3.81 (1H, m), 3.76 (3H, s), 3.73 (3H, s), 3.71 (1H, m), 3.16 (1H, m), 3.01 (1H, m), 2.86 (1H, m), 2.63 (1H, m), 2.24 (3H, s), 2.15 (6H, s), 2.01 (1H, m), 1.90 (1H, m), 1.82 (2H, m), 1.69 (1H, m).

^{13}C NMR (*Py-d$_5$*): δ 192.6 (-C=O), 187.8 (-C=O), 180.5 (-C=O), 179.9 (-C=O), 148.7 (-C), 148.5 (-C), 147.1 (-C), 146.8 (-C), 140.4 (-C), 139.3 (-C), 131.7 (-CH), 131.4 (-C), 130.2 (3×-C), 129.9 (-C), 129.6 (-CH), 127.5 (-CH), 126.0 (-C), 125.9 (-CH), 120.5 (-CH), 120.3 (-CH), 118.4 (-C), 116.7 (-CH), 116.5 (-CH), 111.8 (-CH), 111.4 (-CH), 109.7 (-CH), 101.2 (-CH), 76.2 (-CH), 75.0 (-C), 74.1 (-C), 72.6 (-CH), 65.0 (-CH), 61.4 (-CH), 55.5 (2×-OMe), 54.4 (-CH_2), 51.7 (-CH), 50.5 (-CH), 48.0 (-CH_2), 36.1 (-CH_2), 30.2 (-CH_2), 27.0 (-CH_2), 20.7 (-CH_3), 20.6 (-CH_3), 16.6 (-CH_3).

MS [ESI-MS, positive mode]: found *m/z* 829 $[M+H]^+$.
HRMS [ESI-MS, positive mode]: MF: $C_{47}H_{49}N_4O_8S$; found m/z 829.3268 $[M+H]^+$ [calcd. 829.3271].

Compound 6Eb (±):

Color: Obtained as white solid; **Yield**: 36% (512 mg); **Mp**: 169-171 ºC; $\mathbf{R_f}$ 0.45 (5 % MeOH in $CHCl_3$);

IR (KBr, ν_{max} Cm^{-1}): 3404, 2926, 1712, 1605, 1517, 1485;

^{1}H NMR (*Py-d$_5$*): δ 7.41 (1H, s), 7.17 (2H, m), 7.09 (3H, m), 6.90 (1H, d, *J*=7.8 Hz), 6.82 (2H, m), 6.78 (1H, d, *J*=7.8 Hz), 6.64 (1H, s), 5.85 (1H, s), 4.27 (3H, m), 4.16 (1H, m), 3.93 (1H, d, *J*=9.6 Hz), 3.83 (2H, q, *J*=9.6, 21 Hz), 3.70 (1H, d, *J*=9.6 Hz), 3.63 (3H, s), 3.58 (3H, s), 3.13 (1H, m), 2.99 (1H, m), 2.78 (1H, m), 2.59 (1H, m), 2.21 (3H, s), 2.05 (3H, s), 2.00 (3H, s), 1.84 (1H, m), 1.75 (2H, m), 1.64 (1H, m).

^{13}C NMR (*Py-d$_5$*): δ 190.6 (-C=O), 188.8 (-C=O), 180.8 (-C=O), 180.1 (-C=O), 148.6 (-C), 148.4 (-C), 147.2 (-C), 146.8 (-C), 140.7 (-C), 139.5 (-C), 131.8 (-CH), 131.1 (-C), 130.2 (2×-C), 130.1 (-C), 129.8 (-C), 129.7 (-CH), 127.5 (-CH), 126.0 (-C), 125.9 (-CH), 120.1 (-CH), 119.7 (-CH), 118.5 (-C), 116.6 (-CH), 116.4 (-CH), 111.9 (-CH), 111.7 (-CH), 109.7 (-CH), 100.7 (-CH), 76.1 (-CH), 75.5 (-C), 74.7 (-C), 73.0 (-CH), 64.5 (-CH), 62.5 (-CH), 55.5 (-OMe), 55.4 (-OMe), 54.5 (-CH_2), 51.1 (-CH), 50.2 (-CH), 47.9 (-CH_2), 36.3 (-CH_2), 30.7 (-CH_2), 27.5 (-CH_2), 20.8 (-CH_3), 20.7 (-CH_3), 16.7 (-CH_3).

MS [ESI-MS, positive mode]: found *m/z* 829 $[M+H]^+$.

HRMS [ESI-MS, positive mode]: MF: $C_{47}H_{49}N_4O_8S$; found m/z 829.3268 $[M+H]^+$ [calcd. 829.3271].

Compound 7A (±):

Color: Obtained as white solid; **Yield**: 54% (636 mg); **Mp**: 192-194 ºC; $\mathbf{R_f}$ 0.52 (5 % MeOH in $CHCl_3$);

IR (KBr, ν_{max} Cm^{-1}): 3511, 2960, 1720, 1607, 1515;

^{1}H NMR (*Py-d$_5$*): δ 11.82 (1H, s, -NH), 7.87 (2H, m), 7.84 (2H, m), 7.76 (1H, t, *J*= 7.2 Hz), 7.69 (1H, d, *J*=7.2 Hz), 7.41 (1H, t, *J*=7.2 Hz), 7.37 (1H, d, *J*=1.2 Hz), 7.30 (1H, t, *J*=7.8 Hz), 7.16 (3H, m), 7.02 (1H, d, *J*=7.8 Hz), 6.79 (1H, dd, *J*=1.2, 8.4 Hz), 6.75 (1H, d, *J*=8.4 Hz), 6.70 (1H, s), 6.03 (1H, s), 4.96 (1H, m), 4.61 (2H, m), 4.45 (1H, d, *J*=11.4 Hz), 4.38 (1H, m), 4.01 (1H, dd, *J*=9.6, 12 Hz), 3.71 (3H, s), 3.36 (3H, s), 3.14 (1H, m), 2.90 (1H, m), 2.74 (1H, m), 2.54 (1H, t, *J*=7.2 Hz), 1.98 (1H, m), 1.90 (2H, m), 1.78 (1H, m), 1.70 (1H, m), 1.64 (1H, m), 1.50 (1H, m), 1.31 (1H, m).

^{13}C NMR (*Py-d$_5$*): δ 204.6 (-C=O), 197.4 (-C=O), 186.5 (-C=O), 181.2 (-C=O), 149.3 (-C), 148.1 (-C), 147.7 (-C), 147.2 (-C), 144.0 (-C), 143.2 (-C), 138.1 (-C), 133.2 (-C), 131.7 (-CH), 131.5 (-C), 131.3 (-C), 129.9 (-CH), 128.83 (-CH), 128.79 (-CH), 128.0 (-CH), 127.5 (-C), 126.9 (-C), 125.9 (-CH), 123.7 (-CH), 121.9 (-CH), 121.8 (-CH), 121.7 (-CH), 121.4 (-CH), 117.1 (-CH), 116.3 (-CH), 113.2 (-CH), 112.3 (-CH), 110.8 (-CH), 103.0 (-CH), 79.3 (-C), 75.3 (-C), 73.7 (-CH), 66.0 (-CH), 64.6 (-CH), 56.1 (-OMe), 55.6 (-OMe), 54.8 (-CH), 52.6 (-CH), 51.8 (-CH$_2$), 50.7 (-CH), 48.6 (-CH$_2$), 31.3 (-CH$_2$), 29.5 (-CH$_2$), 28.2 (-CH$_2$), 26.7 (-CH$_2$).

MS [ESI-MS, positive mode]: found *m/z* 804 [M+H]$^+$, 826 [M+Na]$^+$.
HRMS [ESI-MS, positive mode]: MF: $C_{49}H_{45}N_3O_8$; found m/z 826.3113 [M+Na]$^+$ [calcd. 826.3104].

Compound 7B (±):
Color: Obtained as yellowish white solid; **Yield**: 56% (629 mg); **Mp**: 163-165 ºC; **R$_f$** 0.50 (5 % MeOH in $CHCl_3$);
^{1}H NMR (*Py-d$_5$*): δ 12.17 (1H, s, -NH), 8.06 (2H, d, *J*= 7.2 Hz), 7.88 (1H, d, *J*= 8.4 Hz), 7.84 (1H, s), 7.67 (2H, m), 7.62 (1H, t, *J*=7.2 Hz), 7.47 (1H, d, *J*=7.2 Hz), 7.34 (1H, d, *J*=7.8 Hz), 7.30 (1H, s), 7.19 (1H, d, *J*=8.4 Hz), 7.15 (1H, d, *J*=8.4 Hz), 7.03 (1H, s), 6.91 (1H, d, *J*= 8.4 Hz), 6.83 (1H, d, *J*= 7.8 Hz), 5.81 (1H, s), 4.52 (1H, d, *J*= 11.4 Hz), 4.30 (1H, d, *J*= 12.0 Hz), 4.23 (1H, m), 4.09 (1H, m), 3.88 (3H, s), 3.74 (2H, m), 3.66 (3H, s), 2.62 (2H, m), 2.52 (1H, m), 2.35 (1H, m), 1.84 (2H, m), 1.70 (5H, m), 1.58 (1H, m).

^{13}C NMR (*Py-d$_5$*): δ 205.1 (-C=O), 191.4 (-C=O), 189.4 (-C=O), 179.9 (-C=O), 148.8 (-C), 148.6 (-C), 147.1 (-C), 147.0 (-C), 142.9 (-C), 142.2 (-C), 138.2 (-CH), 135.0 (-CH), 132.1 (-C), 131.8 (-CH), 130.75 (-C), 130.67 (-C), 130.45 (-C), 128.9 (-C), 128.2 (-CH), 128.1 (-CH), 125.3 (-CH), 123.6 (-C), 122.9 (-CH), 121.6 (-CH), 120.4 (-CH), 120.3 (-CH), 116.7 (-CH), 116.4 (-CH), 112.4 (-CH), 111.5 (-CH), 111.1 (-CH), 100.7 (-CH), 84.2 (-C), 77.5 (-C), 74.1 (-C), 73.5 (-CH), 73.0 (-CH), 64.5 (-CH), 62.9 (-CH), 55.7 (-OMe), 55.4 (-OMe), 52.9 (-CH), 51.4 (-CH), 48.2 (-CH$_2$), 47.7 (-CH$_2$), 30.7 (-CH$_2$), 30.4 (-CH$_2$), 27.7 (-CH$_2$), 27.0 (-CH$_2$).

MS [ESI-MS, positive mode]: found *m/z* 930 [M+H]$^+$.
HRMS [ESI-MS, positive mode]: MF: $C_{49}H_{45}IN_3O_8$; found m/z 930.2252 [M+H]$^+$ [calcd. 930.2251].

Compound 7C (±):

Color: Obtained as white solid; **Yield**: 50% (607 mg); **Mp**: 204-206 ºC; $\mathbf{R_f}$ 0.49 (5 % MeOH in $CHCl_3$);

^{1}H NMR (*Py-d$_5$*): δ 11.78 (1H, s, -NH), 8.08 (1H, dd, *J*=2.4, 6.6 Hz), 8.05 (1H, dd, *J*=2.4, 8.4 Hz), 7.83 (1H, dd, *J*=2.4, 8.4 Hz), 7.74 (1H, m), 7.62 (2H, m), 7.47 (1H, dd, *J*=1.8, 7.8 Hz), 7.41 (1H, dd, *J*=1.8, 7.8 Hz), 7.31 (1H, s), 7.26 (1H, d, *J*=8.4 Hz), 7.18 (2H, m), 7.12 (1H, d, *J*=7.8 Hz), 6.96 (1H, d, *J*=7.8 Hz), 6.91 (2H, m), 5.86 (1H, s), 4.46 (1H, m), 4.32 (2H, m), 4.11 (1H, m), 3.87 (3H, s), 3.82 (3H, s), 3.76 (2H, m), 3.49 (1H, m), 3.24 (1H, d, *J*=10.8 Hz), 3.20 (1H, d, *J*=11.4 Hz), 3.02 (1H, m), 2.57 (2H, m), 1.86 (1H, m), 1.71 (2H, m), 1.64 (1H, m).

^{13}C NMR (*Py-d$_5$*): δ 205.0 (-C=O), 190.9 (-C=O), 190.5 (-C=O), 180.9 (-C=O), 149.8 (-C), 149.6 (-C), 148.3 (-C), 147.9 (-C), 143.9 (-C), 143.4 (-C), 133.2 (-C), 132.51 (-CH), 132.47 (-C), 131.2 (-C), 131.0 (-C), 130.3 (-C), 129.7 (-CH), 128.7 (-CH), 128.6 (-CH), 126.8 (-CH), 126.4 (-C), 126.2 (-CH), 125.1 (-CH), 122.4 (-CH), 121.9 (-CH), 121.7 (-CH), 121.5 (-CH), 117.5 (-CH), 117.3 (-CH), 111.9 (-CH), 111.3 (-CH), 110.6 (-CH), 100.9 (-CH), 78.7 (-C), 77.4 (-CH), 74.4 (-C), 73.8 (-CH), 63.8 (-CH), 61.2 (-CH), 56.3 (-OMe), 56.2 (-OMe), 55.9 (-CH_2), 53.0 (-CH), 52.4 (-CH), 48.4 (-CH_2), 37.2 (-CH_2), 31.3 (-CH_2), 27.9 (-CH_2).

MS [ESI-MS, positive mode]: found *m/z* 822 $[M+H]^+$, 844 $[M+Na]^+$.

HRMS [ESI-MS, positive mode]: MF: $C_{48}H_{43}N_3O_8S$; found m/z 844.2670 $[M+Na]^+$ [calcd. 844.2669].

<u>Compound 7D (±):</u>

Color: Obtained as yellowish white solid; **Yield**: 49% (588 mg); **Mp**: 160-162 ºC; $\mathbf{R_f}$ 0.48 (5 % MeOH in $CHCl_3$);

^{1}H NMR (*Py-d$_5$*): δ 8.05 (2H, m), 7.87 (1H, d, *J*=8.4 Hz), 7.76 (1H, d, *J*= 8.4 Hz), 7.65 (1H, t, *J*= 7.8 Hz), 7.60 (1H, d, *J*=7.2 Hz), 7.39 (1H, d, *J*=7.8 Hz), 7.31 (1H, d, *J*=1.2 Hz), 7.27 (1H, d, *J*=8.4 Hz), 7.20 (1H, m), 7.15 (1H, s), 7.07 (2H, m), 7.01 (1H, d, *J*= 7.8 Hz), 6.96 (1H, dd, *J*= 1.2, 7.8 Hz), 5.88 (1H, s), 4.46 (1H, d, *J*= 12.6 Hz), 4.33 (2H, m), 4.12 (1H, m), 3.88 (3H, s), 3.76 (2H, m), 3.73 (3H, s), 3.70 (1H, m), 3.24 (1H, d, *J*=10.8 Hz), 3.12 (1H, d, *J*=11.4 Hz), 3.00 (1H, m), 2.72 (1H, m), 2.63 (1H, m), 2.13 (3H, s), 1.87 (1H, m), 1.77 (2H, m), 1.68 (1H, m).

^{13}C NMR (*Py-d$_5$*): δ 204.5 (-C=O), 192.0 (-C=O), 188.1 (-C=O), 180.4 (-C=O), 149.0 (-C), 148.7 (-C), 147.6 (-C), 147.1 (-C), 142.8 (-C), 140.8 (-C), 132.8 (-C), 131.9 (-CH), 131.8 (-

C), 130.8 (-C), 130.5 (-C), 130.3 (-C), 129.8 (-CH, -C), 128.1 (-CH), 128.0 (-CH), 127.3 (-CH), 126.1 (-C), 125.5 (-CH), 124.3 (-CH), 121.8 (-CH), 120.9 (-CH), 120.6 (-CH), 116.8 (-CH), 116.5 (-CH), 111.4 (-CH), 110.8 (-CH), 109.9 (-CH), 100.4 (-CH), 78.2 (-C), 77.1 (-CH), 74.1 (-C), 73.1 (-CH), 64.1 (-CH), 60.5 (-CH), 55.6 (-OMe), 55.5 (-OMe), 55.2 (-CH_2), 51.8 (-CH), 51.6 (-CH), 47.7 (-CH_2), 36.8 (-CH_2), 30.8 (-CH_2), 27.7 (-CH_2), 20.8 (-CH_3).

MS [ESI-MS, positive mode]: found *m/z* 836 $[M+H]^+$.

HRMS [ESI-MS, positive mode]: MF: $C_{49}H_{46}N_3O_8S$; found m/z 836.3009 $[M+H]^+$ [calcd. 836.3006].

1H NMR in Py-d5

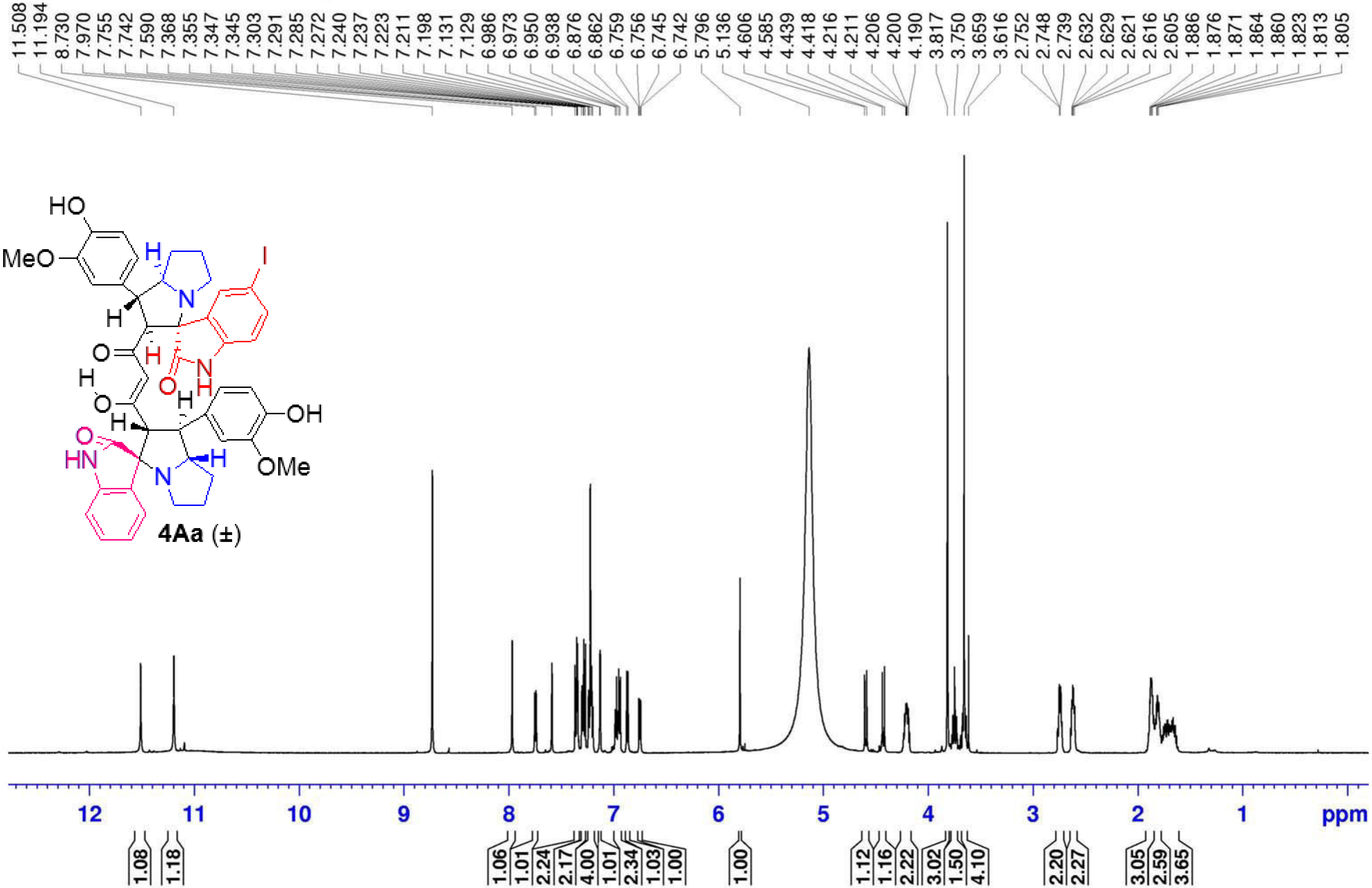

13C-NMR in Py-d5

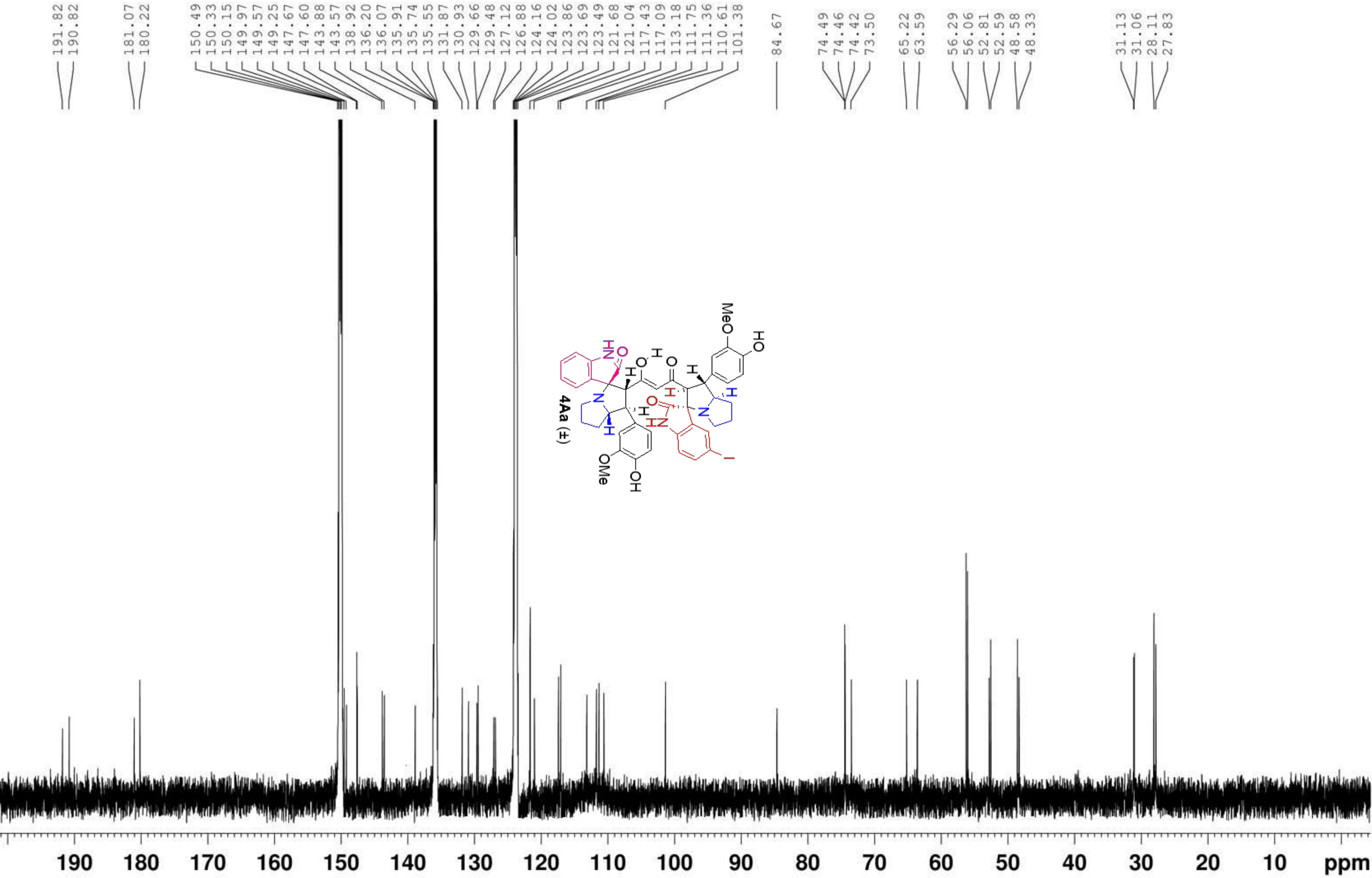

4Aa (±)

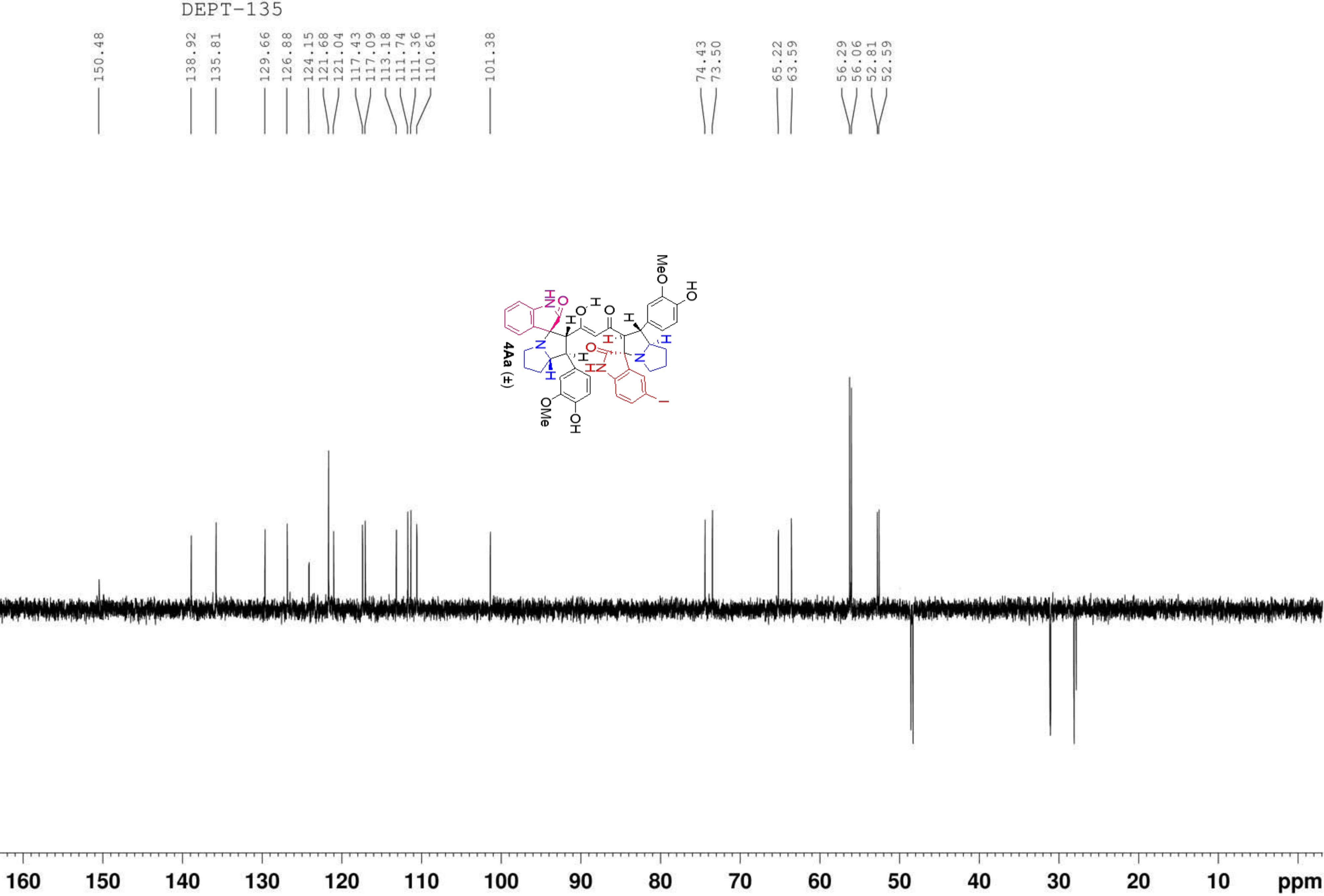
DEPT-135
150.48
138.92
135.81
129.66
126.88
124.15
121.68
121.04
117.43
117.09
113.18
111.74
111.36
110.61
101.38
74.43
73.50
65.22
63.59
56.29
56.06
52.81
52.59
MeO
HO
OH
OMe
4Aa (±)
160
150
140
130
120
110
100
90
80
70
60
50
40
30
20
10
ppm

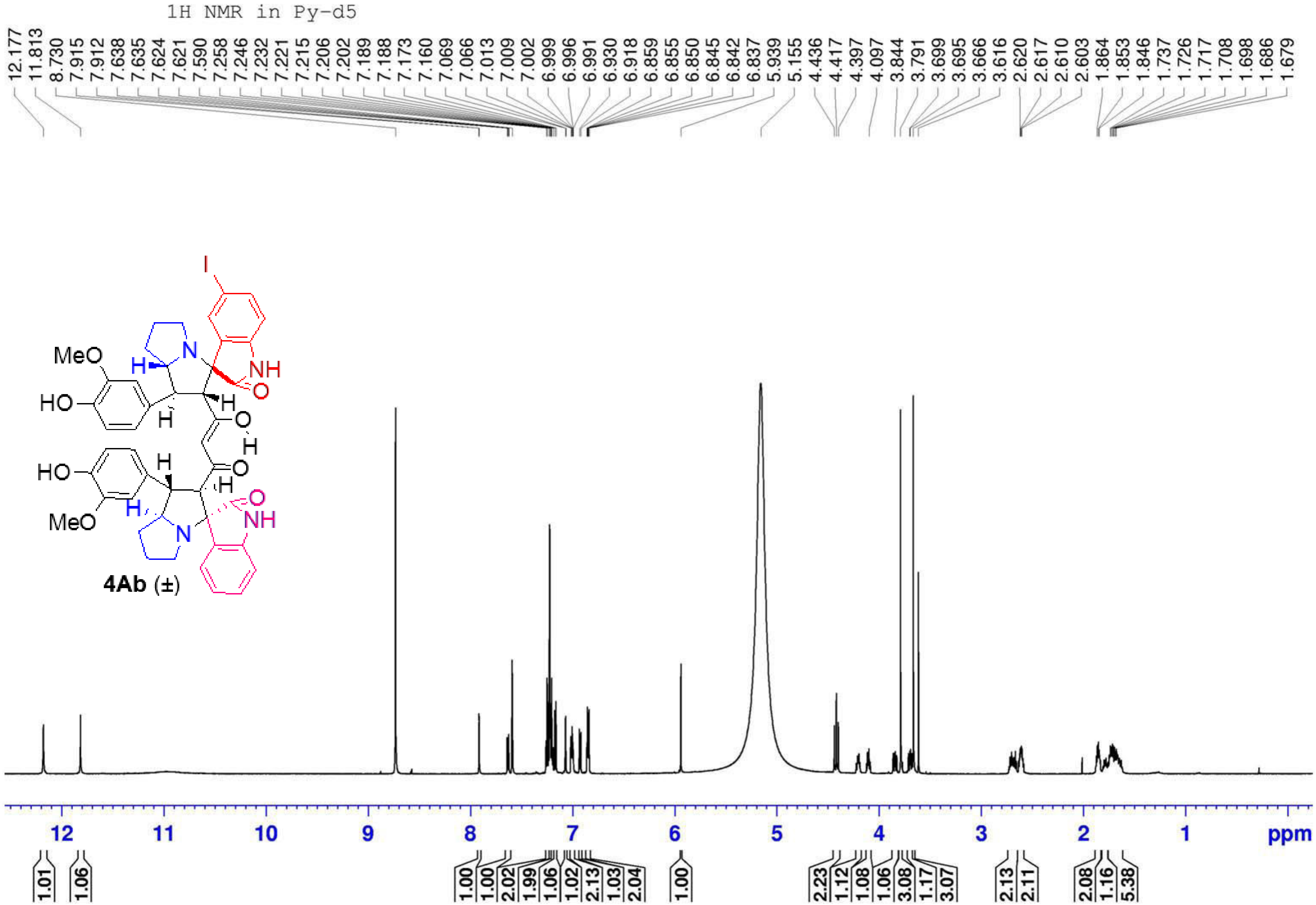
1H NMR in Py-d5
12.177
11.813
8.730
7.915
7.912
7.638
7.635
7.624
7.621
7.590
7.258
7.246
7.232
7.221
7.215
7.206
7.202
7.189
7.188
7.173
7.160
7.069
7.066
7.013
7.009
7.002
6.999
6.996
6.991
6.930
6.918
6.859
6.855
6.850
6.845
6.842
6.837
5.939
5.155
4.436
4.417
4.397
4.097
3.844
3.791
3.699
3.695
3.666
3.616
2.620
2.617
2.610
2.603
1.864
1.853
1.846
1.737
1.726
1.717
1.708
1.698
1.686
1.679
I
MeO
HO
H
N
NH
O
H
H
O
H
HO
H
O
H
O
MeO
H
NH
N
4Ab (±)
12
11
10
9
8
7
6
5
4
3
2
1
ppm
1.01
1.06
1.00
1.00
2.02
1.99
1.06
1.02
2.13
1.03
2.04
1.00
2.23
1.12
1.08
1.06
3.08
1.17
3.07
2.13
2.11
2.08
1.16
5.38

13C-NMR in Py-d5

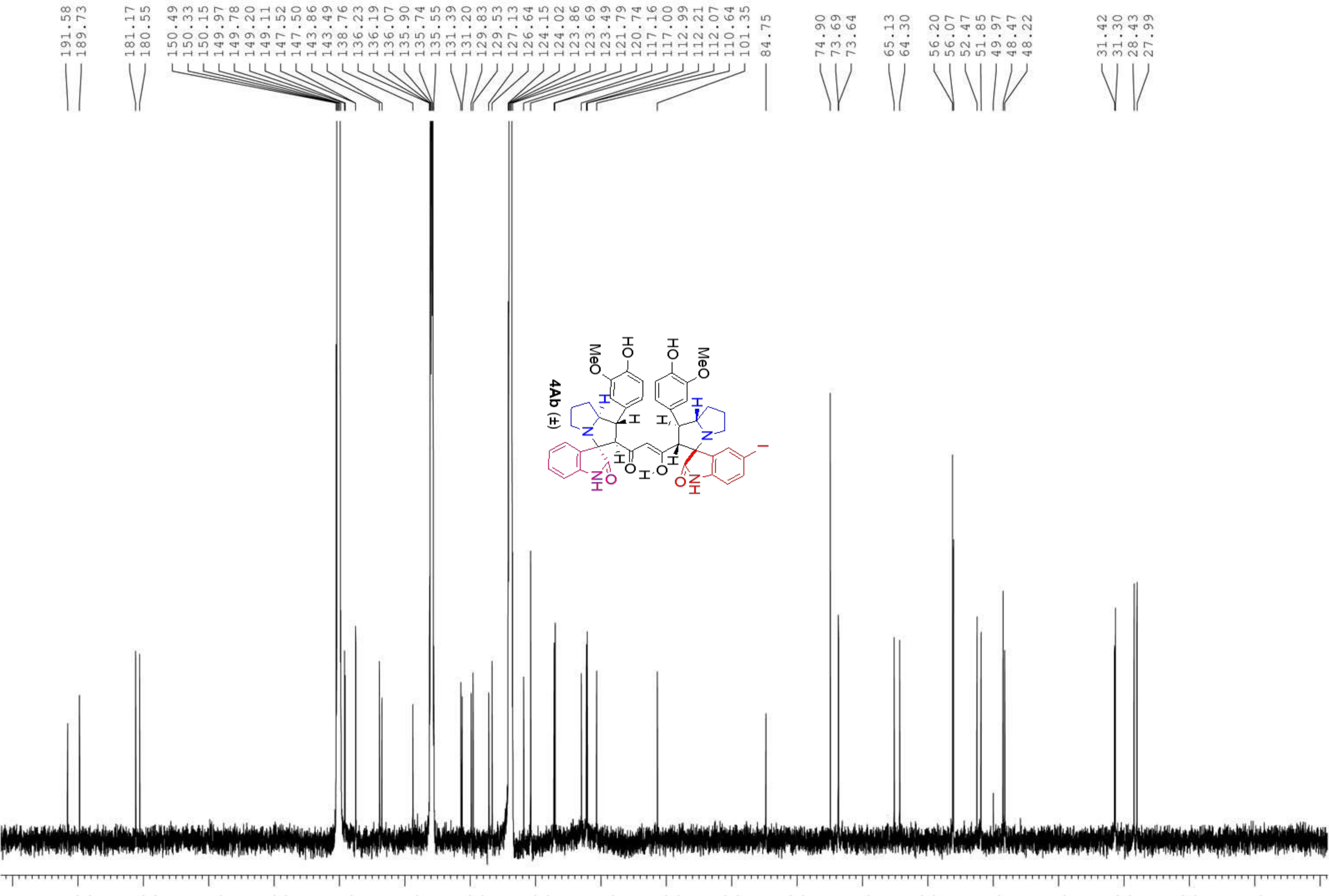

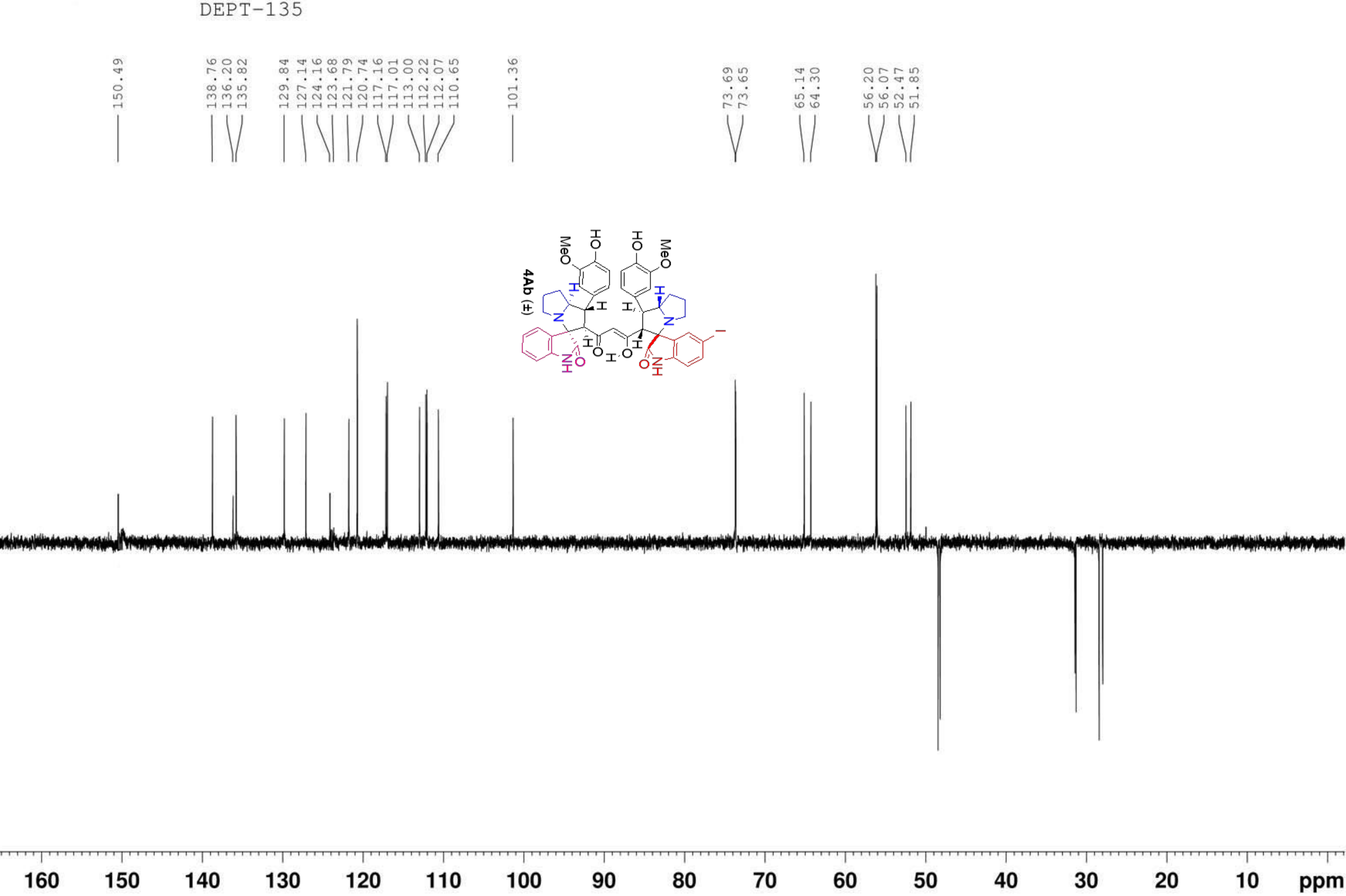

DEPT-135
150.49
138.76
136.20
135.82
129.84
127.14
124.16
123.68
121.79
120.74
117.16
117.01
113.00
112.22
112.07
110.65
101.36
73.69
73.65
65.14
64.30
56.20
56.07
52.47
51.85
MeO
HO
HO
MeO
H
N
NH
O
I
4Ab (±)
160
150
140
130
120
110
100
90
80
70
60
50
40
30
20
10
ppm

1H NMR in Py-d5

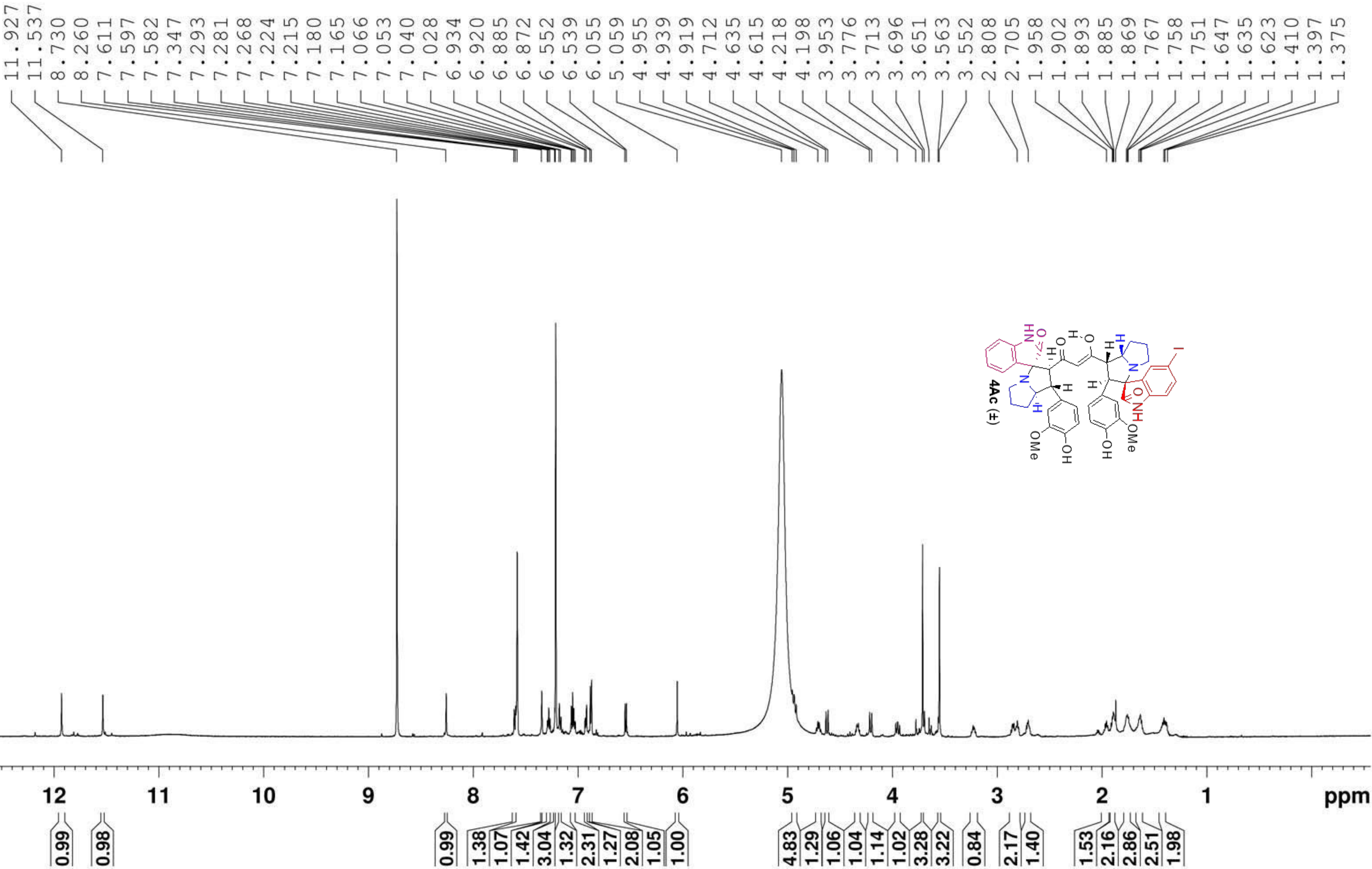

13C-NMR in Py-d5

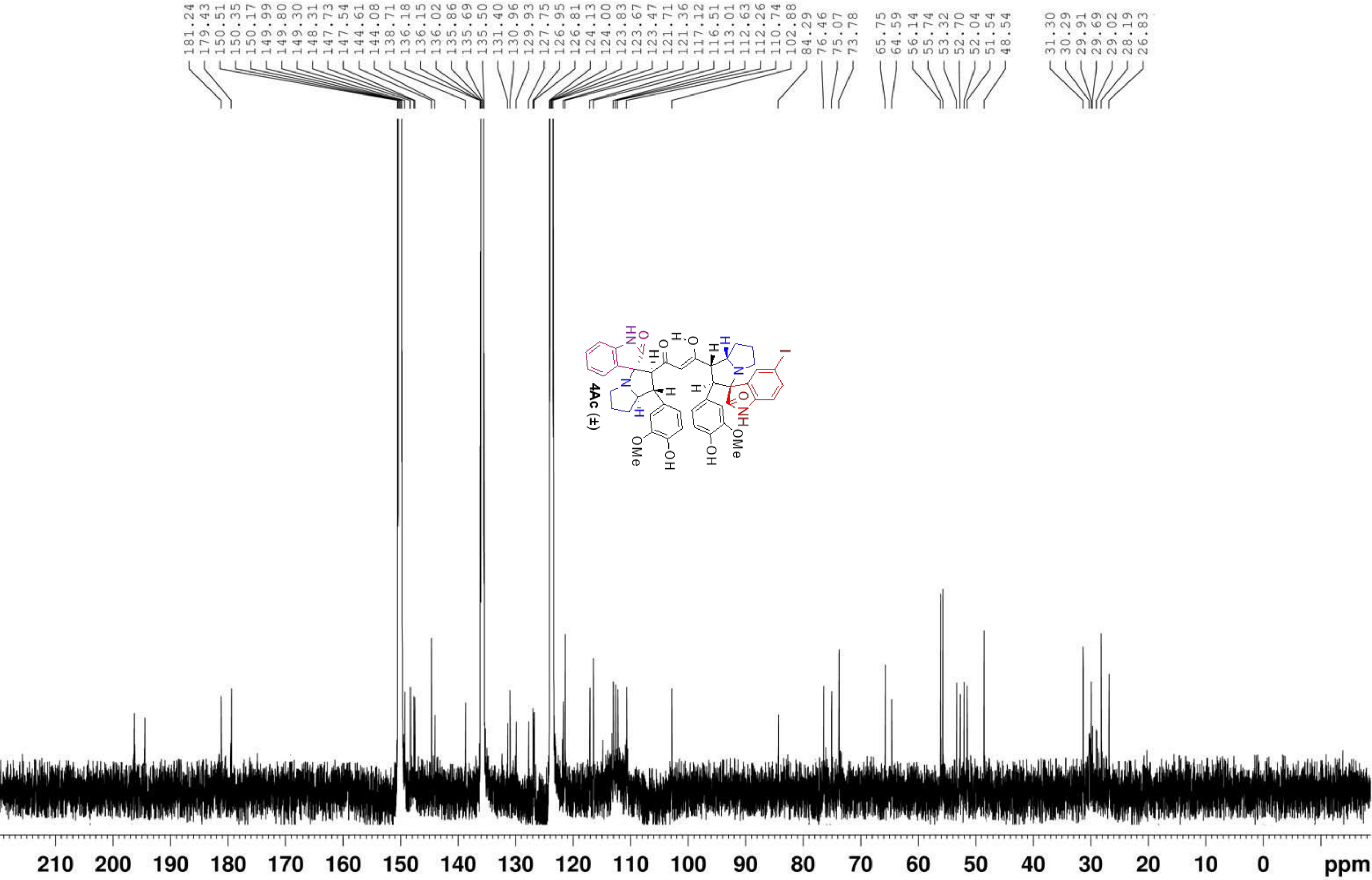

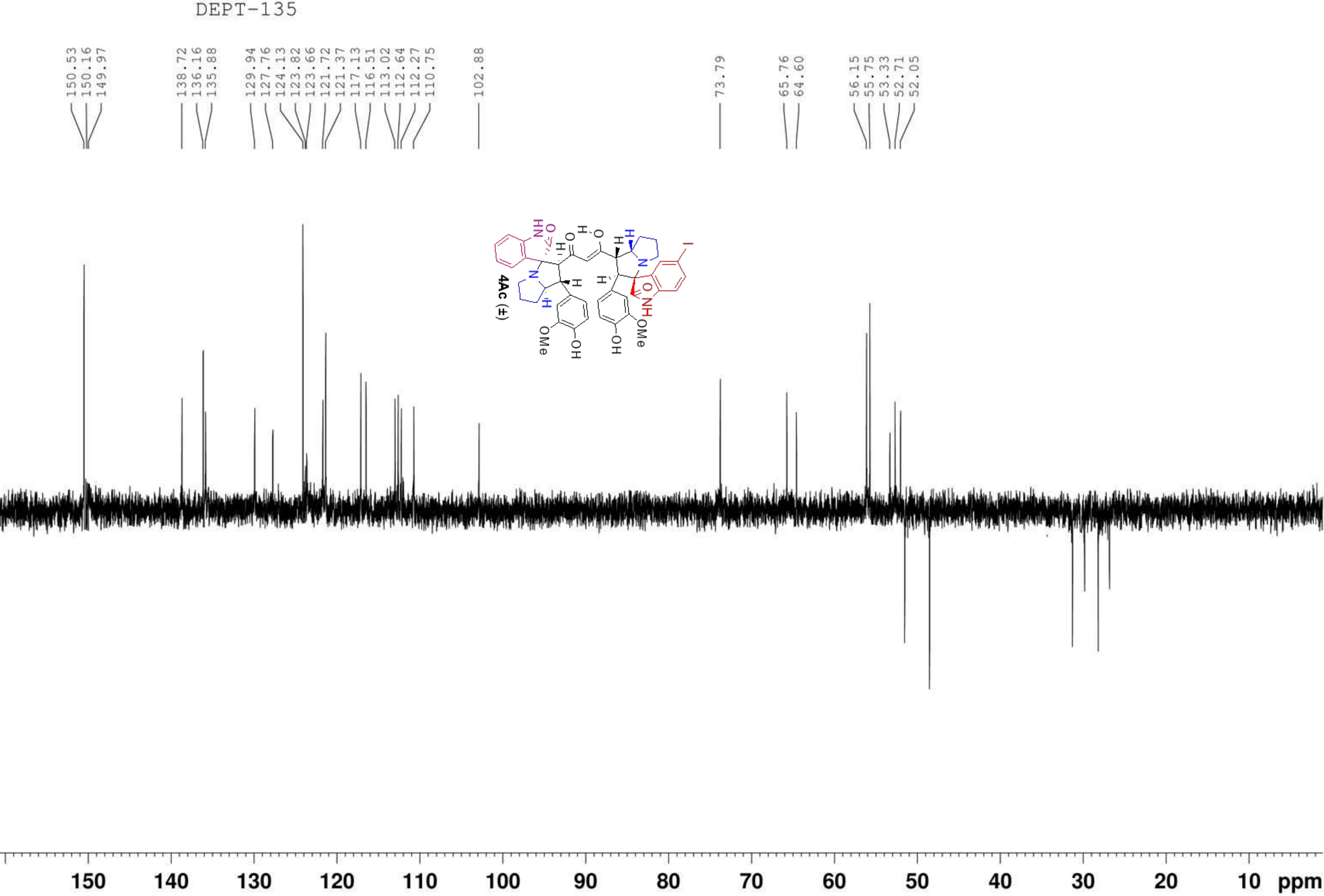

DEPT-135
150.53
150.16
149.97
138.72
136.16
135.88
129.94
127.76
124.13
123.82
123.66
121.72
121.37
117.13
116.51
113.02
112.64
112.27
110.75
102.88
73.79
65.76
64.60
56.15
55.75
53.33
52.71
52.05
4Ac (±)
150
140
130
120
110
100
90
80
70
60
50
40
30
20
10
ppm

1H NMR in Py-d5

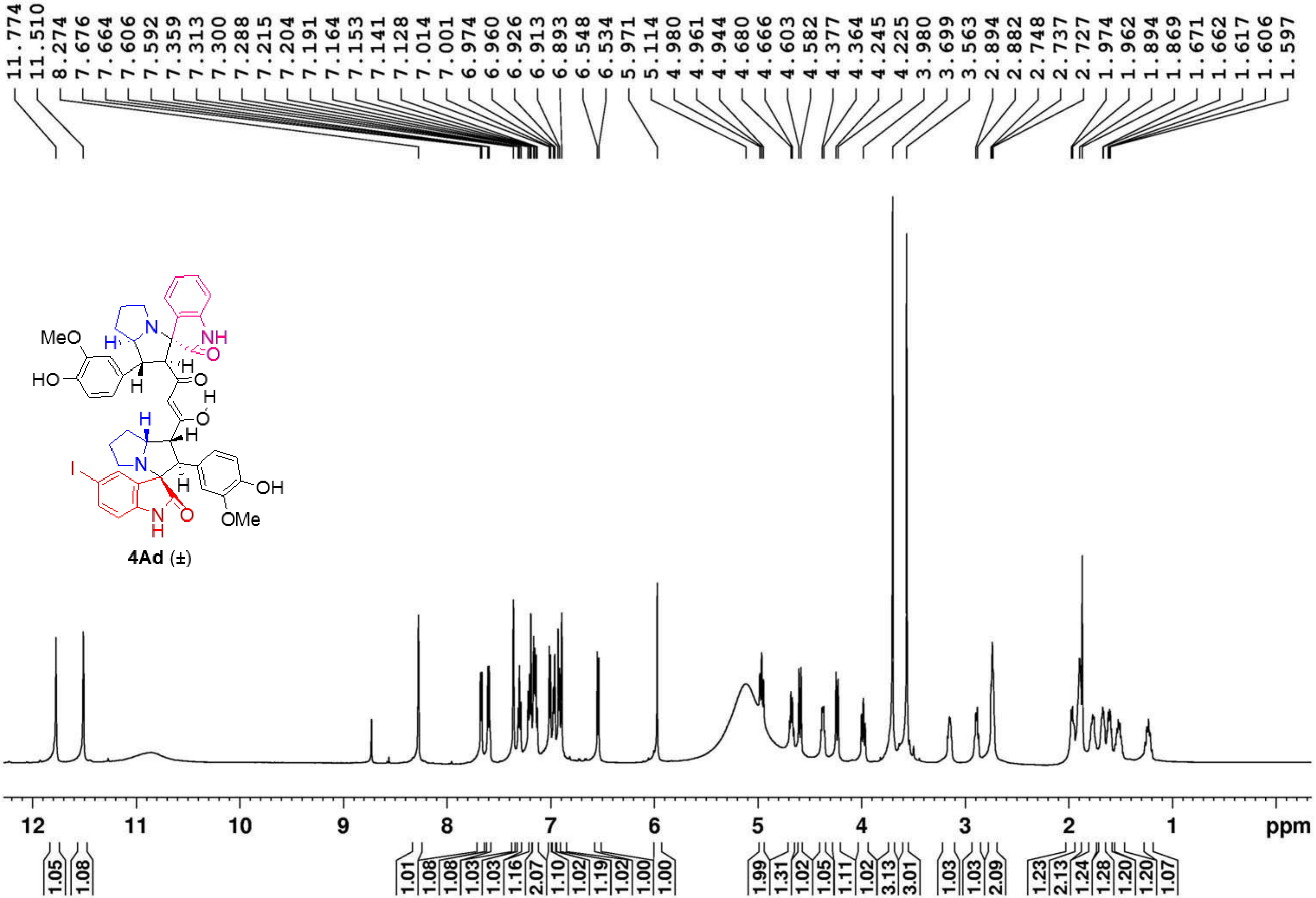

13C-NMR in Py-d5

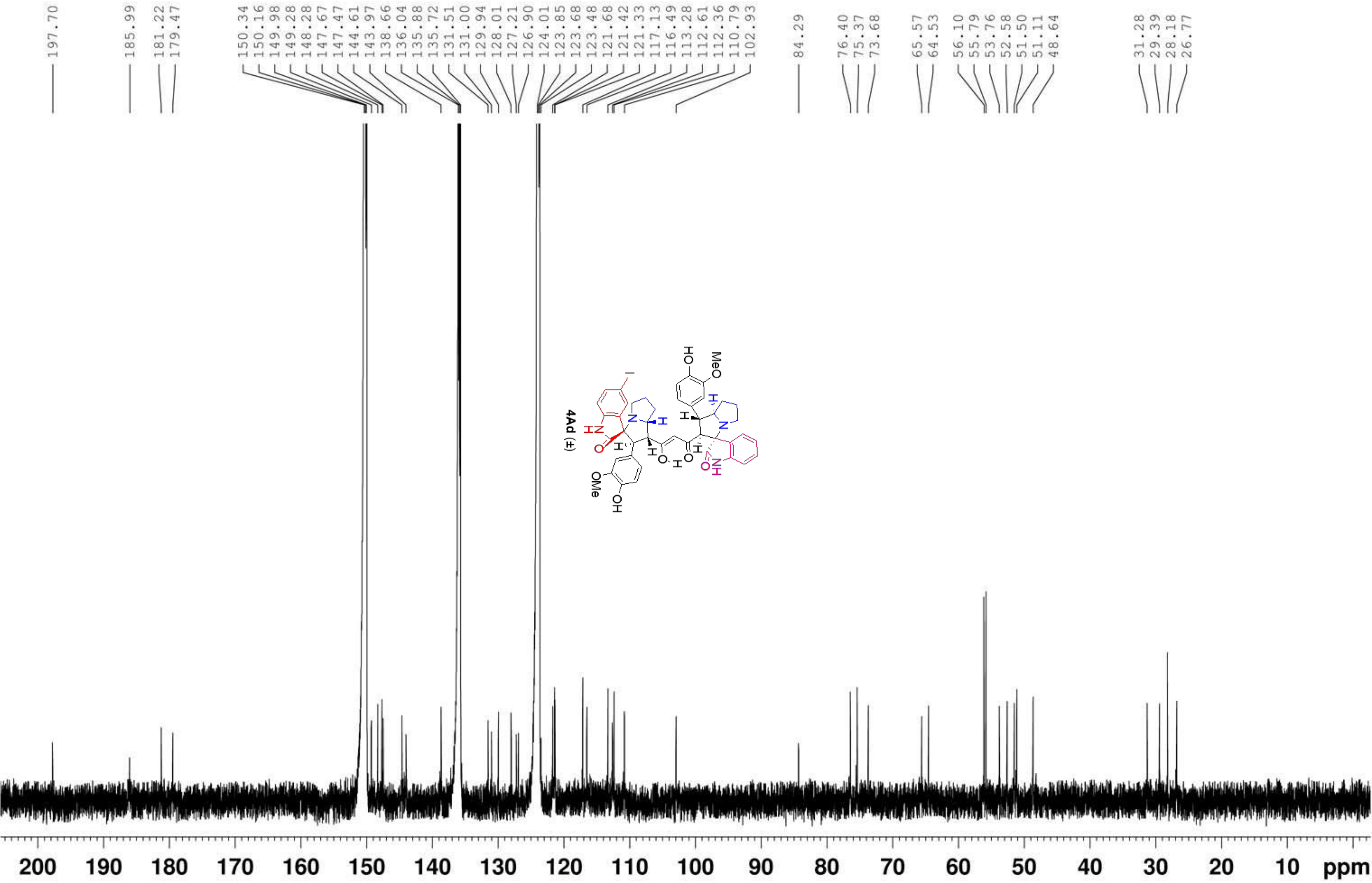

DEPT-135

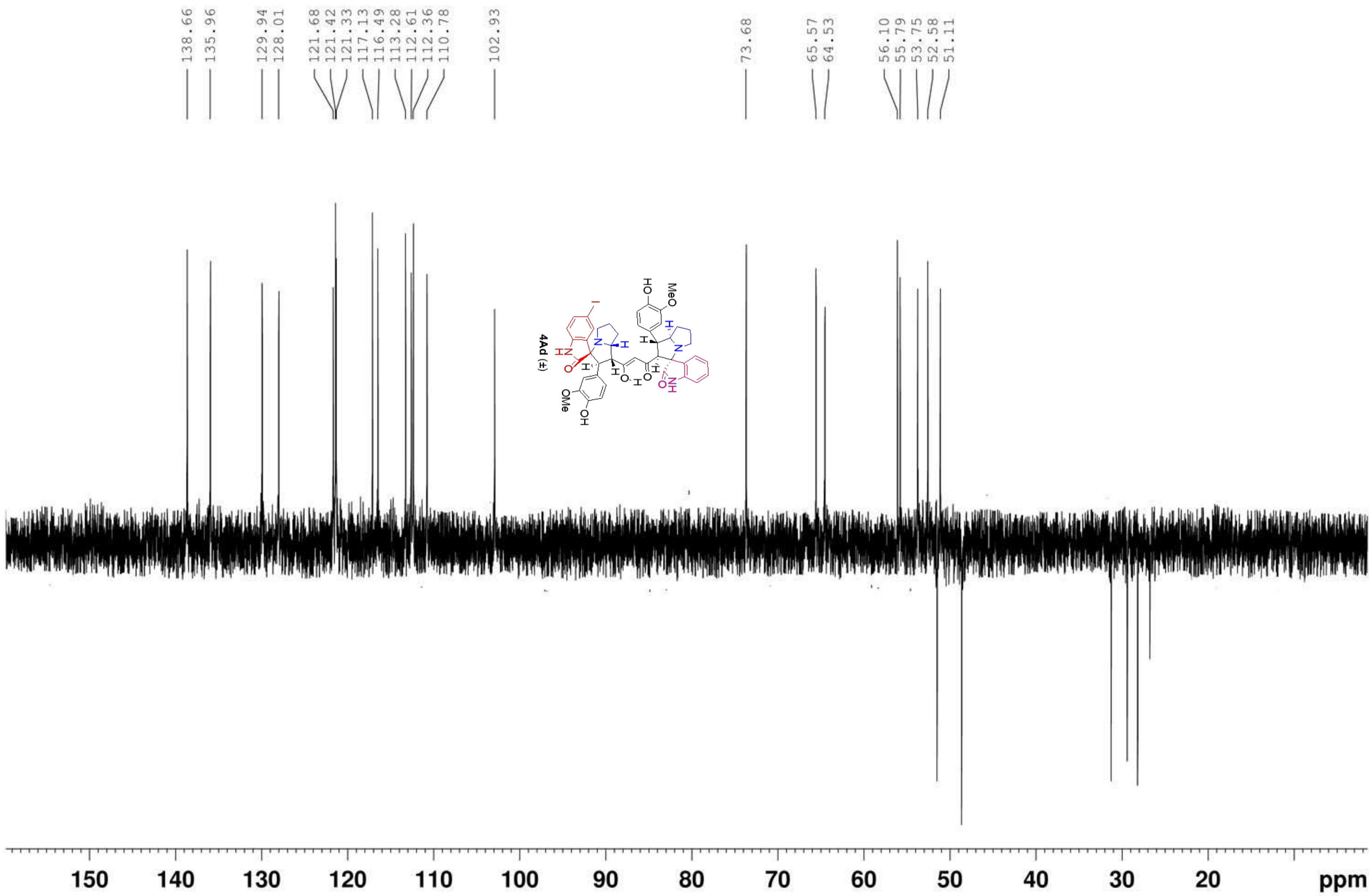
138.66
135.96
129.94
128.01
121.68
121.42
121.33
117.13
116.49
113.28
112.61
112.36
110.78
102.93
73.68
65.57
64.53
56.10
55.79
53.75
52.58
51.11
MeO
HO
NH
O
H
N
I
OH
OMe
4Ad (±)
150
140
130
120
110
100
90
80
70
60
50
40
30
20
ppm

1H NMR in Py-d5

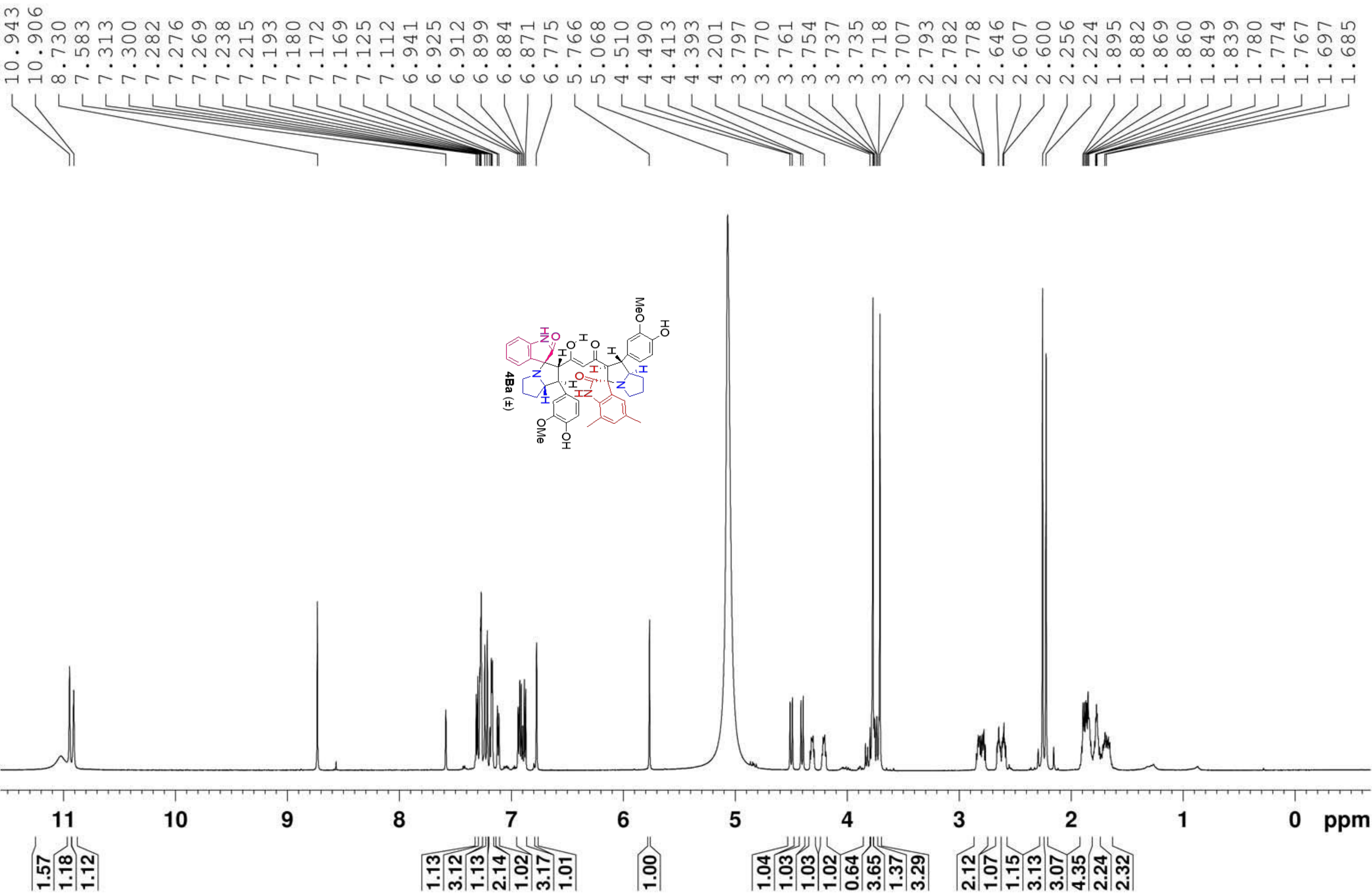

13C-NMR in Py-d5

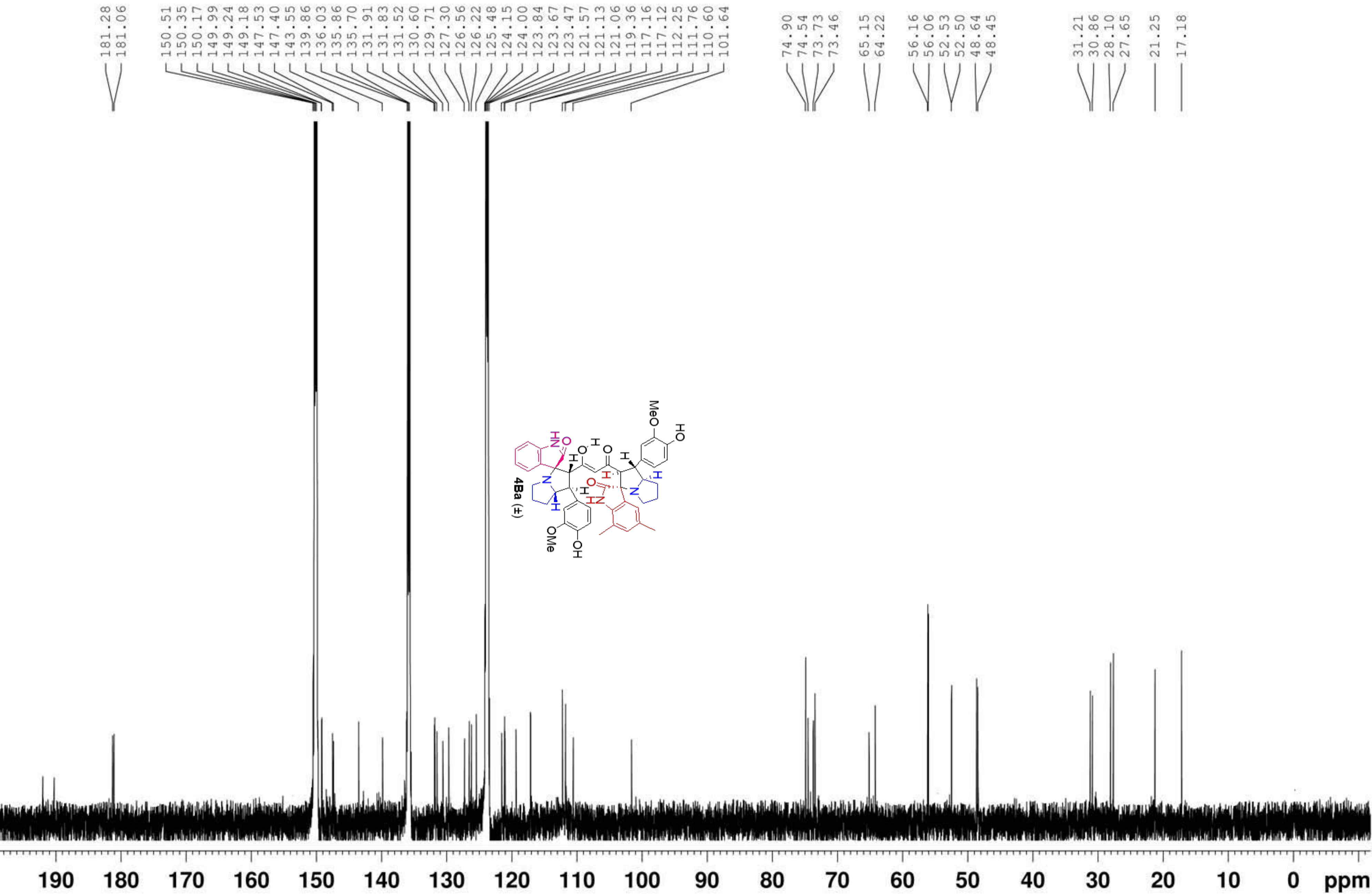

DEPT-135

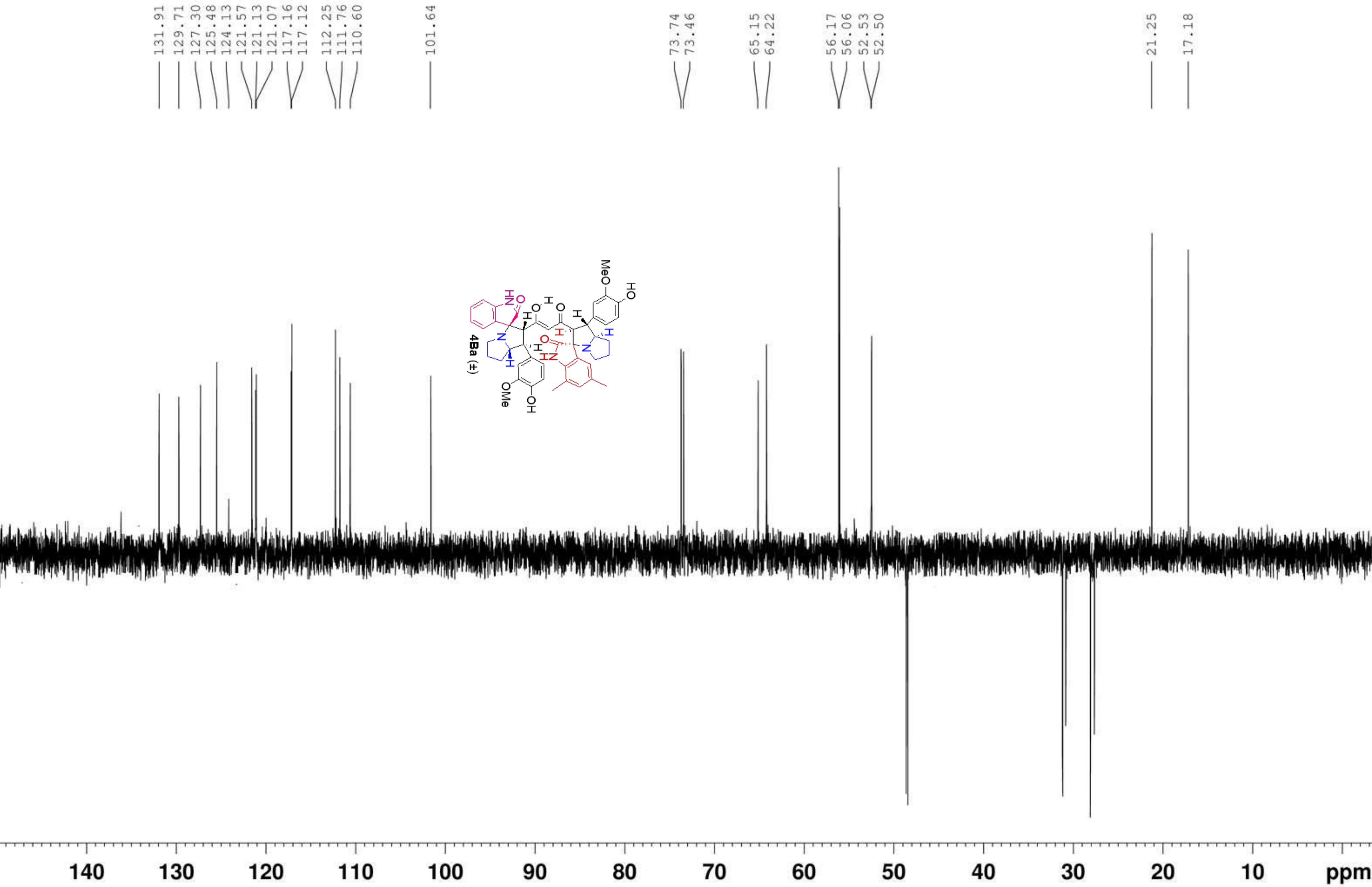

131.91
129.71
127.30
125.48
124.13
121.57
121.13
121.07
117.16
117.12
112.25
111.76
110.60
101.64
73.74
73.46
65.15
64.22
56.17
56.06
52.53
52.50
21.25
17.18
MeO
HO
HN
O
H
N
OMe
OH
4Ba (±)
140
130
120
110
100
90
80
70
60
50
40
30
20
10
ppm

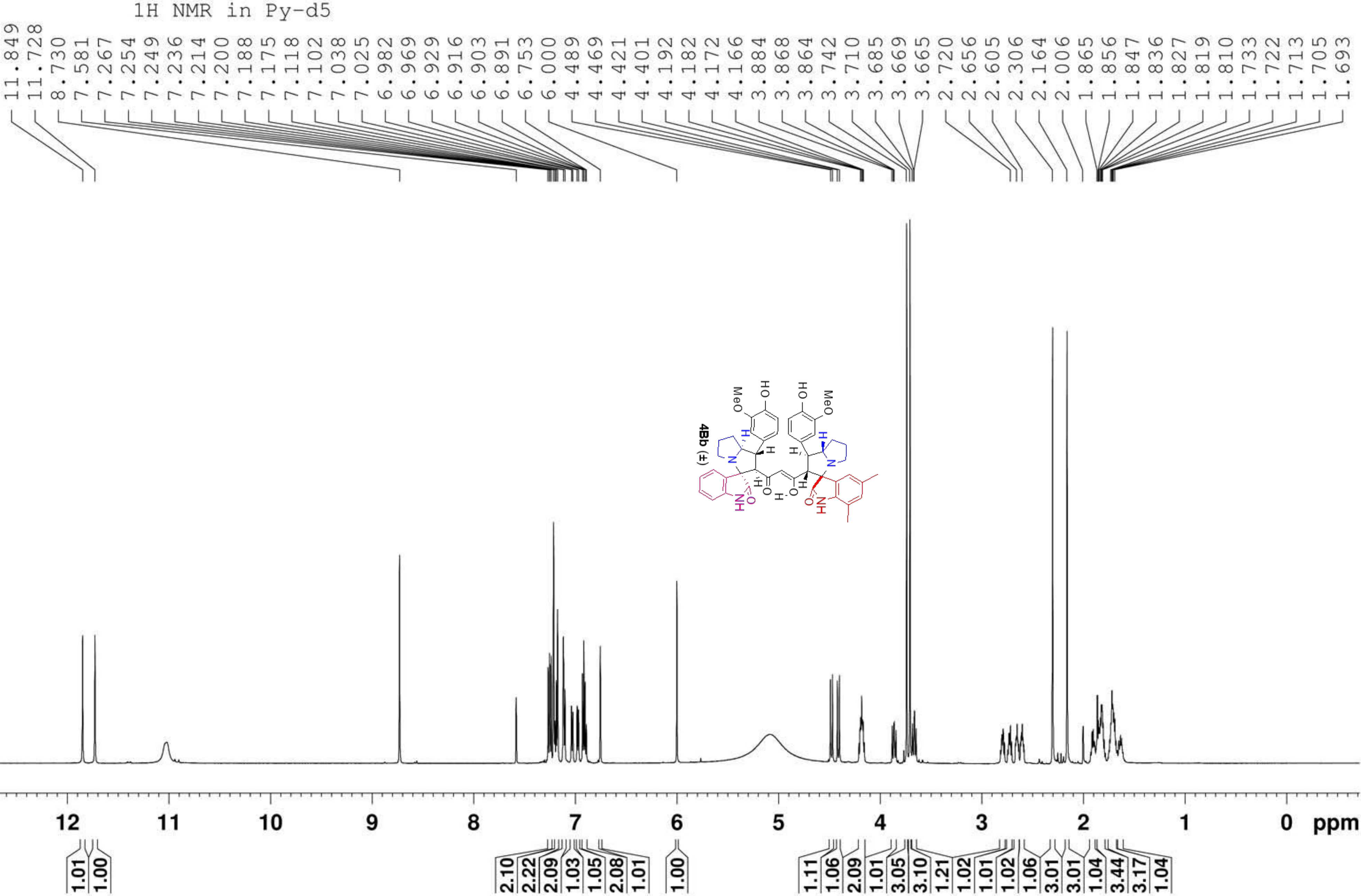
1H NMR in Py-d5
11.849
11.728
8.730
7.581
7.267
7.254
7.249
7.236
7.214
7.200
7.188
7.175
7.118
7.102
7.038
7.025
6.982
6.969
6.929
6.916
6.903
6.891
6.753
6.000
4.489
4.469
4.421
4.401
4.192
4.182
4.172
4.166
3.884
3.868
3.864
3.742
3.710
3.685
3.669
3.665
2.720
2.656
2.605
2.306
2.164
2.006
1.865
1.856
1.847
1.836
1.827
1.819
1.810
1.733
1.722
1.713
1.705
1.693
MeO
HO
NH
4Bb (±)
12
11
10
9
8
7
6
5
4
3
2
1
0
ppm
1.01
1.00
2.10
2.22
2.09
1.03
1.05
2.08
1.01
1.00
1.11
1.06
2.09
1.01
3.05
3.10
1.21
1.02
1.01
1.02
1.06
3.01
3.01
1.04
3.44
3.17
1.04

13C-NMR in Py-d5

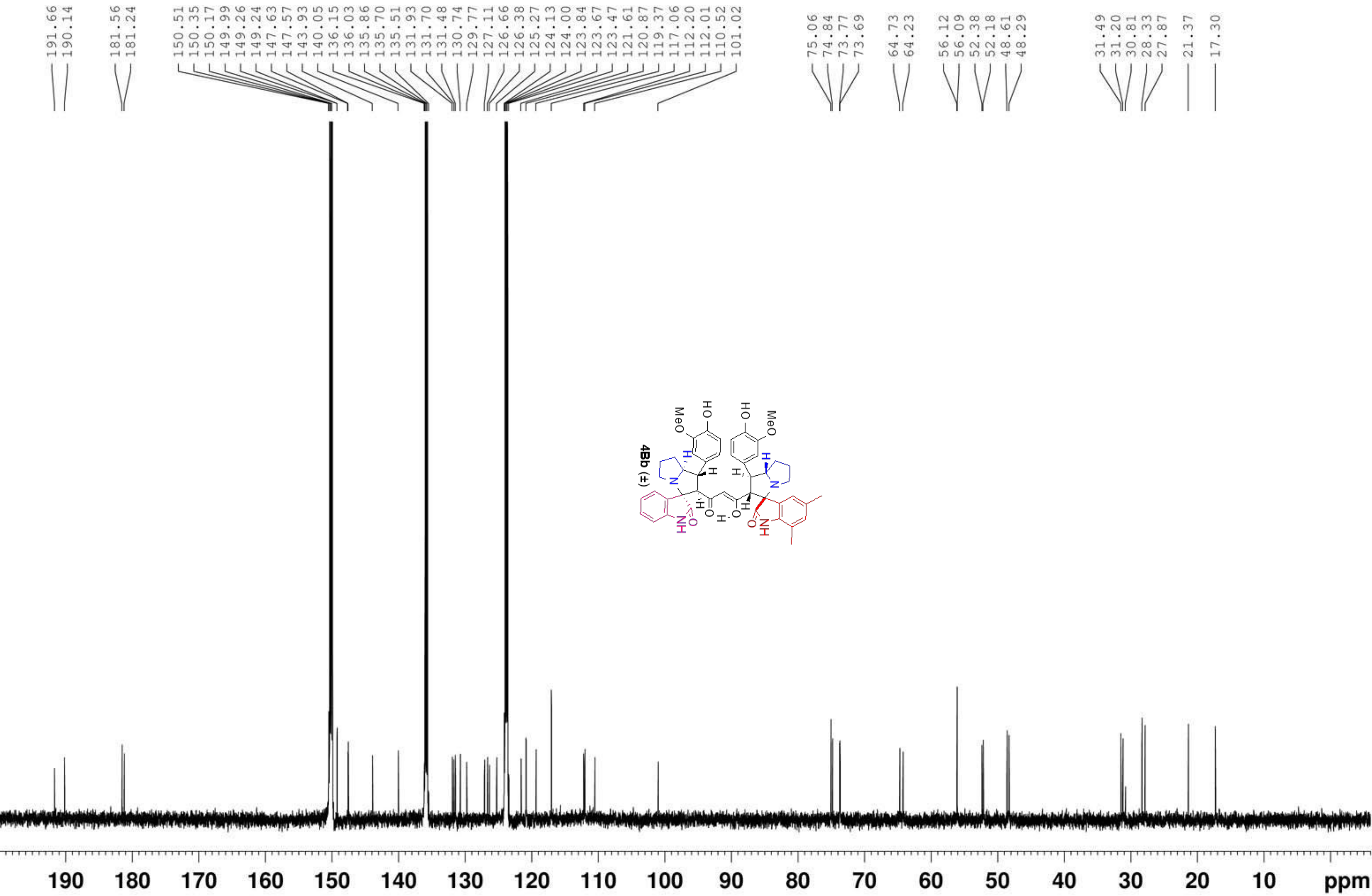

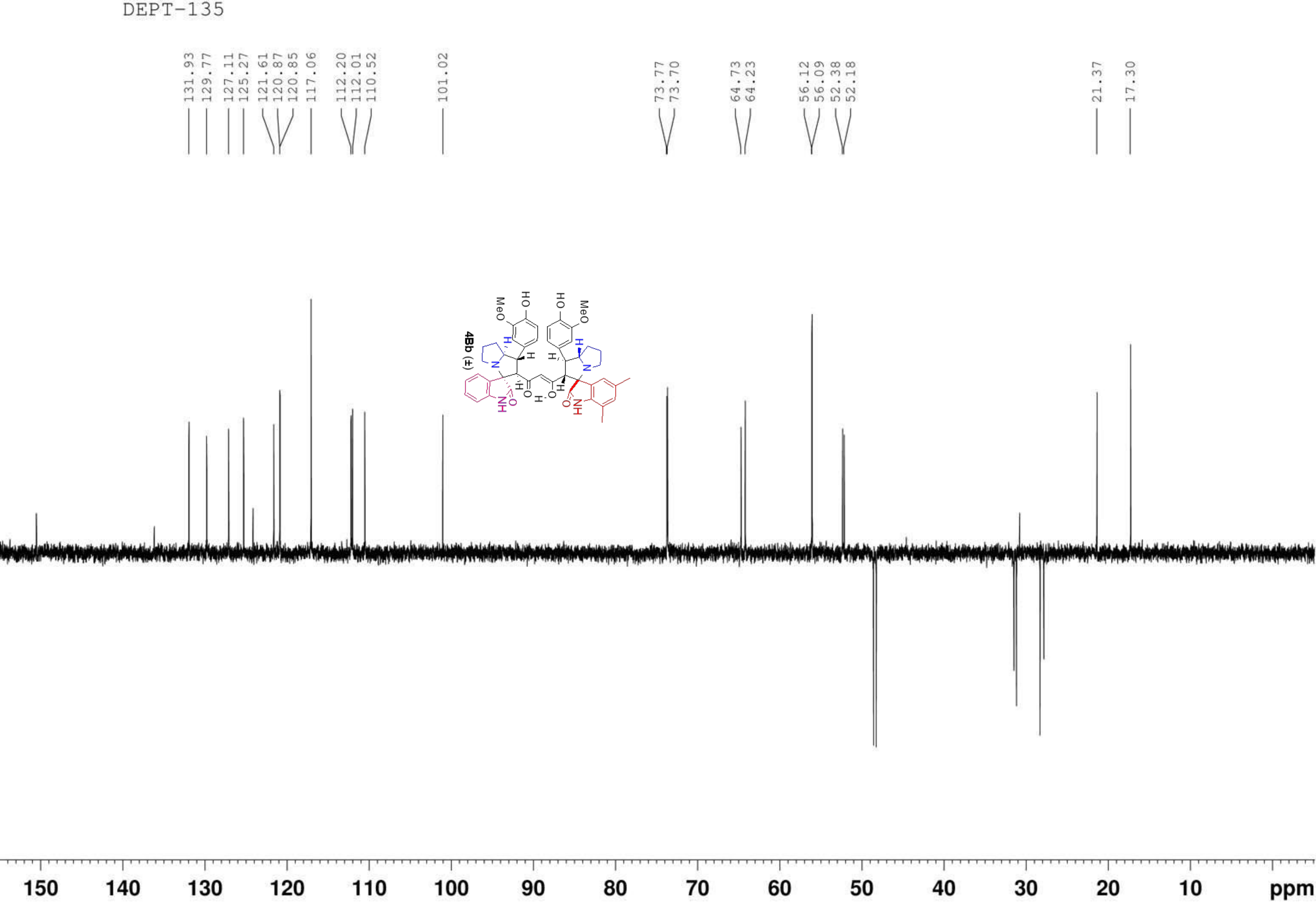
DEPT-135
131.93
129.77
127.11
125.27
121.61
120.87
120.85
117.06
112.20
112.01
110.52
101.02
73.77
73.70
64.73
64.23
56.12
56.09
52.38
52.18
21.37
17.30
MeO
HO
HO
MeO
NH
O
NH
O
4Bb (±)
150
140
130
120
110
100
90
80
70
60
50
40
30
20
10
ppm

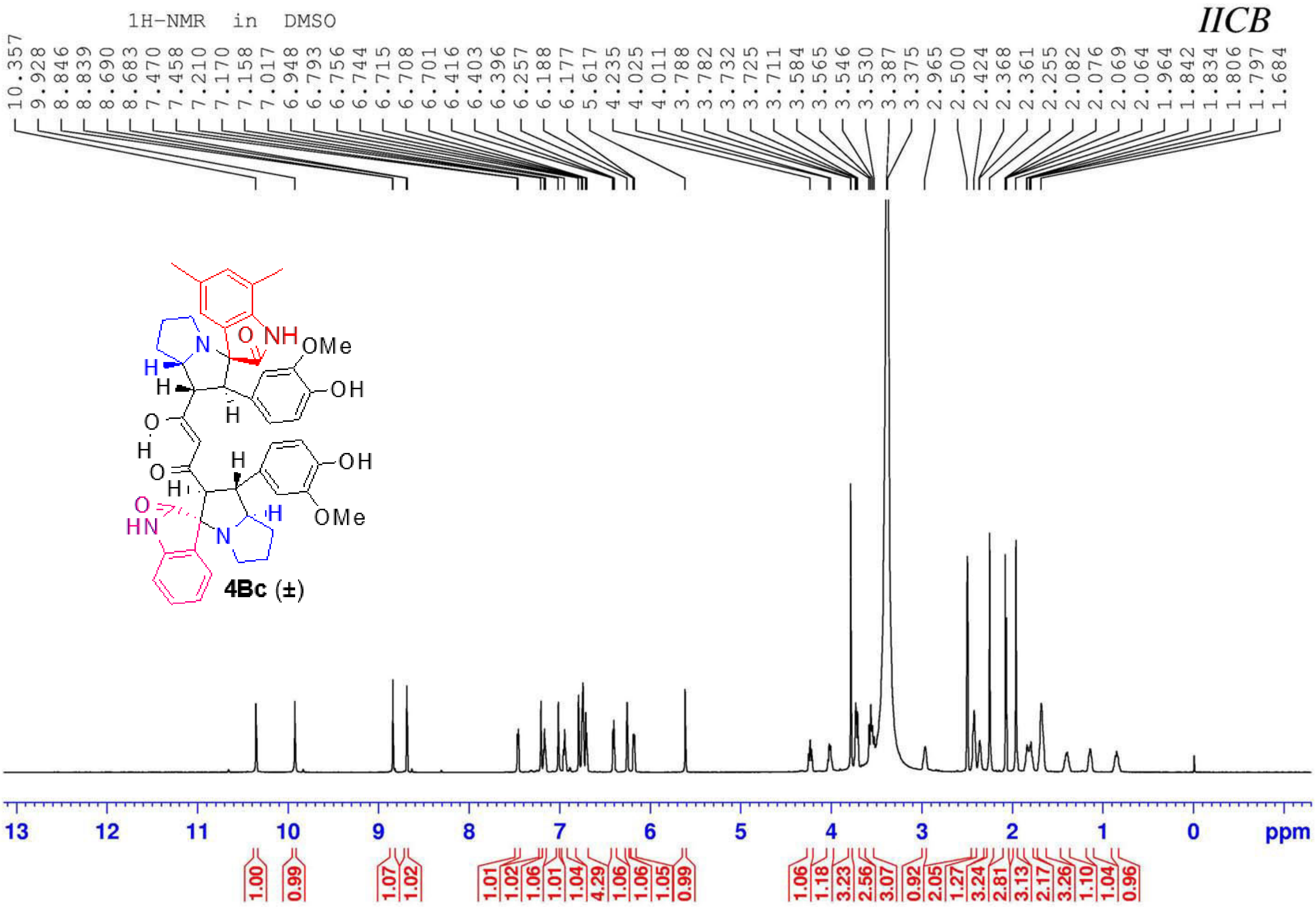
1H-NMR in DMSO
IICB
10.357 9.928 8.846 8.839 8.690 8.683 7.470 7.458 7.210 7.170 7.158 7.017 6.948 6.793 6.756 6.744 6.715 6.708 6.701 6.416 6.403 6.396 6.257 6.188 6.177 5.617 4.235 4.025 4.011 3.788 3.782 3.732 3.725 3.711 3.584 3.565 3.546 3.530 3.387 3.375 2.965 2.500 2.424 2.368 2.361 2.255 2.082 2.076 2.069 2.064 1.964 1.842 1.834 1.806 1.797 1.684
O
NH
OMe
N
H
H
OH
H
O
H
O
H
OH
H
O
HN
N
H
OMe
4Bc (±)
13 12 11 10 9 8 7 6 5 4 3 2 1 0 ppm
1.00 0.99 1.07 1.02 1.01 1.02 1.06 1.01 1.04 4.29 1.06 1.06 1.05 0.99 1.06 1.18 3.23 2.56 3.07 0.92 2.05 1.27 3.24 2.81 3.13 2.17 3.26 1.10 1.04 0.96

13C-NMR in DMSO

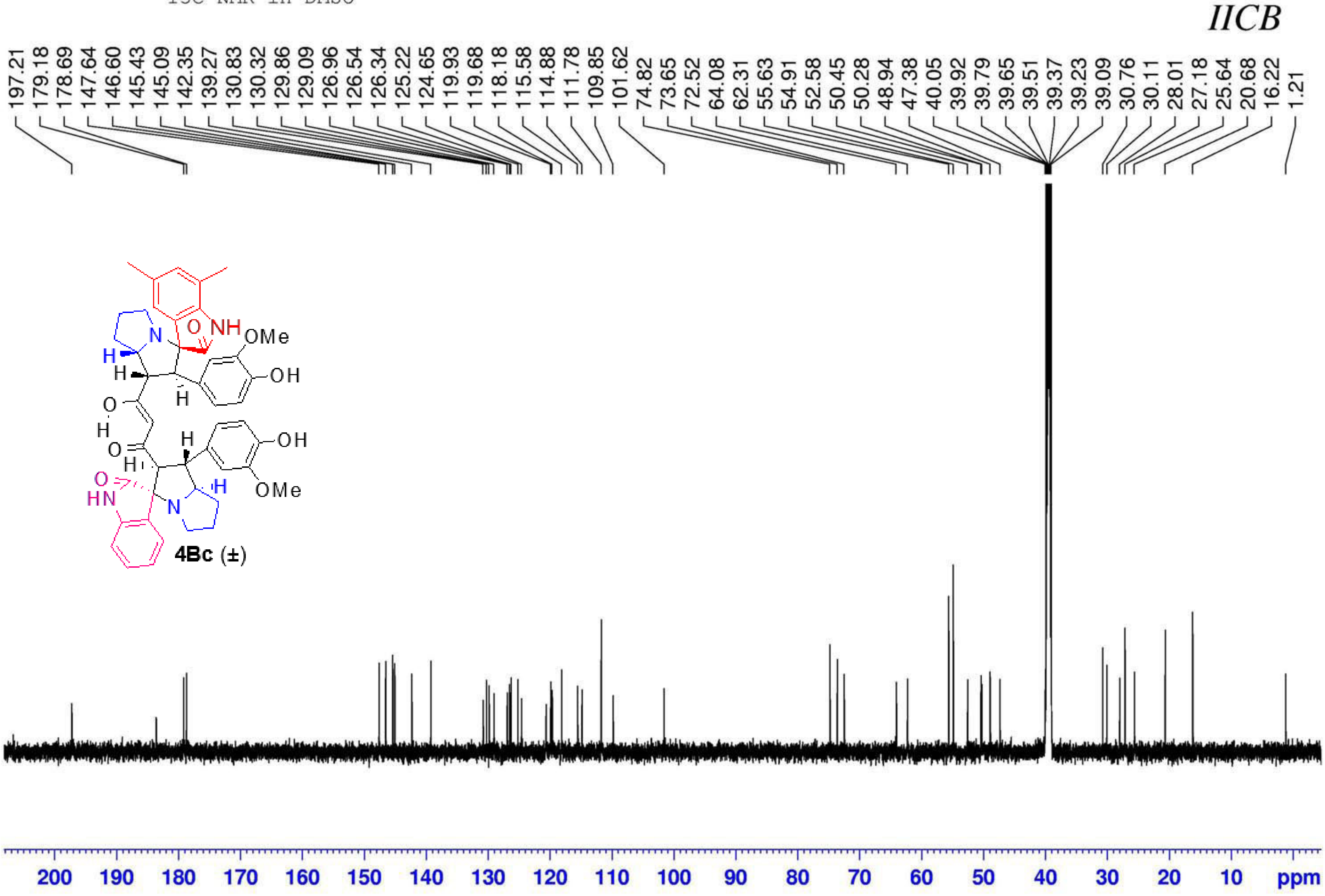

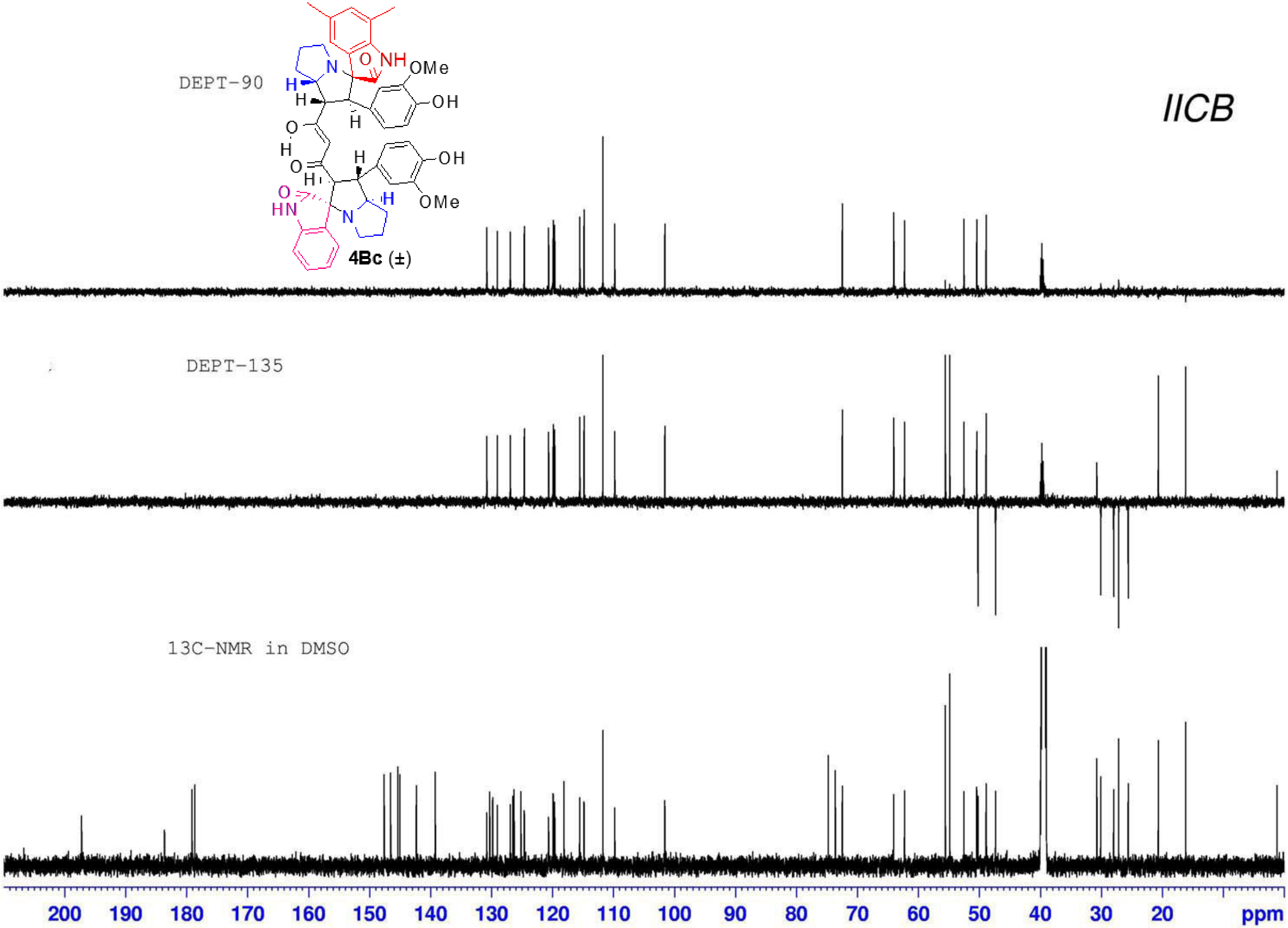
IICB
DEPT-90
DEPT-135
13C-NMR in DMSO
OMe
OH
OH
OMe
NH
HN
4Bc (±)
200
190
180
170
160
150
140
130
120
110
100
90
80
70
60
50
40
30
20
ppm

1H NMR in Py-d5

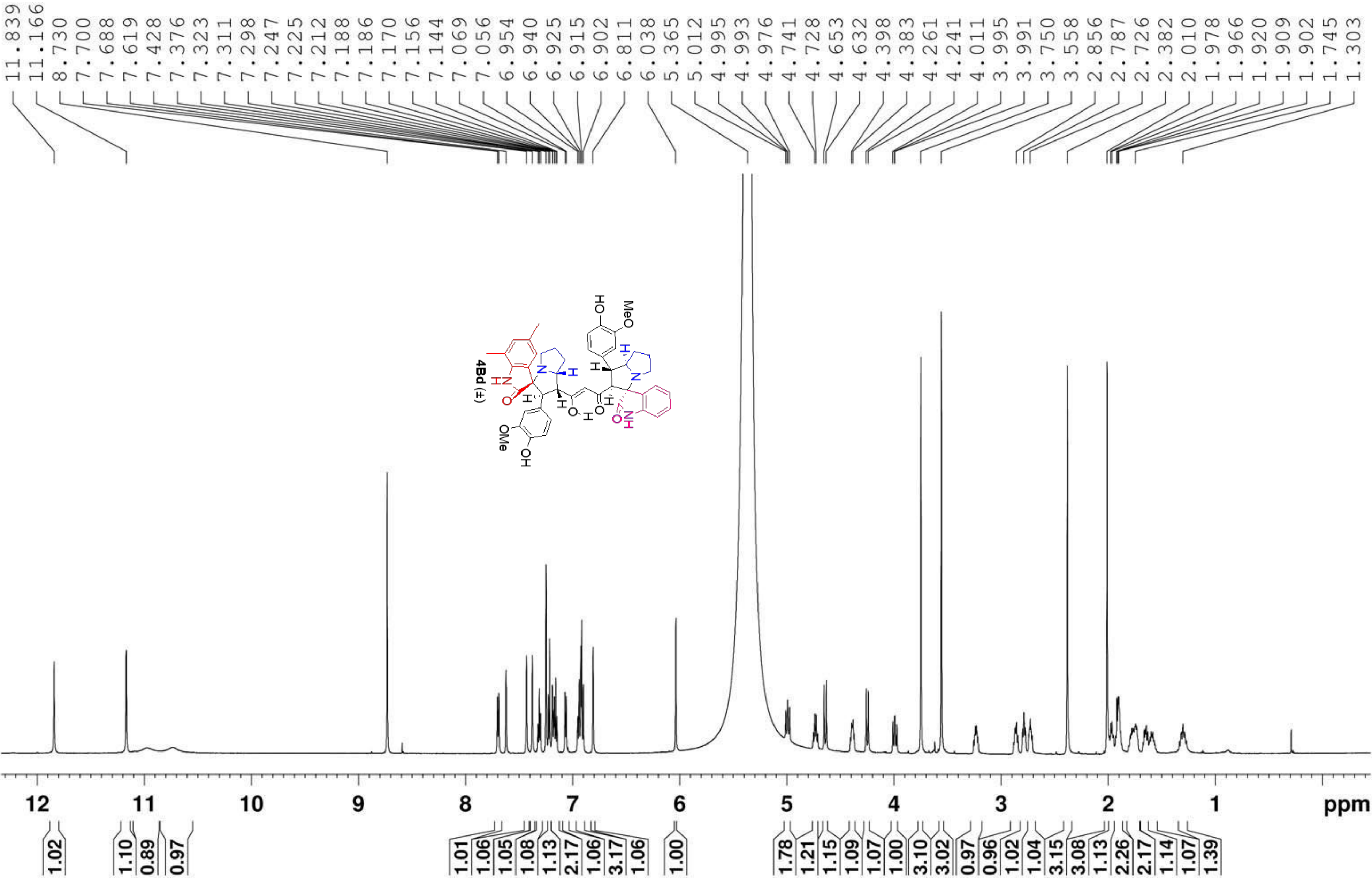

13C-NMR in Py-d5

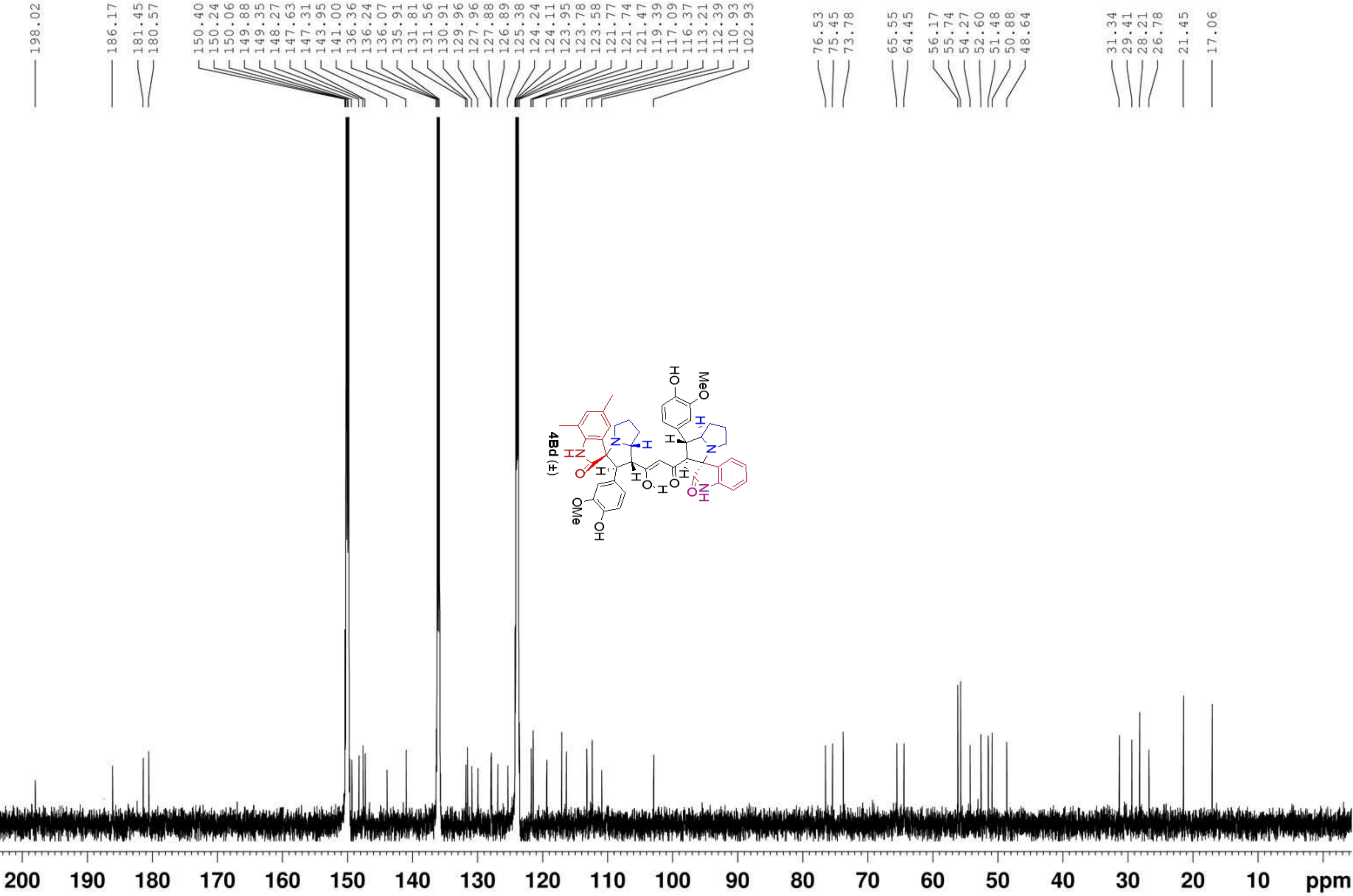

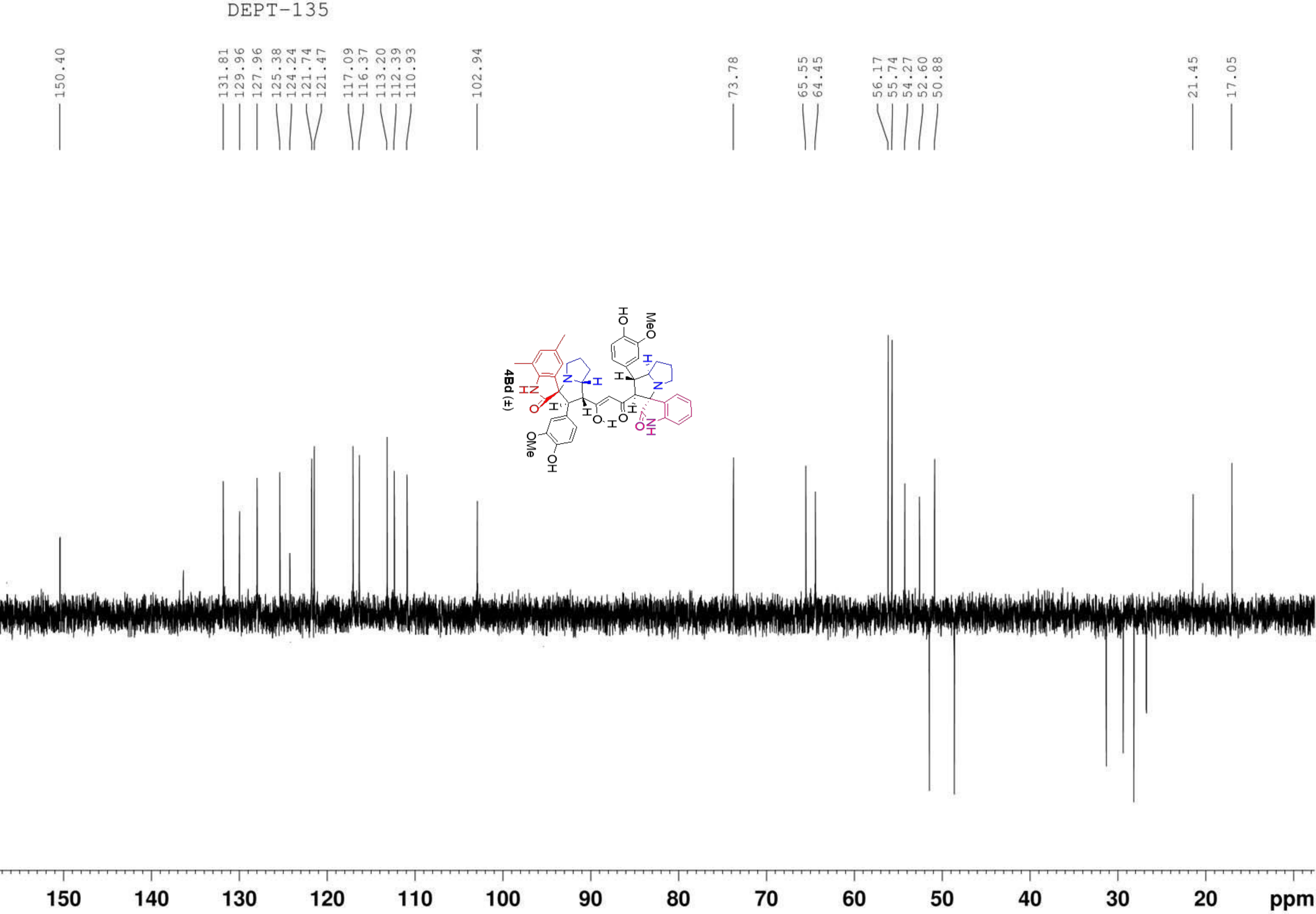
DEPT-135
150.40
131.81
129.96
127.96
125.38
124.24
121.74
121.47
117.09
116.37
113.20
112.39
110.93
102.94
73.78
65.55
64.45
56.17
55.74
54.27
52.60
50.88
21.45
17.05
MeO
HO
OMe
OH
4Bd (±)
150 140 130 120 110 100 90 80 70 60 50 40 30 20 ppm

1H NMR in Py-d5

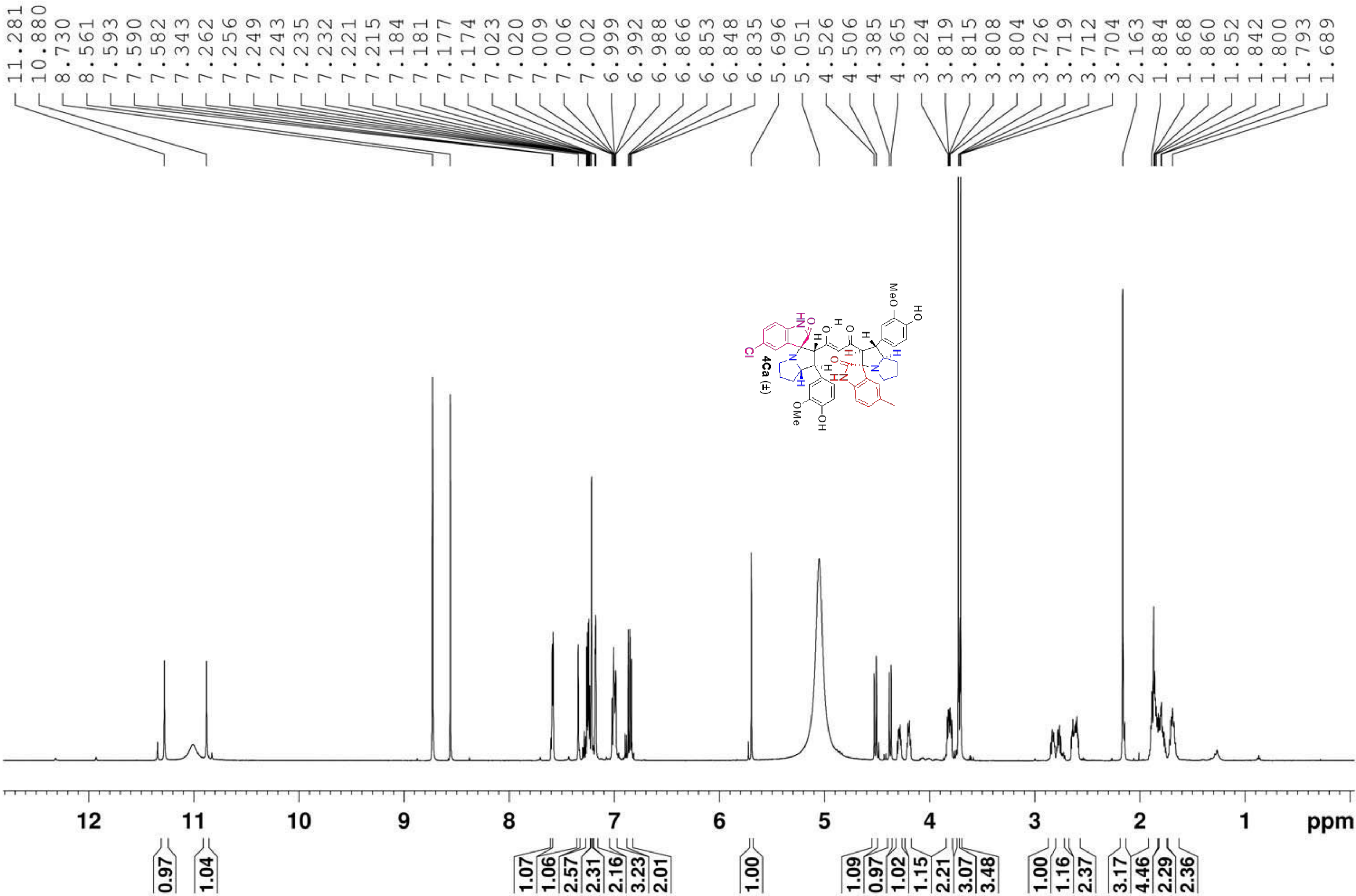

13C-NMR in Py-d5

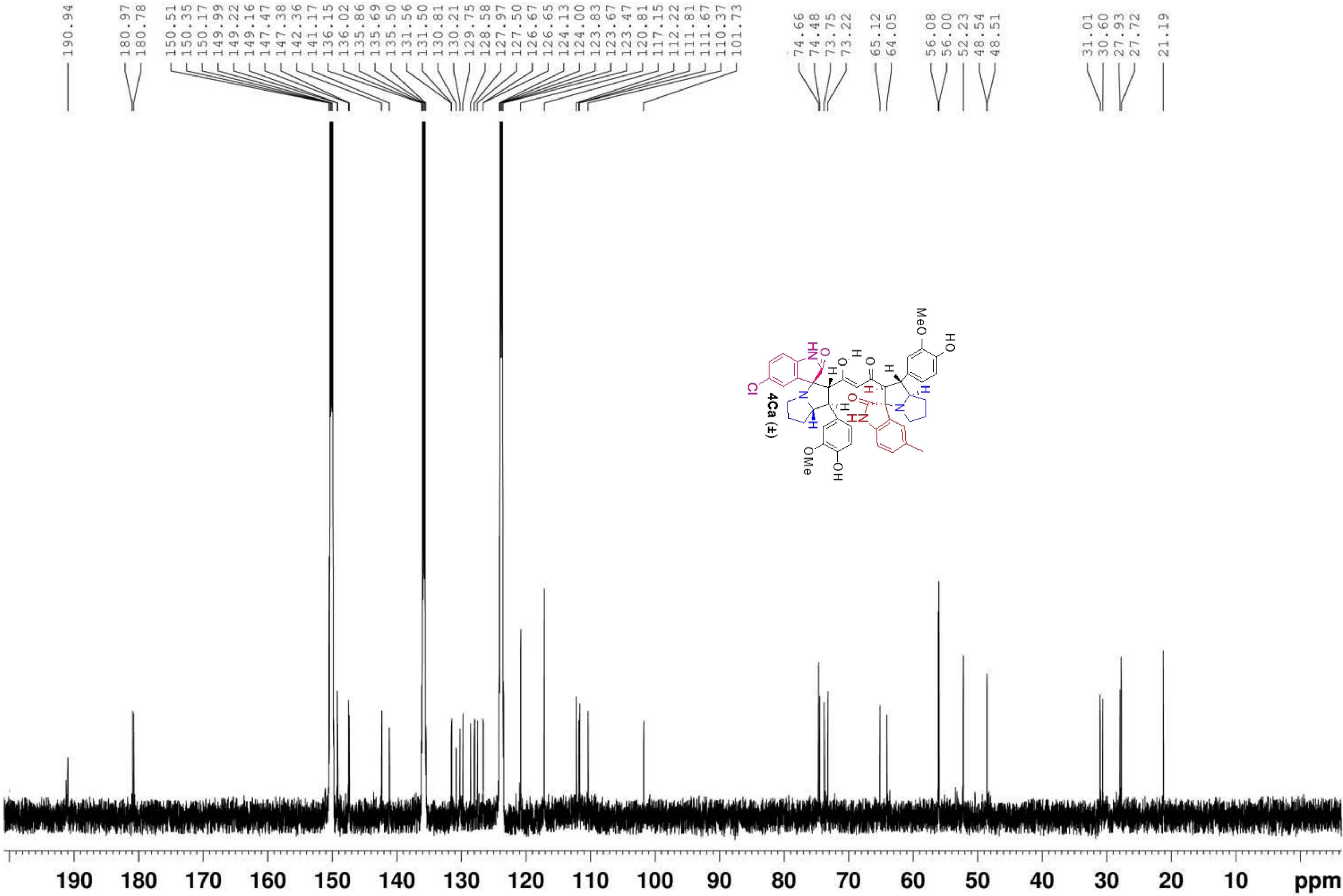

DEPT-135

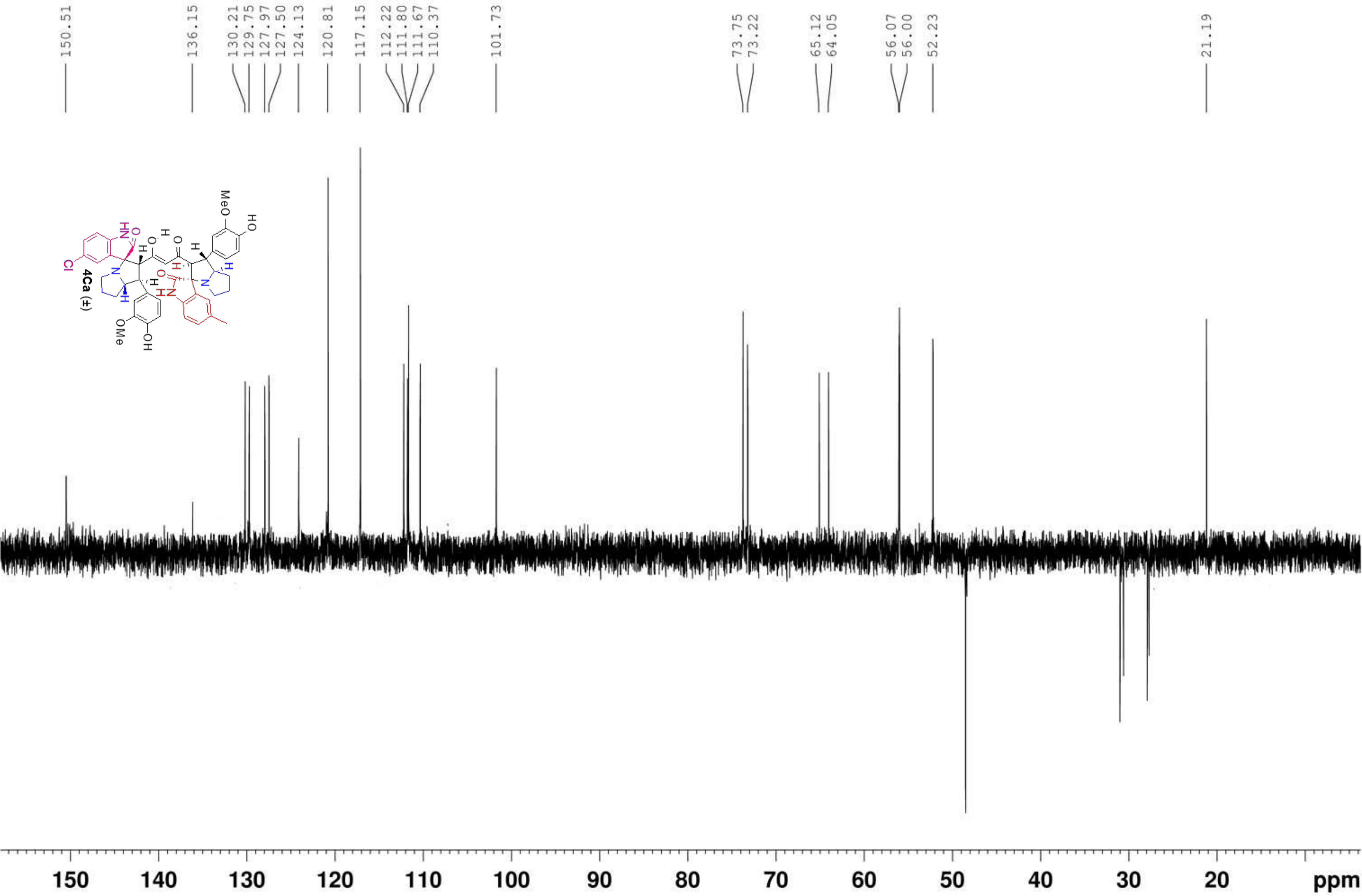

150.51
136.15
130.21
129.75
127.97
127.50
124.13
120.81
117.15
112.22
111.80
111.67
110.37
101.73
73.75
73.22
65.12
64.05
56.07
56.00
52.23
21.19
MeO
HO
OMe
OH
Cl
4Ca (±)
150
140
130
120
110
100
90
80
70
60
50
40
30
20
ppm

1H NMR in Py-d5

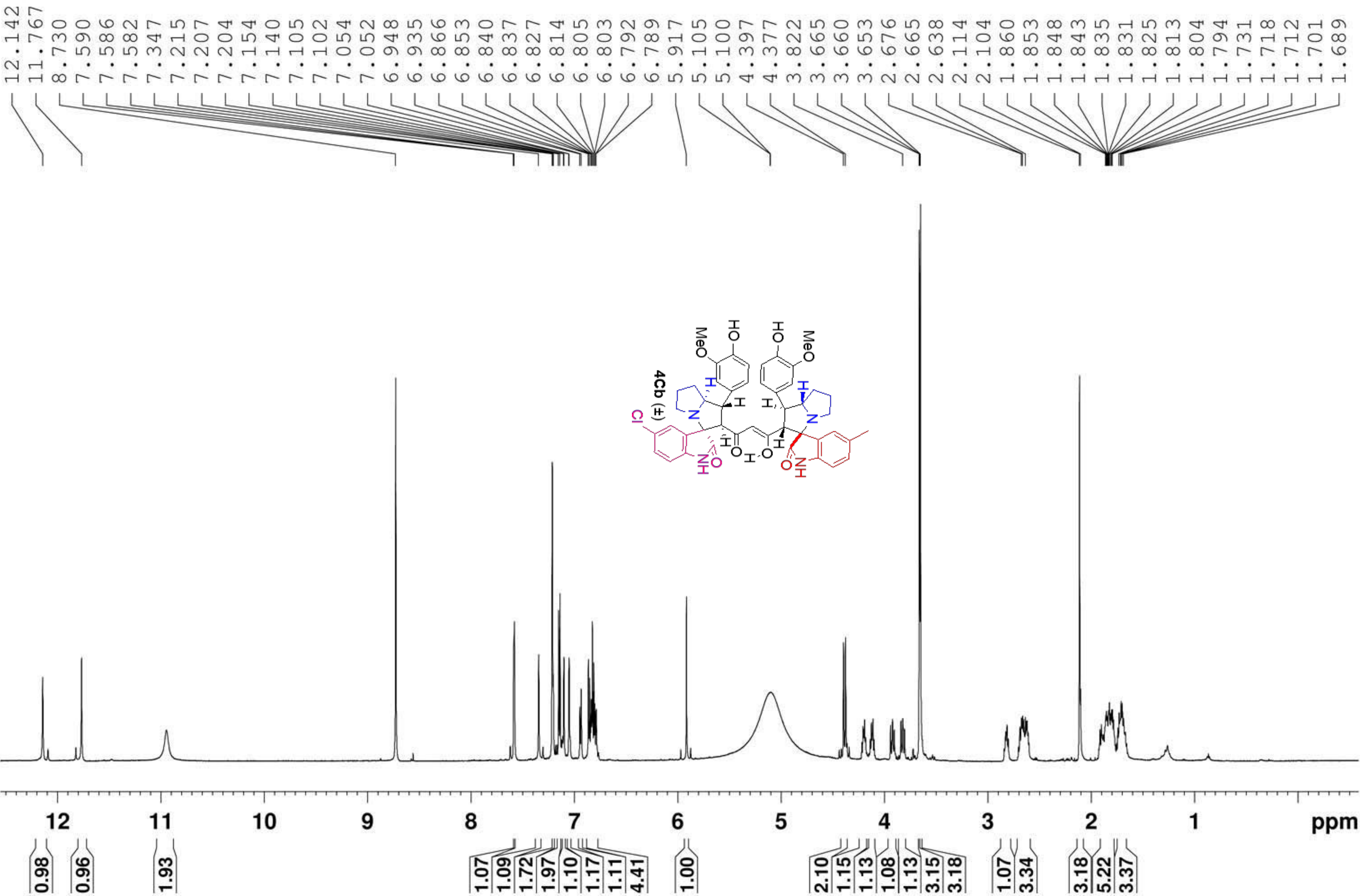

13C-NMR in Py-d5

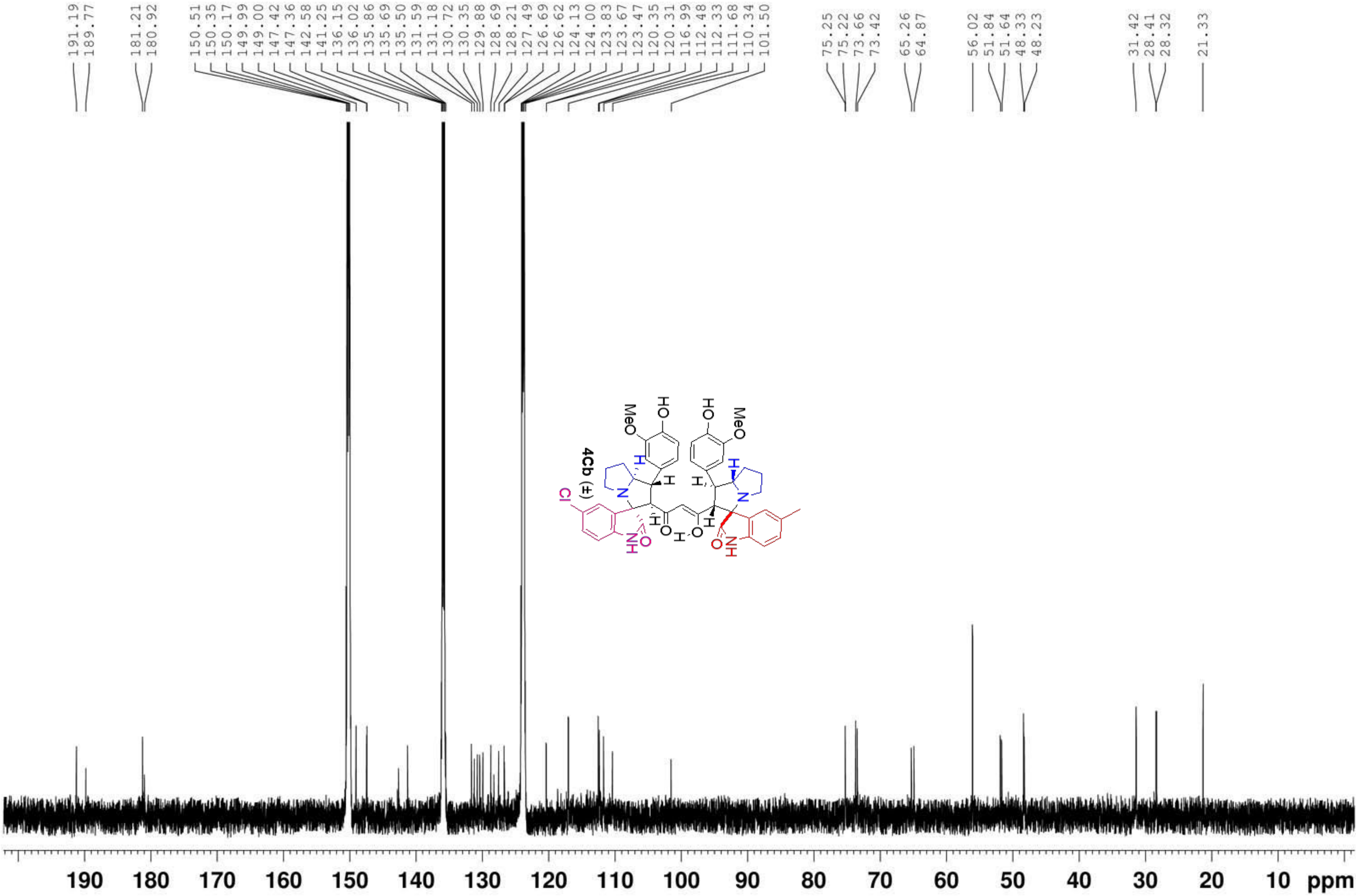

DEPT-135

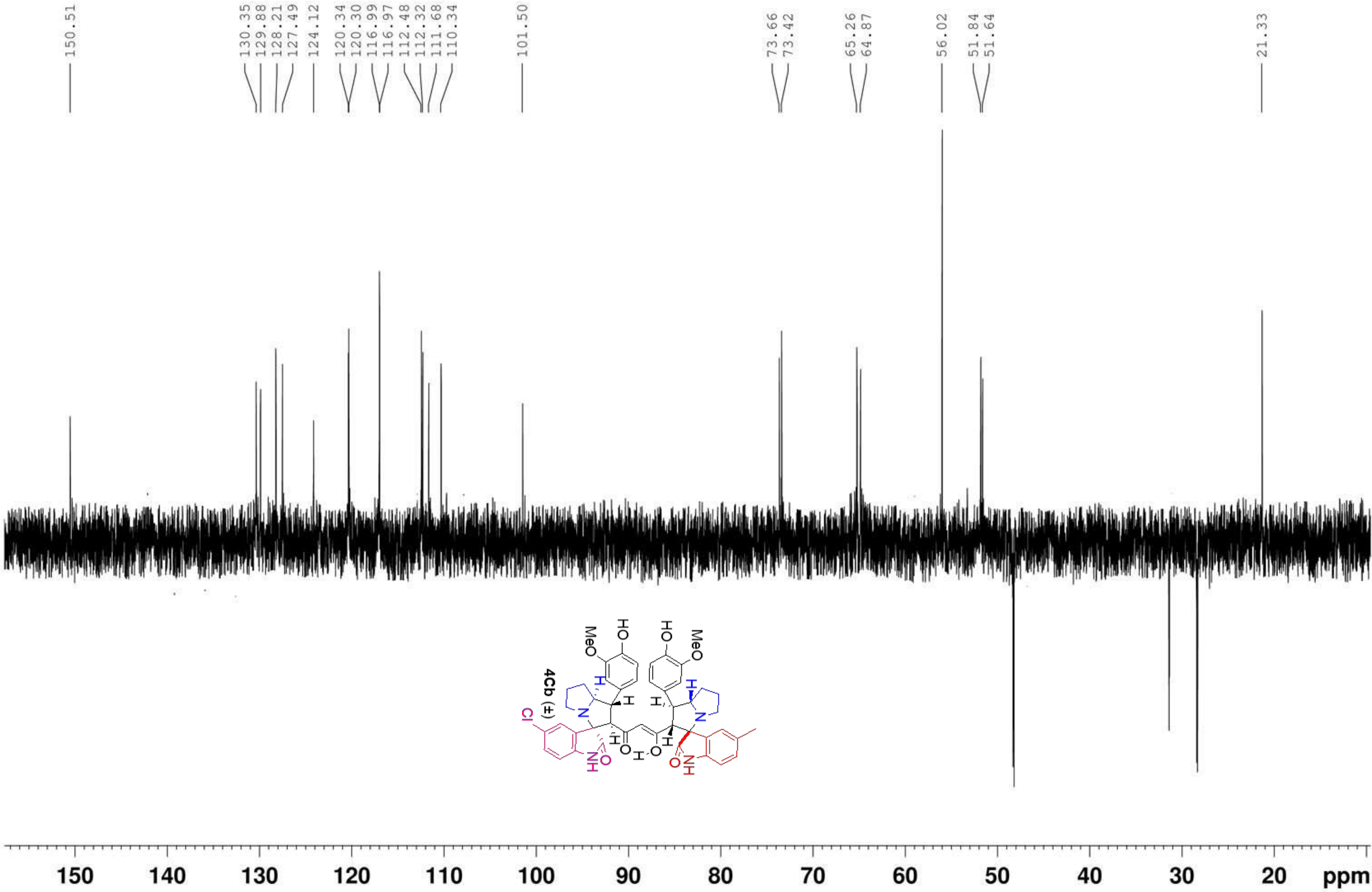
150.51
130.35
129.88
128.21
127.49
124.12
120.34
120.30
116.99
116.97
112.48
112.32
111.68
110.34
101.50
73.66
73.42
65.26
64.87
56.02
51.84
51.64
21.33
MeO
HO
NH
O
N
H
Cl
4Cb (±)
150
140
130
120
110
100
90
80
70
60
50
40
30
20
ppm

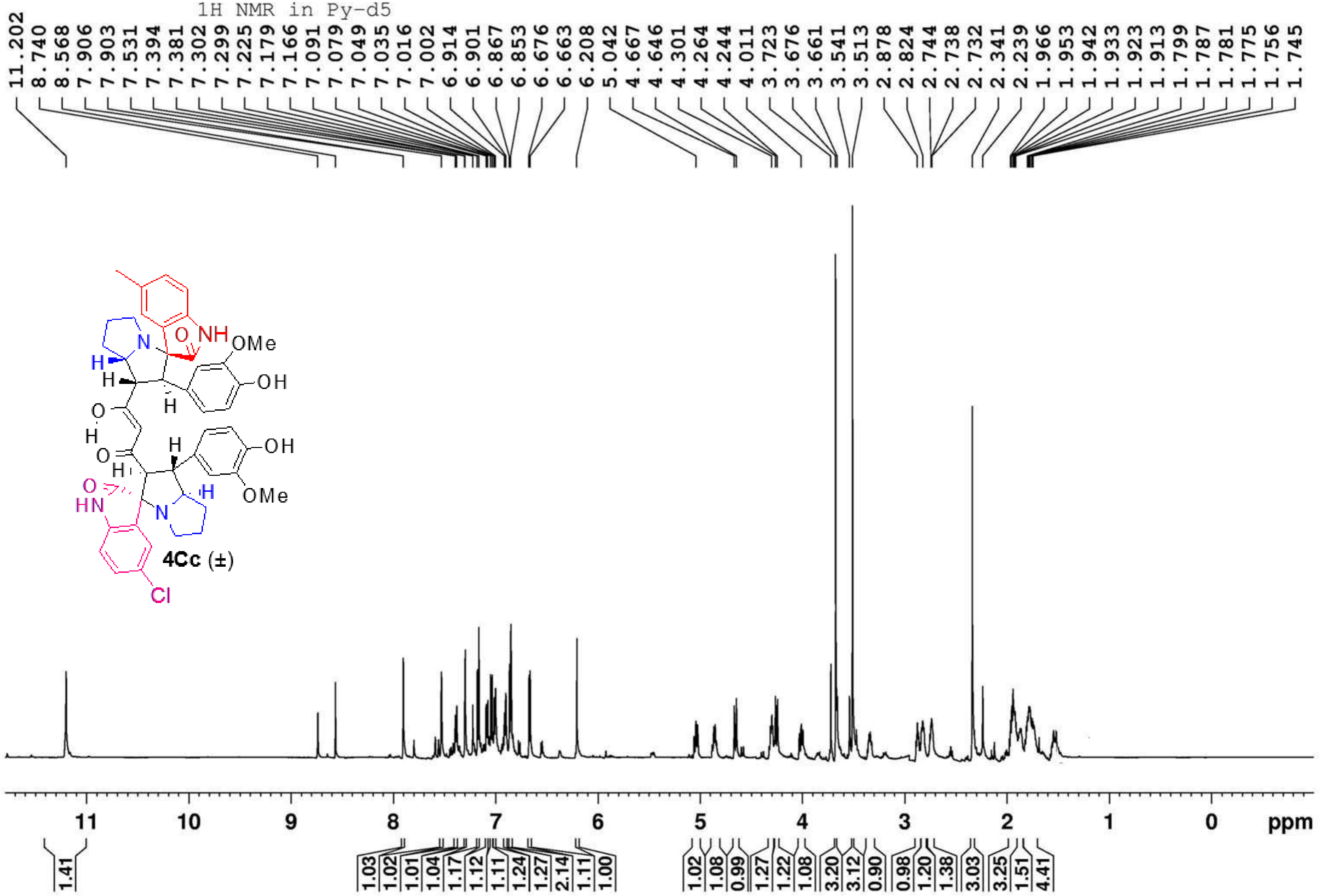
1H NMR in Py-d5
11.202 8.740 8.568 7.906 7.903 7.531 7.394 7.381 7.302 7.299 7.225 7.179 7.166 7.091 7.079 7.049 7.035 7.016 7.002 6.914 6.901 6.867 6.853 6.676 6.663 6.208 5.042 4.667 4.646 4.301 4.264 4.244 4.011 3.723 3.676 3.661 3.541 3.513 2.878 2.824 2.744 2.738 2.732 2.341 2.239 1.966 1.953 1.942 1.933 1.923 1.913 1.799 1.787 1.781 1.775 1.756 1.745
11 10 9 8 7 6 5 4 3 2 1 0 ppm
1.41
1.03 1.02 1.01 1.04 1.17 1.12 1.11 1.24 1.27 2.14 1.11 1.00
1.02 1.08 0.99 1.27 1.22 1.08 3.20 3.12 0.90 0.98 1.20 1.38 3.03 3.25 1.51 4.41
OMe
OH
NH
O
N
H
OH
OMe
HN
Cl
4Cc (±)

13C-NMR in Py-d5

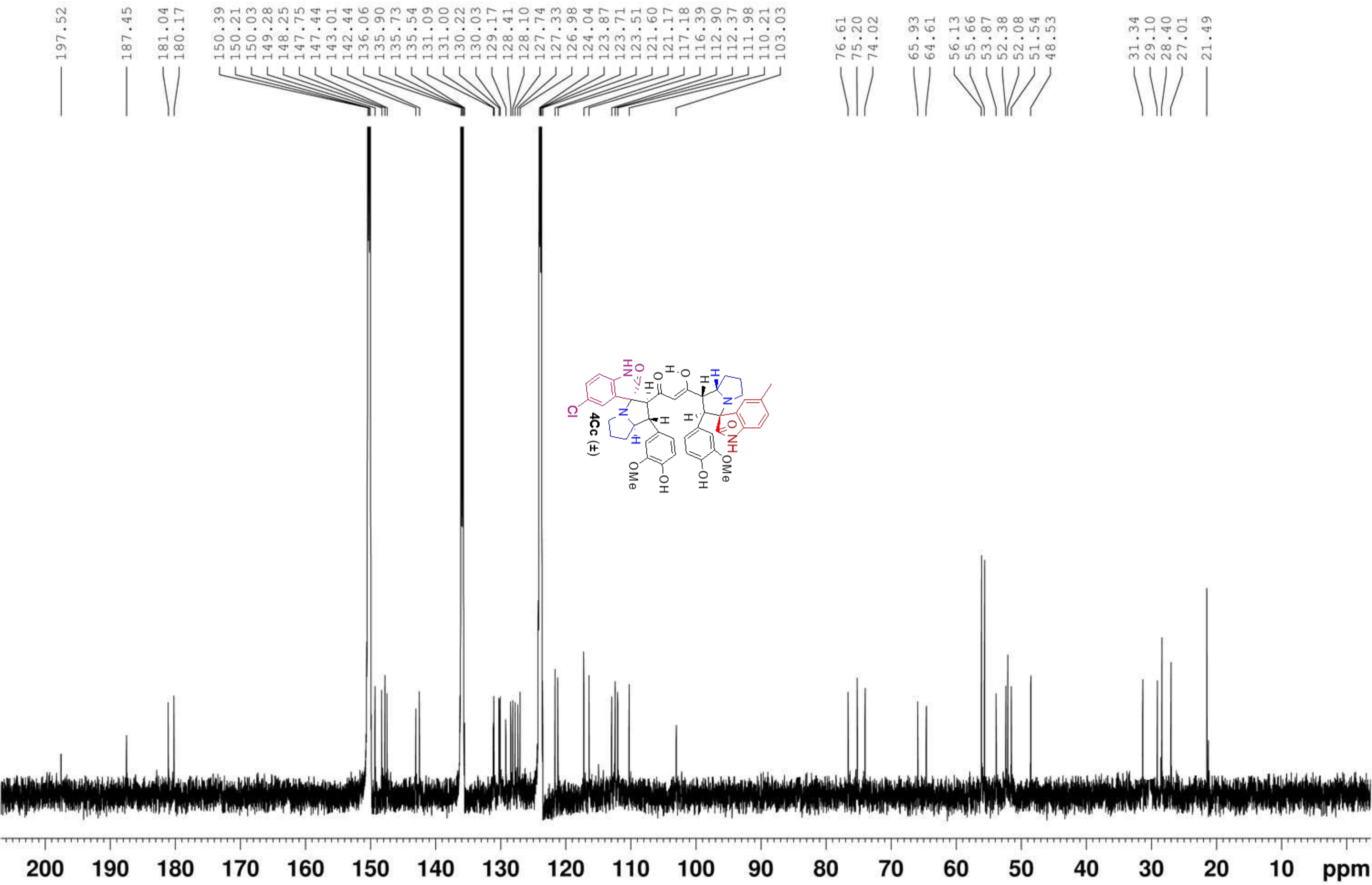

4Cc (±)

DEPT-135

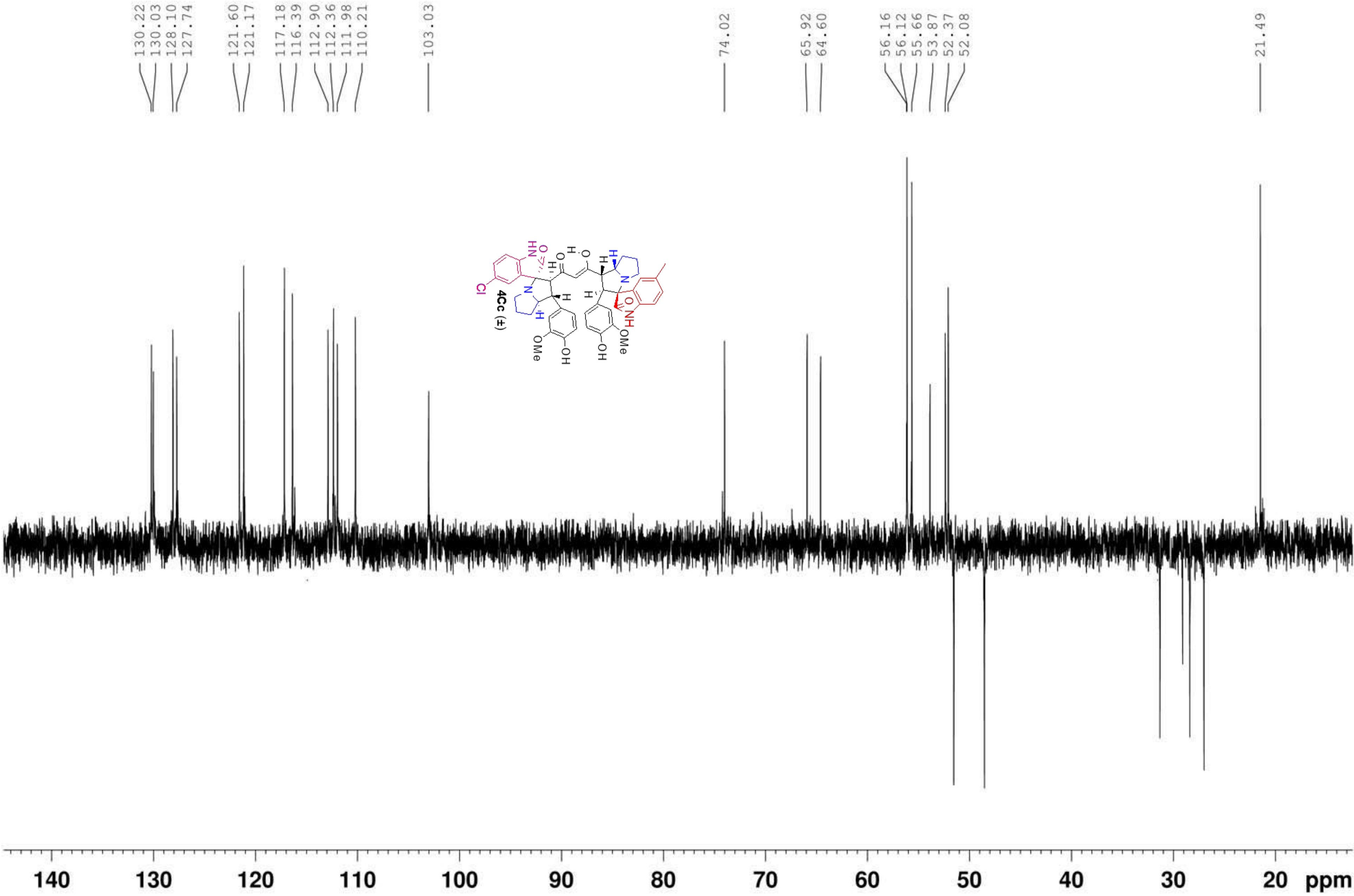
130.22
130.03
128.10
127.74
121.60
121.17
117.18
116.39
112.90
112.36
111.98
110.21
103.03
74.02
65.92
64.60
56.16
56.12
55.66
53.87
52.37
52.08
21.49
4Cc (±)
OMe
OH
NH
HN
Cl
140
130
120
110
100
90
80
70
60
50
40
30
20
ppm

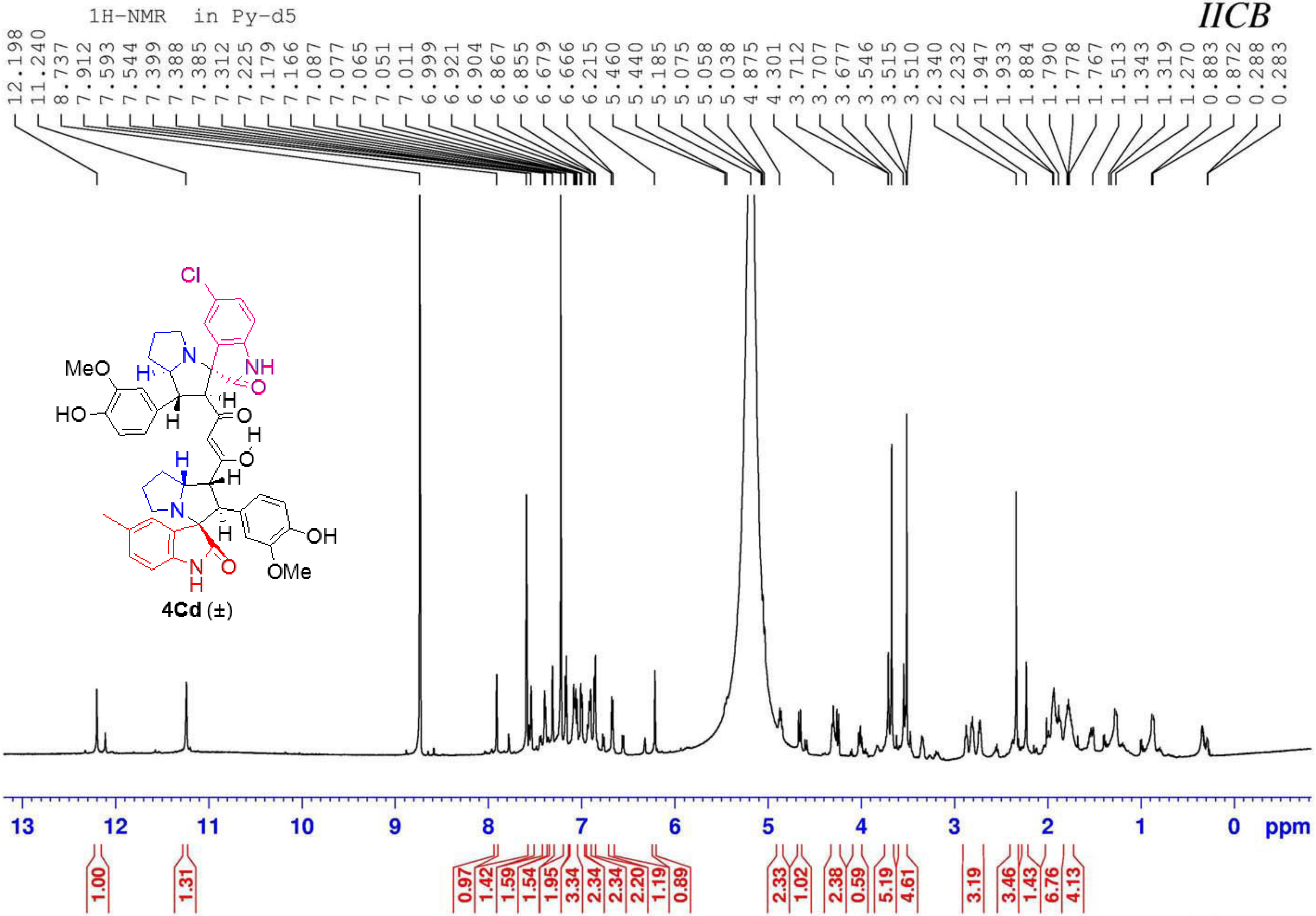
1H-NMR in Py-d5
IICB
12.198 11.240 8.737 7.912 7.593 7.544 7.399 7.388 7.385 7.312 7.225 7.179 7.166 7.087 7.077 7.065 7.051 7.011 6.999 6.921 6.904 6.867 6.855 6.679 6.666 6.215 5.460 5.440 5.185 5.075 5.058 5.038 4.875 4.301 3.712 3.707 3.677 3.546 3.515 3.510 2.340 2.232 1.947 1.933 1.884 1.790 1.778 1.767 1.513 1.343 1.319 1.270 0.883 0.872 0.288 0.283
Cl
NH
O
MeO
HO
H
N
O
H
OH
OMe
4Cd (±)
13 12 11 10 9 8 7 6 5 4 3 2 1 0 ppm
1.00 1.31 0.97 1.42 1.59 1.54 1.95 3.34 2.34 2.34 2.20 1.19 0.89 2.33 1.02 2.38 0.59 5.19 4.61 3.19 3.46 1.43 6.76 4.13

13C-NMR in Py-d5

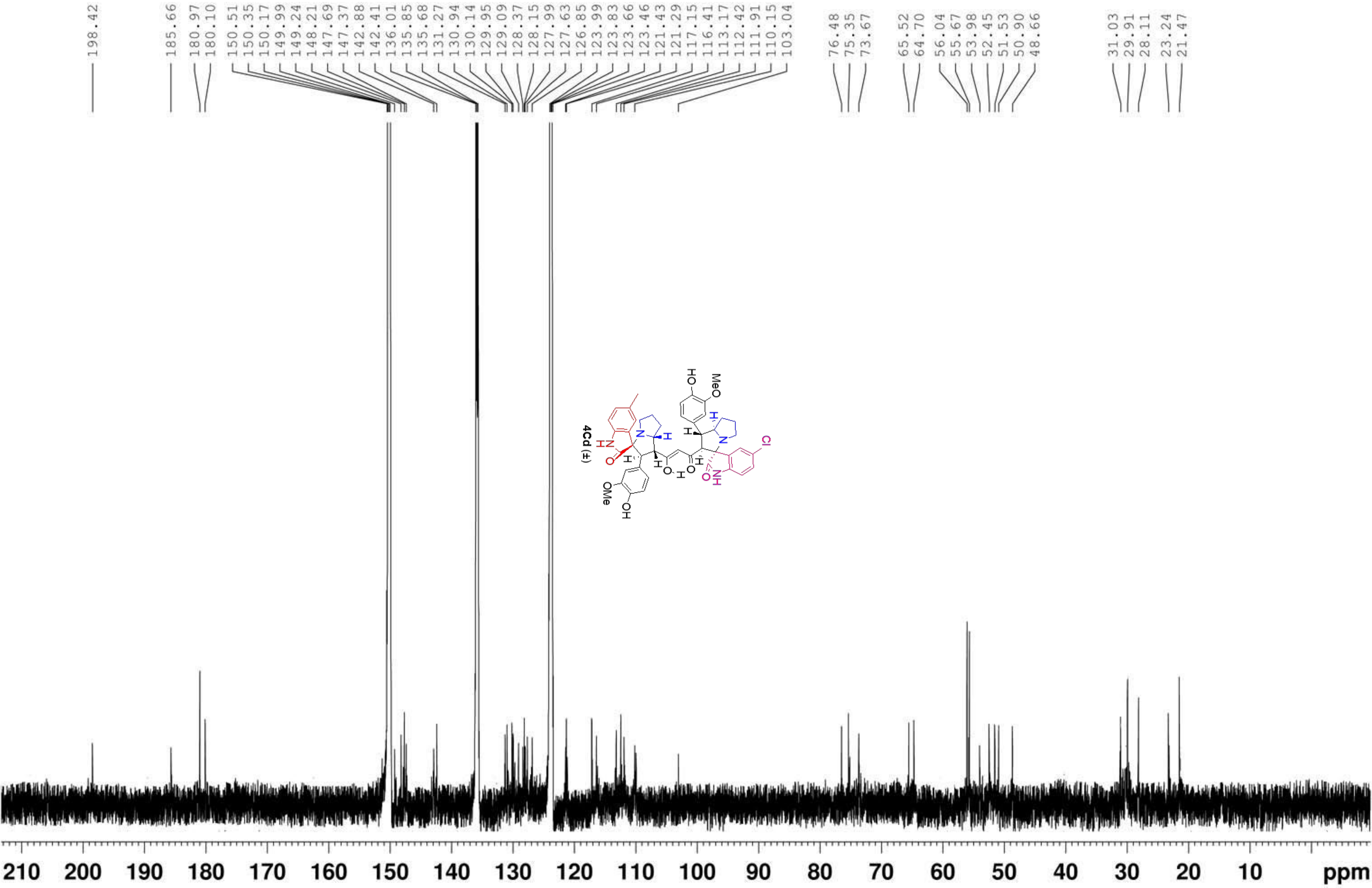

4Cd (±)

DEPT-135

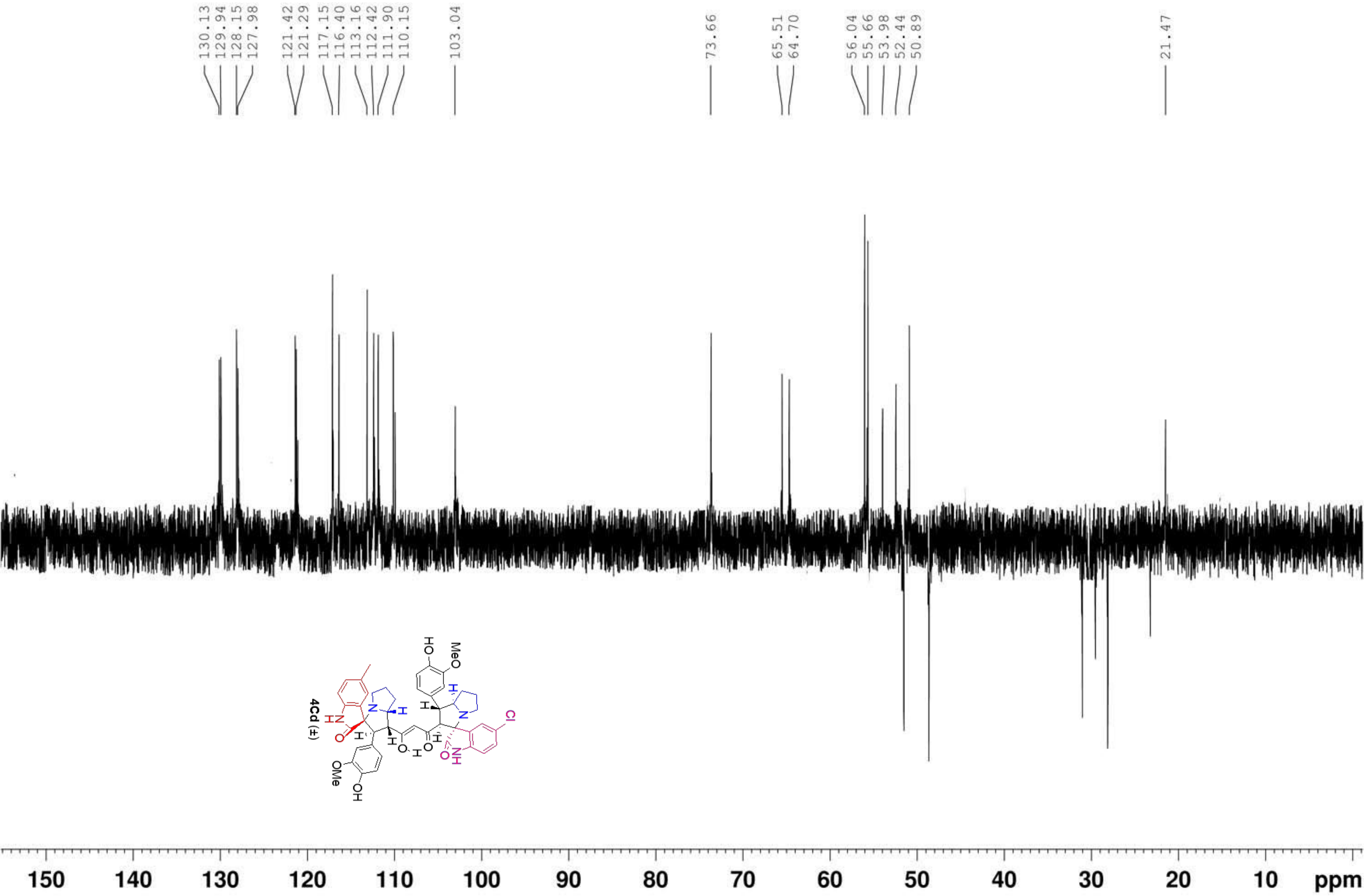

130.13
129.94
128.15
127.98
121.42
121.29
117.15
116.40
113.16
112.42
111.90
110.15
103.04
73.66
65.51
64.70
56.04
55.66
53.98
52.44
50.89
21.47
Cl
MeO
HO
NH
O
H
N
OH
OMe
4Cd (±)
150
140
130
120
110
100
90
80
70
60
50
40
30
20
10
ppm

1H NMR in Py-d5

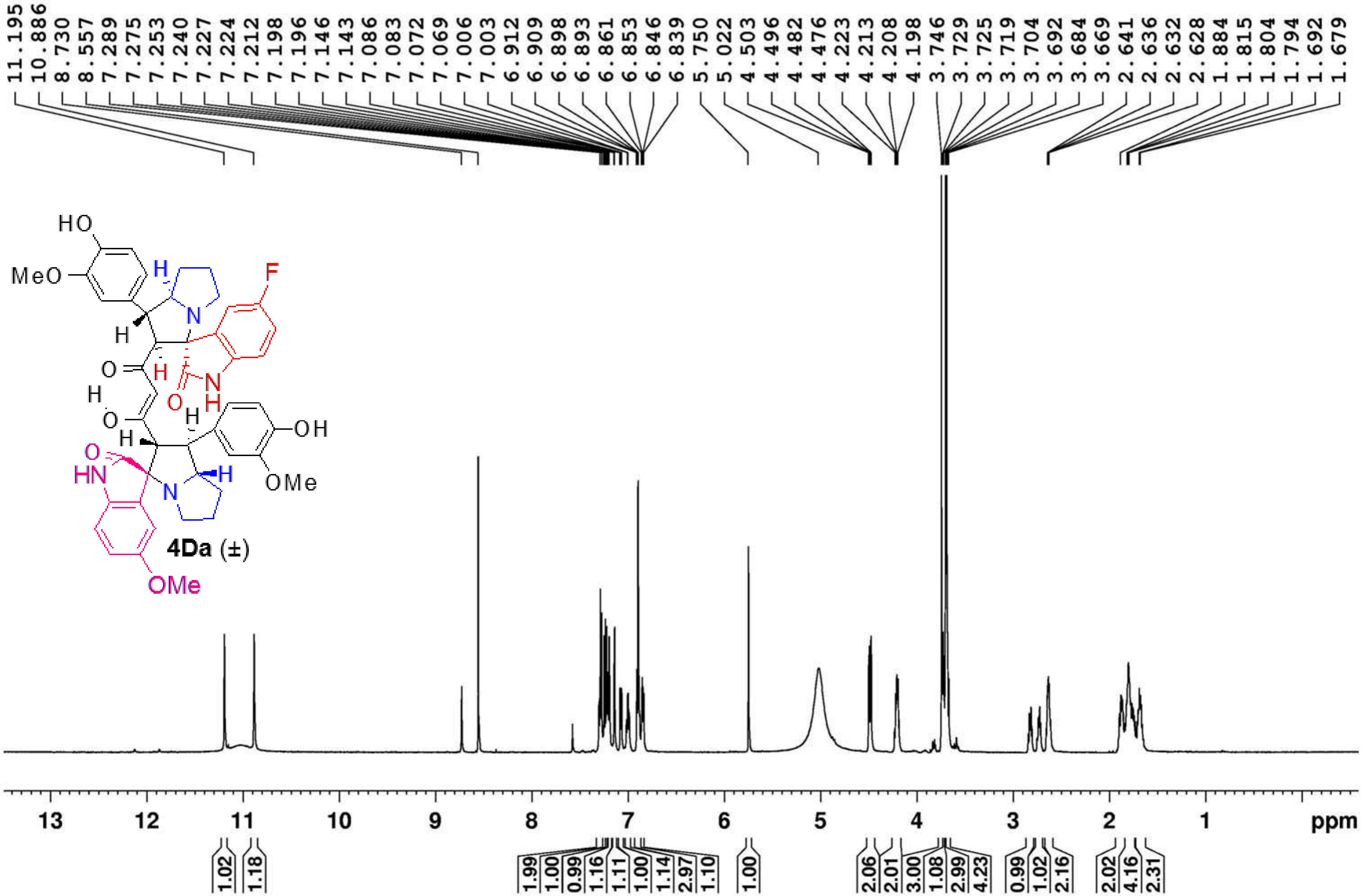

13C-NMR in Py-d5

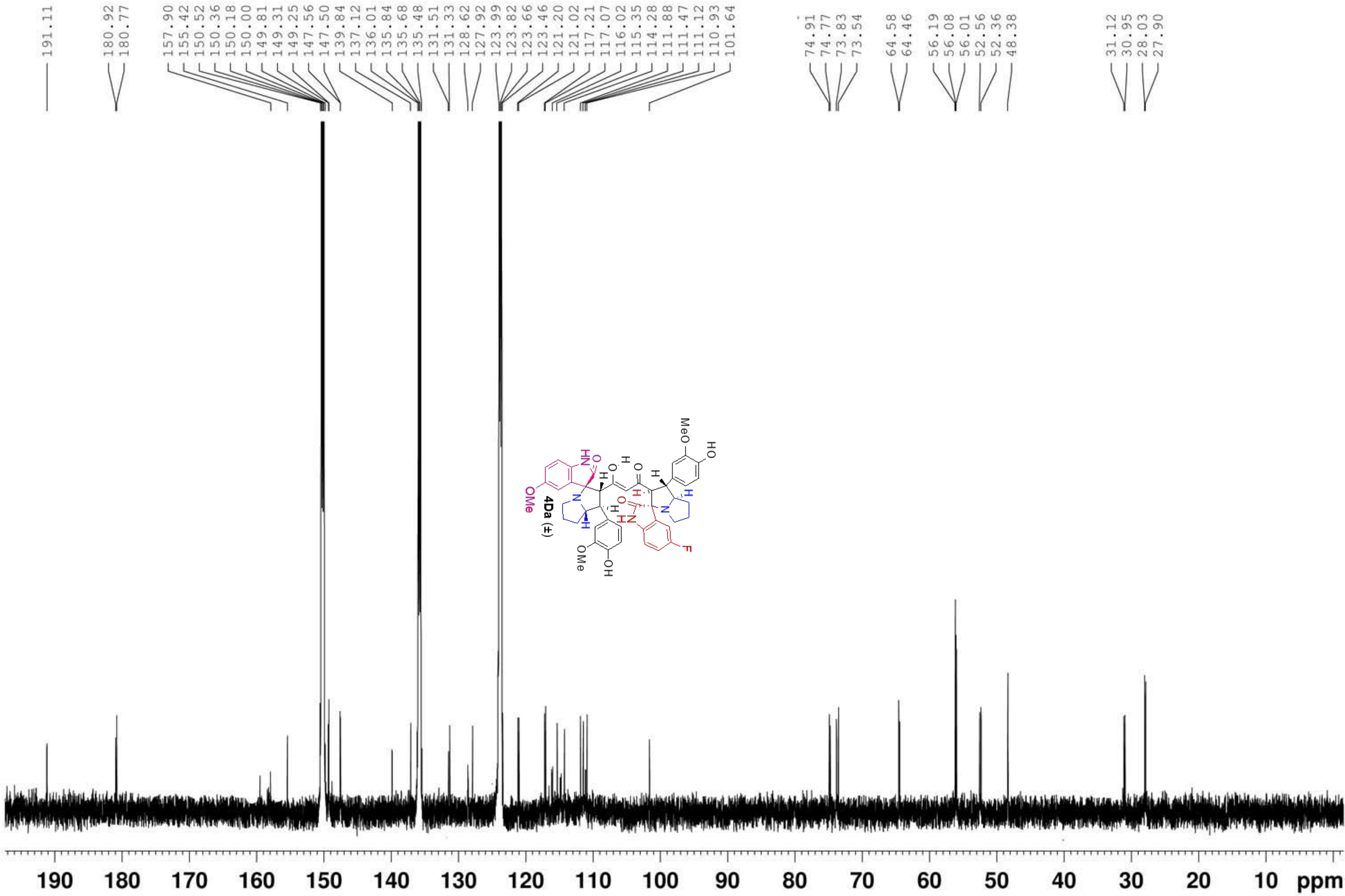

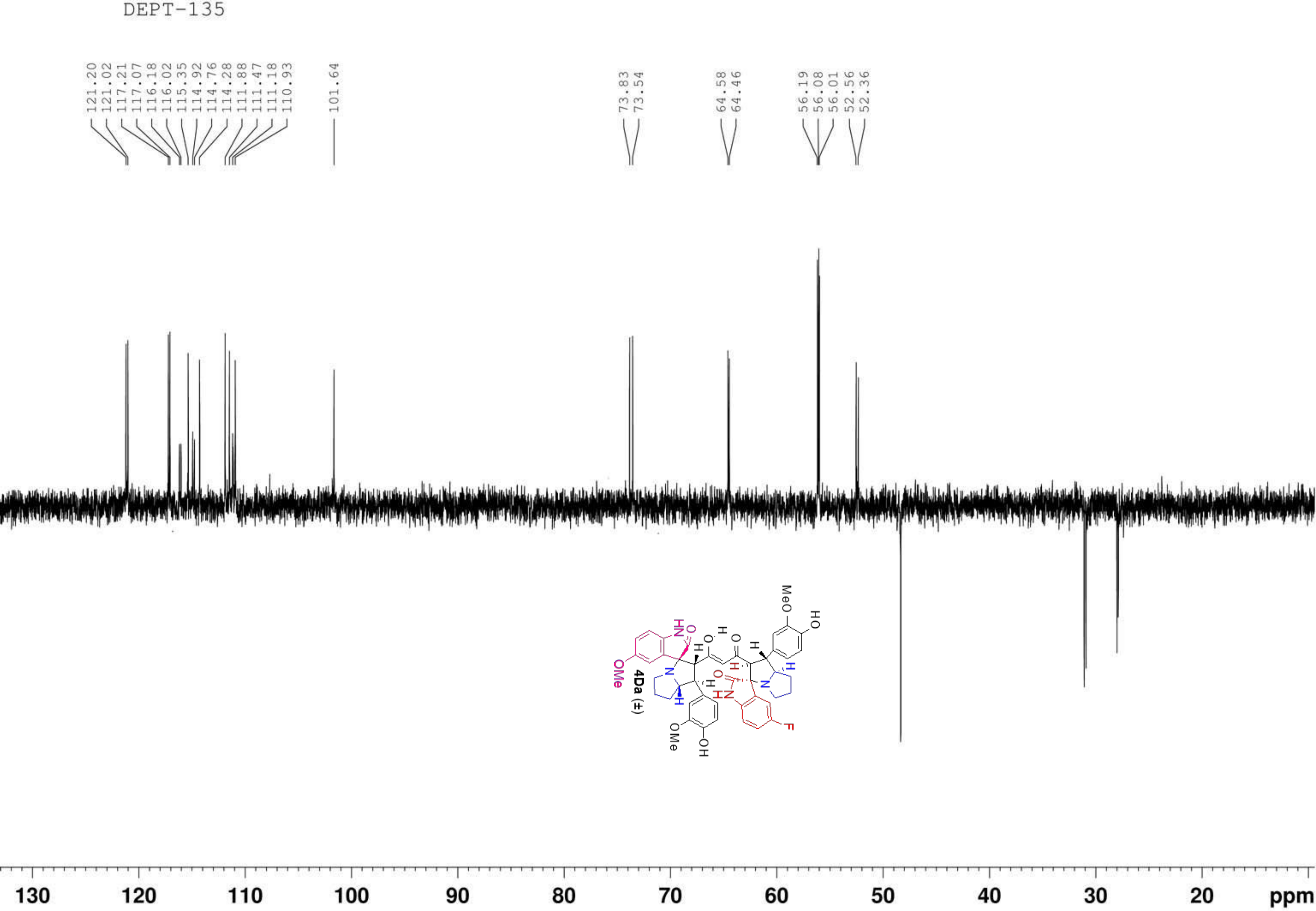
DEPT-135
121.20
121.02
117.21
117.07
116.18
116.02
115.35
114.92
114.76
114.28
111.88
111.47
111.18
110.93
101.64
73.83
73.54
64.58
64.46
56.19
56.08
56.01
52.56
52.36
4Da (±)
130
120
110
100
90
80
70
60
50
40
30
20
ppm

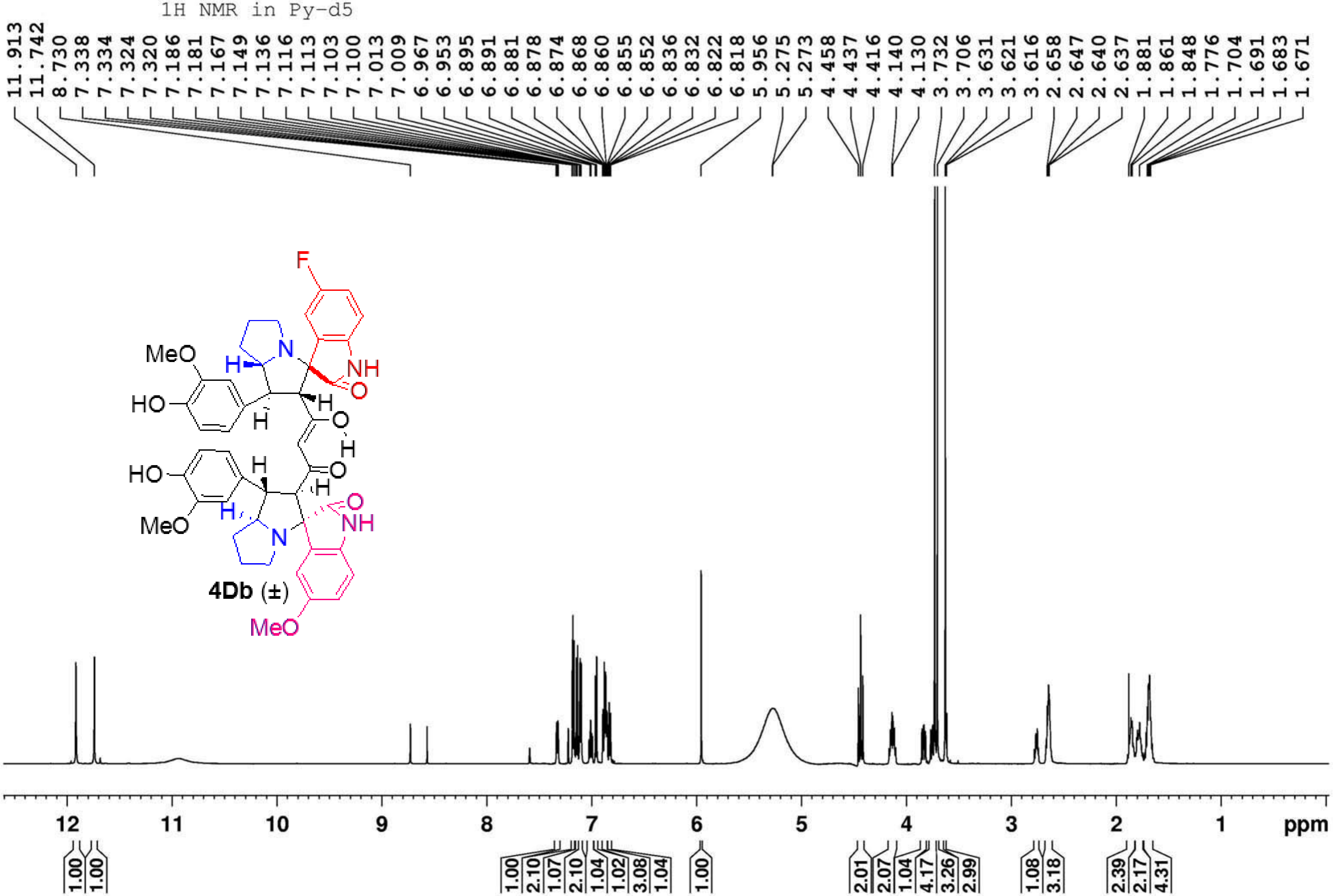
1H NMR in Py-d5
11.913 11.742 8.730 7.338 7.334 7.324 7.320 7.186 7.181 7.167 7.149 7.136 7.116 7.113 7.103 7.100 7.013 7.009 6.967 6.953 6.895 6.891 6.881 6.878 6.874 6.868 6.860 6.855 6.852 6.836 6.832 6.822 6.818 5.956 5.275 5.273 4.458 4.437 4.416 4.140 4.130 3.732 3.706 3.631 3.621 3.616 2.658 2.647 2.640 2.637 1.881 1.861 1.848 1.776 1.704 1.691 1.683 1.671
F
MeO
HO
N
H
NH
O
HO
MeO
4Db (±)
MeO
12 11 10 9 8 7 6 5 4 3 2 1 ppm
1.00 1.00 1.00 2.10 1.07 2.10 1.04 1.02 3.08 1.04 1.00 2.01 2.07 1.04 4.17 3.26 2.99 1.08 3.18 2.39 2.17 4.31

13C-NMR in Py-d5

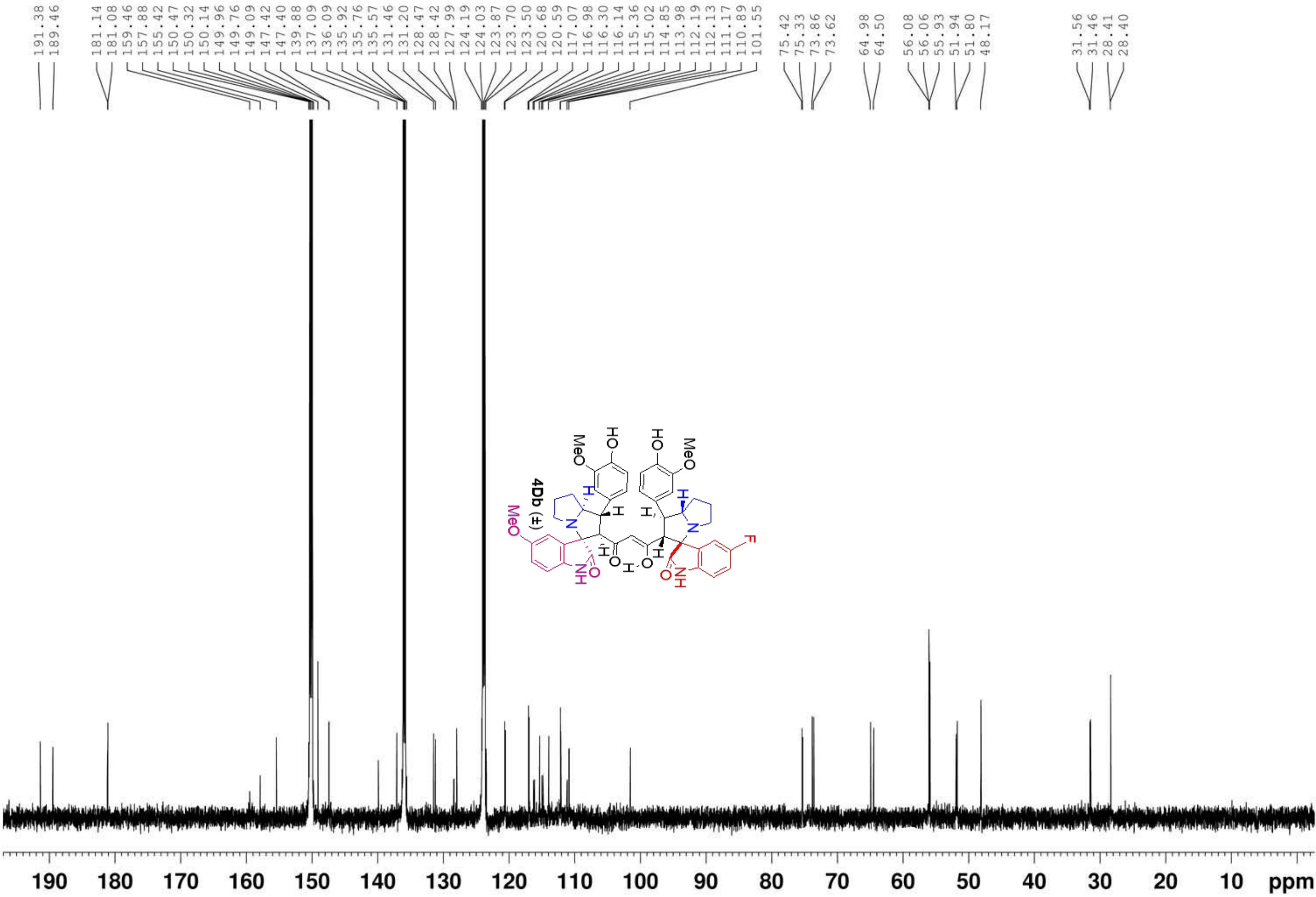

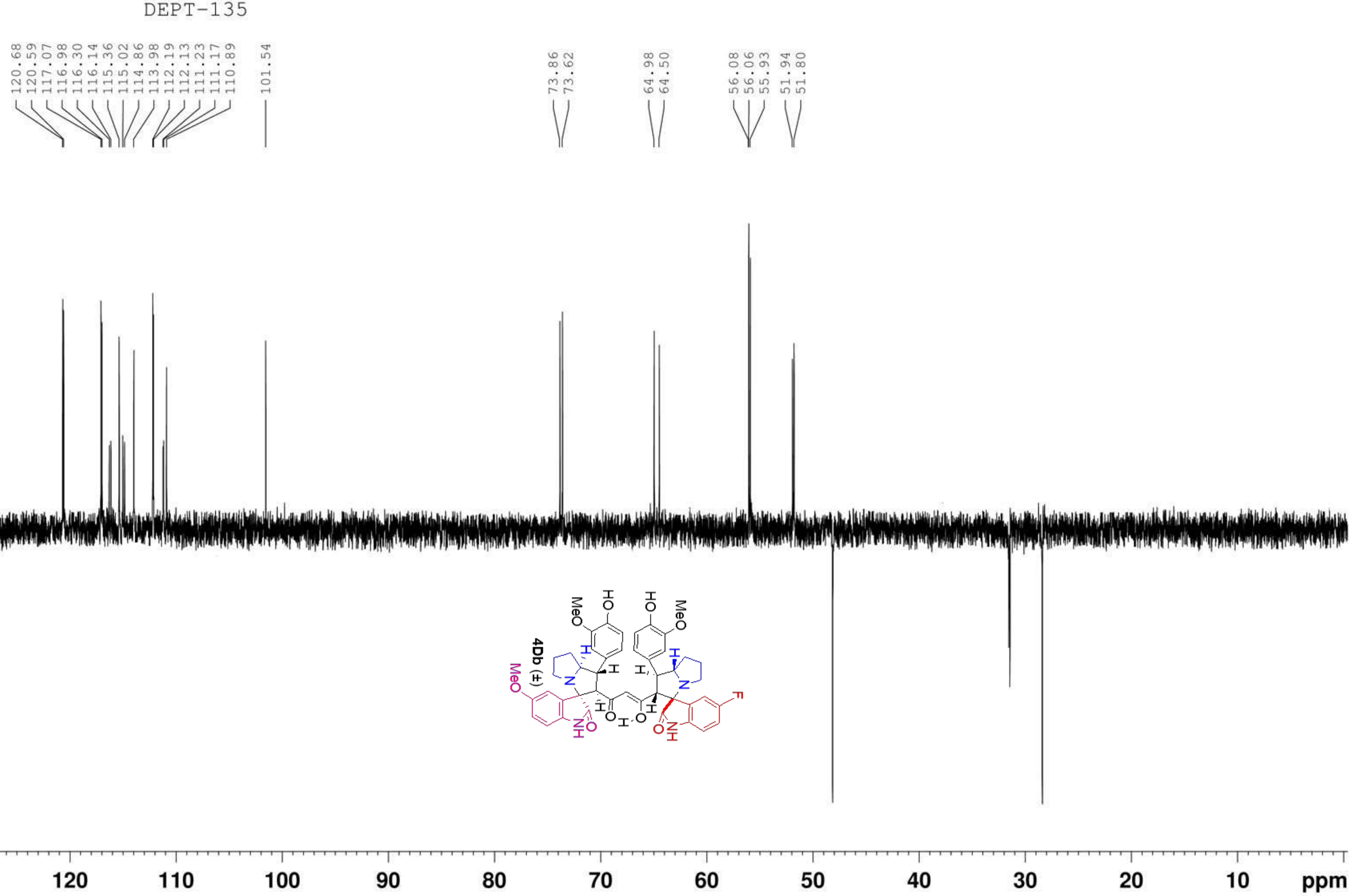
DEPT-135
120.68
120.59
117.07
116.98
116.30
116.14
115.36
115.02
114.86
113.98
112.19
112.13
111.23
111.17
110.89
101.54
73.86
73.62
64.98
64.50
56.08
56.06
55.93
51.94
51.80
MeO
HO
HO
MeO
F
NH
O
N
MeO
4Db (±)
120
110
100
90
80
70
60
50
40
30
20
10
ppm

1H NMR in Py-d5

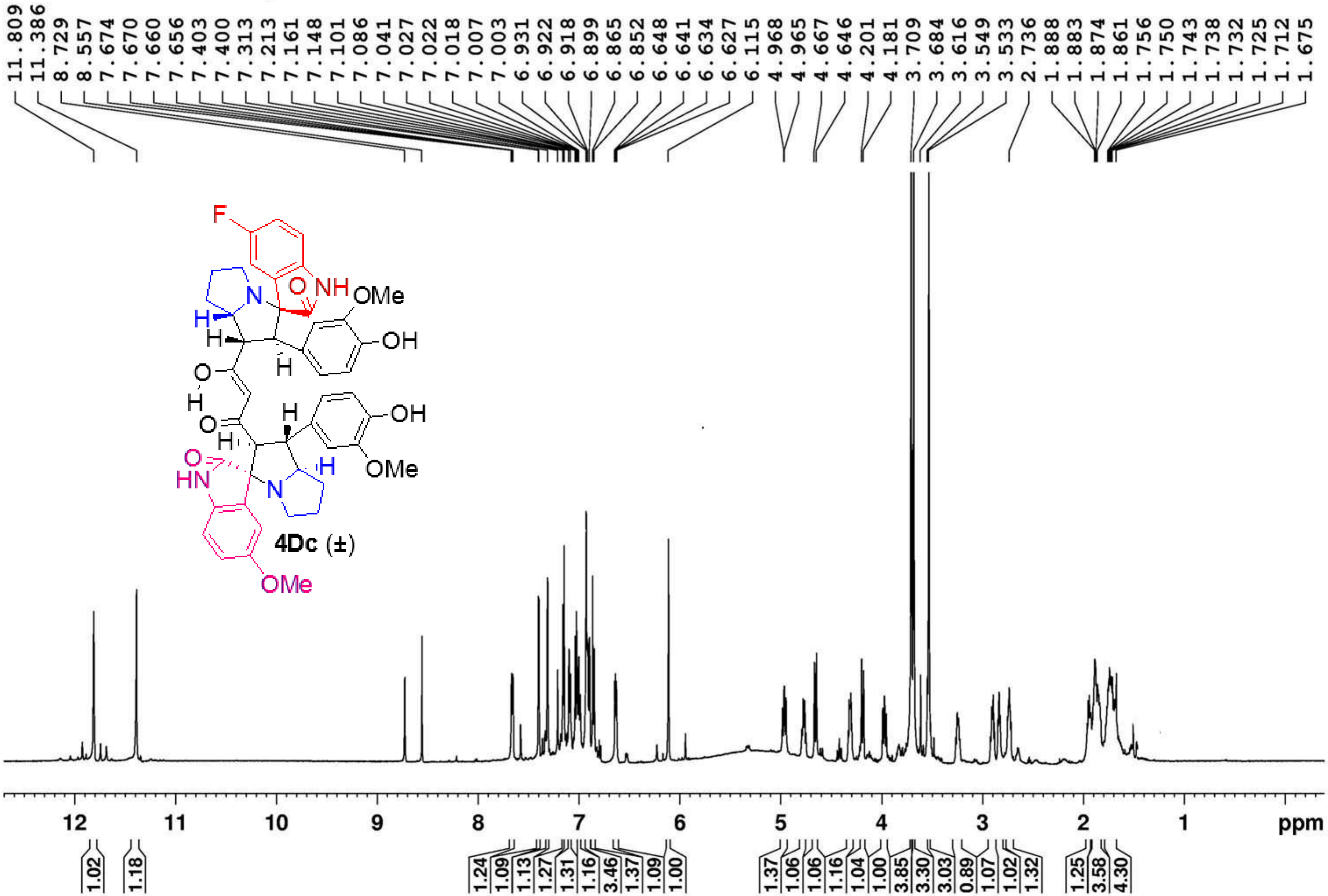

13C-NMR in Py-d5

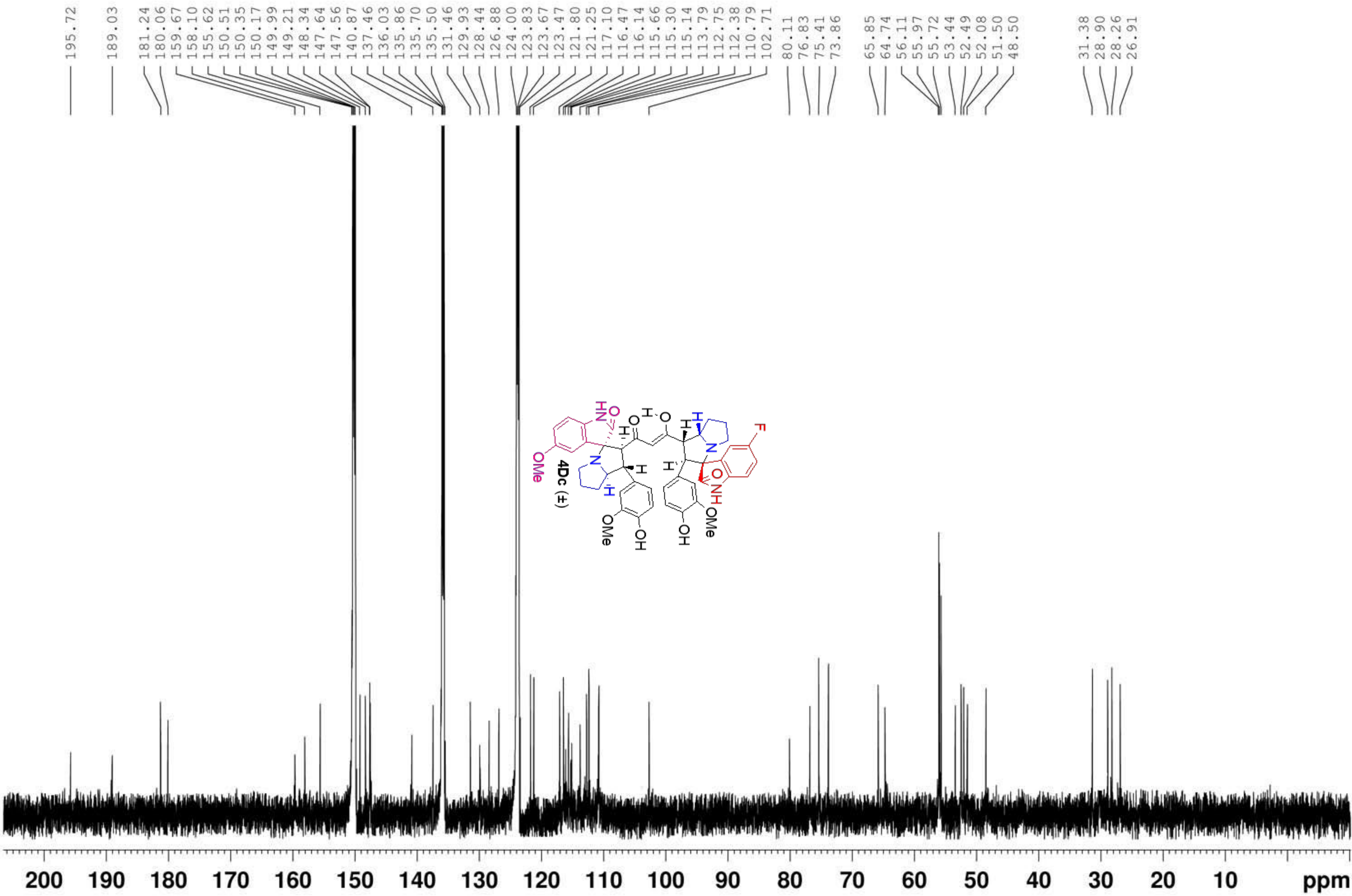

DEPT-135

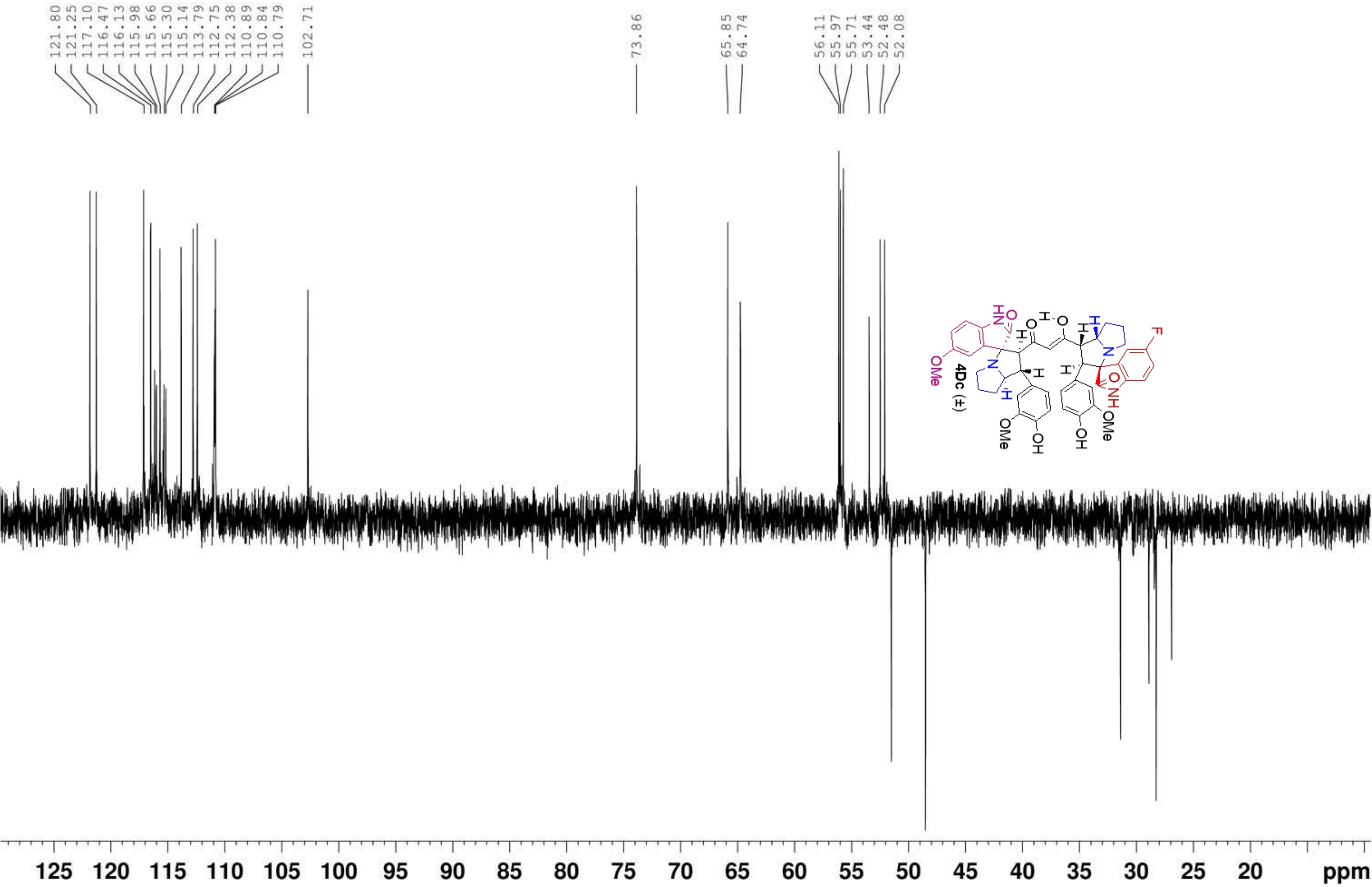

121.80
121.25
117.10
116.47
116.13
115.98
115.66
115.30
115.14
113.79
112.75
112.38
110.89
110.84
110.79
102.71
73.86
65.85
64.74
56.11
55.97
55.71
53.44
52.48
52.08
F
NH
O
OMe
OH
H
O
H
O
HN
O
N
OH
OMe
OMe
4Dc (±)
125
120
115
110
105
100
95
90
85
80
75
70
65
60
55
50
45
40
35
30
25
20
ppm

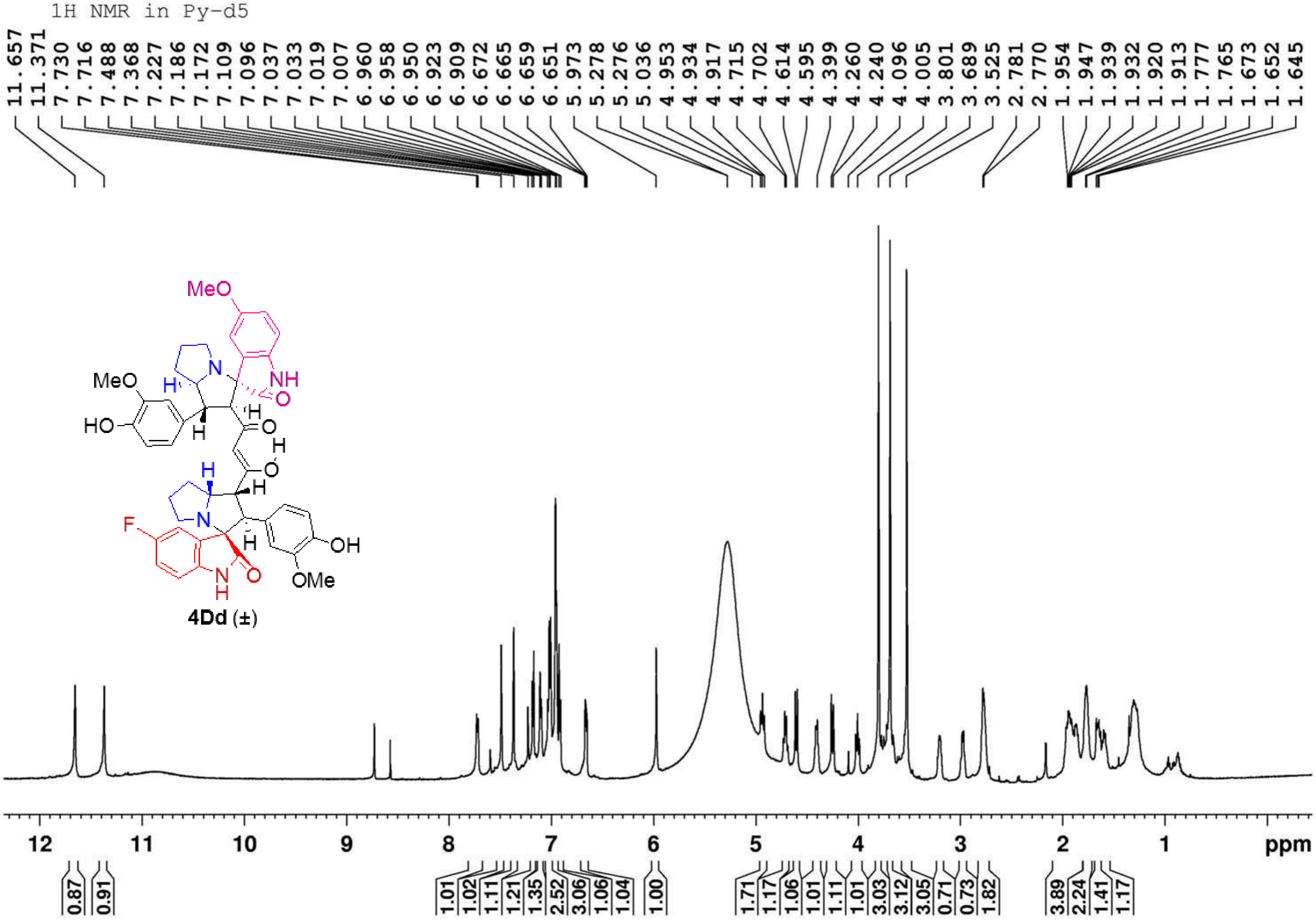
1H NMR in Py-d5
11.657 11.371 7.730 7.716 7.488 7.368 7.227 7.186 7.172 7.109 7.096 7.037 7.033 7.019 7.007 6.960 6.958 6.950 6.923 6.909 6.672 6.665 6.659 6.651 5.973 5.278 5.276 5.036 4.953 4.934 4.917 4.715 4.702 4.614 4.595 4.399 4.260 4.240 4.096 4.005 3.801 3.689 3.525 2.781 2.770 1.954 1.947 1.939 1.932 1.920 1.913 1.777 1.765 1.673 1.652 1.645
MeO
MeO
HO
N
NH
O
OH
F
N
OMe
4Dd (±)
12 11 10 9 8 7 6 5 4 3 2 1 ppm
0.87 0.91 1.01 1.02 1.11 1.21 1.35 2.52 3.06 1.06 1.04 1.00 1.71 1.17 1.06 1.01 1.11 1.01 3.03 3.12 3.05 0.71 0.73 1.82 3.89 2.24 1.41 1.17

13C-NMR in Py-d5

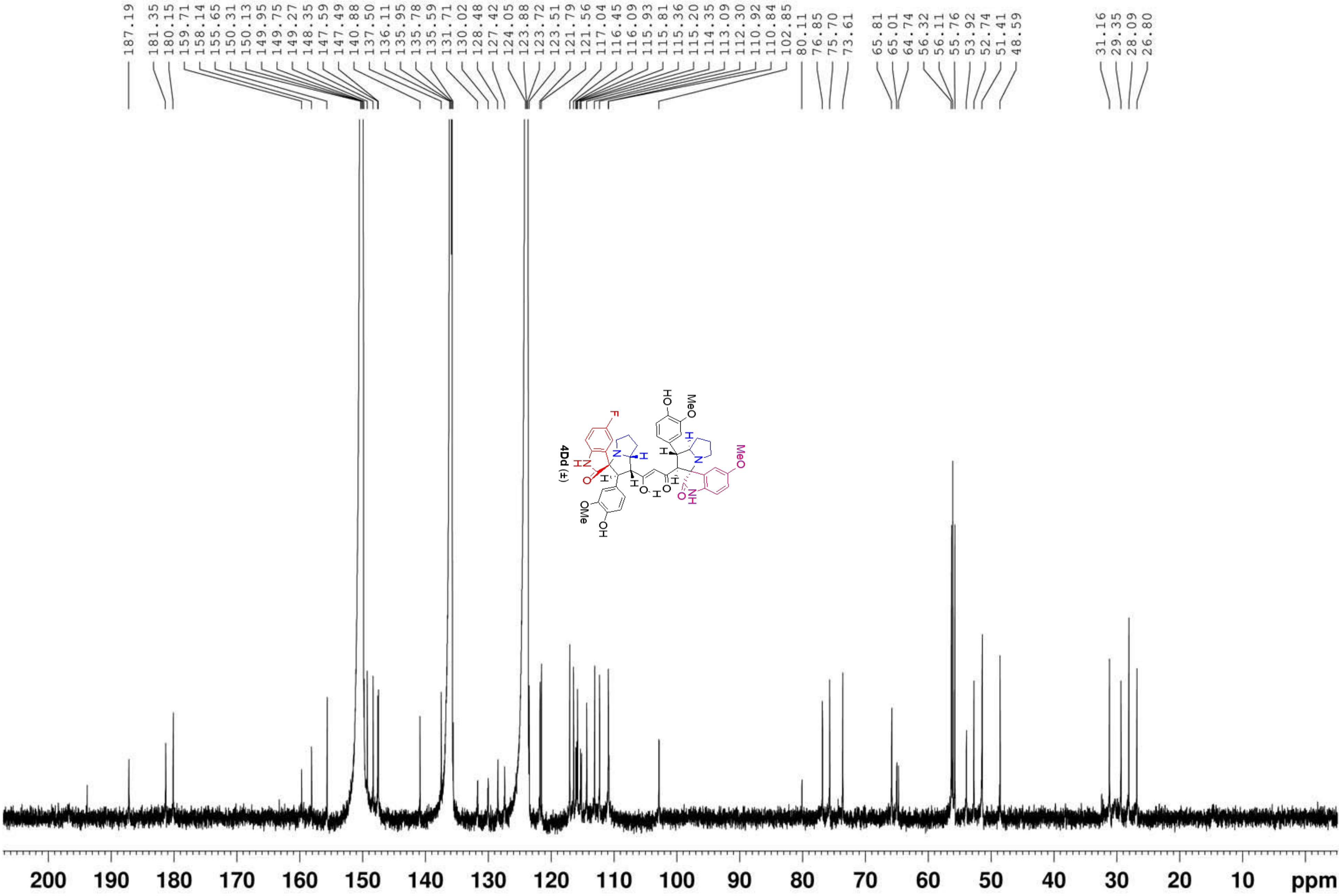

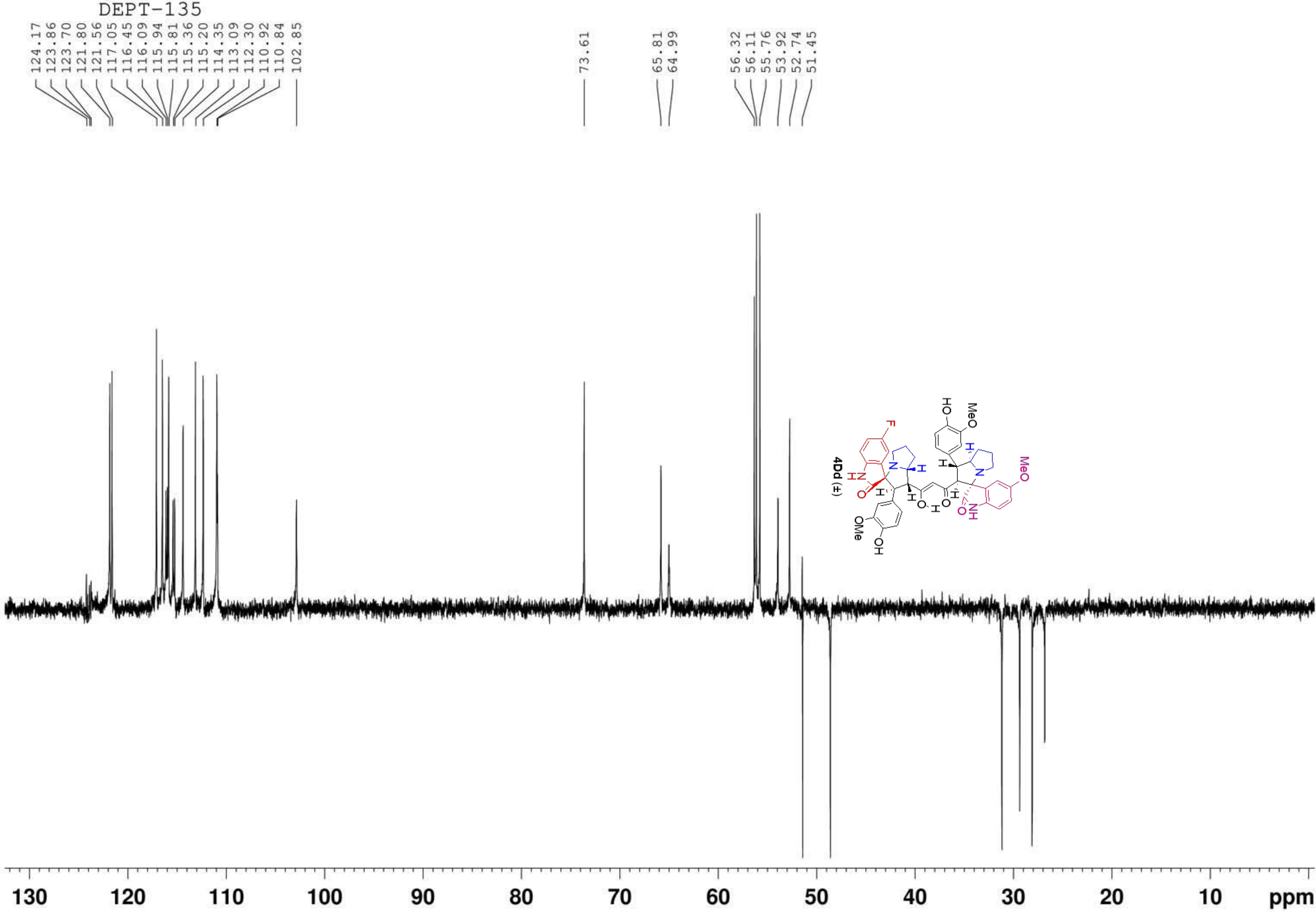
DEPT-135
124.17
123.86
123.70
121.80
121.56
117.05
116.45
116.09
115.94
115.81
115.36
115.20
114.35
113.09
112.30
110.92
110.84
102.85
73.61
65.81
64.99
56.32
56.11
55.76
53.92
52.74
51.45
MeO
HO
F
OMe
OH
NH
4Dd (±)
130
120
110
100
90
80
70
60
50
40
30
20
10
ppm

1H NMR in Py-d5

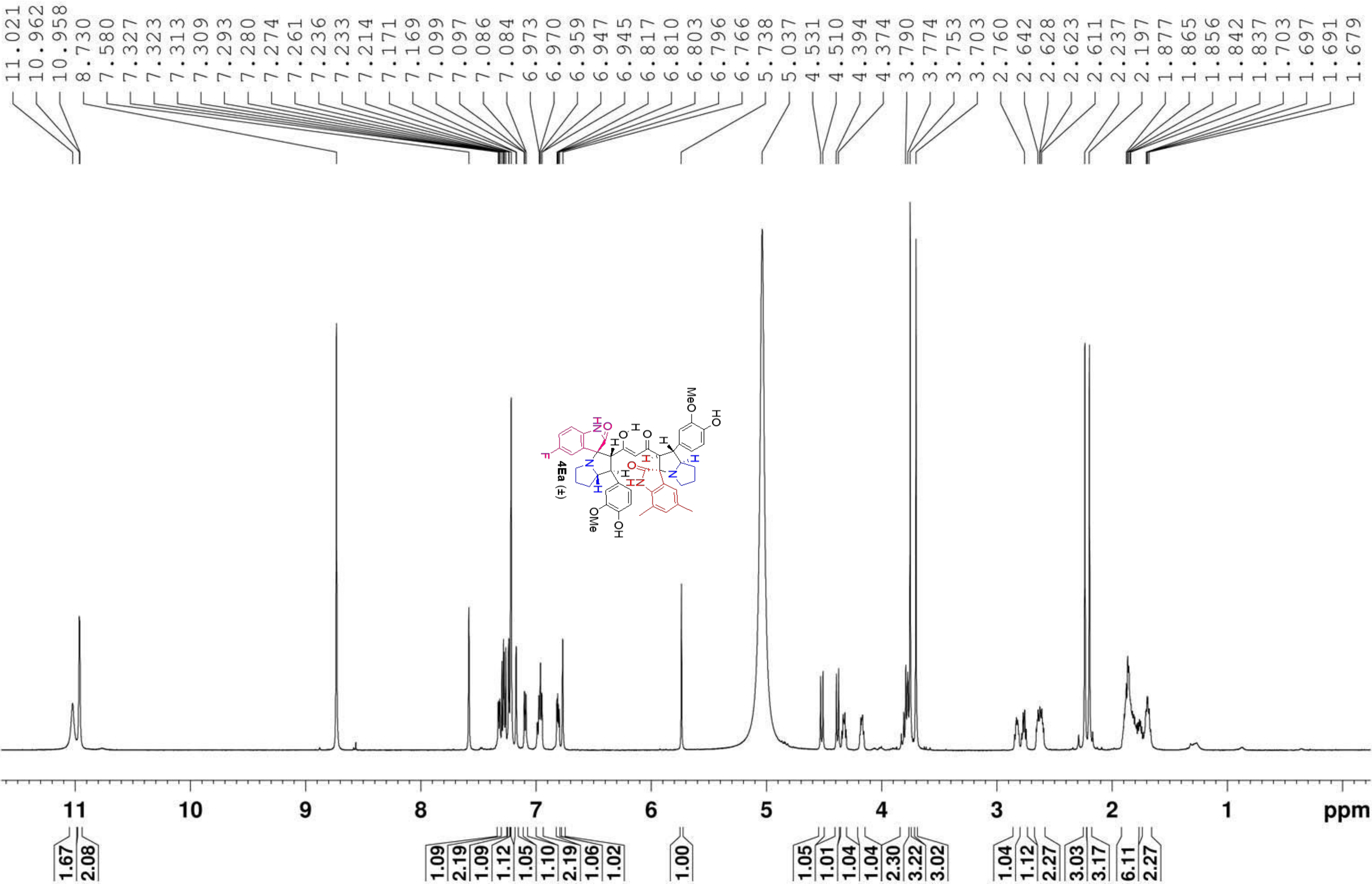

13C-NMR in Py-d5

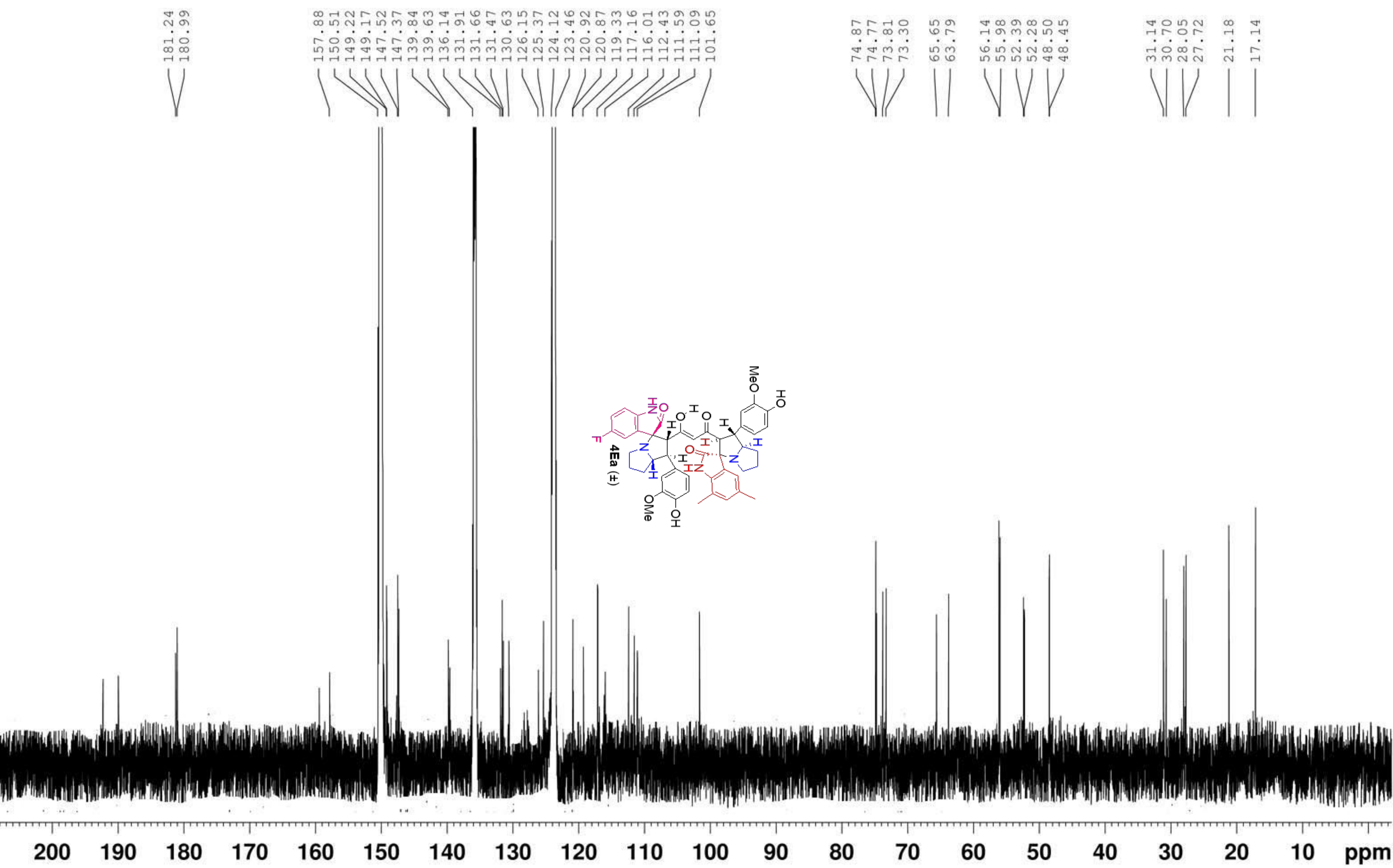

DEPT-135

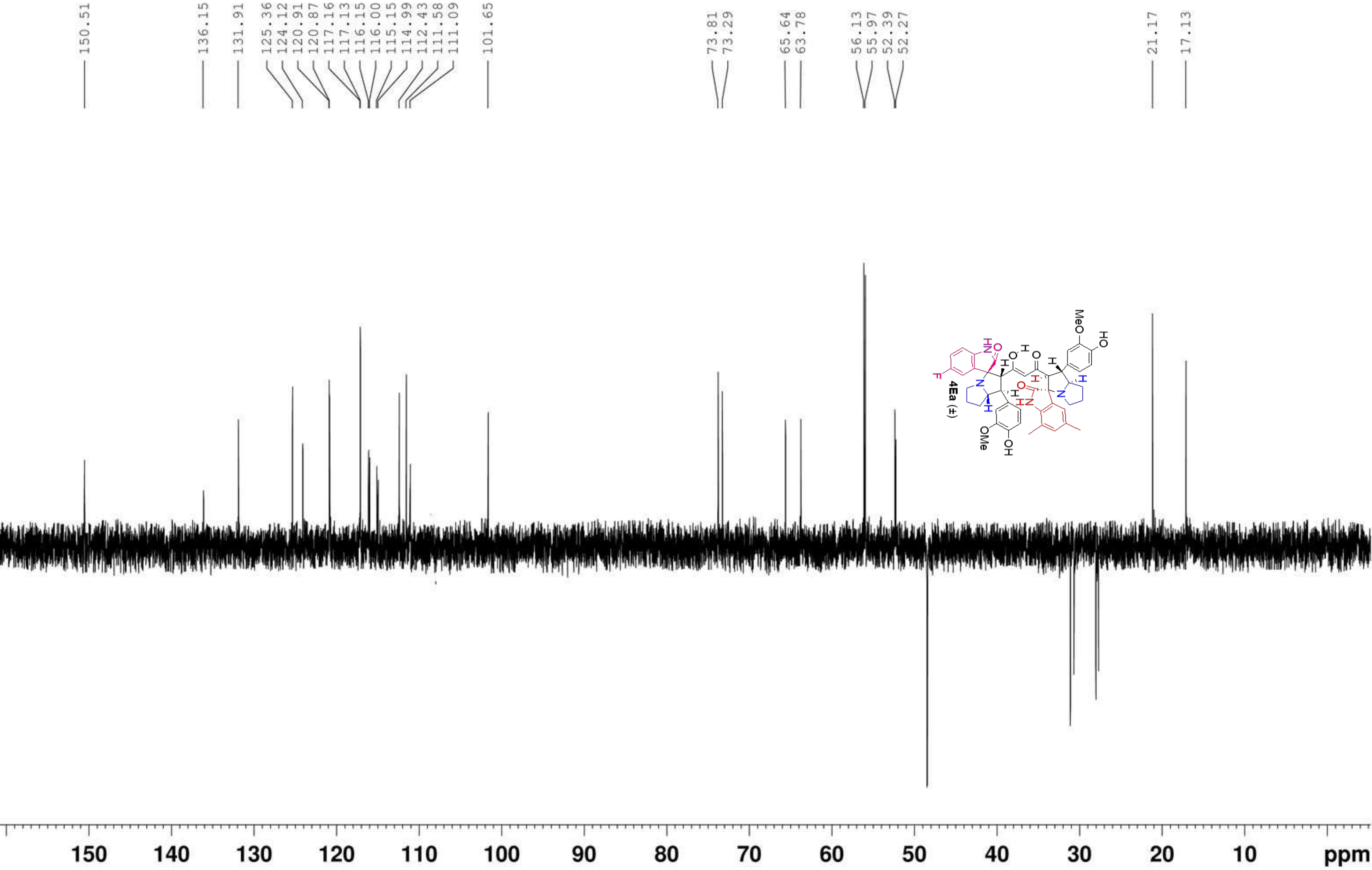
150.51
136.15
131.91
125.36
124.12
120.91
120.87
117.16
117.13
116.15
116.00
115.15
114.99
112.43
111.58
111.09
101.65
73.81
73.29
65.64
63.78
56.13
55.97
52.39
52.27
21.17
17.13
4Ea (±)
150
140
130
120
110
100
90
80
70
60
50
40
30
20
10
ppm

1H NMR in Py-d5

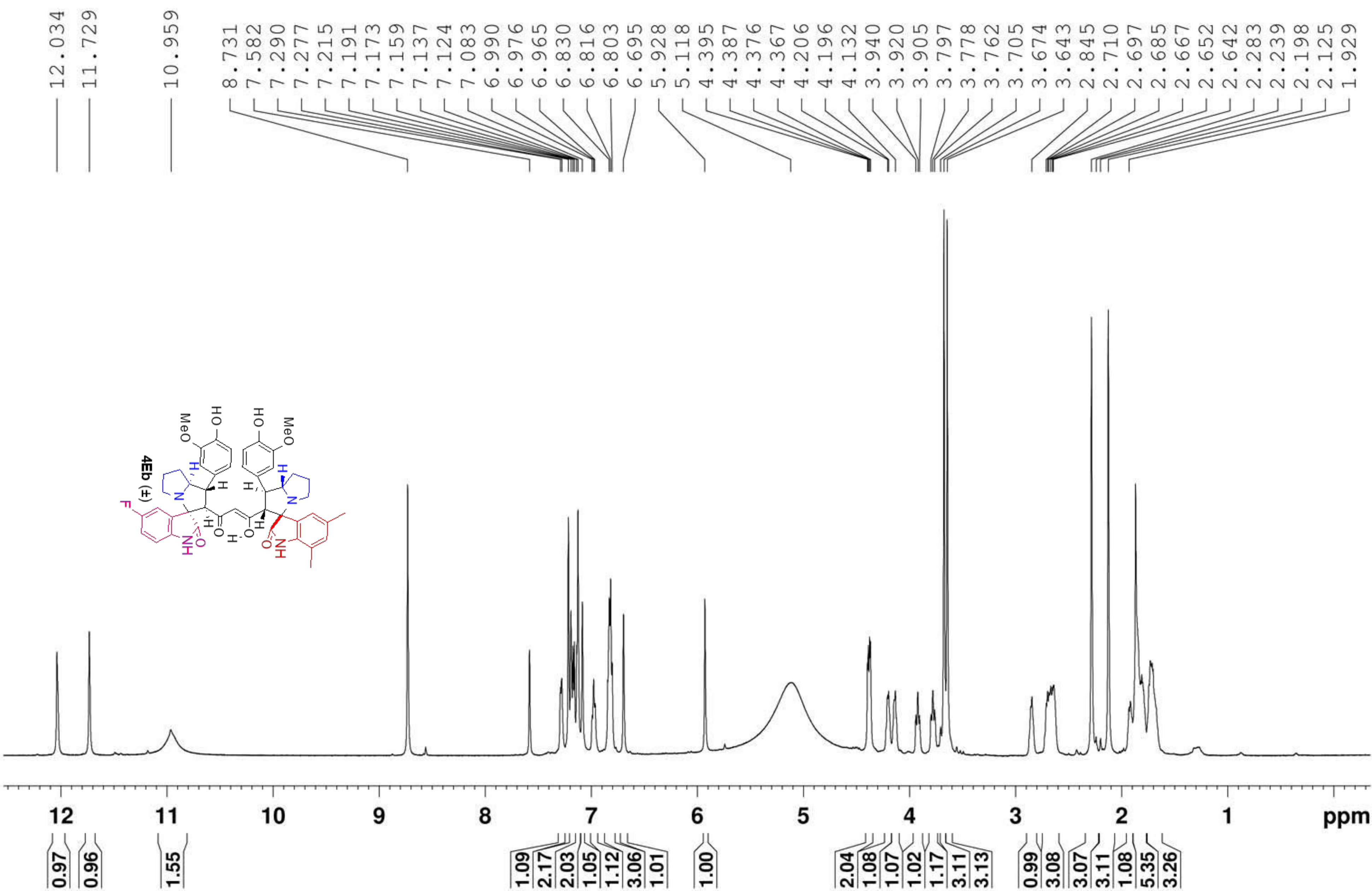

13C-NMR in Py-d5

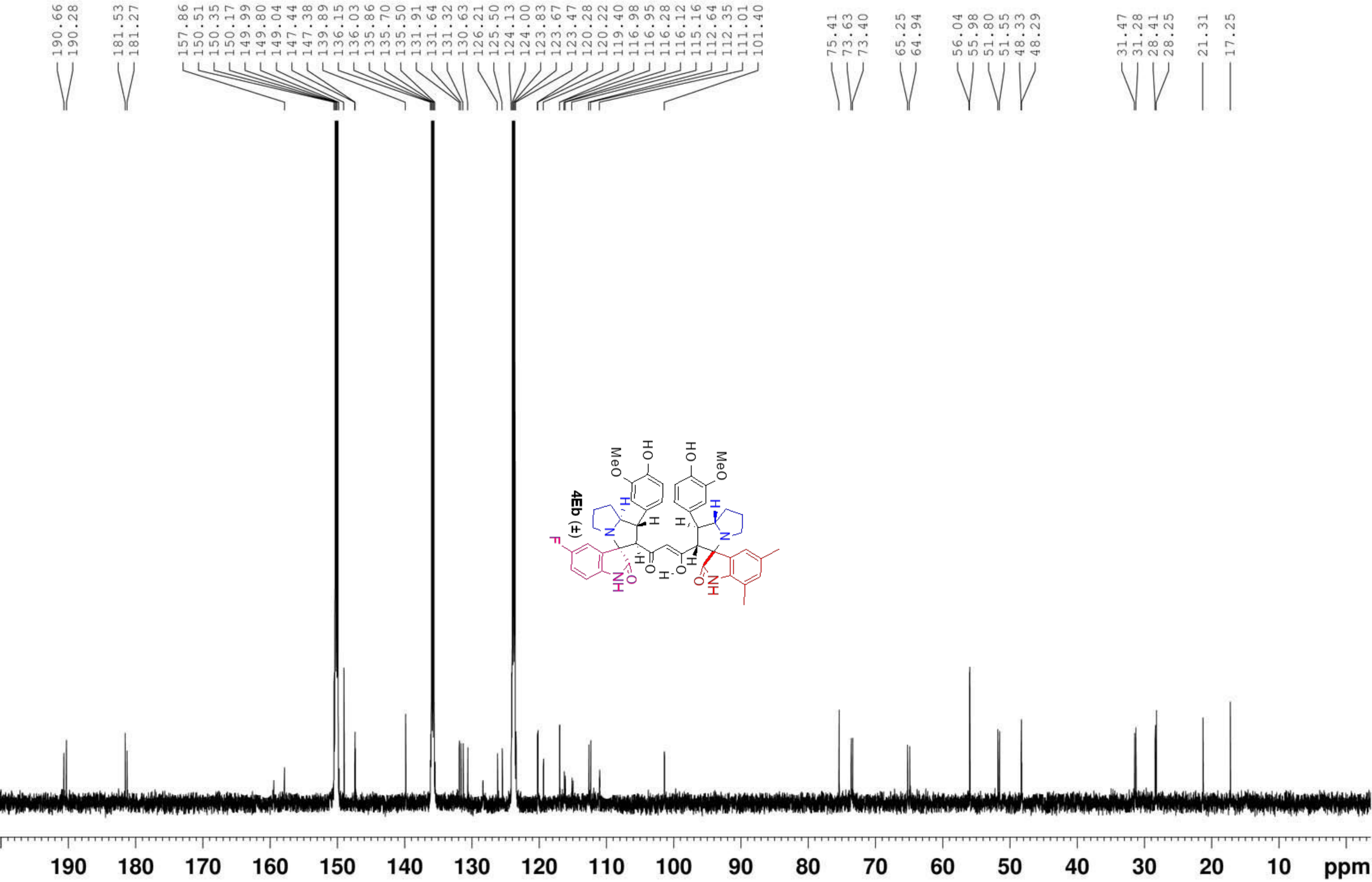

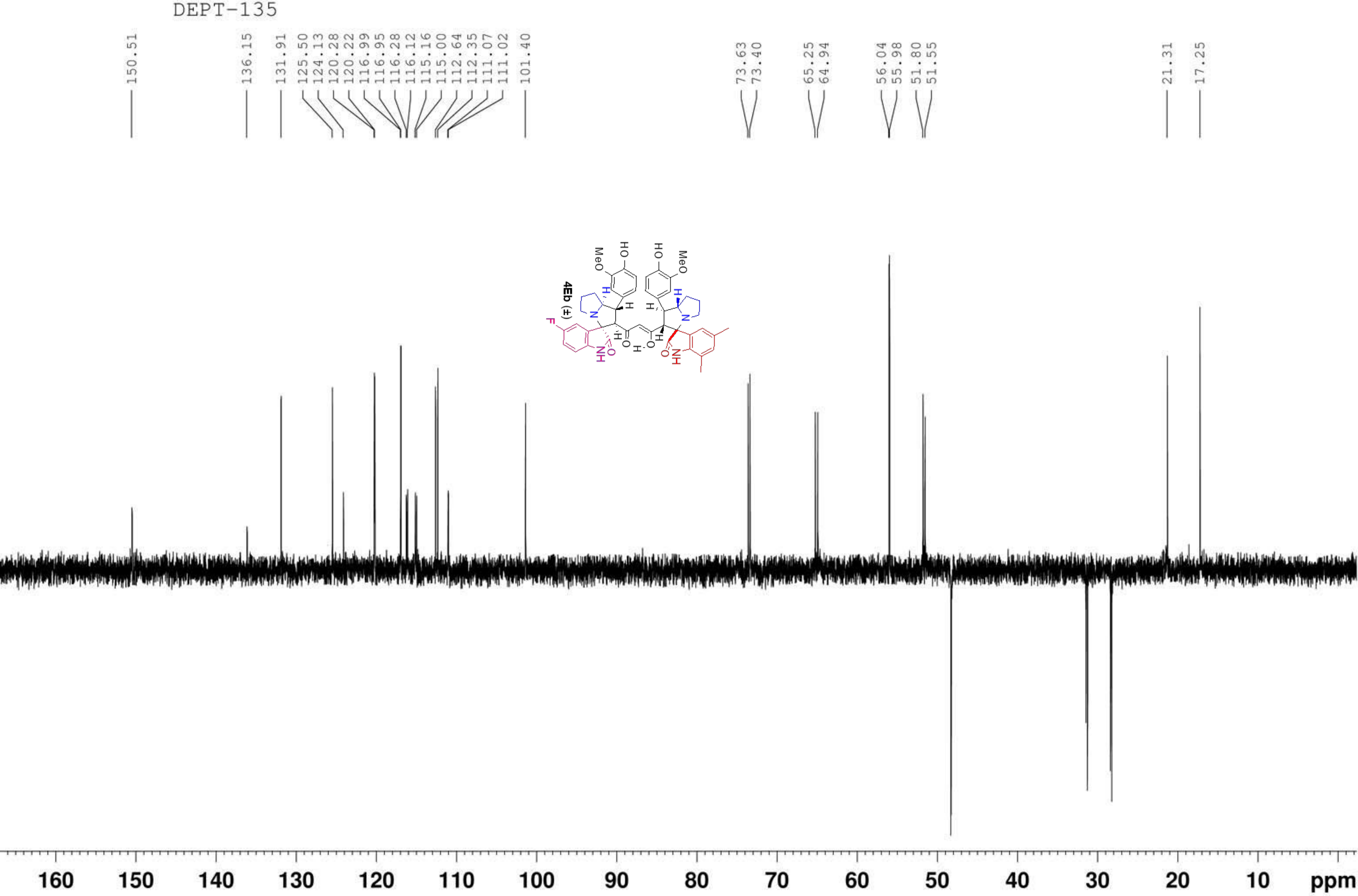
DEPT-135
150.51
136.15
131.91
125.50
124.13
120.28
120.22
116.99
116.95
116.28
116.12
115.16
115.00
112.64
112.35
111.07
111.02
101.40
73.63
73.40
65.25
64.94
56.04
55.98
51.80
51.55
21.31
17.25
MeO
HO
NH
O
4Eb (±)
F
160
150
140
130
120
110
100
90
80
70
60
50
40
30
20
10
ppm

1H NMR in Py-d5

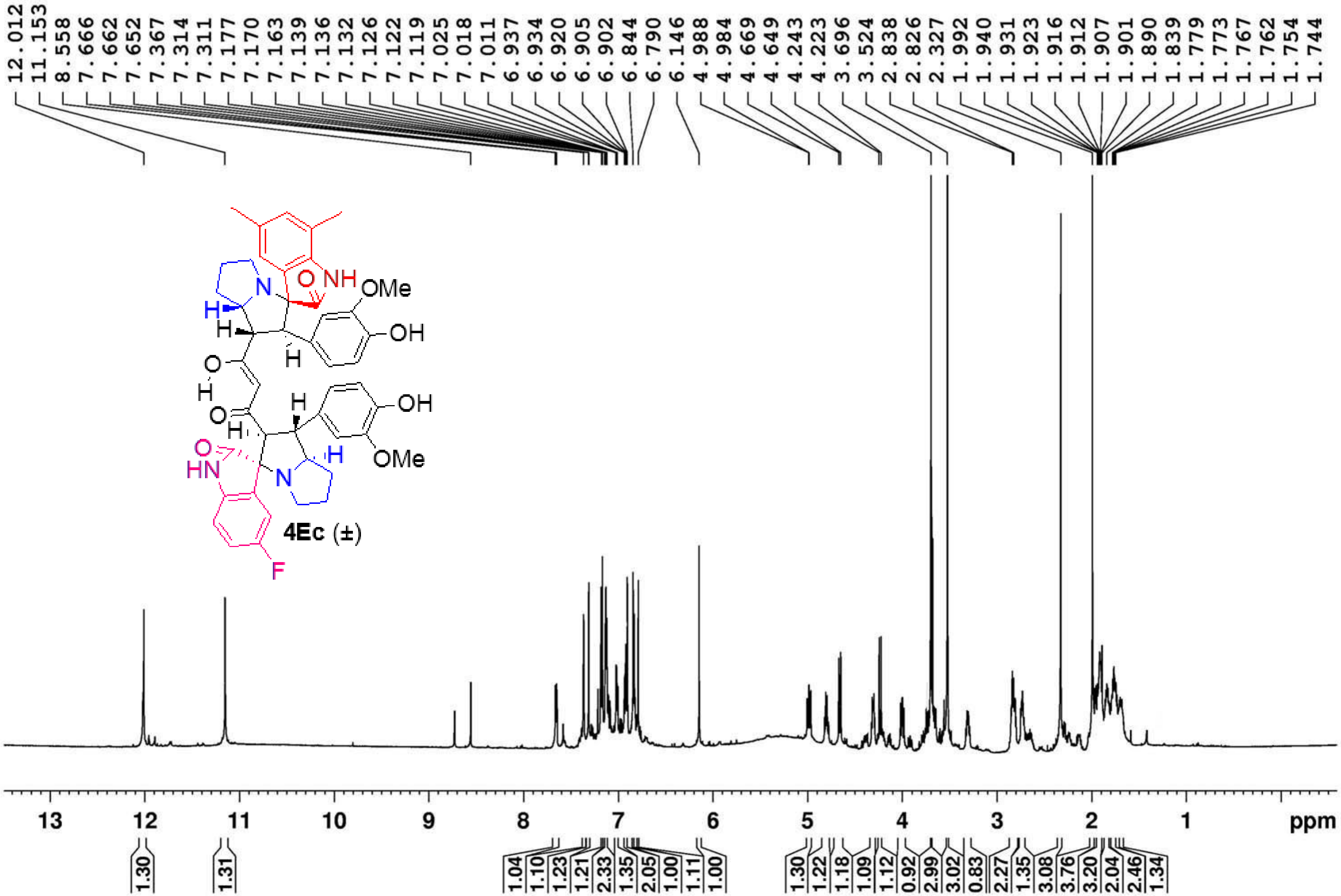

13C-NMR in Py-d5

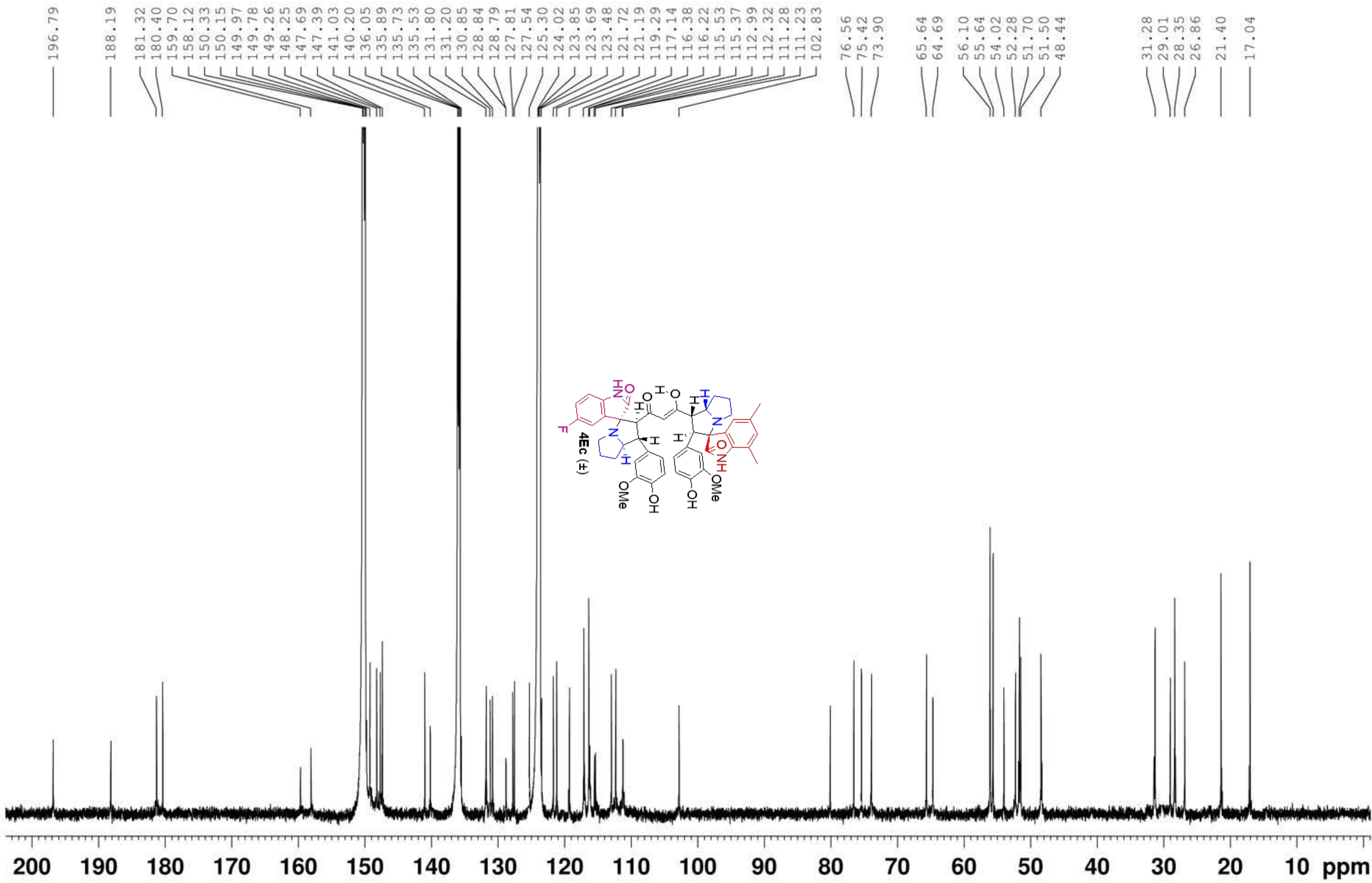

DEPT-135

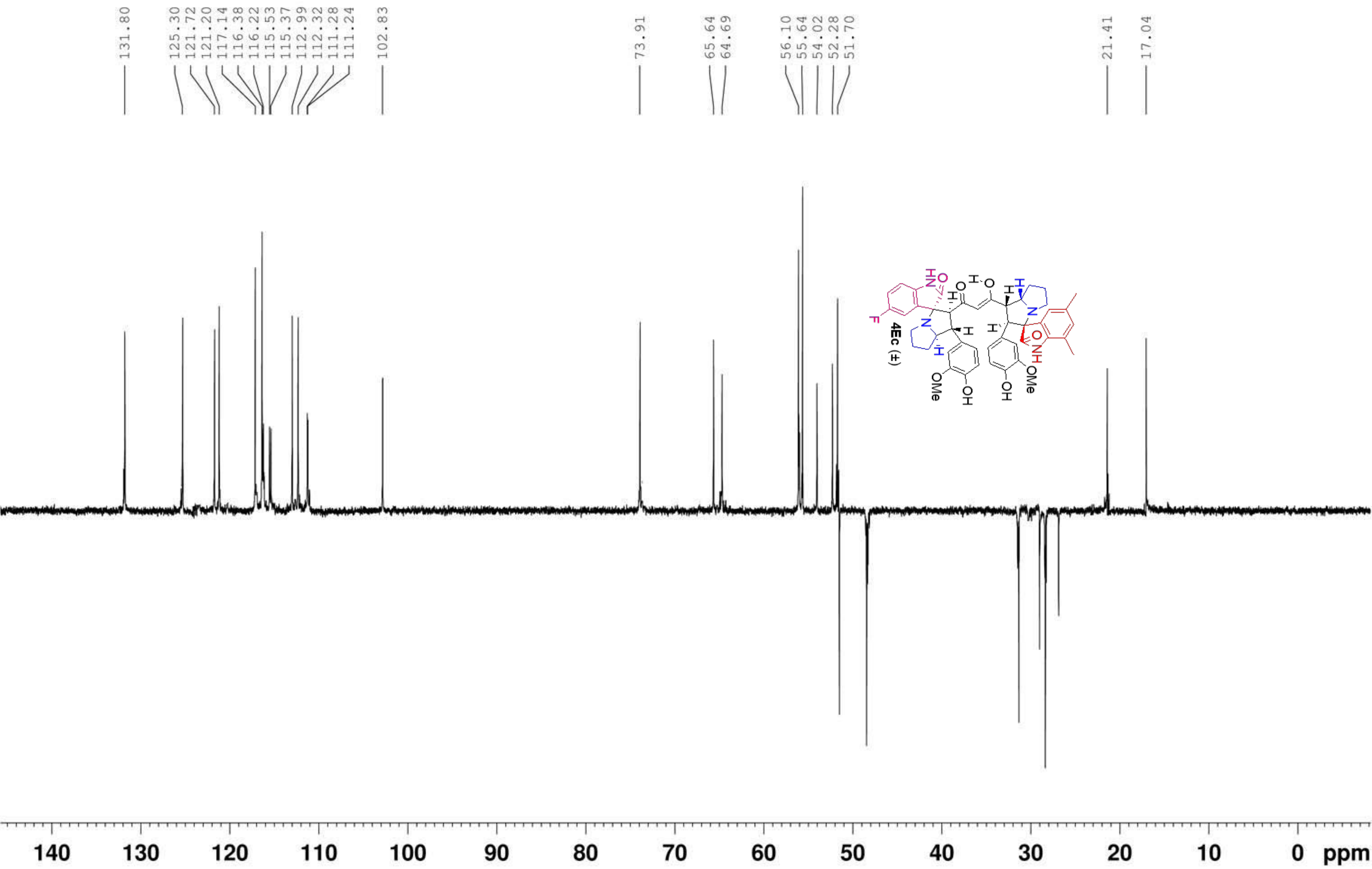
131.80
125.30
121.72
121.20
117.14
116.38
116.22
115.53
115.37
112.99
112.32
111.28
111.24
102.83
73.91
65.64
64.69
56.10
55.64
54.02
52.28
51.70
21.41
17.04
4Ec (±)
140
130
120
110
100
90
80
70
60
50
40
30
20
10
0
ppm

1H NMR in Py-d5

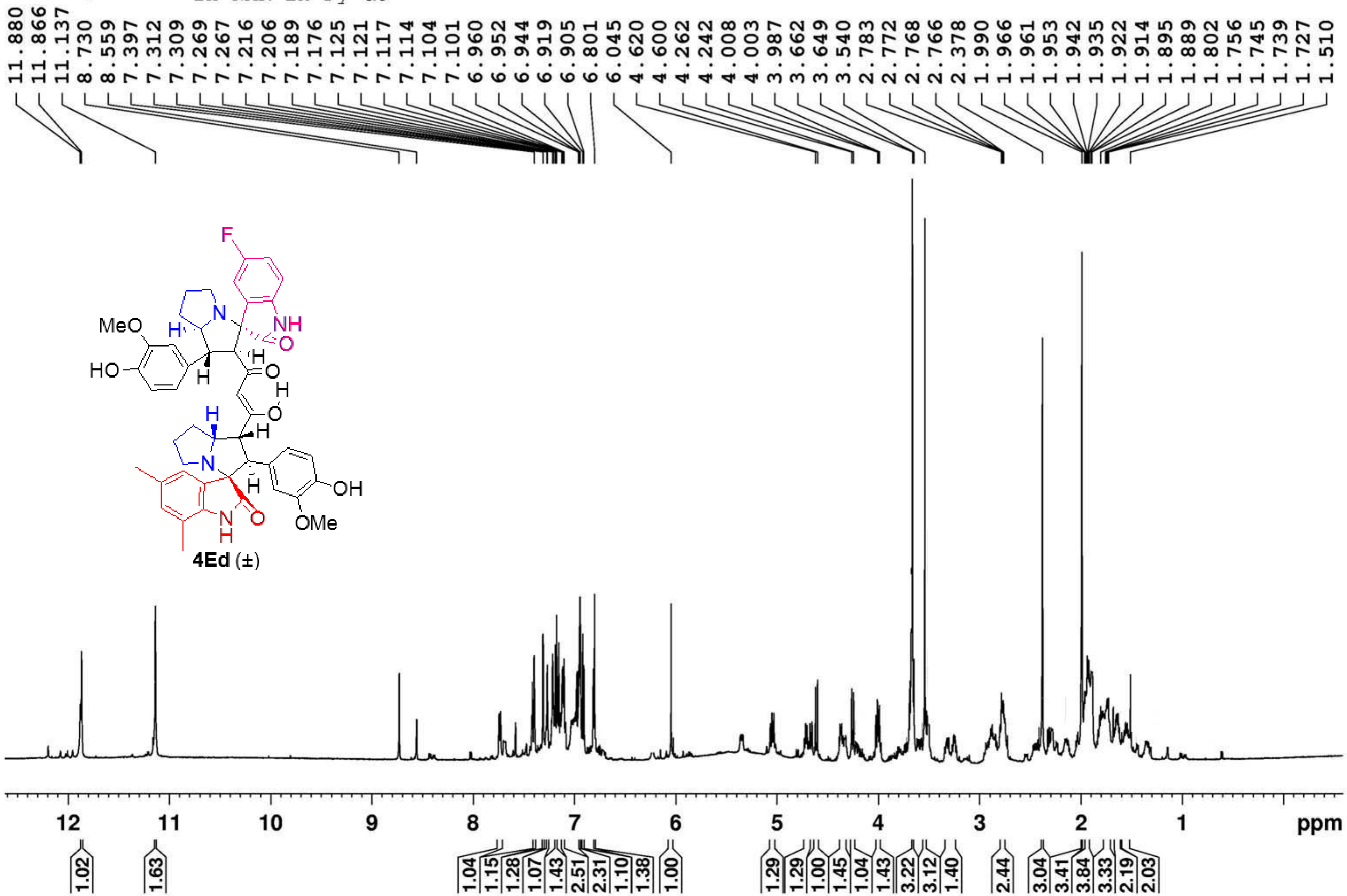

13C-NMR in Py-d5

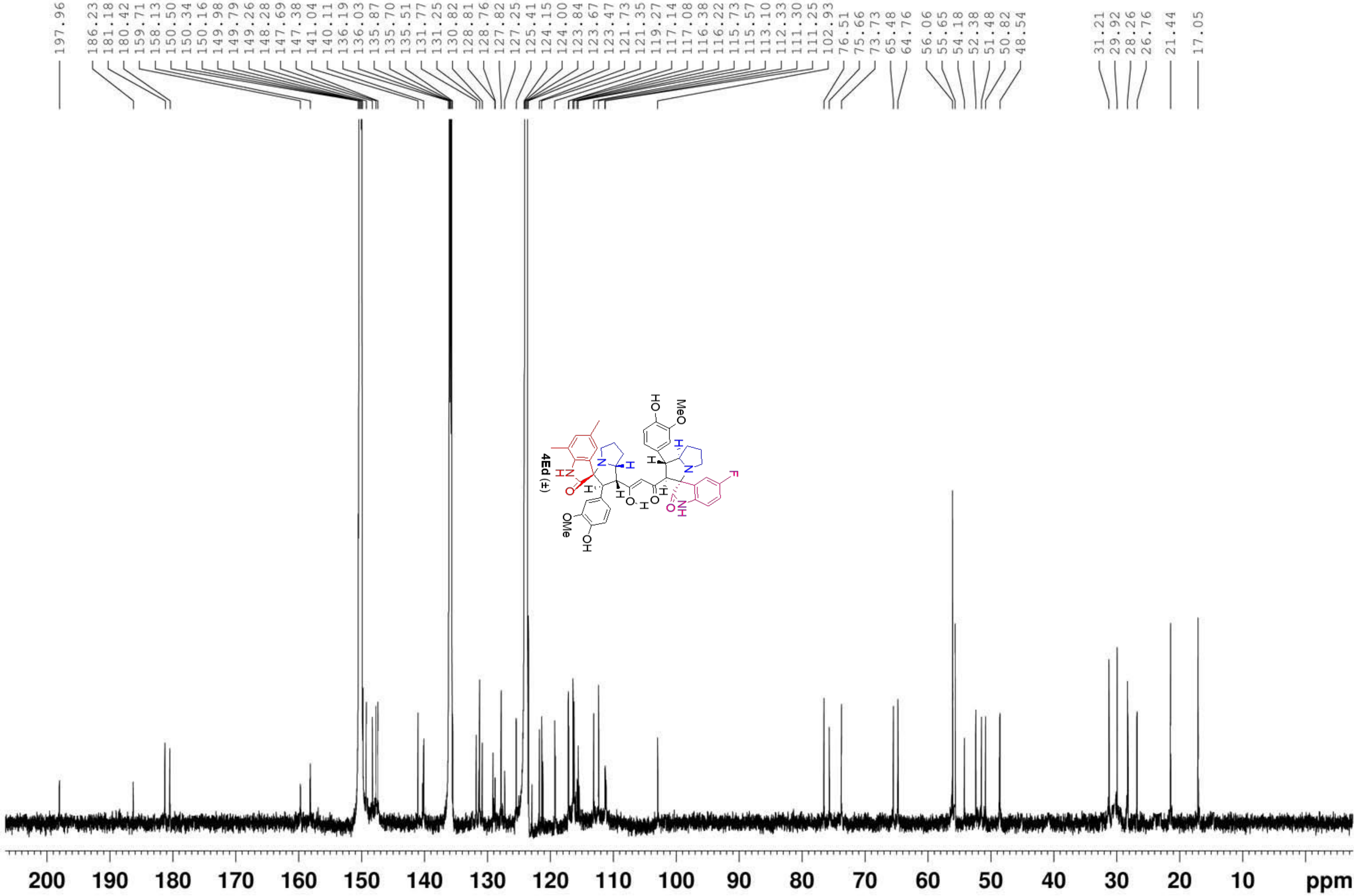

DEPT-135

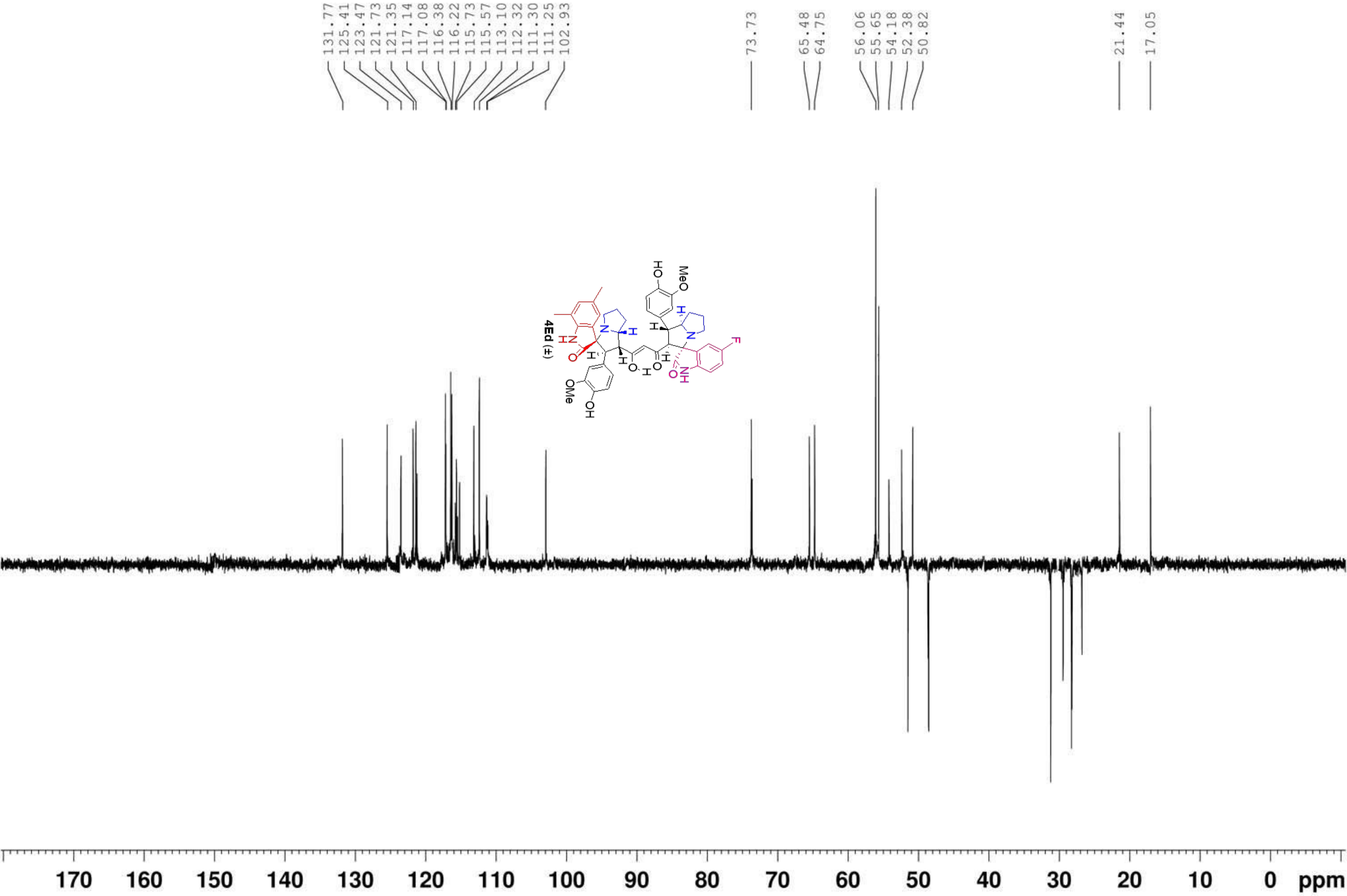
131.77
125.41
123.47
121.73
121.35
117.14
117.08
116.38
116.22
115.73
115.57
113.10
112.32
111.30
111.25
102.93
73.73
65.48
64.75
56.06
55.65
54.18
52.38
50.82
21.44
17.05
4Ed (±)
170
160
150
140
130
120
110
100
90
80
70
60
50
40
30
20
10
0
ppm

1H NMR in Py-d5

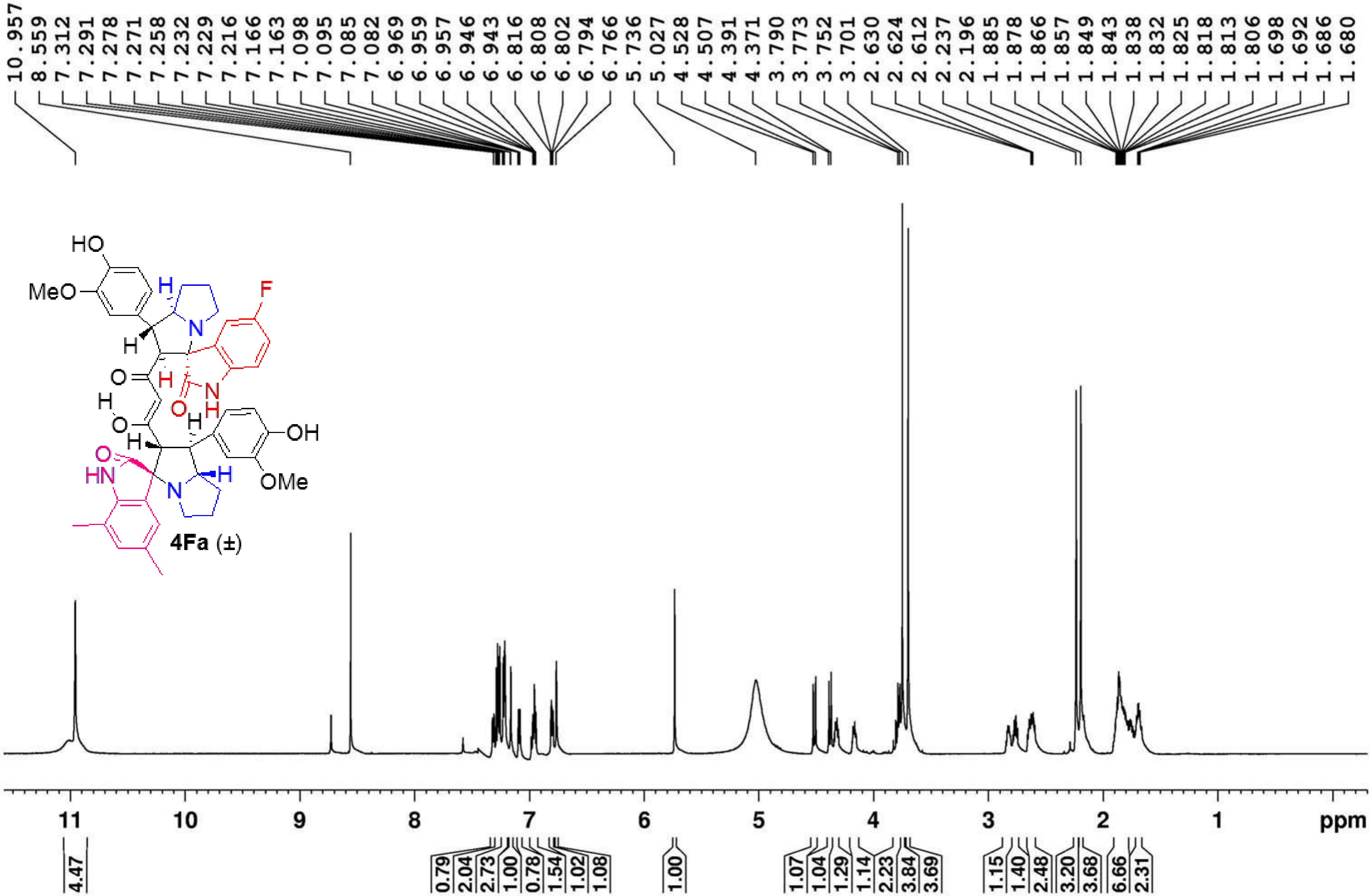

13C-NMR in Py-d5

192.29, 181.24, 180.99, 157.88, 150.36, 150.18, 150.00, 149.21, 149.16, 147.52, 147.37, 139.85, 139.62, 136.01, 135.85, 135.68, 135.50, 131.91, 131.65, 131.46, 130.62, 128.30, 126.15, 125.37, 123.99, 123.83, 123.66, 120.91, 120.87, 119.32, 117.13, 116.15, 112.43, 111.57, 111.14, 101.66, 74.87, 74.77, 73.81, 73.29, 65.65, 63.78, 56.13, 55.97, 52.39, 52.27, 48.50, 48.45, 31.14, 30.70, 28.05, 27.72, 21.17, 17.14

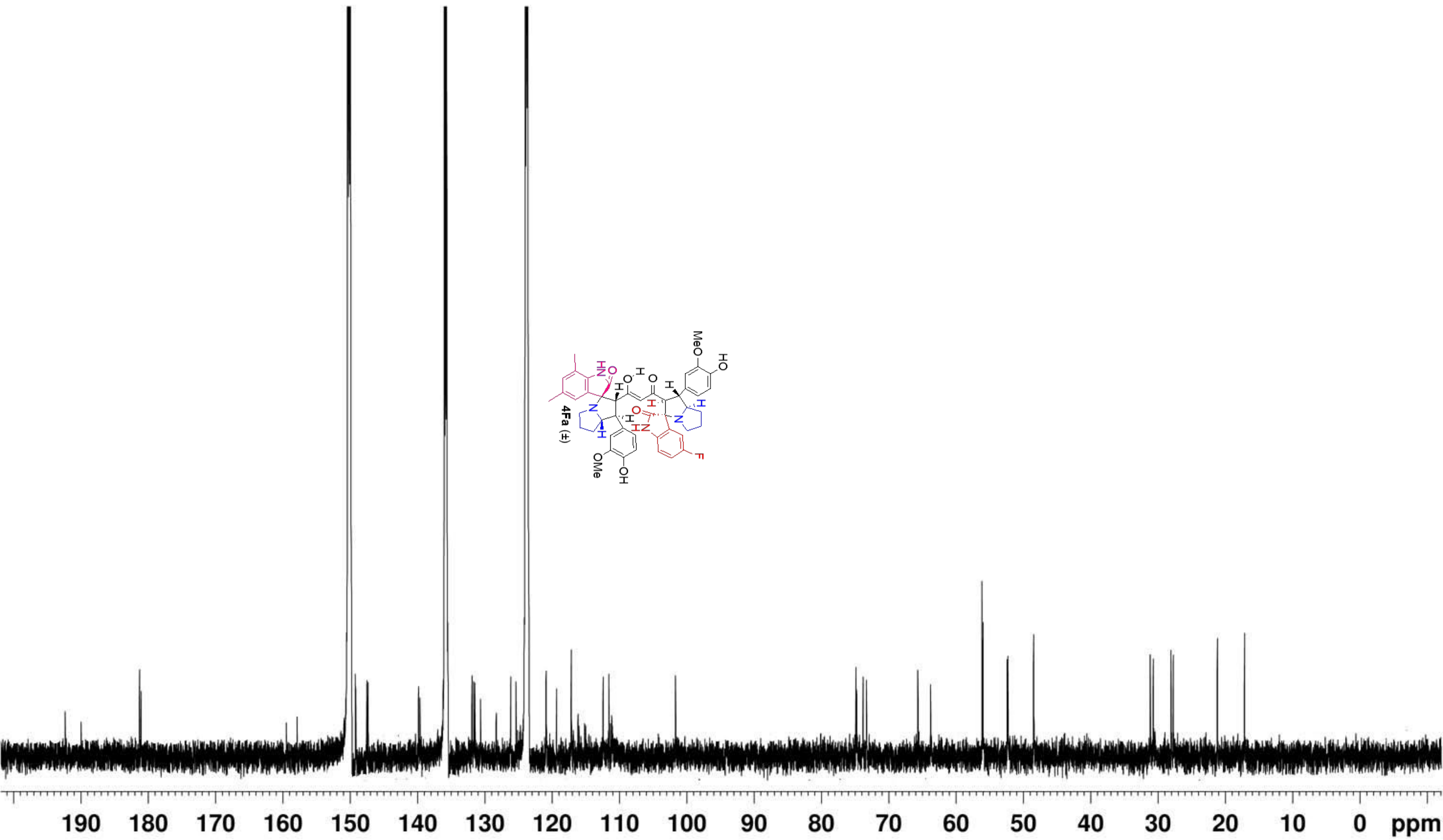

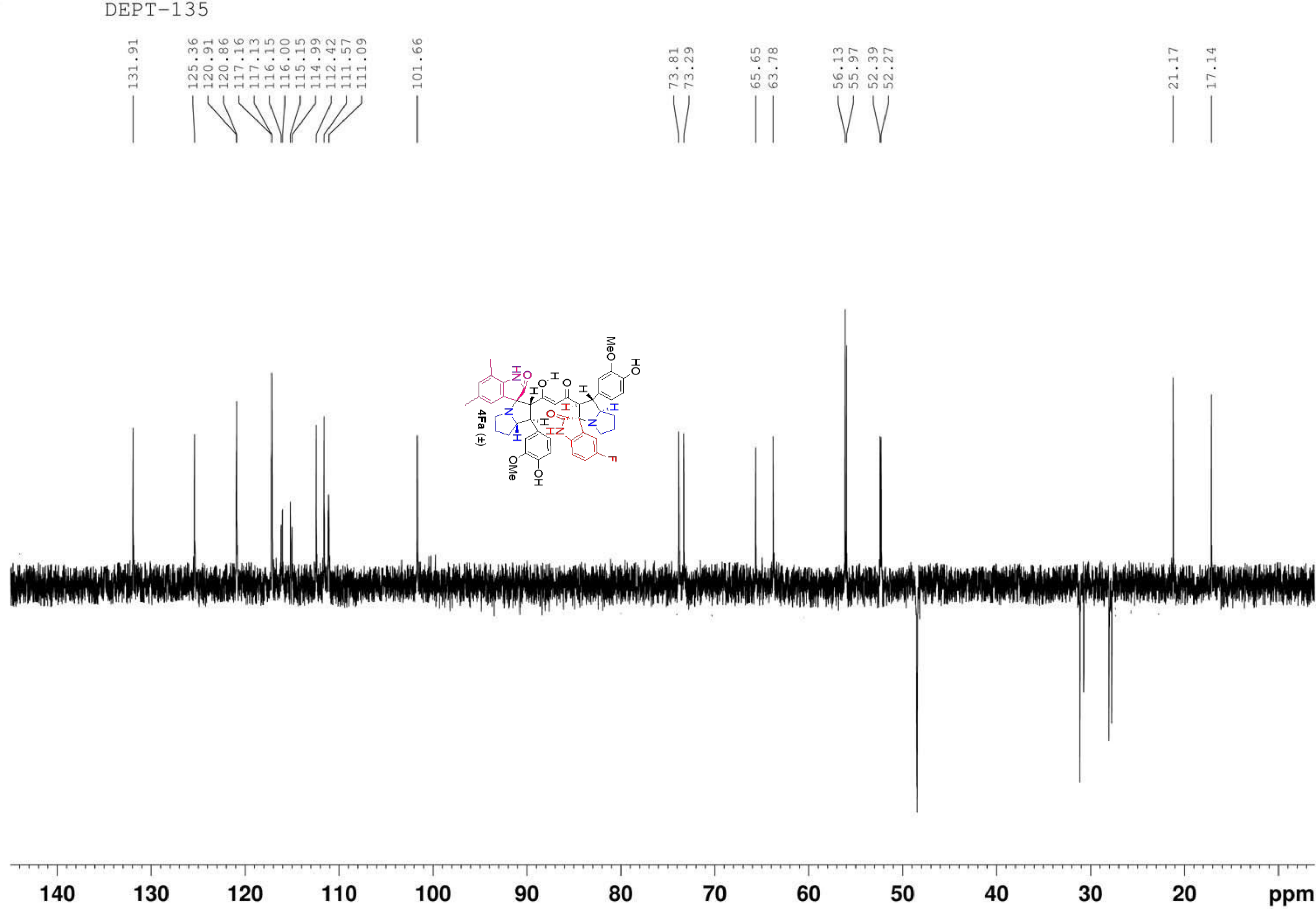
DEPT-135
131.91
125.36
120.91
120.86
117.16
117.13
116.15
116.00
115.15
114.99
112.42
111.57
111.09
101.66
73.81
73.29
65.65
63.78
56.13
55.97
52.39
52.27
21.17
17.14
4Fa (±)
140
130
120
110
100
90
80
70
60
50
40
30
20
ppm

1H NMR in Py-d5

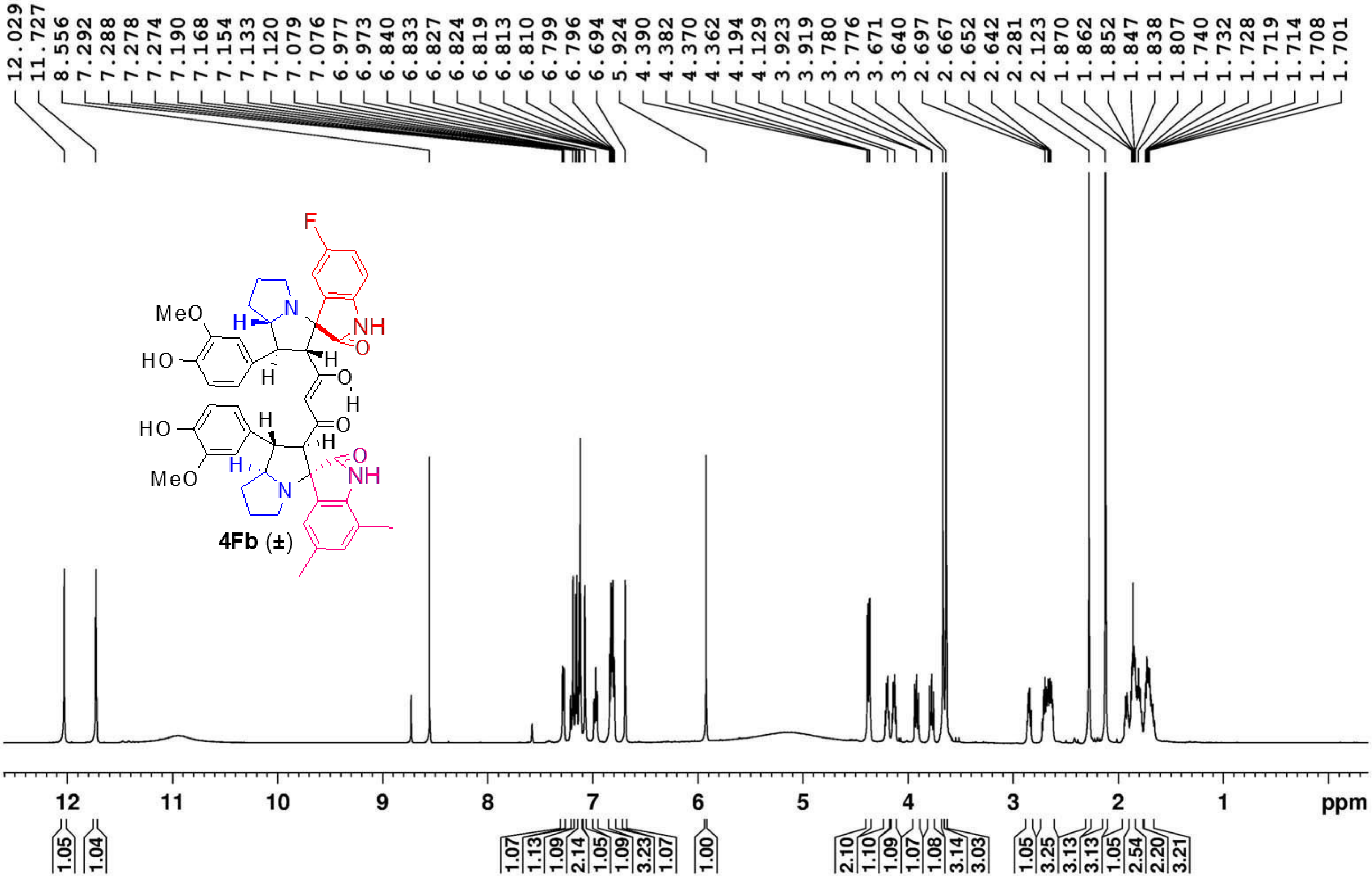

13C-NMR in Py-d5

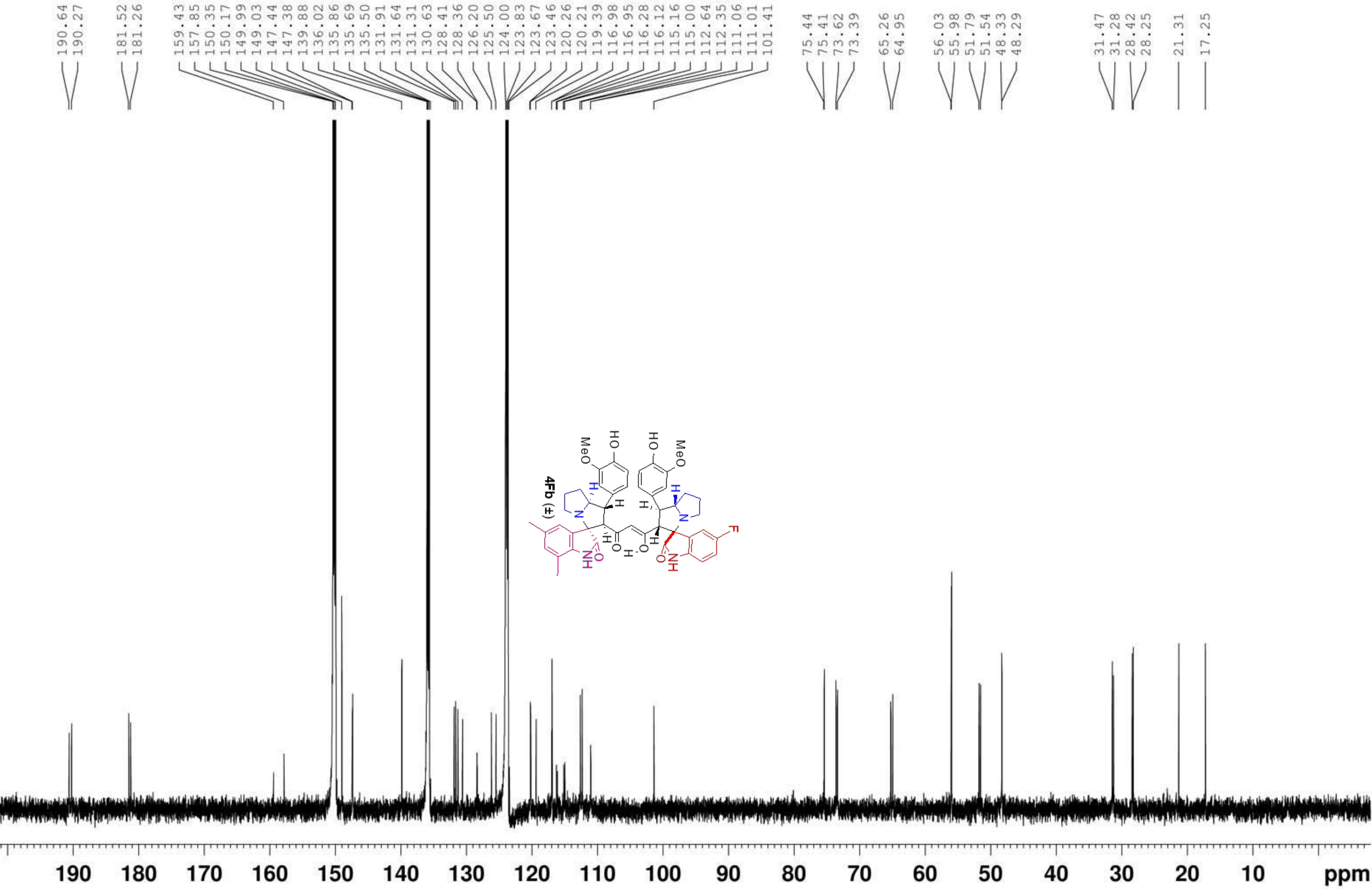

DEPT-135

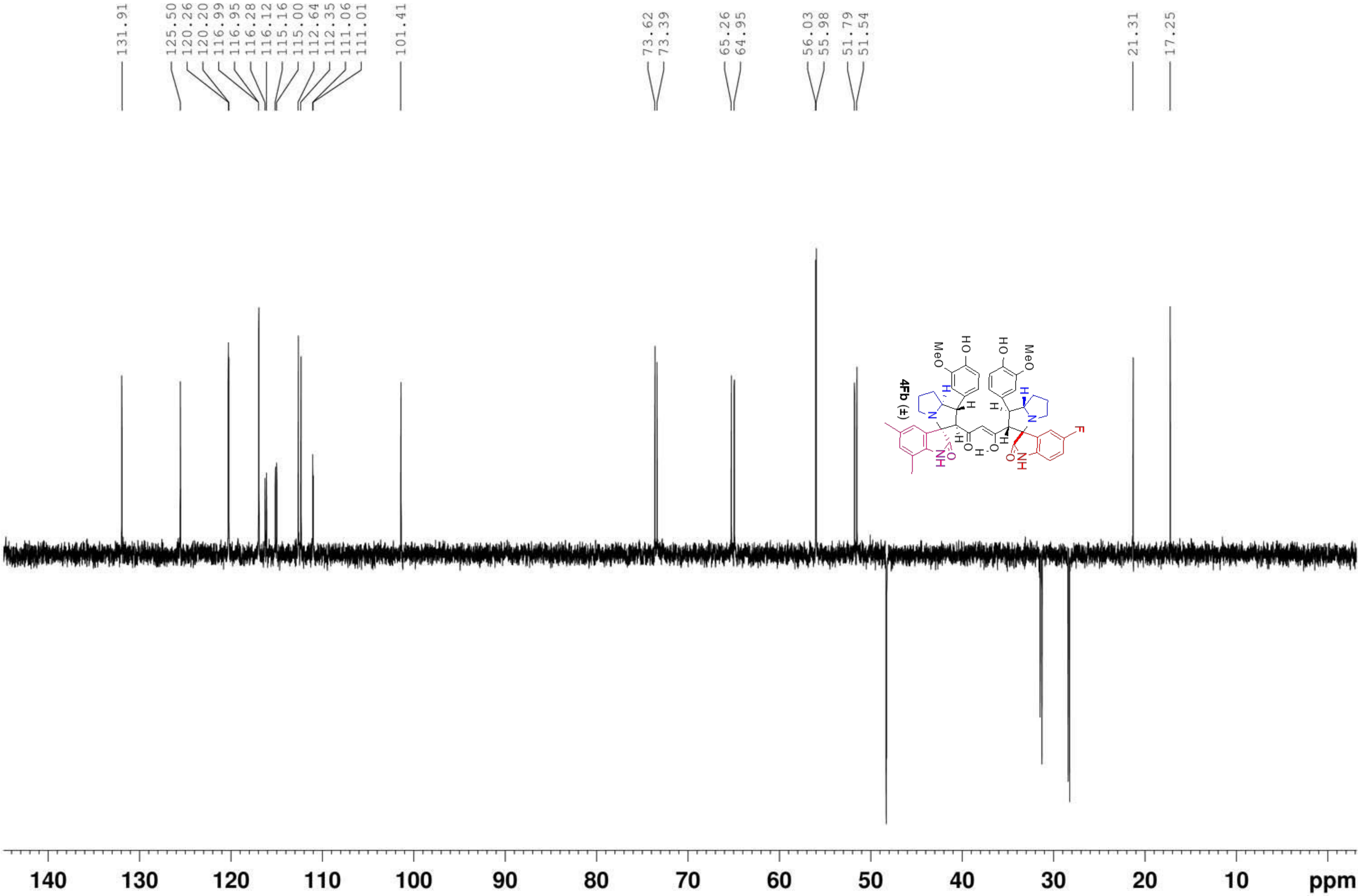
131.91
125.50
120.26
120.20
116.99
116.95
116.28
116.12
115.16
115.00
112.64
112.35
111.06
111.01
101.41
73.62
73.39
65.26
64.95
56.03
55.98
51.79
51.54
21.31
17.25
MeO
HO
F
NH
O
H
4Fb (±)
140
130
120
110
100
90
80
70
60
50
40
30
20
10
ppm

1H NMR in Py-d5

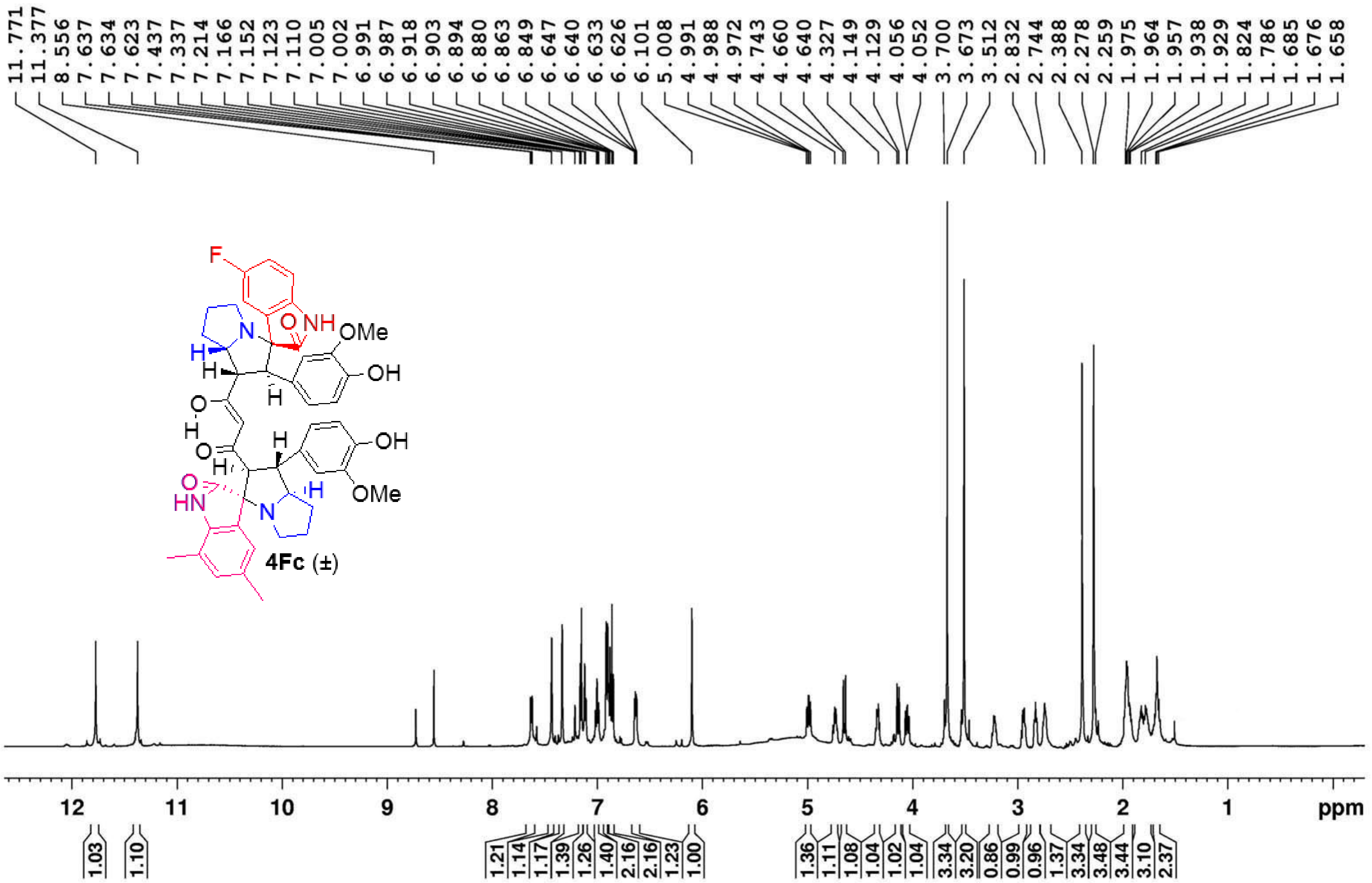

13C-NMR in Py-d5

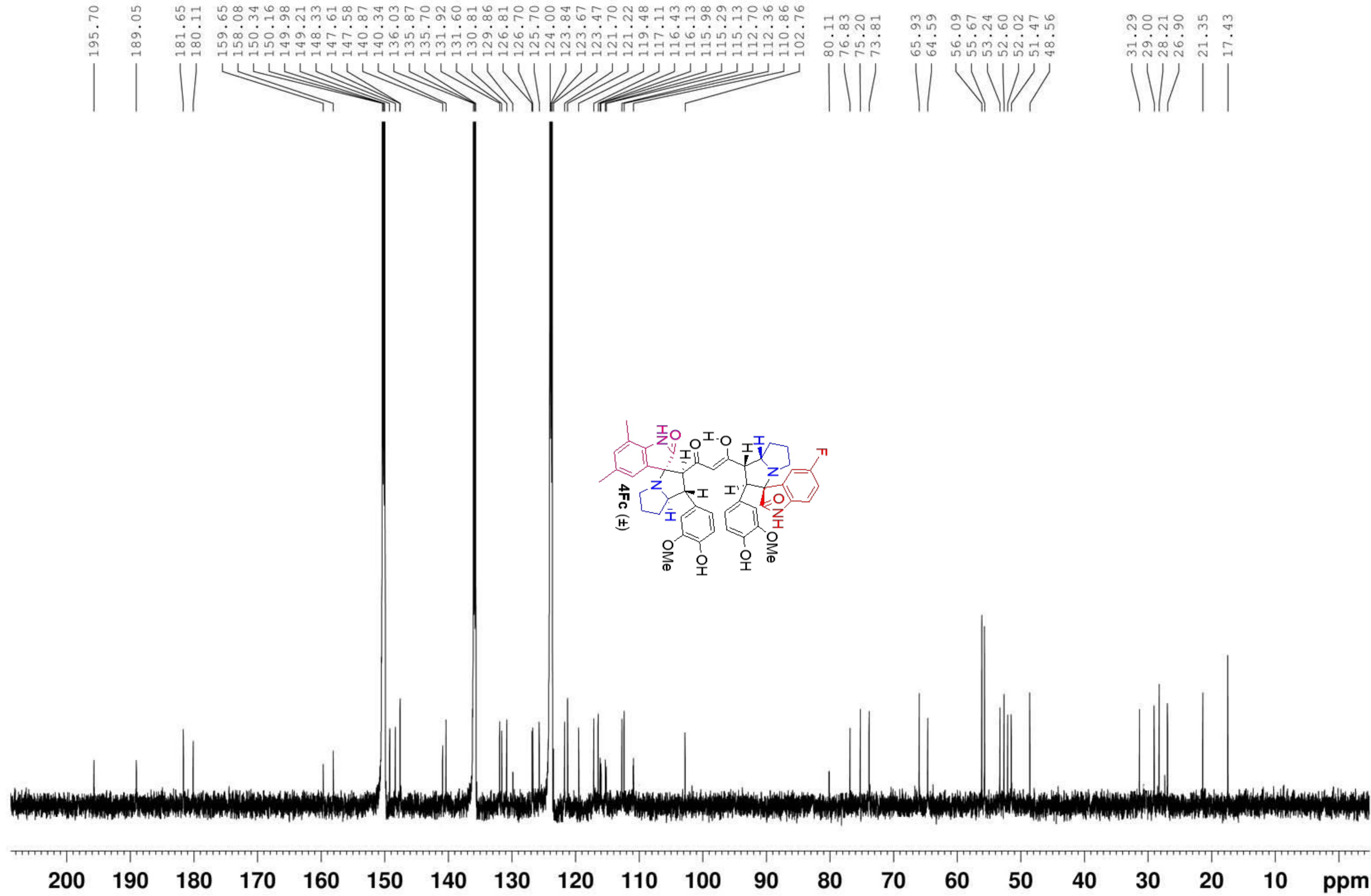

DEPT-135

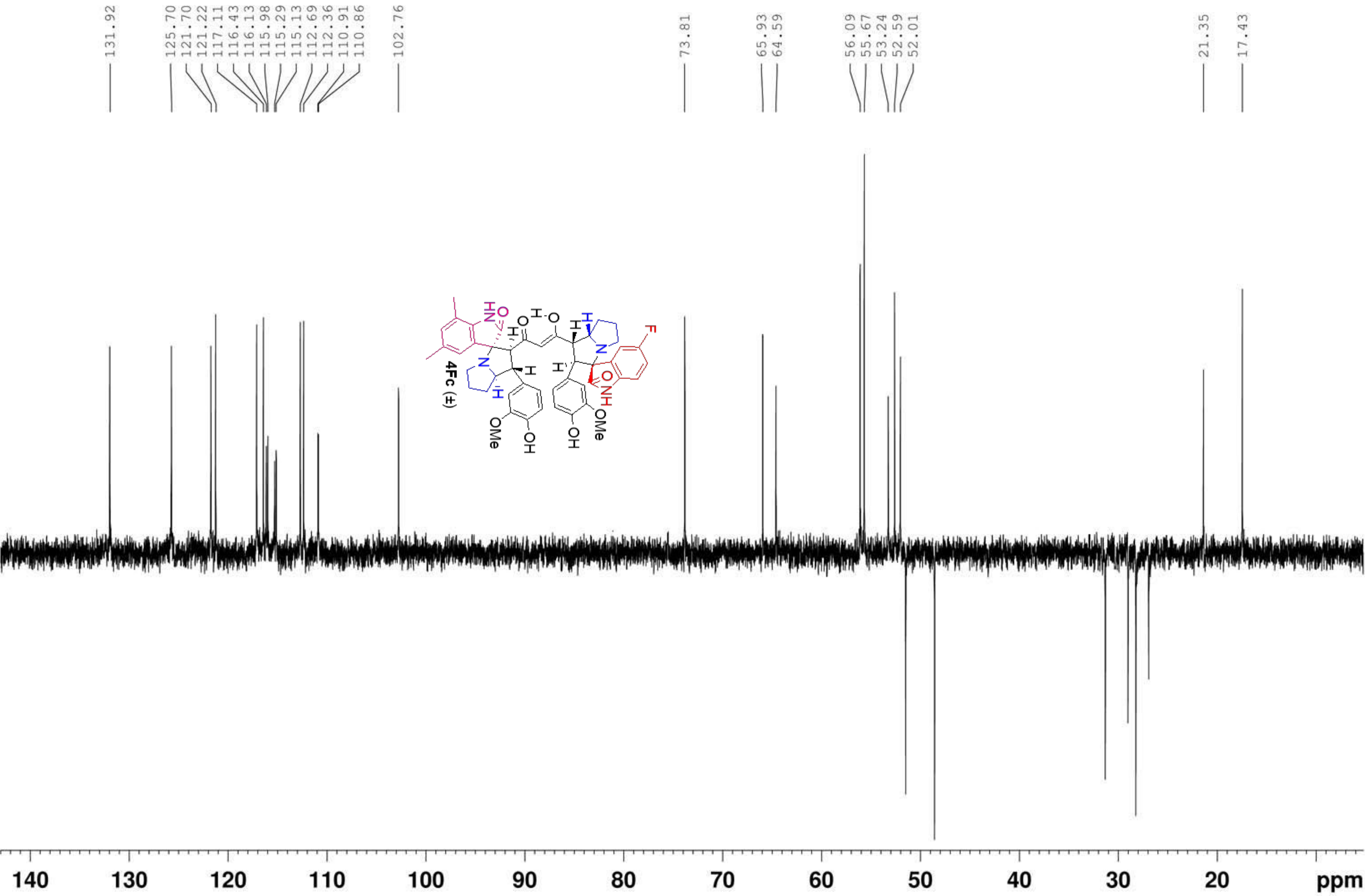
131.92
125.70
121.70
121.22
117.11
116.43
116.13
115.98
115.29
115.13
112.69
112.36
110.91
110.86
102.76
73.81
65.93
64.59
56.09
55.67
53.24
52.59
52.01
21.35
17.43
F
NH
O
OMe
OH
H
N
O
H
O
HN
OMe
4Fc (±)
140
130
120
110
100
90
80
70
60
50
40
30
20
ppm

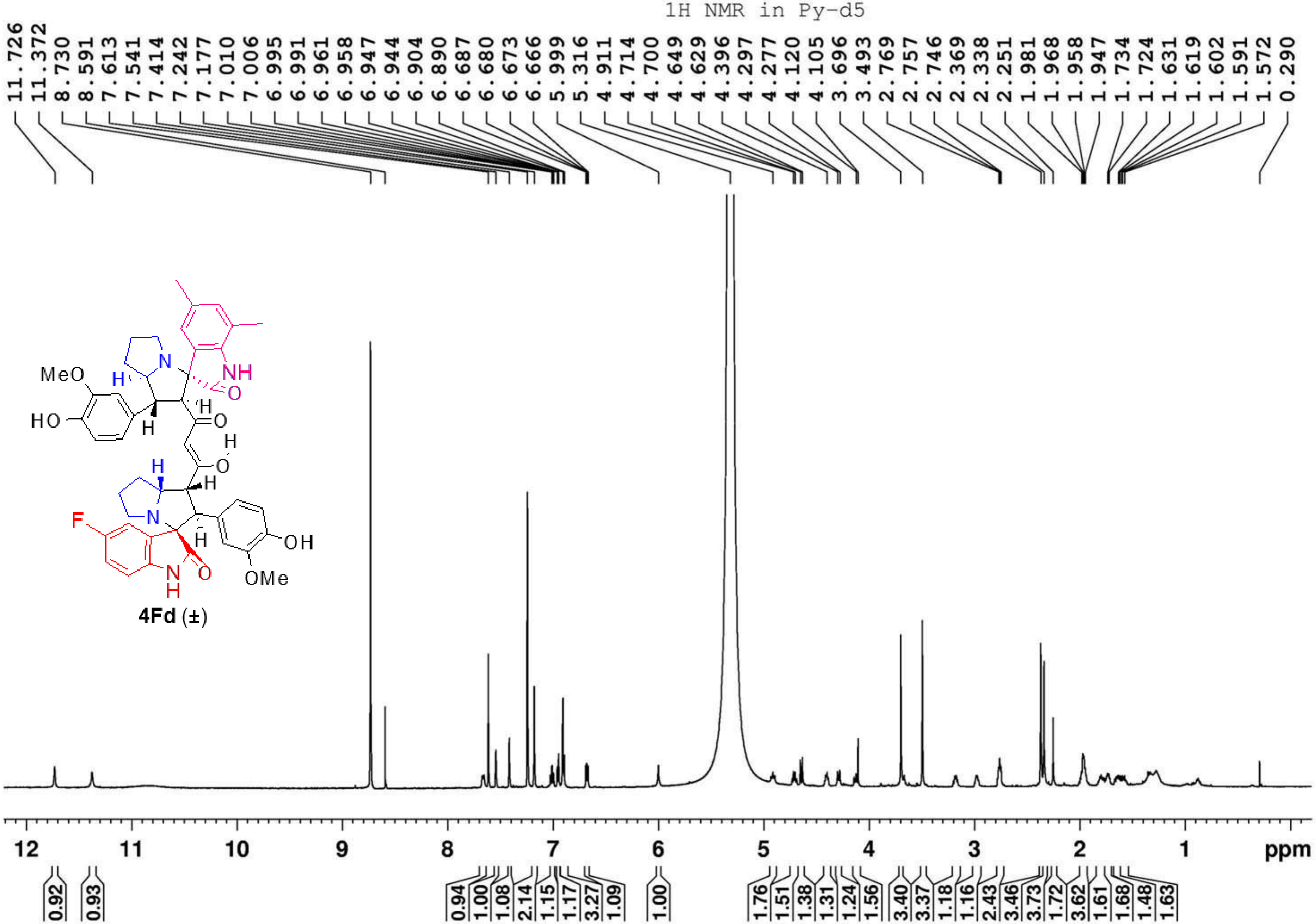

1H NMR in Py-d5
11.726
11.372
8.730
8.591
7.613
7.541
7.414
7.242
7.177
7.010
7.006
6.995
6.991
6.961
6.958
6.947
6.944
6.904
6.890
6.687
6.680
6.673
6.666
5.999
5.316
4.911
4.714
4.700
4.649
4.629
4.396
4.297
4.277
4.120
4.105
3.696
3.493
2.769
2.757
2.746
2.369
2.338
2.251
1.981
1.968
1.958
1.947
1.734
1.724
1.631
1.619
1.602
1.591
1.572
0.290
MeO
HO
H
N
NH
O
H
H
O
H
O
H
H
F
N
H
OH
N
H
O
OMe
4Fd (±)
12
11
10
9
8
7
6
5
4
3
2
1
ppm
0.92
0.93
0.94
1.00
1.08
2.14
1.15
1.17
3.27
1.09
1.00
1.76
1.51
1.38
1.31
1.24
1.56
3.40
3.37
1.18
1.16
2.43
3.46
3.73
1.72
3.62
1.61
1.68
1.48
1.63

13C-NMR in Py-d5

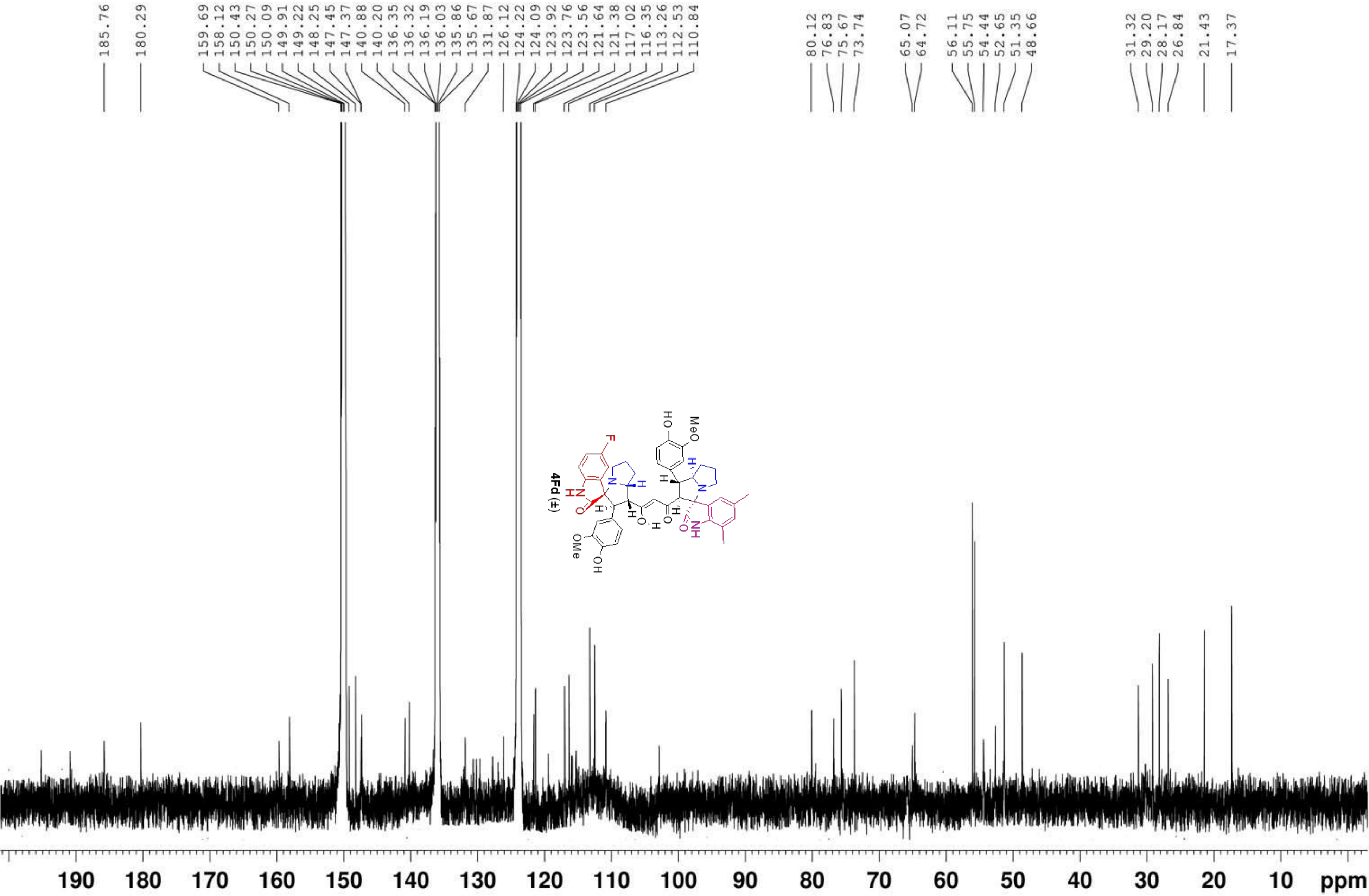

DEPT-135

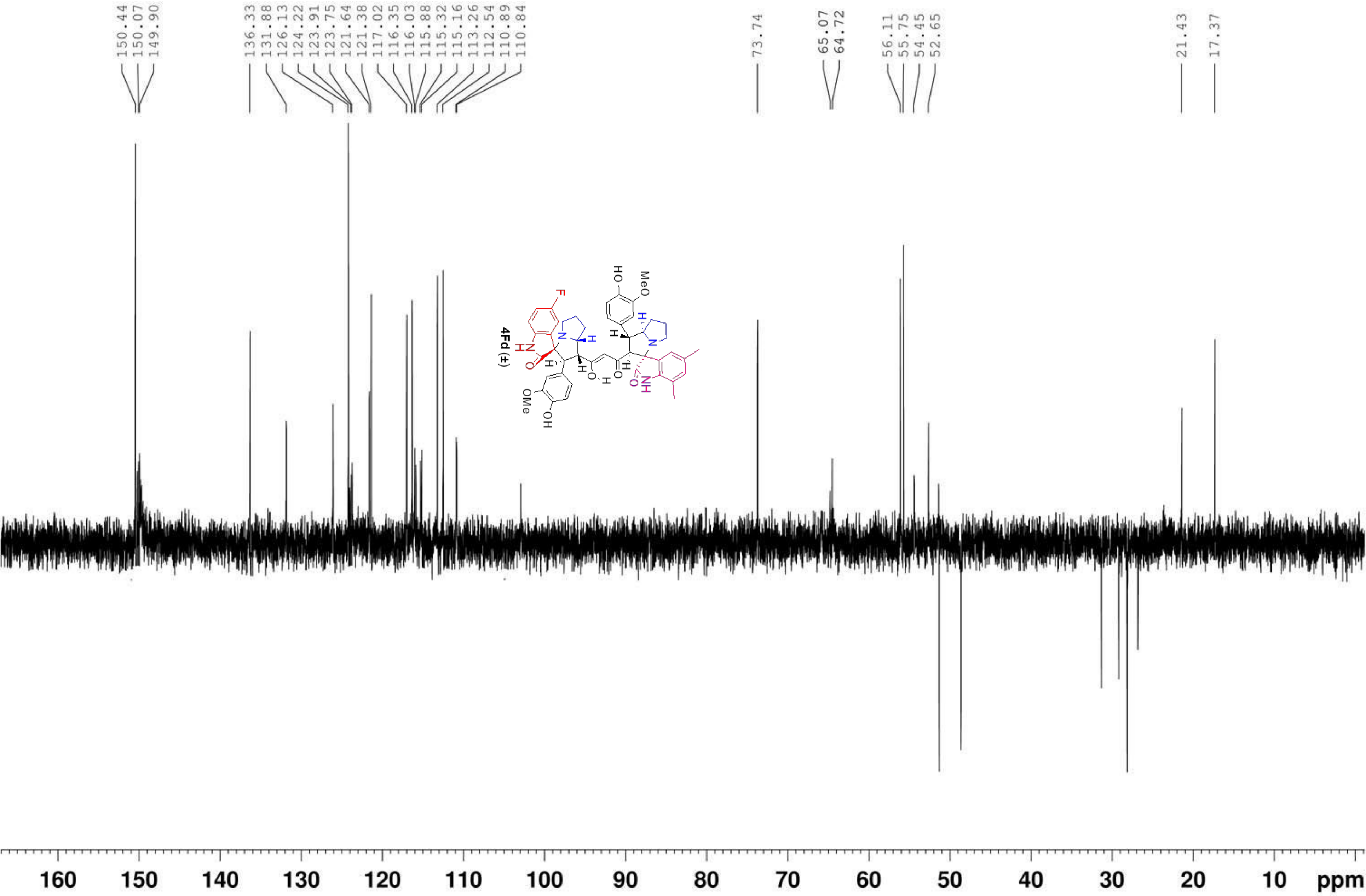
150.44
150.07
149.90
136.33
131.88
126.13
124.22
123.91
123.75
121.64
121.38
117.02
116.35
116.03
115.88
115.32
115.16
113.26
112.54
110.89
110.84
73.74
65.07
64.72
56.11
55.75
54.45
52.65
21.43
17.37
MeO
HO
NH
O
H
OH
OMe
F
N
4Fd (±)
160
150
140
130
120
110
100
90
80
70
60
50
40
30
20
10
ppm

1H NMR in Py-d5

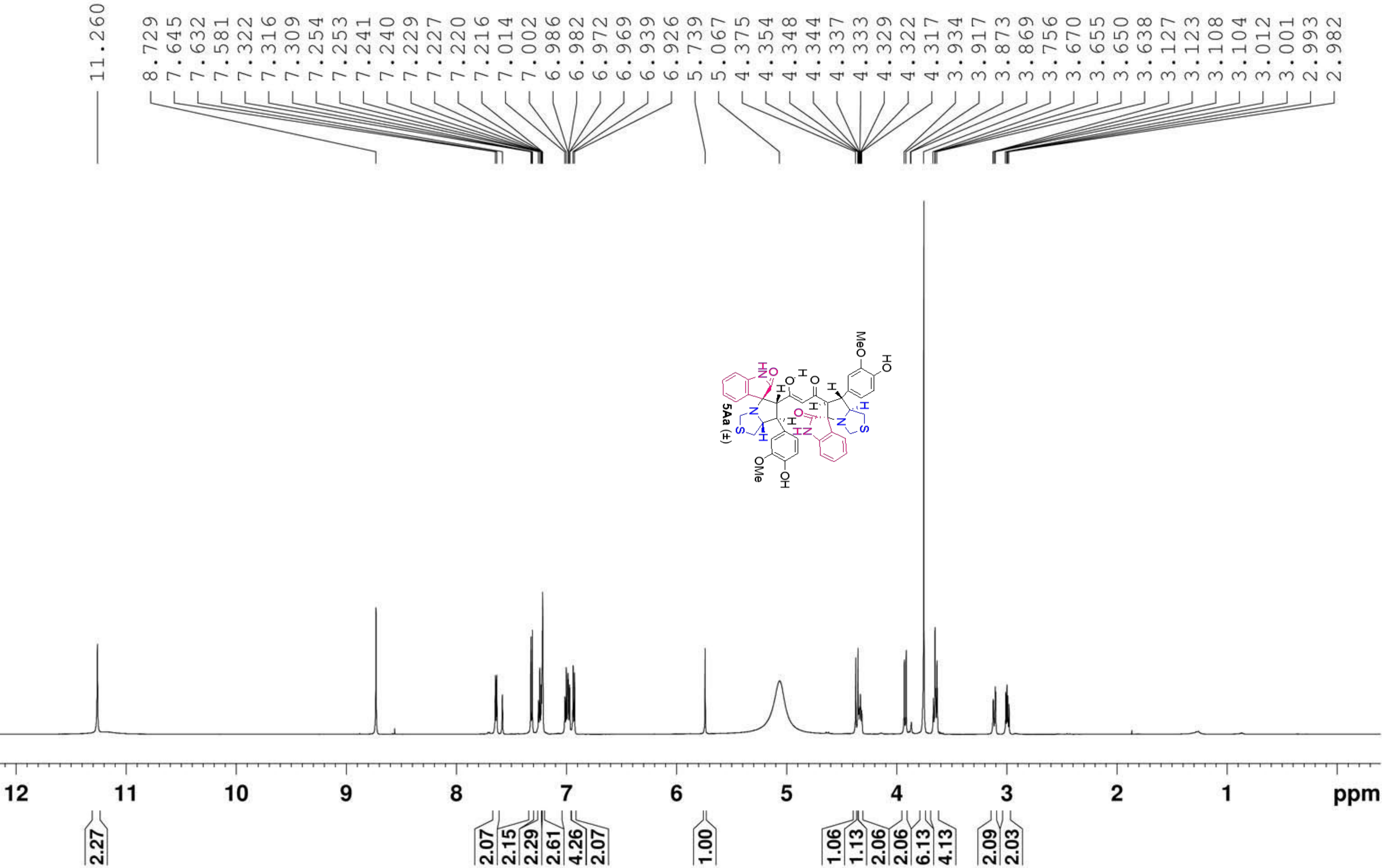

13C-NMR in Py-d5

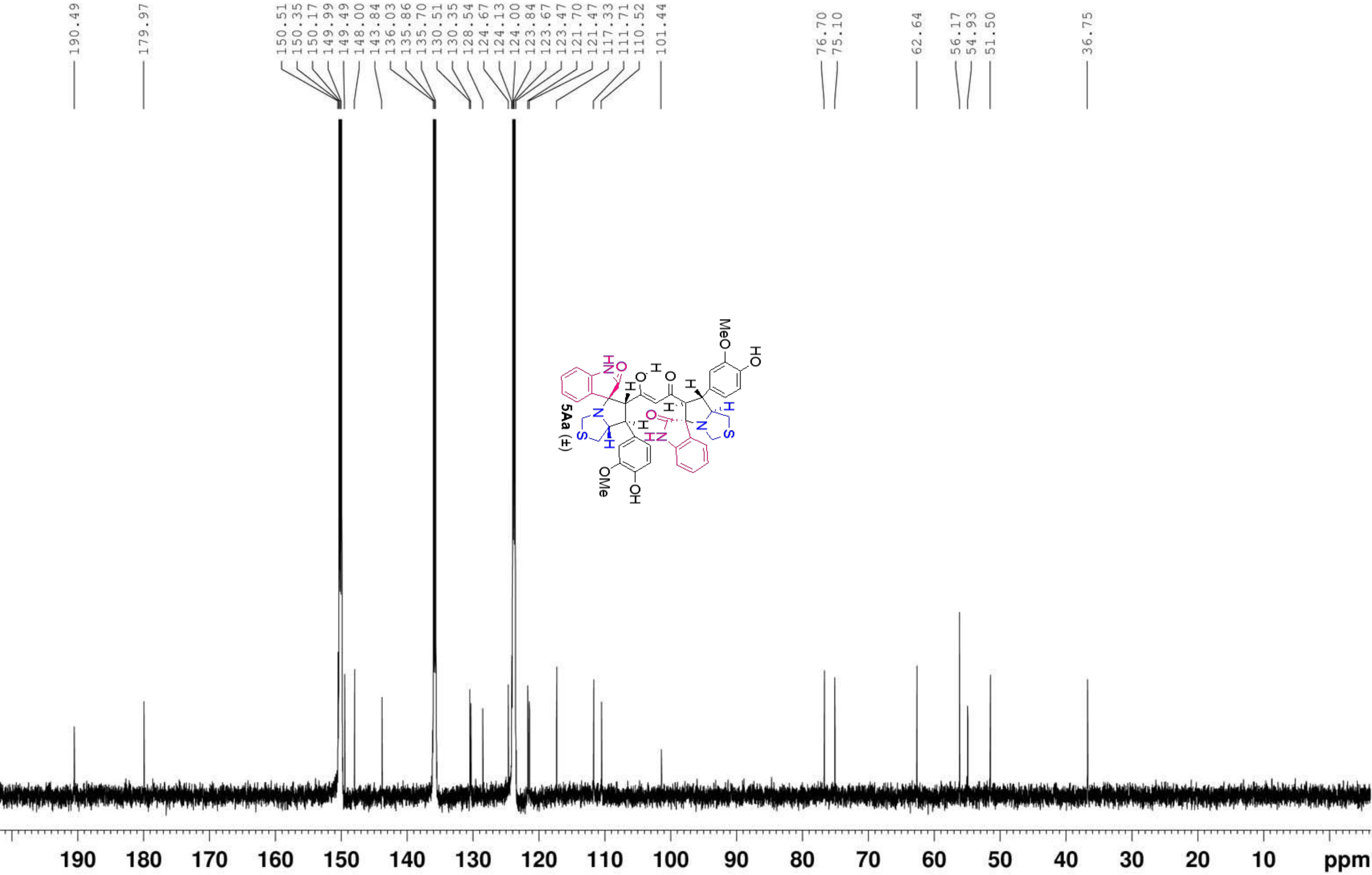

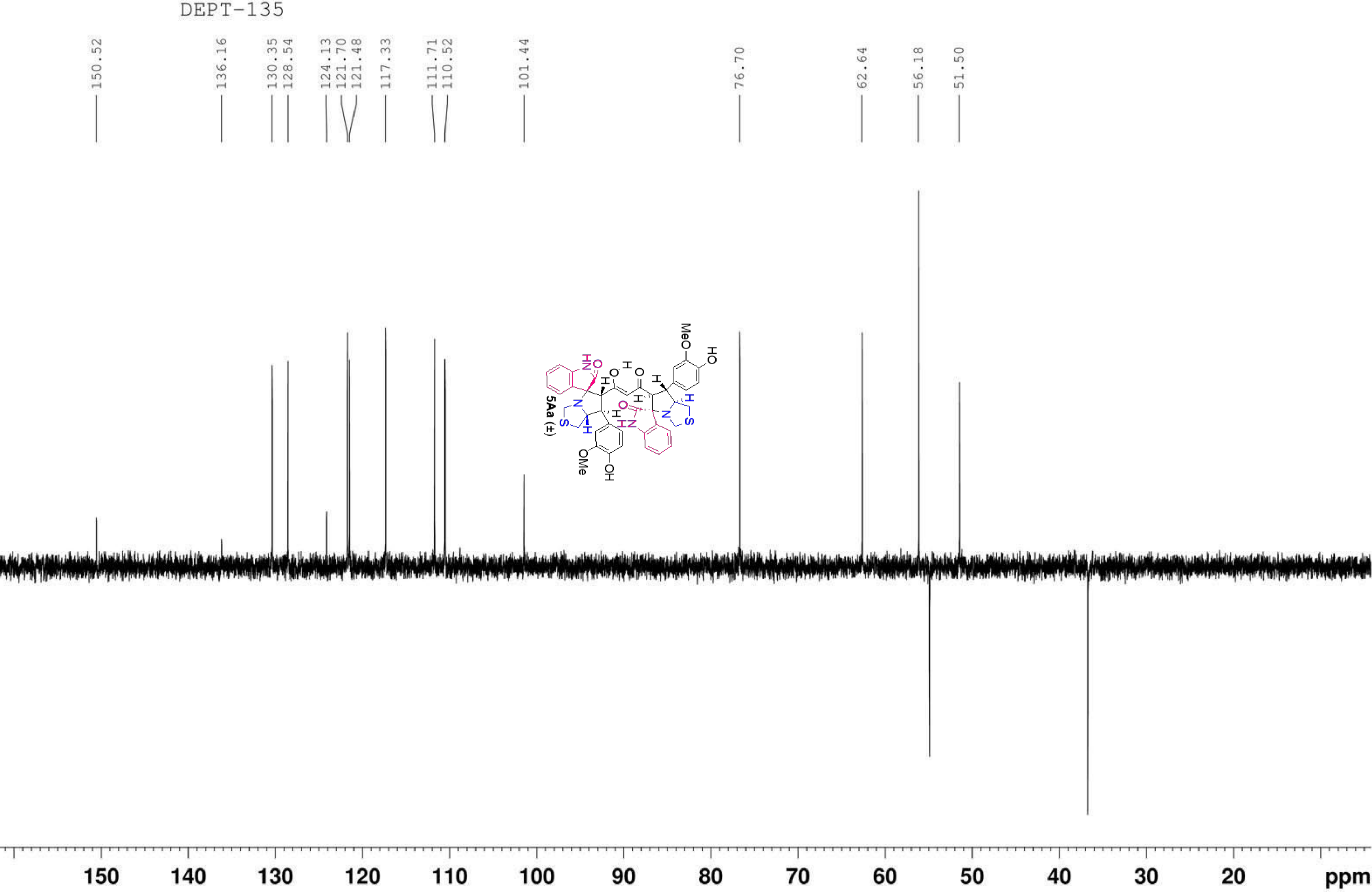
DEPT-135
150.52
136.16
130.35
128.54
124.13
121.70
121.48
117.33
111.71
110.52
101.44
76.70
62.64
56.18
51.50
5Aa (±)
150
140
130
120
110
100
90
80
70
60
50
40
30
20
ppm

1H NMR in Py-d5

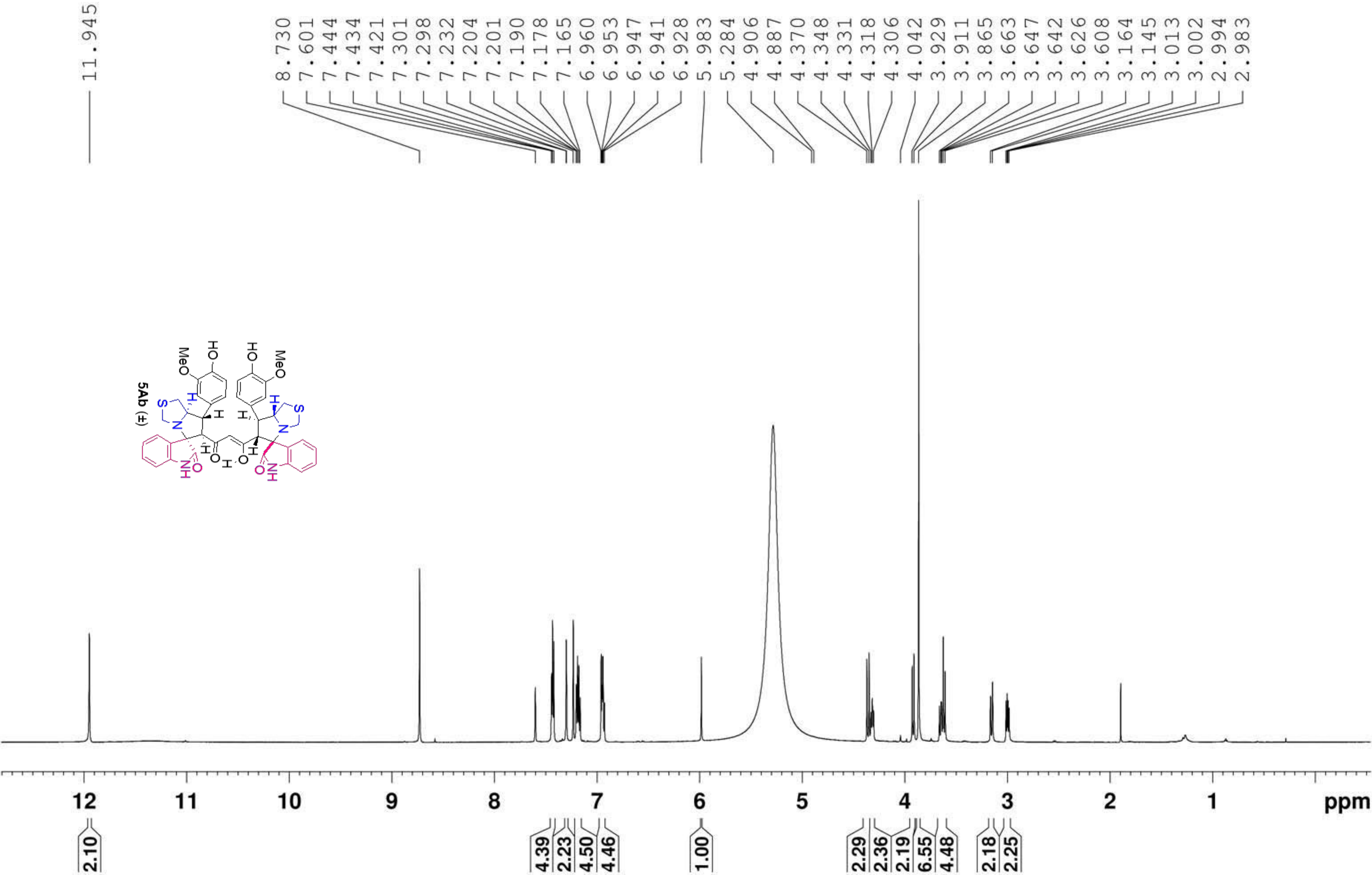

13C-NMR in Py-d5

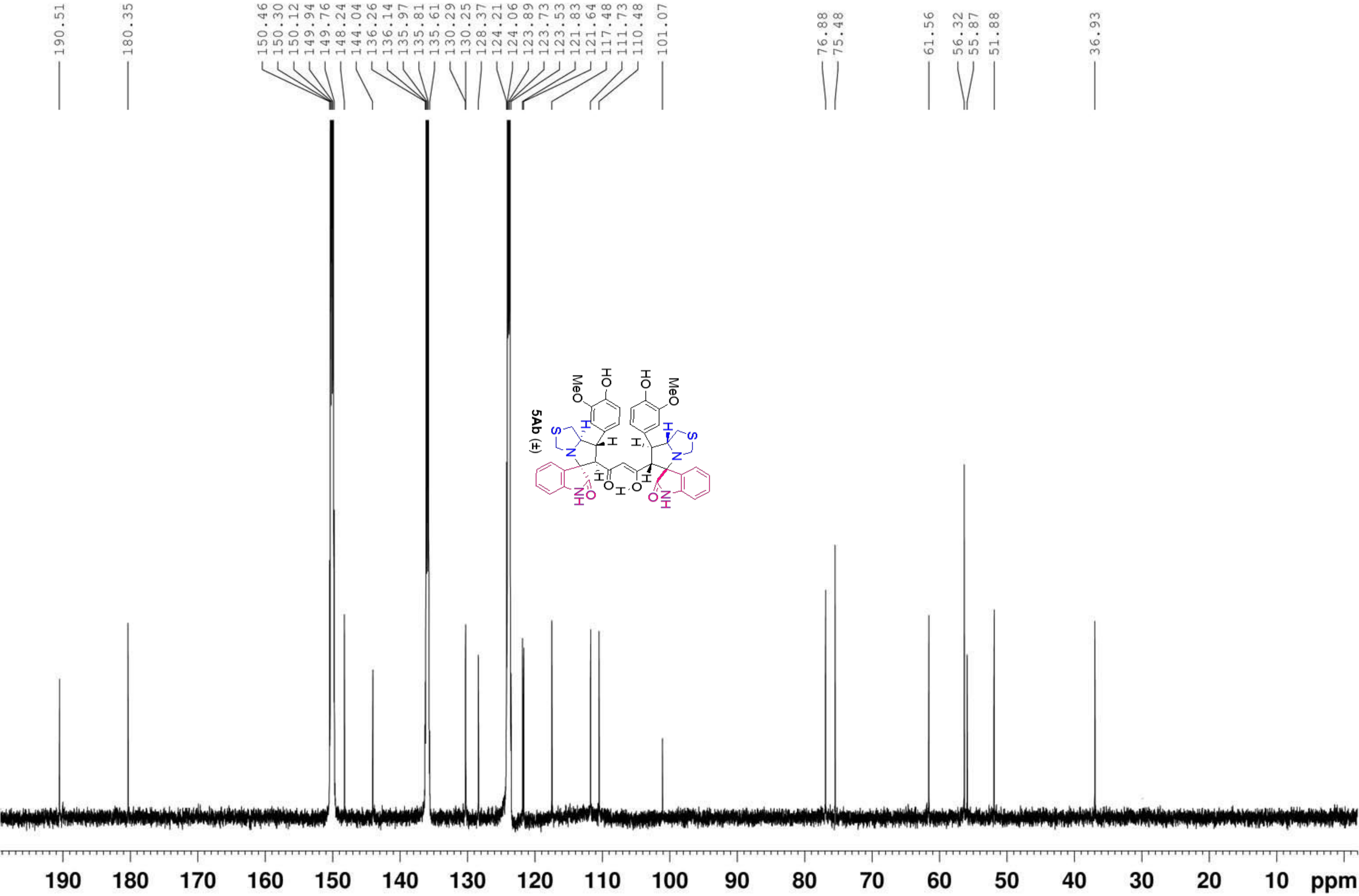

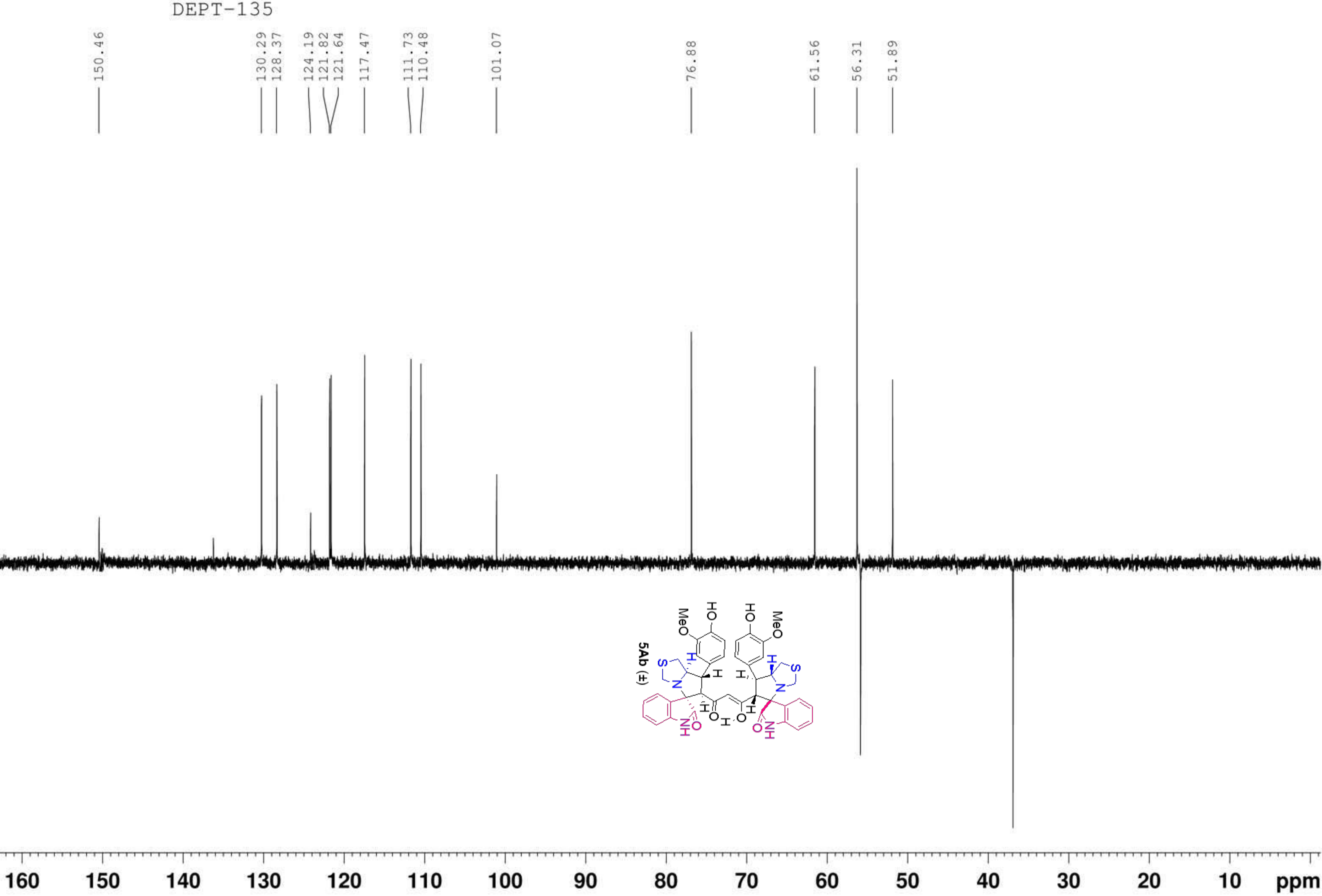
DEPT-135
150.46
130.29
128.37
124.19
121.82
121.64
117.47
111.73
110.48
101.07
76.88
61.56
56.31
51.89
MeO
HO
S
N
NH
O
H
5Ab (±)
160
150
140
130
120
110
100
90
80
70
60
50
40
30
20
10
ppm

1H NMR in Py-d5

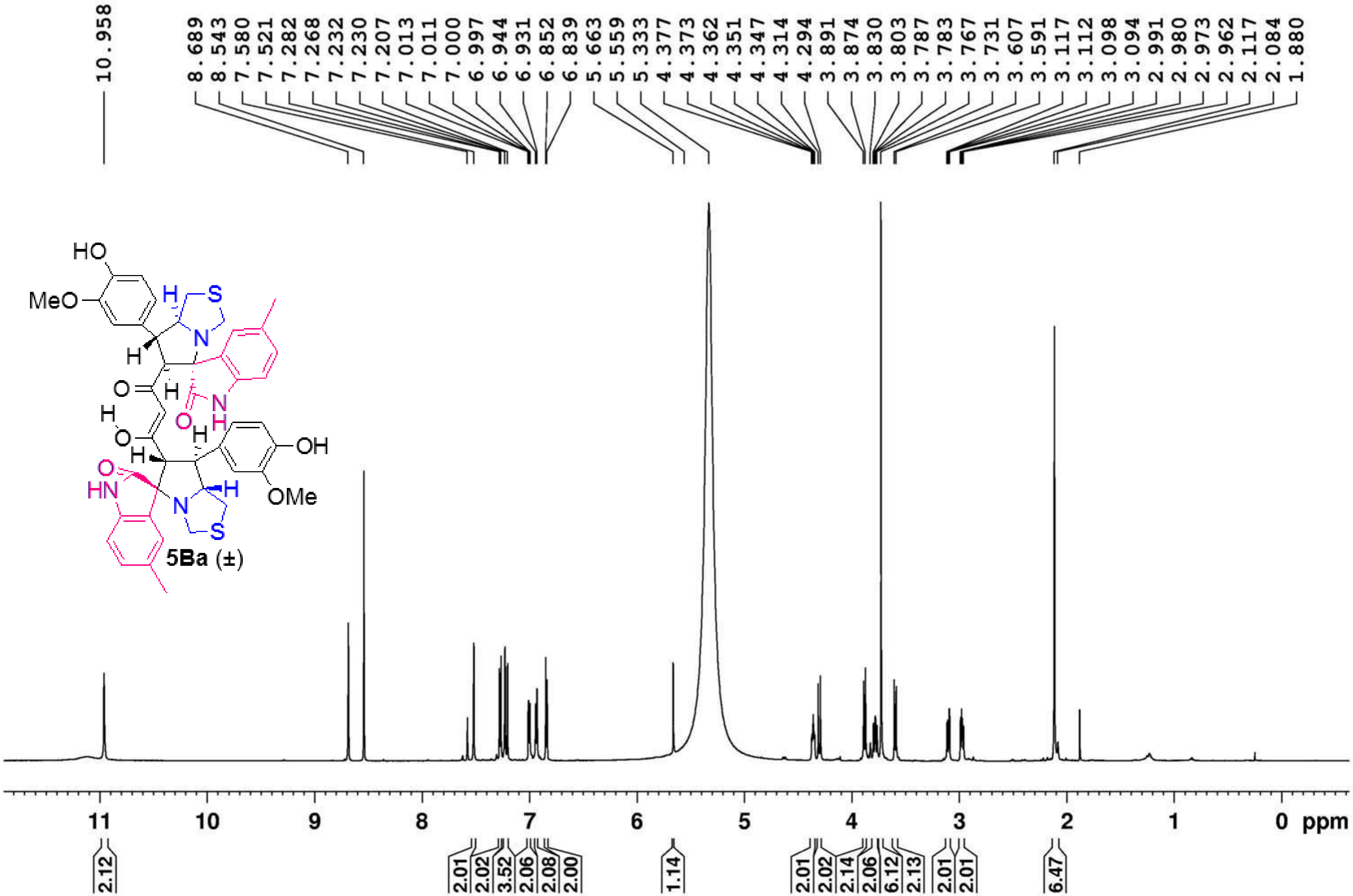

13C-NMR in Py-d5

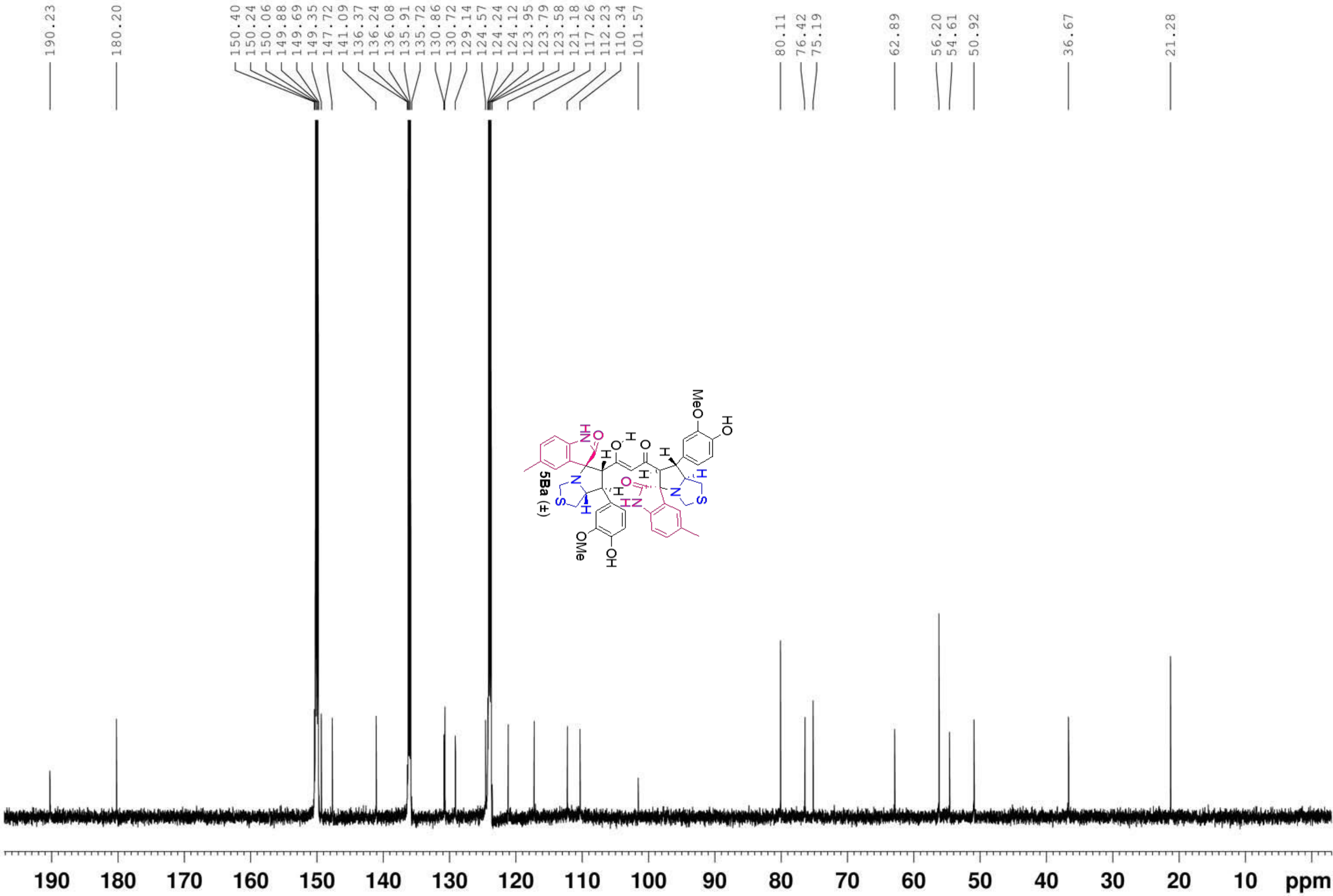

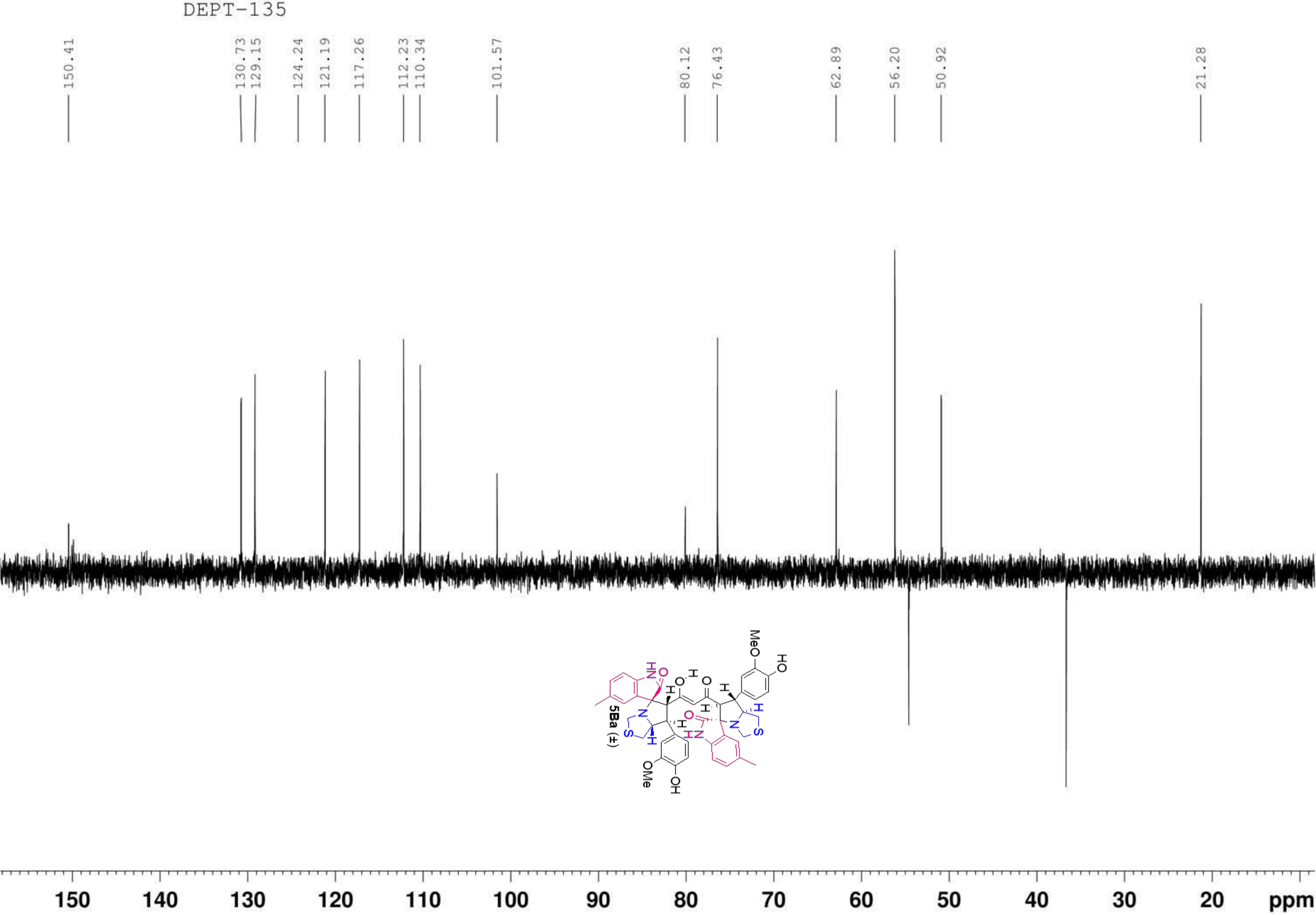
DEPT-135
150.41
130.73
129.15
124.24
121.19
117.26
112.23
110.34
101.57
80.12
76.43
62.89
56.20
50.92
21.28
150
140
130
120
110
100
90
80
70
60
50
40
30
20
ppm
5Ba (±)

1H NMR in Py-d5

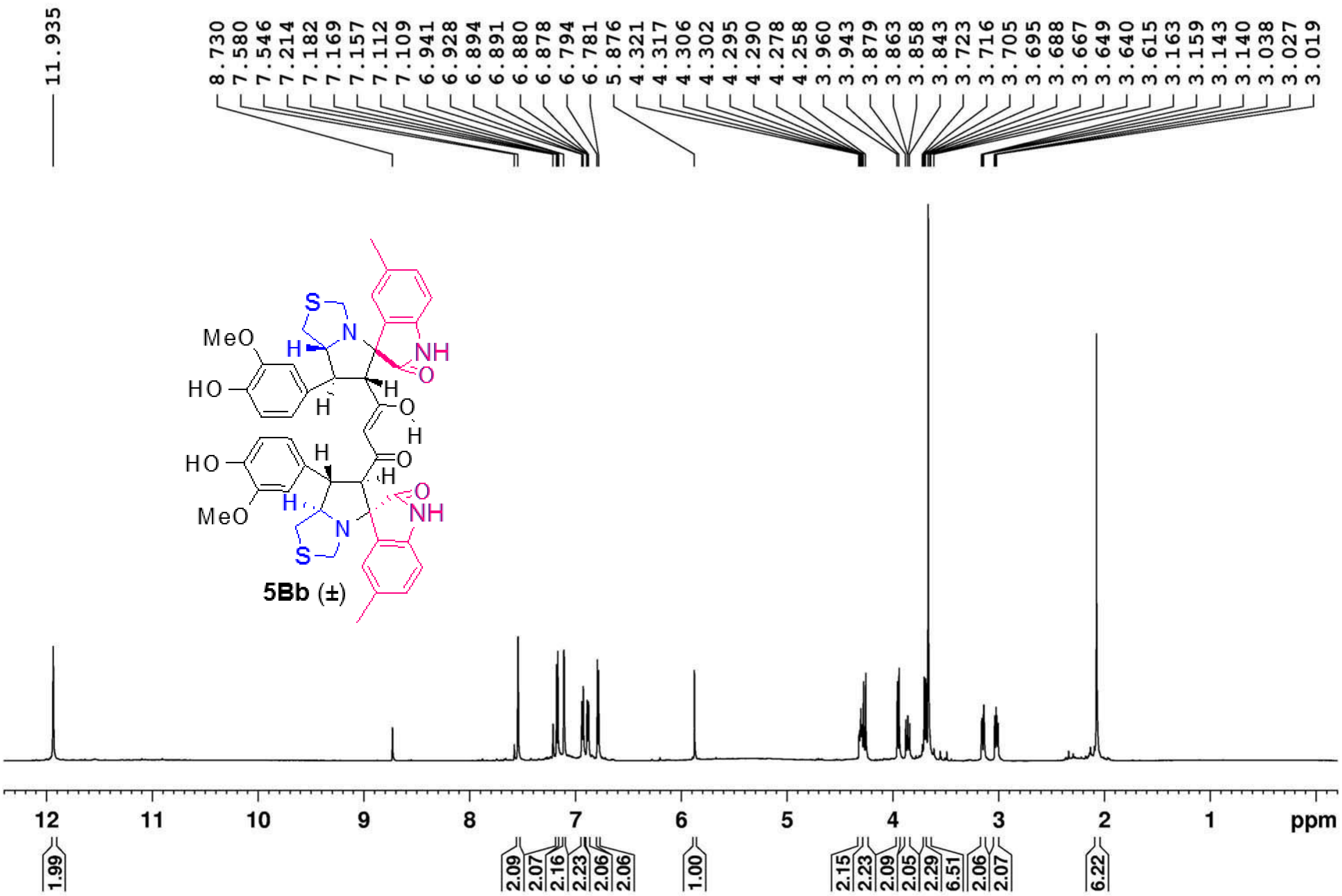

13C-NMR in Py-d5

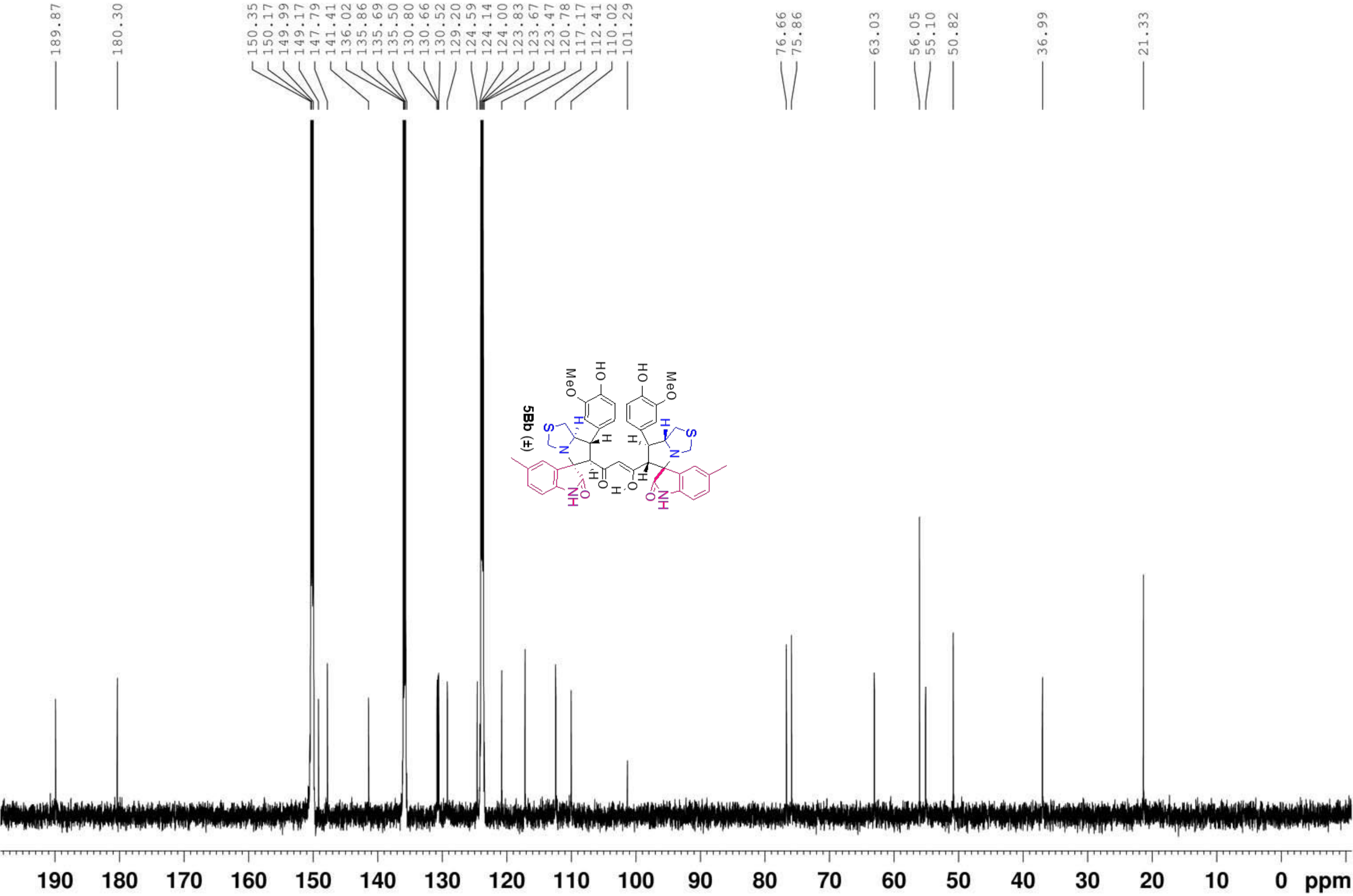

DEPT-135

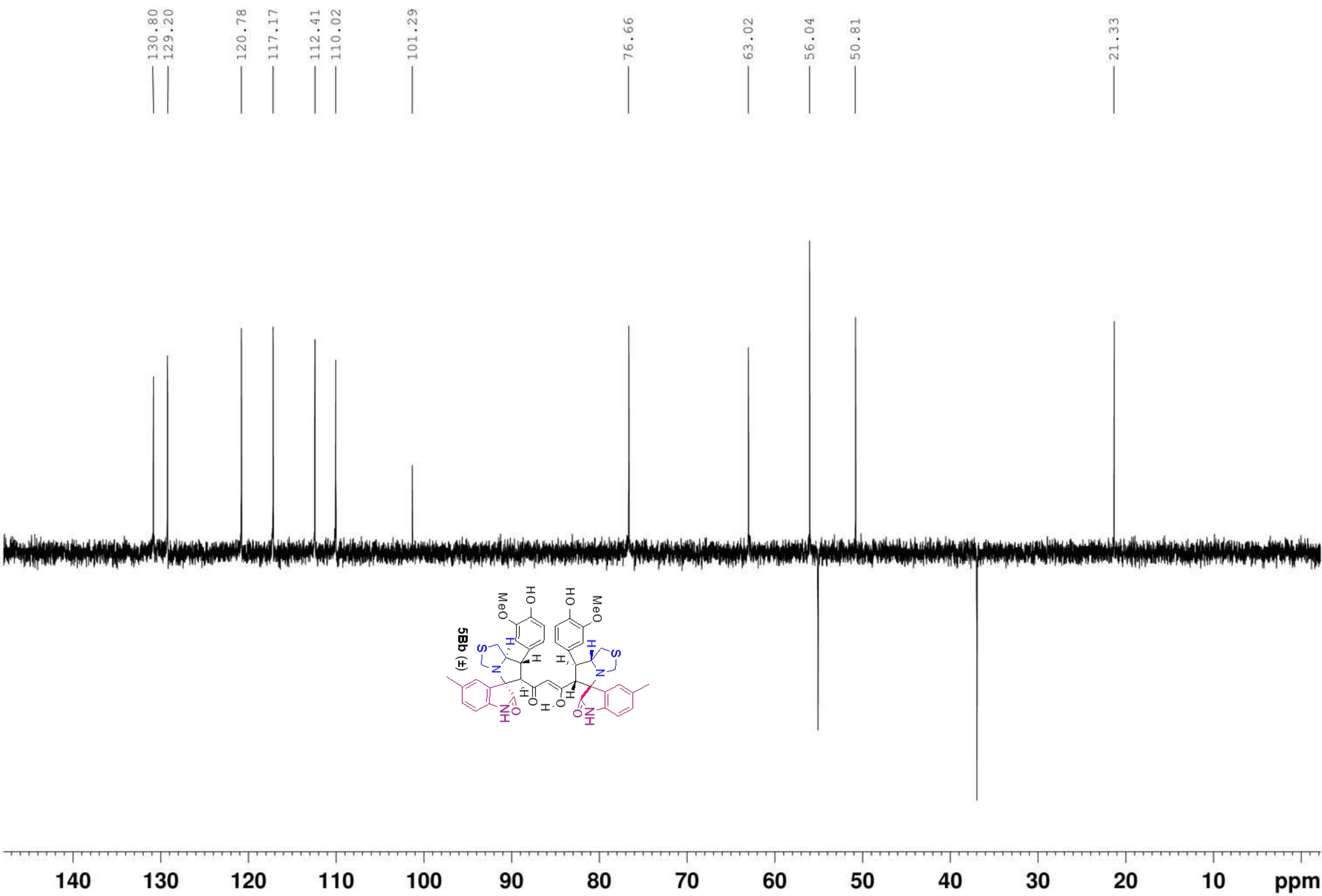
130.80
129.20
120.78
117.17
112.41
110.02
101.29
76.66
63.02
56.04
50.81
21.33
MeO
HO
HO
MeO
S
S
N
N
NH
NH
O
O
O
O
H
140
130
120
110
100
90
80
70
60
50
40
30
20
10
ppm

5Bb (±)

1H NMR in Py-d5

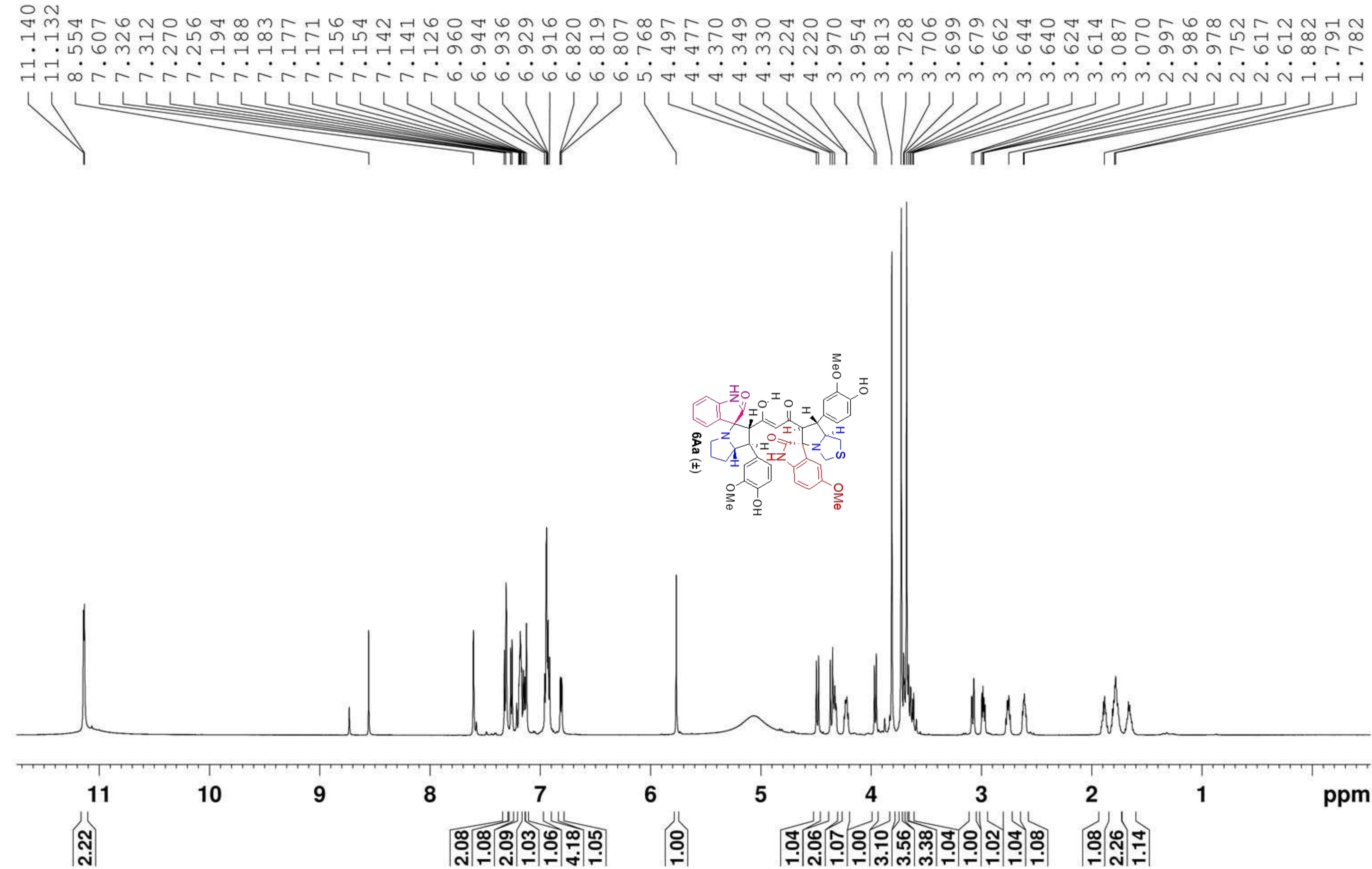

13C-NMR in Py-d5

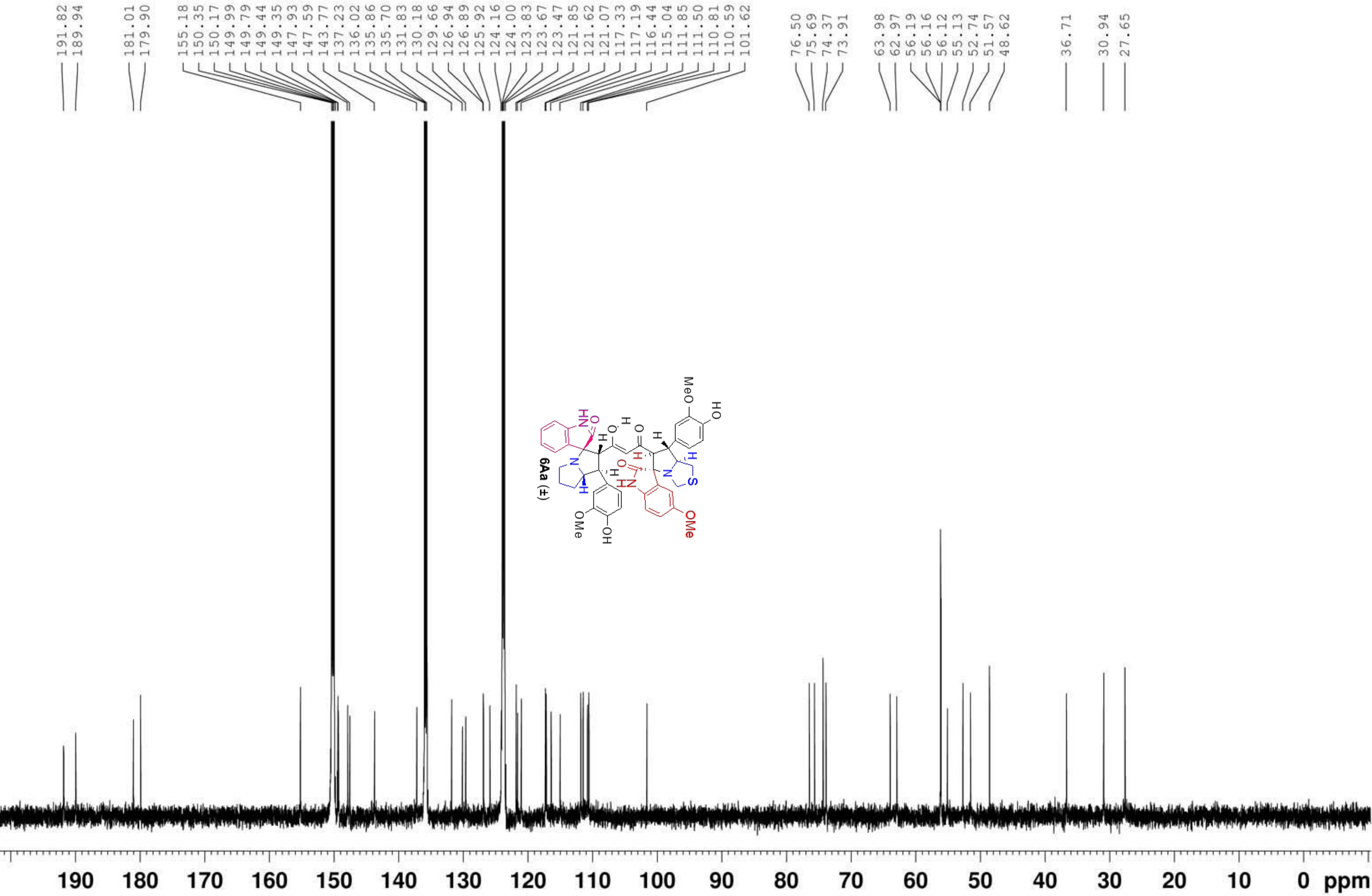

DEPT-135

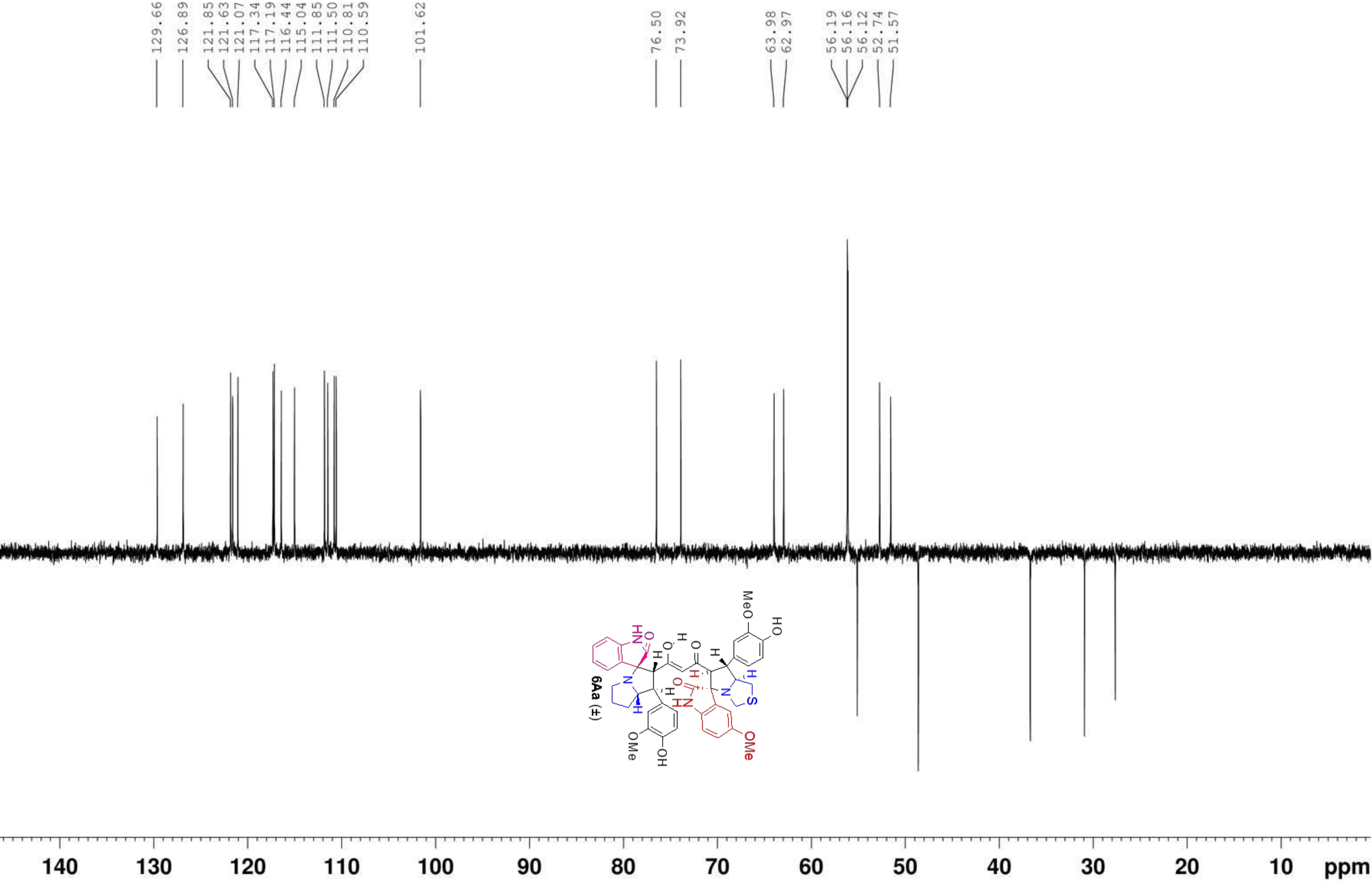
129.66
126.89
121.85
121.63
121.07
117.34
117.19
116.44
115.04
111.85
111.50
110.81
110.59
101.62
76.50
73.92
63.98
62.97
56.19
56.16
56.12
52.74
51.57
6Aa (±)
140 130 120 110 100 90 80 70 60 50 40 30 20 10 ppm

1H NMR in Py-d5

11.913
11.782
8.730
8.558
7.495
7.491
7.237
7.223
7.210
7.183
7.182
7.169
7.164
7.161
7.151
7.148
7.137
7.125
7.015
7.012
7.002
6.999
6.976
6.973
6.969
6.963
6.957
6.943
6.940
6.926
6.918
6.905
6.877
6.873
6.863
6.859
5.954
4.405
4.385
4.374
4.353
4.288
3.980
3.962
3.758
3.720
3.699
3.656
3.651
3.642
3.614
3.125
3.123
3.020
3.009
3.001
1.864

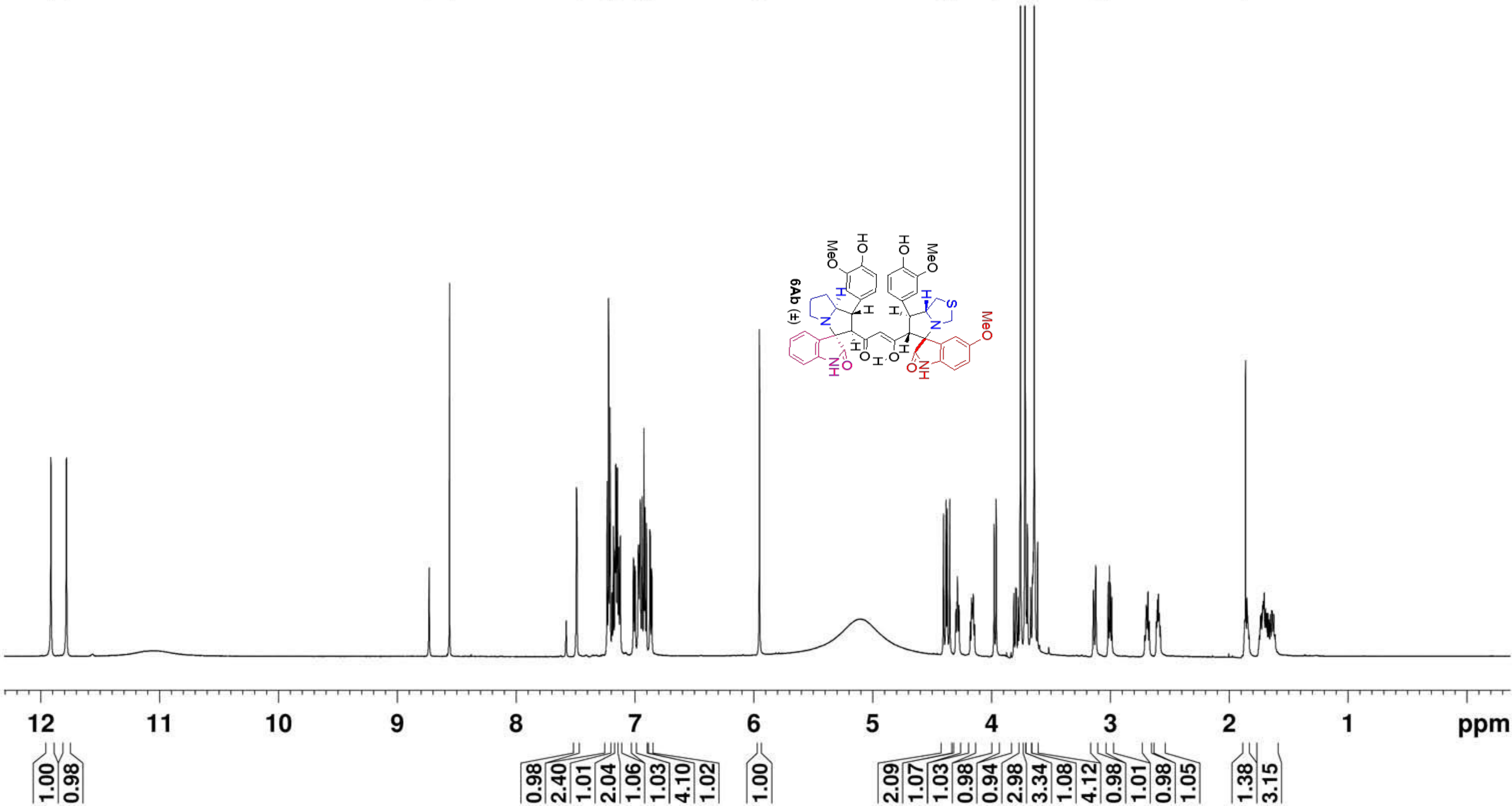

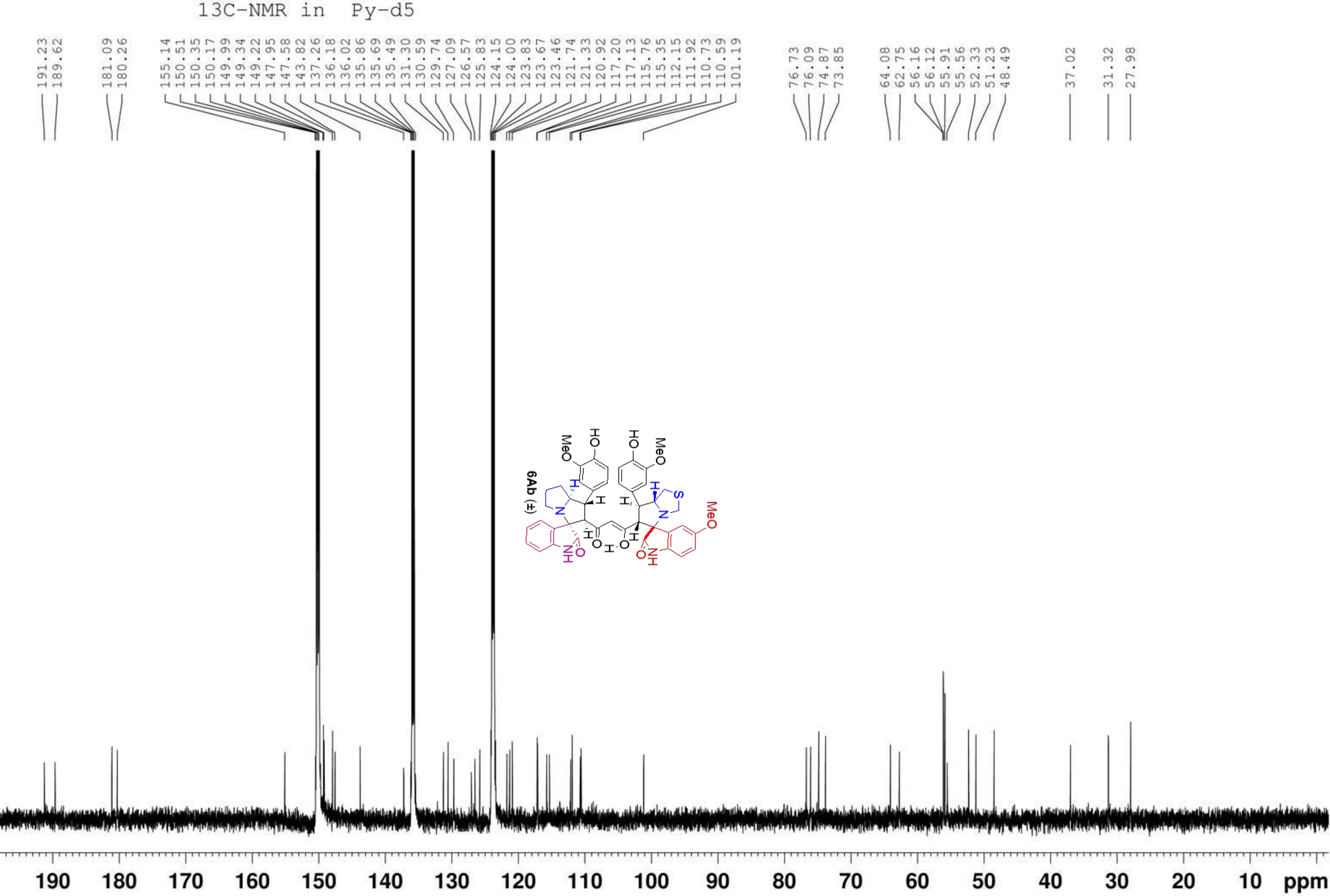
13C-NMR in Py-d5
191.23
189.62
181.09
180.26
155.14
150.51
150.35
150.17
149.99
149.34
149.22
147.95
147.58
143.82
137.26
136.18
136.02
135.86
135.69
135.49
131.30
130.59
129.74
127.09
126.57
125.83
124.15
124.00
123.83
123.67
123.46
121.74
121.33
120.92
117.20
117.13
115.76
115.35
112.15
111.92
110.73
110.59
101.19
76.73
76.09
74.87
73.85
64.08
62.75
56.16
56.12
55.91
55.56
52.33
51.23
48.49
37.02
31.32
27.98
6Ab (±)
ppm

DEPT-135

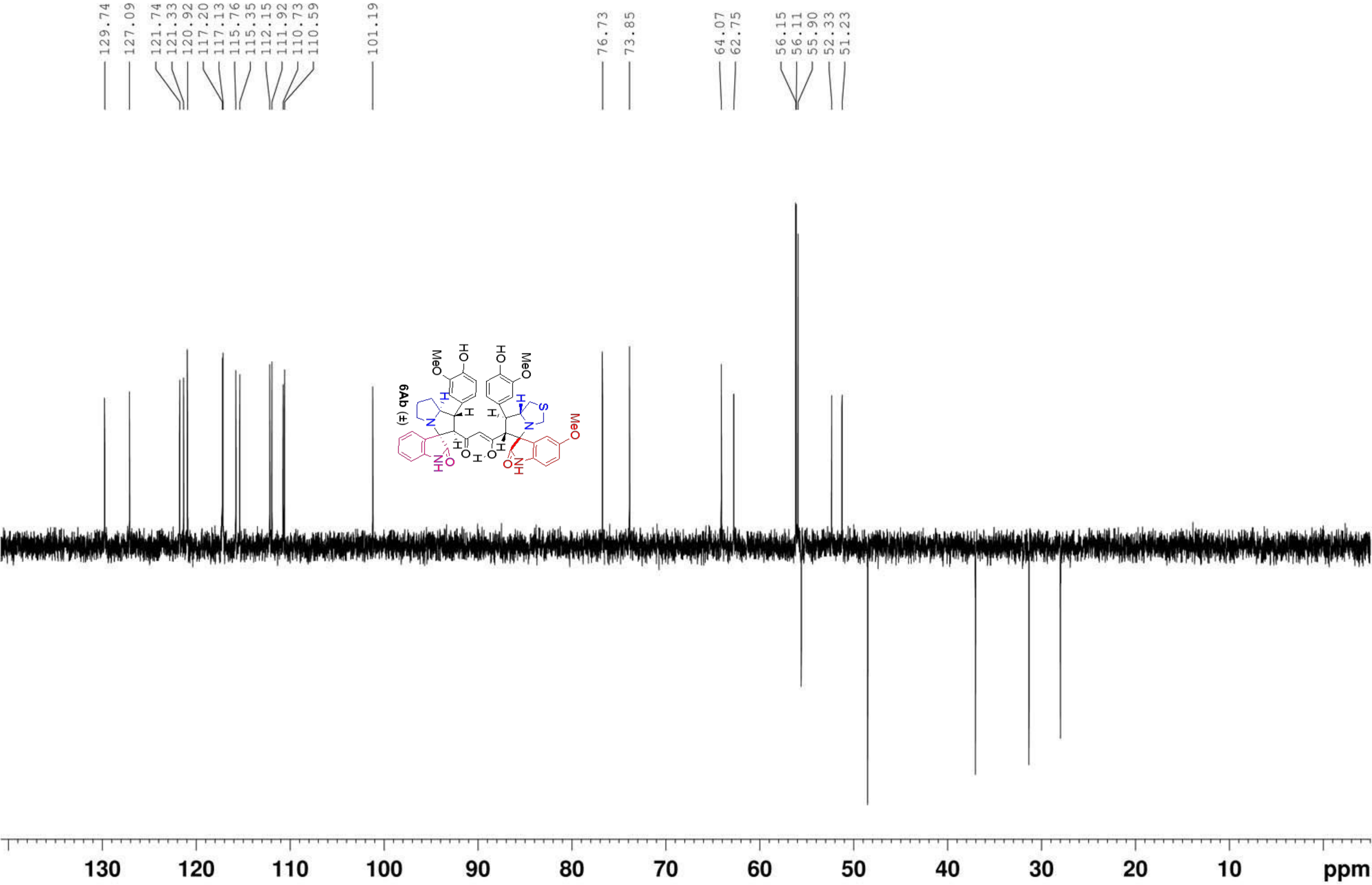
129.74
127.09
121.74
121.33
120.92
117.20
117.13
115.76
115.35
112.15
111.92
110.73
110.59
101.19
76.73
73.85
64.07
62.75
56.15
56.11
55.90
52.33
51.23
6Ab (±)
130
120
110
100
90
80
70
60
50
40
30
20
10
ppm

1H NMR in Py-d5

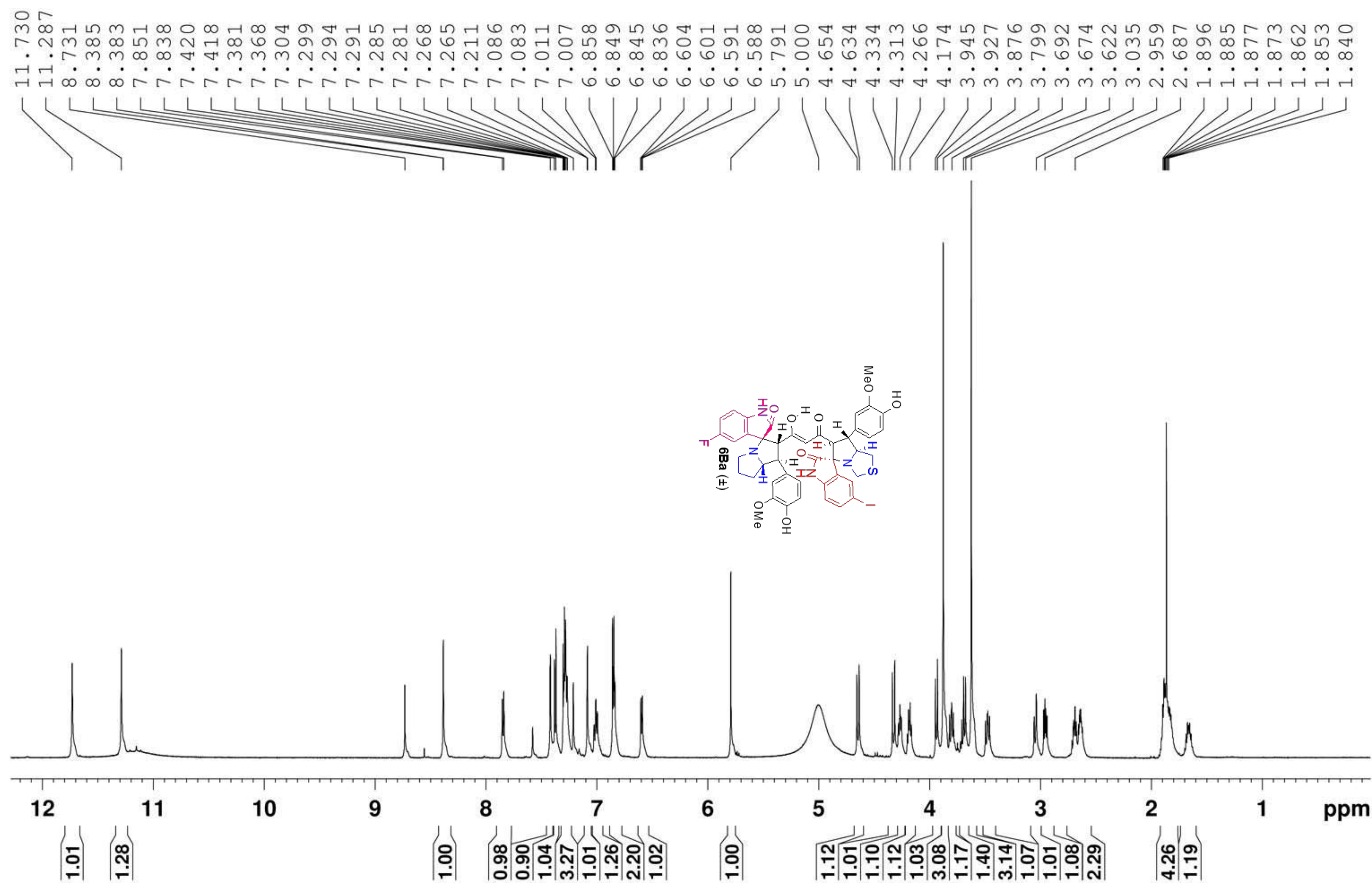

13C-NMR in Py-d5

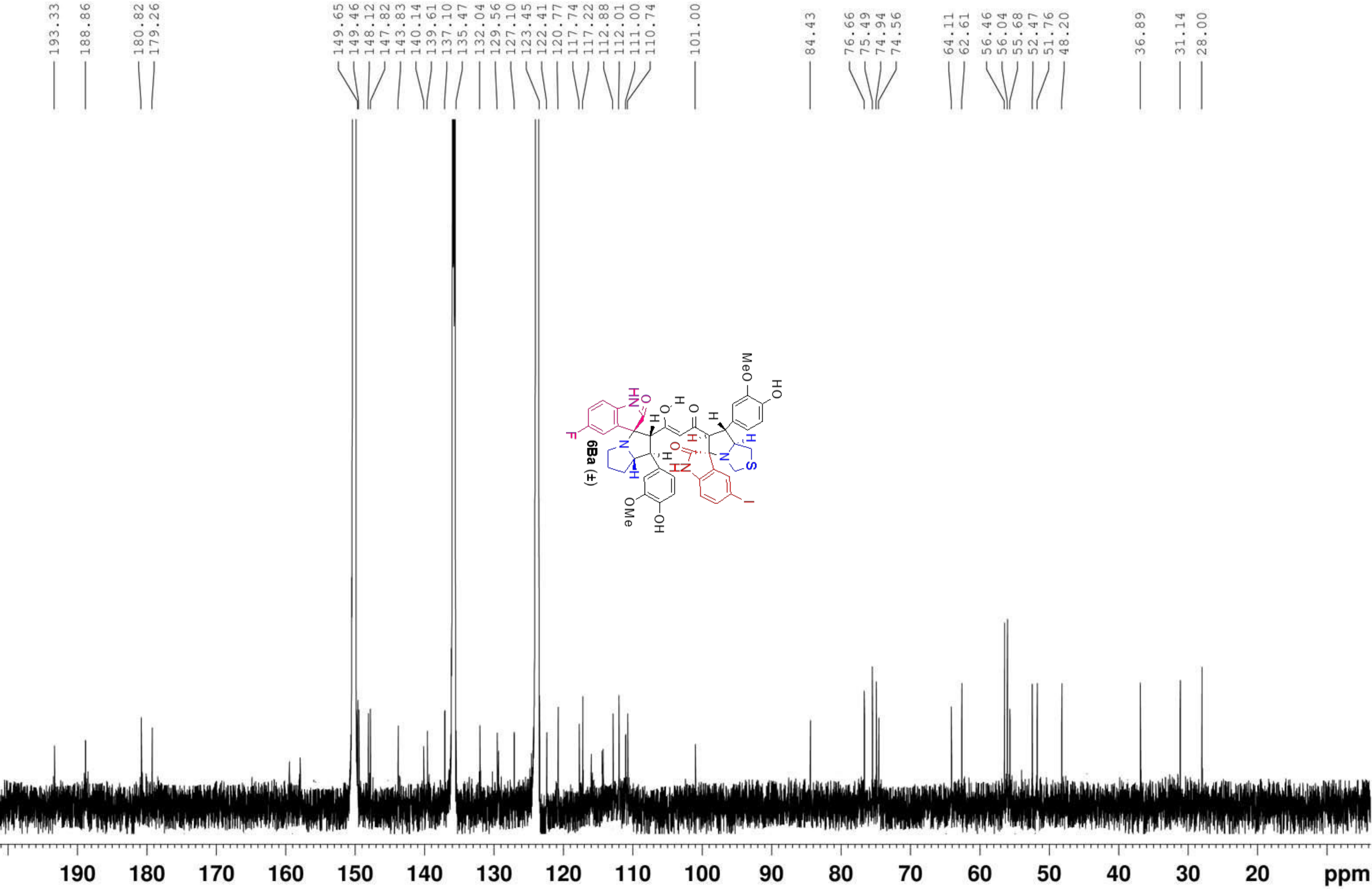

DEPT-135

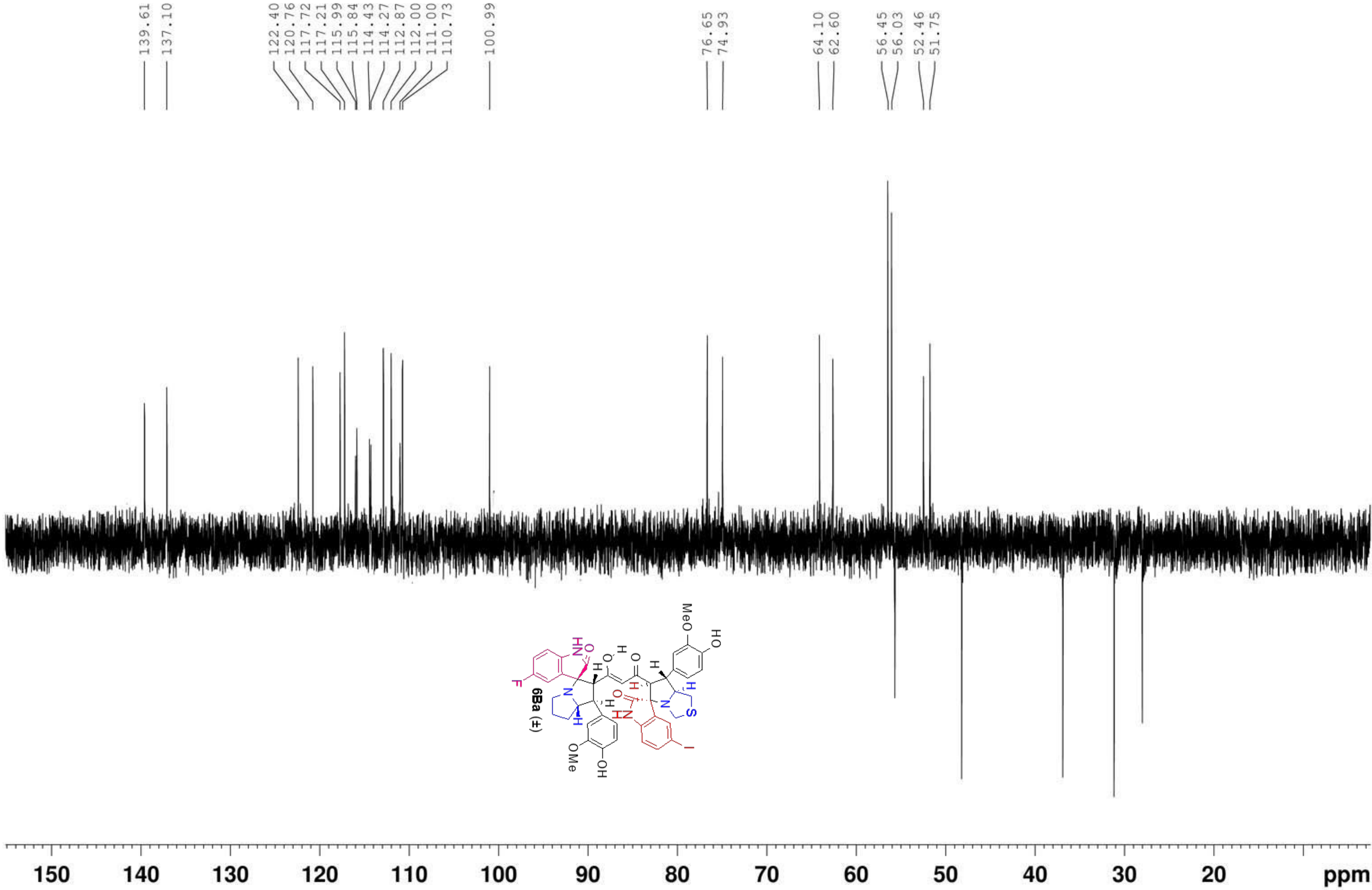
139.61
137.10
122.40
120.76
117.72
117.21
115.99
115.84
114.43
114.27
112.87
112.00
111.00
110.73
100.99
76.65
74.93
64.10
62.60
56.45
56.03
52.46
51.75
MeO
HO
OH
OMe
HN
NH
S
N
I
F
6Ba (±)
150
140
130
120
110
100
90
80
70
60
50
40
30
20
ppm

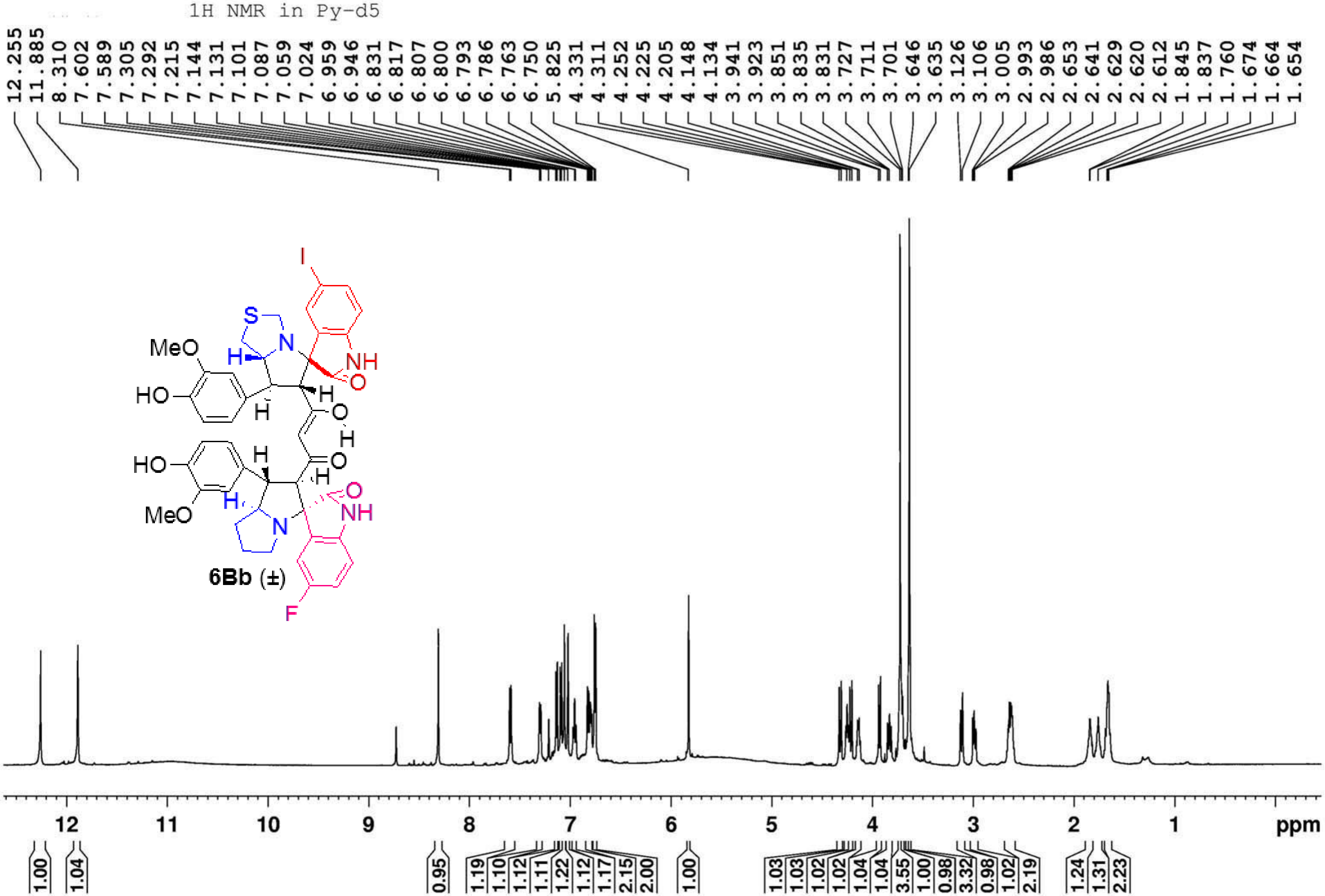
1H NMR in Py-d5
12.255
11.885
8.310
7.602
7.589
7.305
7.292
7.215
7.144
7.131
7.101
7.087
7.059
7.024
6.959
6.946
6.831
6.817
6.807
6.800
6.793
6.786
6.763
6.750
5.825
4.331
4.311
4.252
4.225
4.205
4.148
4.134
3.941
3.923
3.851
3.835
3.831
3.727
3.711
3.701
3.646
3.635
3.126
3.106
3.005
2.993
2.986
2.653
2.641
2.629
2.620
2.612
1.845
1.837
1.760
1.674
1.664
1.654
I
S
N
MeO
H
NH
O
HO
H
H
O
H
HO
H
O
H
O
H
NH
MeO
N
6Bb (±)
F
12
11
10
9
8
7
6
5
4
3
2
1
ppm
1.00
1.04
0.95
1.19
1.10
1.12
1.11
1.22
1.12
1.17
2.15
2.00
1.00
1.03
1.03
1.02
1.02
1.04
1.04
3.55
1.00
0.98
3.32
0.98
1.02
2.19
1.24
1.31
2.23

13C-NMR in Py-d5

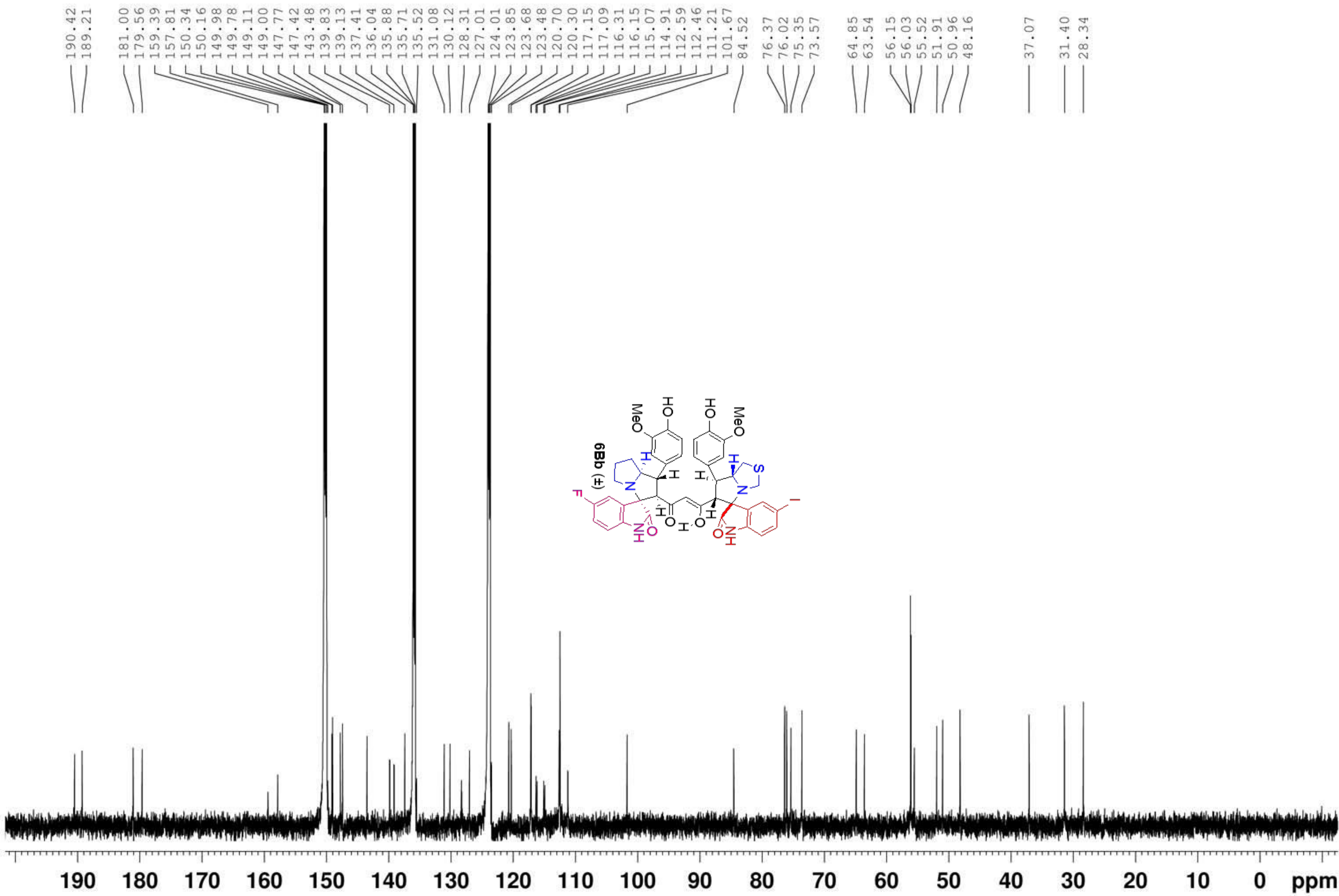

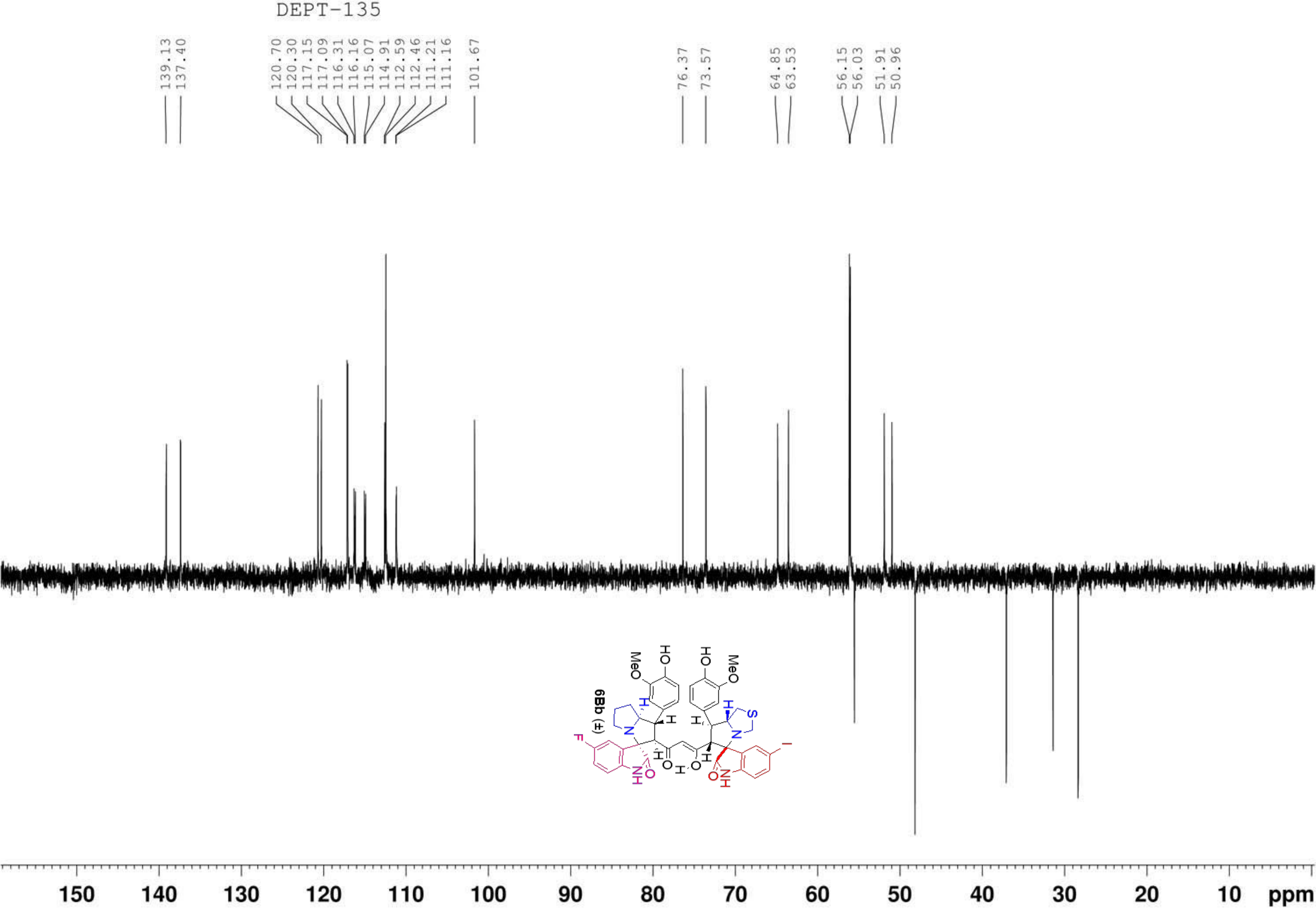

DEPT-135
139.13
137.40
120.70
120.30
117.15
117.09
116.31
116.16
115.07
114.91
112.59
112.46
111.21
111.16
101.67
76.37
73.57
64.85
63.53
56.15
56.03
51.91
50.96
6Bb (±)
150
140
130
120
110
100
90
80
70
60
50
40
30
20
10
ppm

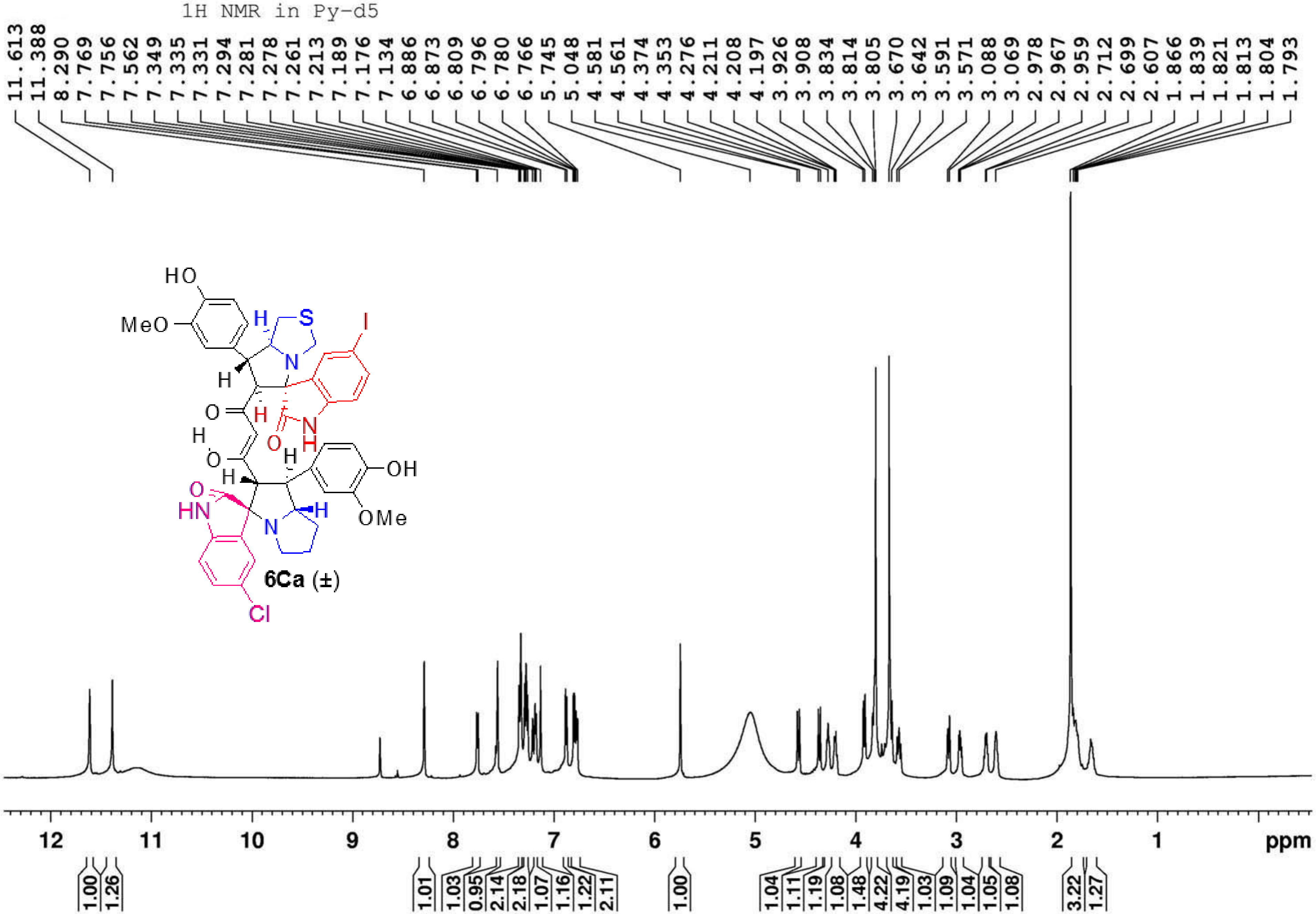
1H NMR in Py-d5
11.613 11.388 8.290 7.769 7.756 7.562 7.349 7.335 7.331 7.294 7.281 7.278 7.261 7.213 7.189 7.176 7.134 6.886 6.873 6.809 6.796 6.780 6.766 5.745 5.048 4.581 4.561 4.374 4.353 4.276 4.211 4.208 4.197 3.926 3.908 3.834 3.814 3.805 3.670 3.642 3.591 3.571 3.088 3.069 2.978 2.967 2.959 2.712 2.699 2.607 1.866 1.839 1.821 1.813 1.804 1.793
HO
MeO
H
S
I
N
H
O
H
N
H
O
H
O
H
OH
O
HN
N
H
OMe
Cl
6Ca (±)
12
11
10
9
8
7
6
5
4
3
2
1
ppm
1.00 1.26 1.01 1.03 0.95 2.14 2.18 1.07 1.16 1.22 2.11 1.00 1.04 1.11 1.19 1.08 1.48 4.22 4.19 1.03 1.09 1.04 1.05 1.08 3.22 1.27

13C-NMR in Py-d5

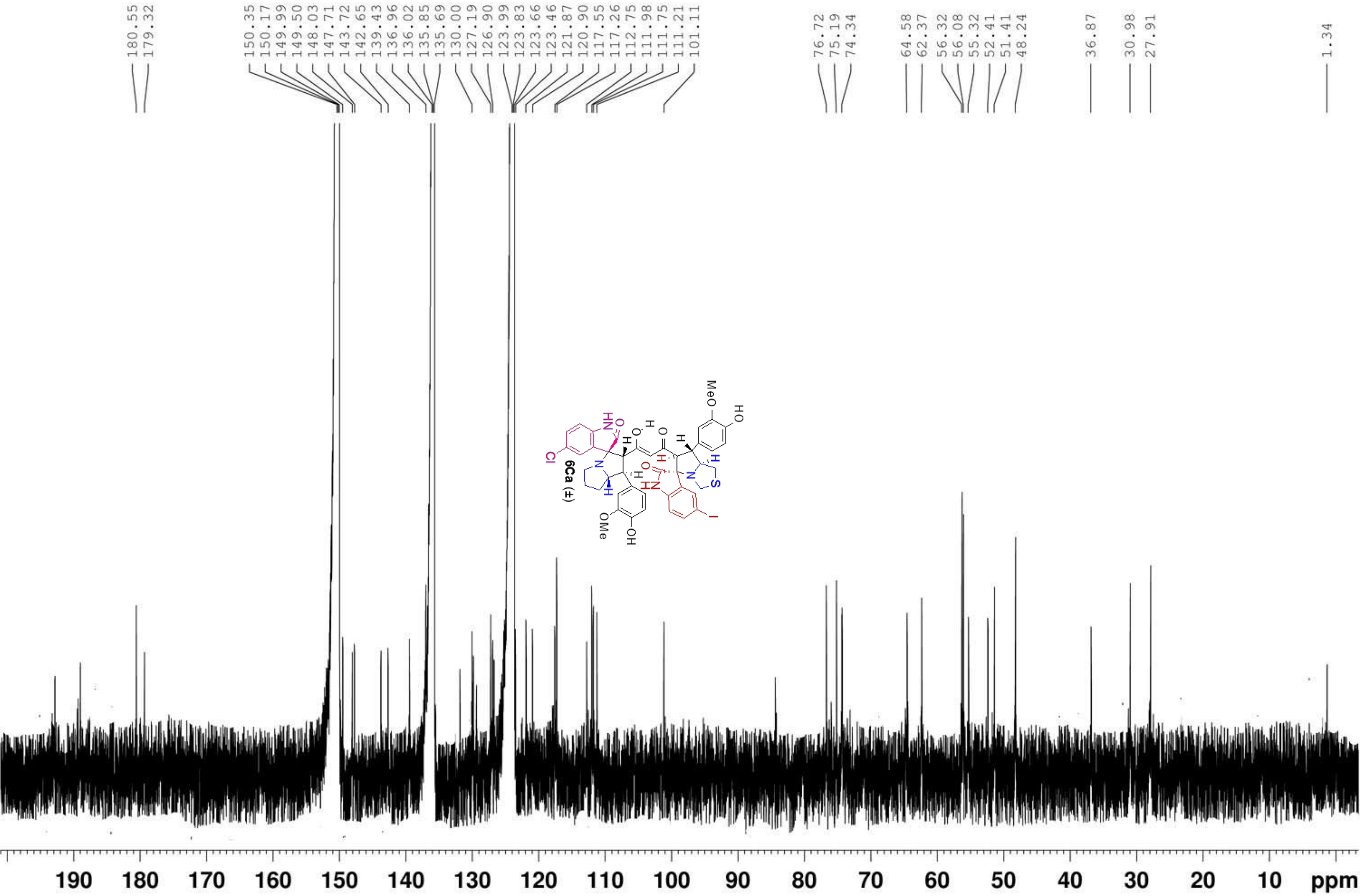

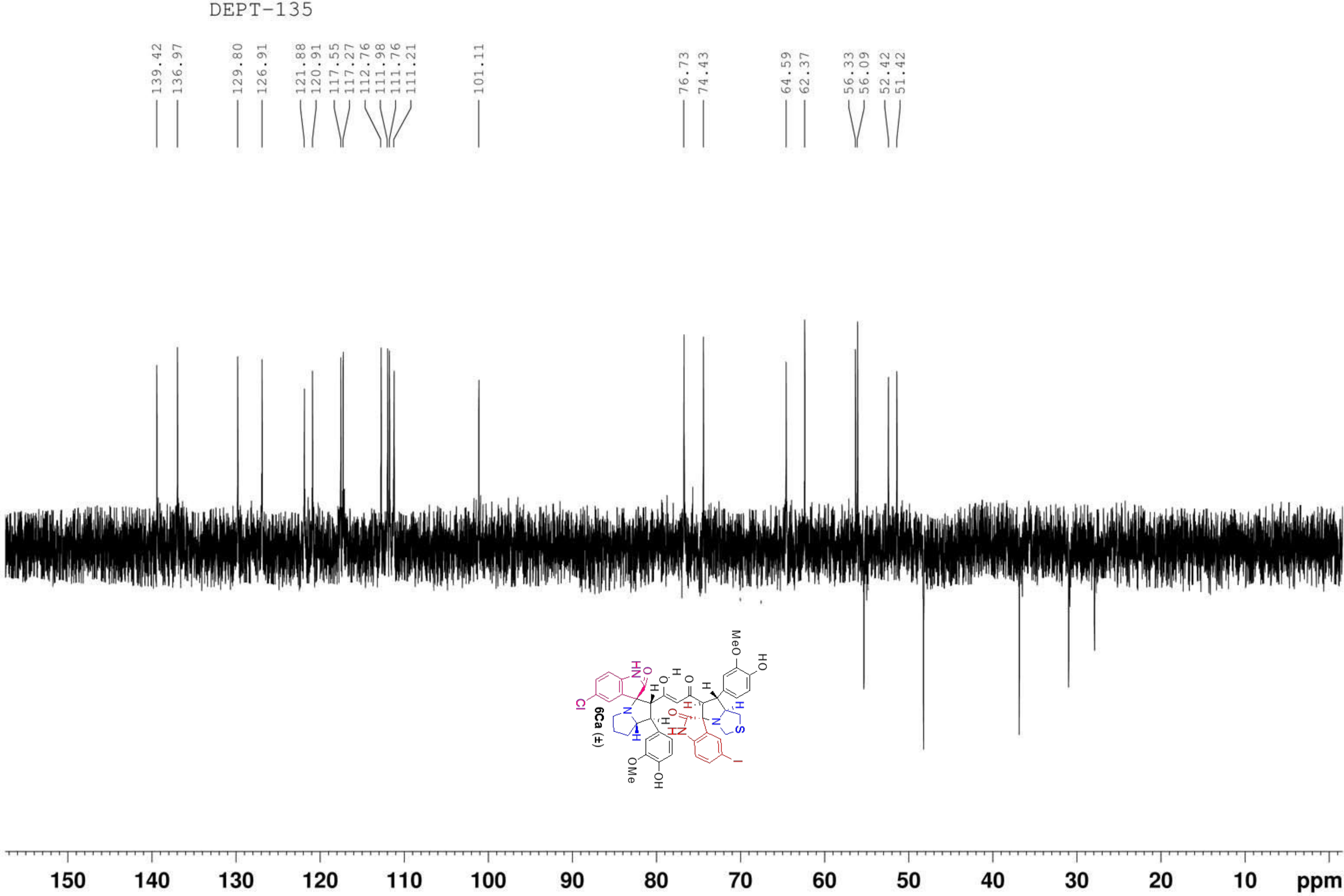
DEPT-135
139.42
136.97
129.80
126.91
121.88
120.91
117.55
117.27
112.76
111.98
111.76
111.21
101.11
76.73
74.43
64.59
62.37
56.33
56.09
52.42
51.42
6Ca (±)
ppm
150
140
130
120
110
100
90
80
70
60
50
40
30
20
10

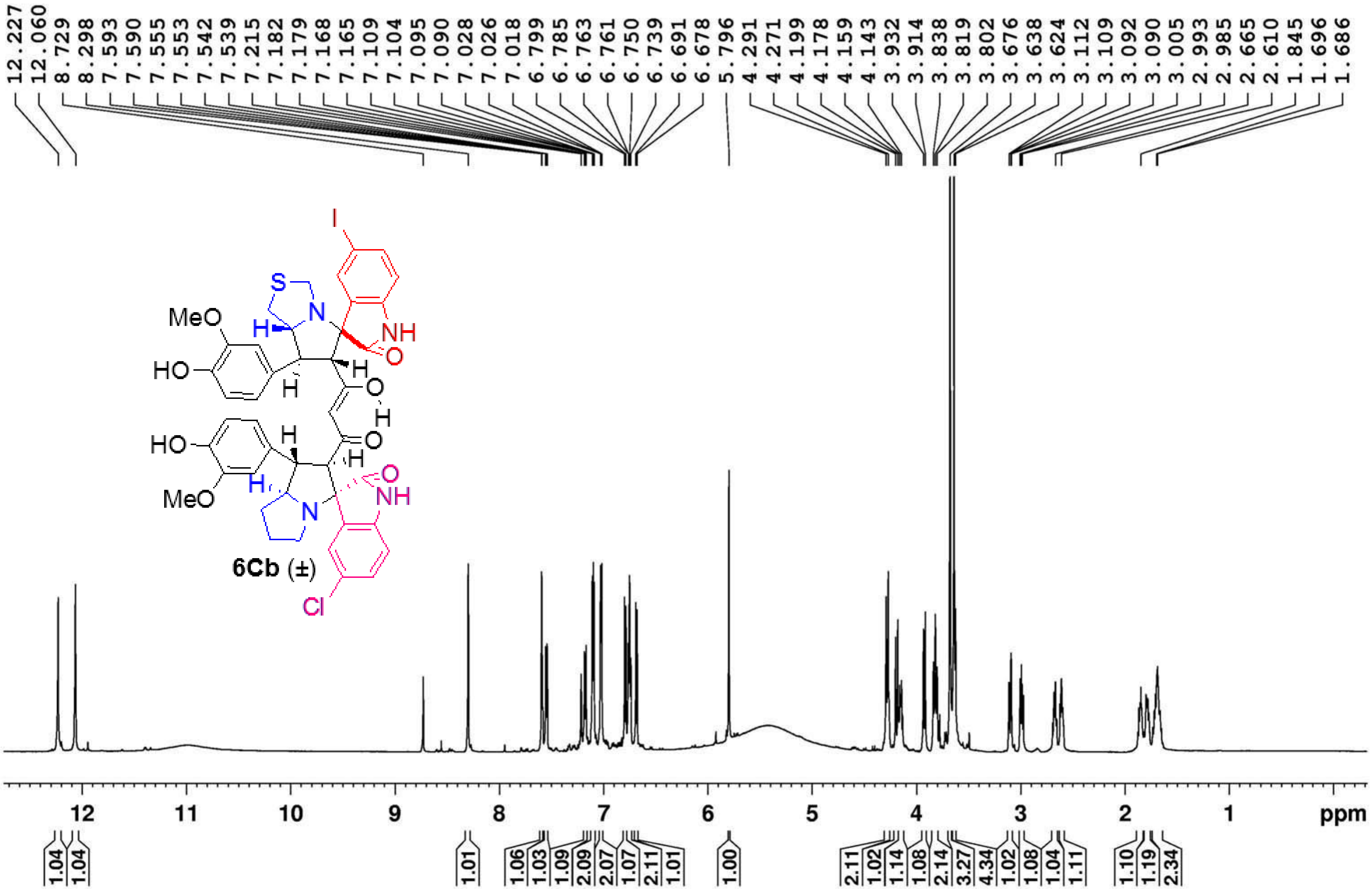

12.227
12.060
8.729
8.298
7.593
7.590
7.555
7.553
7.542
7.539
7.215
7.182
7.179
7.168
7.165
7.109
7.104
7.095
7.090
7.028
7.026
7.018
6.799
6.785
6.763
6.761
6.750
6.739
6.691
6.678
5.796
4.291
4.271
4.199
4.178
4.159
4.143
3.932
3.914
3.838
3.819
3.802
3.676
3.638
3.624
3.112
3.109
3.092
3.090
3.005
2.993
2.985
2.665
2.610
1.845
1.696
1.686
I
S
N
NH
O
MeO
HO
H
O
H
HO
H
O
H
H
O
NH
MeO
N
Cl
6Cb (±)
12
11
10
9
8
7
6
5
4
3
2
1
ppm
1.04
1.04
1.01
1.06
1.03
1.09
2.09
2.07
1.07
2.11
1.01
1.00
2.11
1.02
1.14
1.08
2.14
3.27
4.34
1.02
1.08
1.04
1.11
1.10
1.19
2.34

13C-NMR in Py-d5

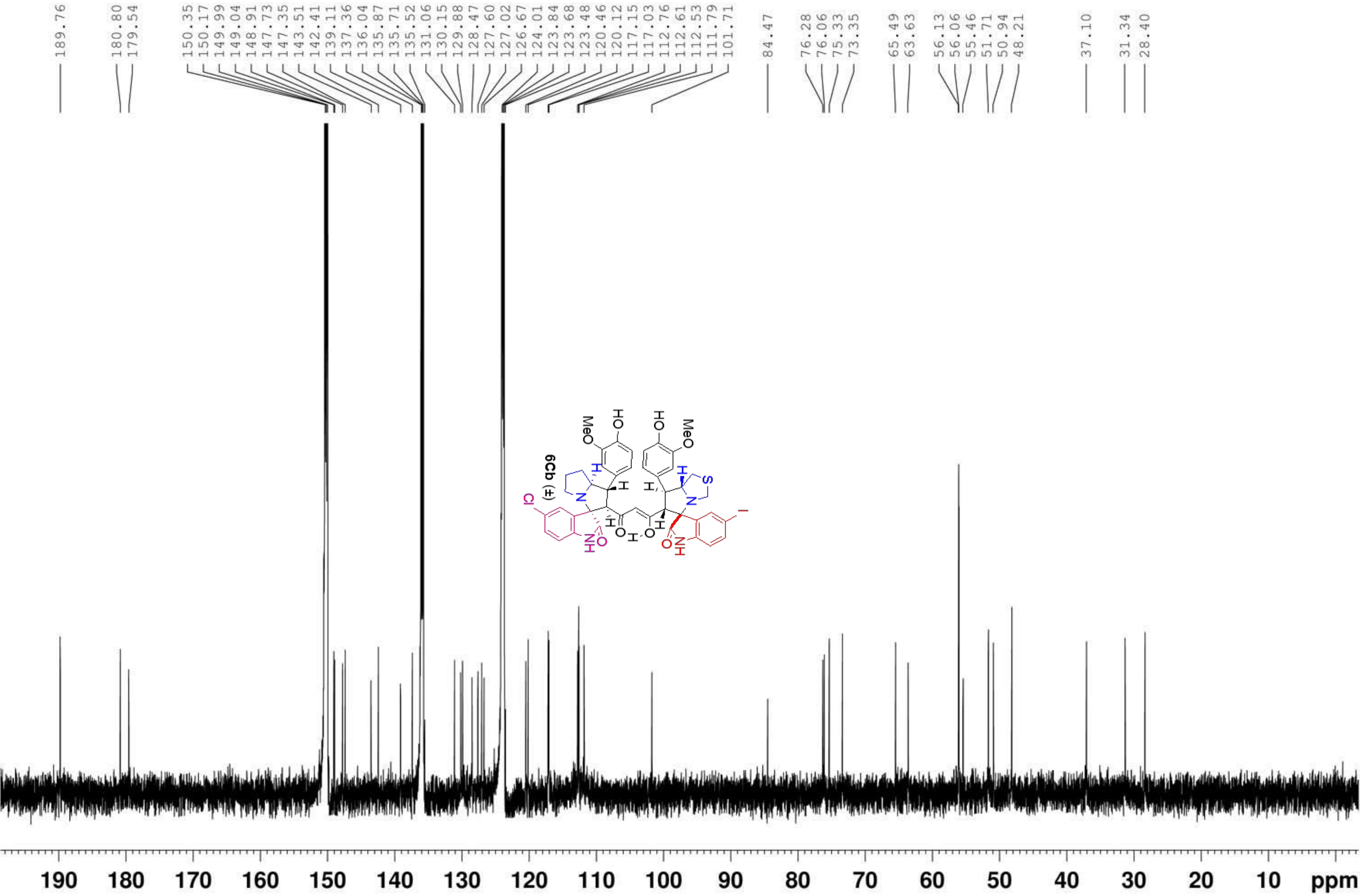

DEPT-135

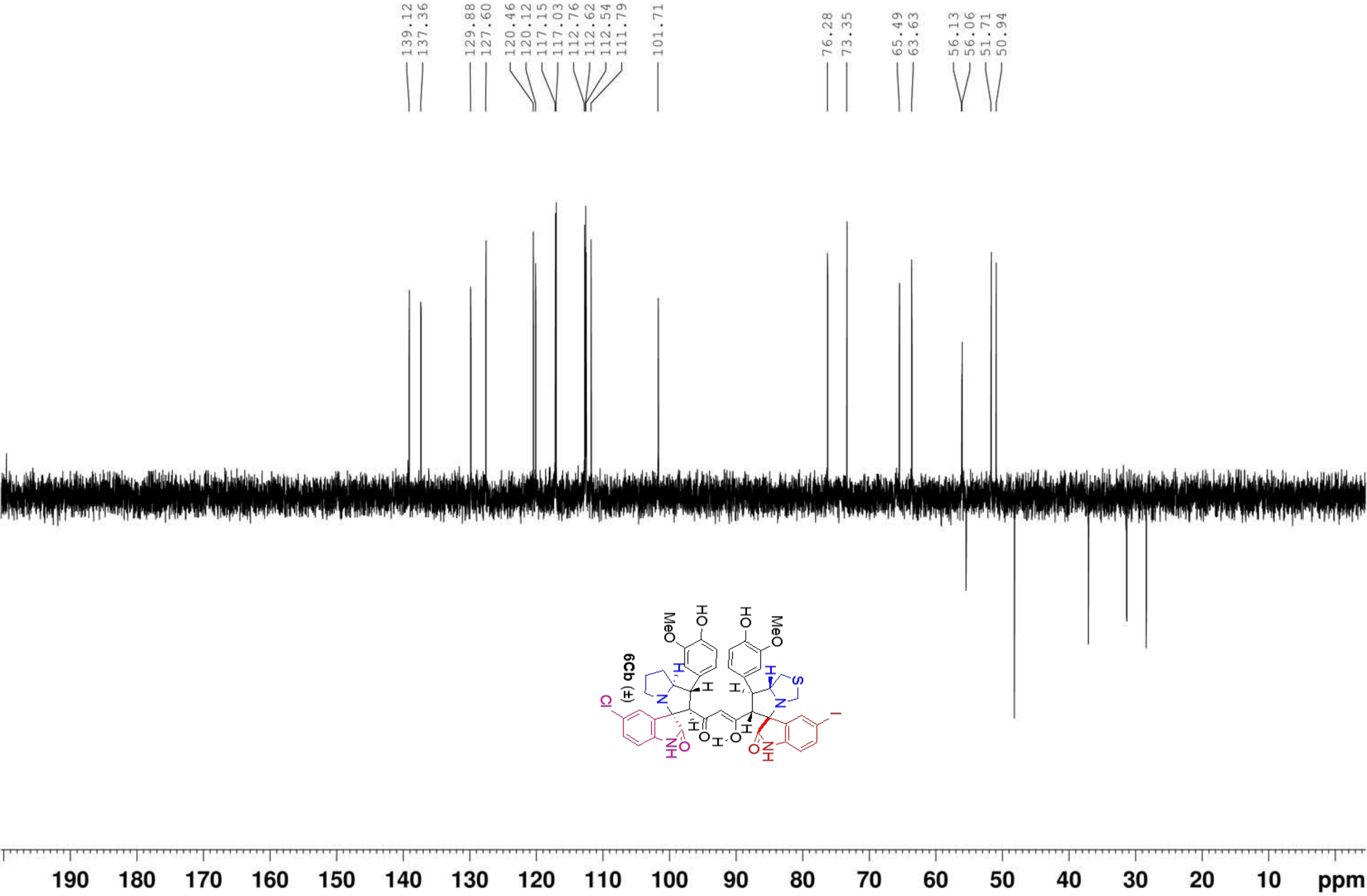
139.12
137.36
129.88
127.60
120.46
120.12
117.15
117.03
112.76
112.62
112.54
111.79
101.71
76.28
73.35
65.49
63.63
56.13
56.06
51.71
50.94
MeO
HO
HO
MeO
S
N
N
H
NH
O
Cl
I
6Cb (±)
190 180 170 160 150 140 130 120 110 100 90 80 70 60 50 40 30 20 10 ppm

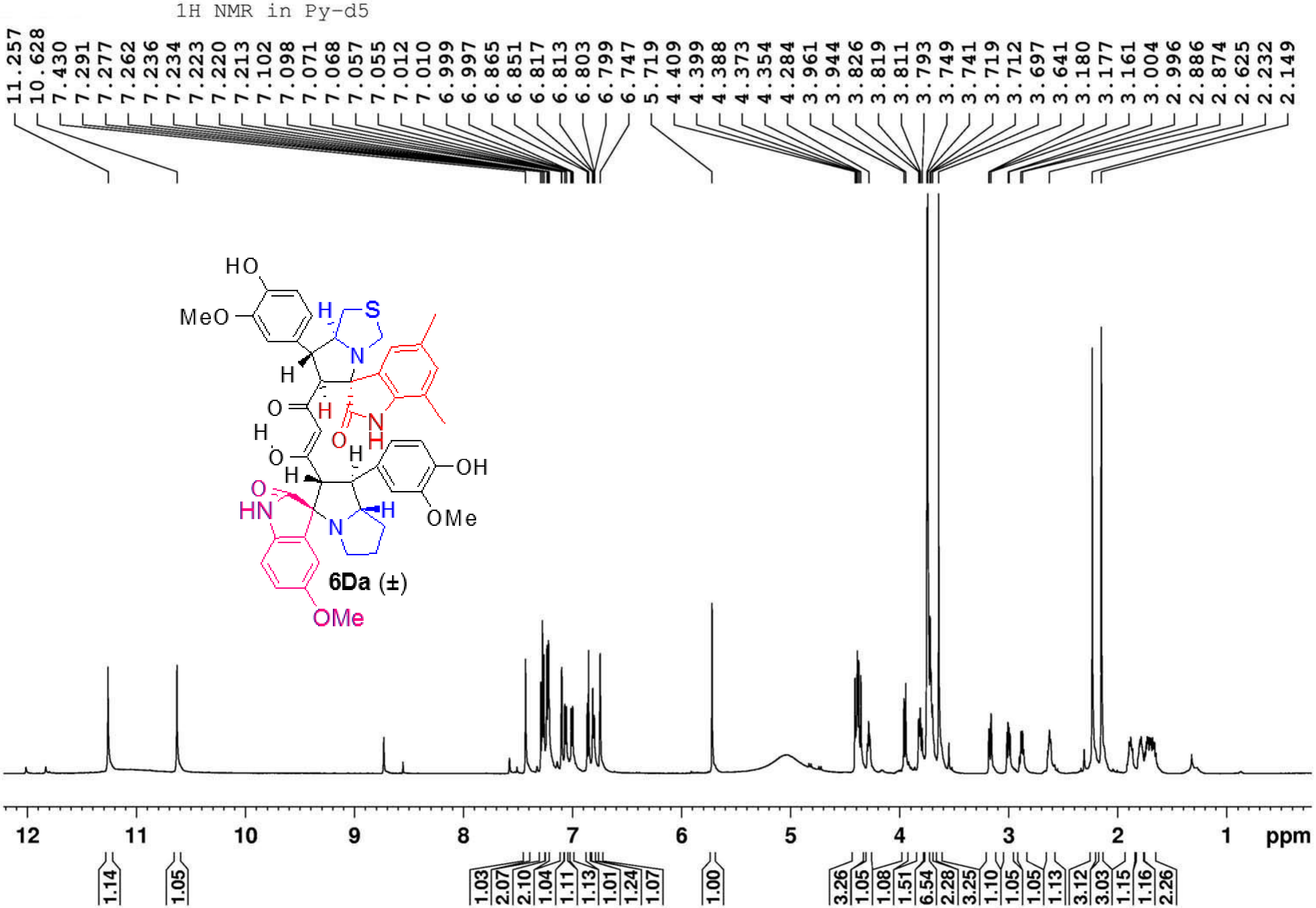
1H NMR in Py-d5
11.257 10.628 7.430 7.291 7.277 7.262 7.236 7.234 7.223 7.220 7.213 7.102 7.098 7.071 7.068 7.057 7.055 7.012 7.010 6.999 6.997 6.865 6.851 6.817 6.813 6.803 6.799 6.747 5.719 4.409 4.399 4.388 4.373 4.354 4.284 3.961 3.944 3.826 3.819 3.811 3.793 3.749 3.741 3.719 3.712 3.697 3.641 3.180 3.177 3.161 3.004 2.996 2.886 2.874 2.625 2.232 2.149
HO
MeO
S
N
O
H
HN
OH
OMe
6Da (±)
12 11 10 9 8 7 6 5 4 3 2 1 ppm
1.14 1.05 1.03 2.07 2.10 1.04 1.11 1.13 1.01 1.24 1.07 1.00 3.26 1.05 1.08 1.51 6.54 2.28 3.25 1.10 1.05 1.05 1.13 3.12 3.03 1.15 1.16 2.26

13C-NMR in Py-d5

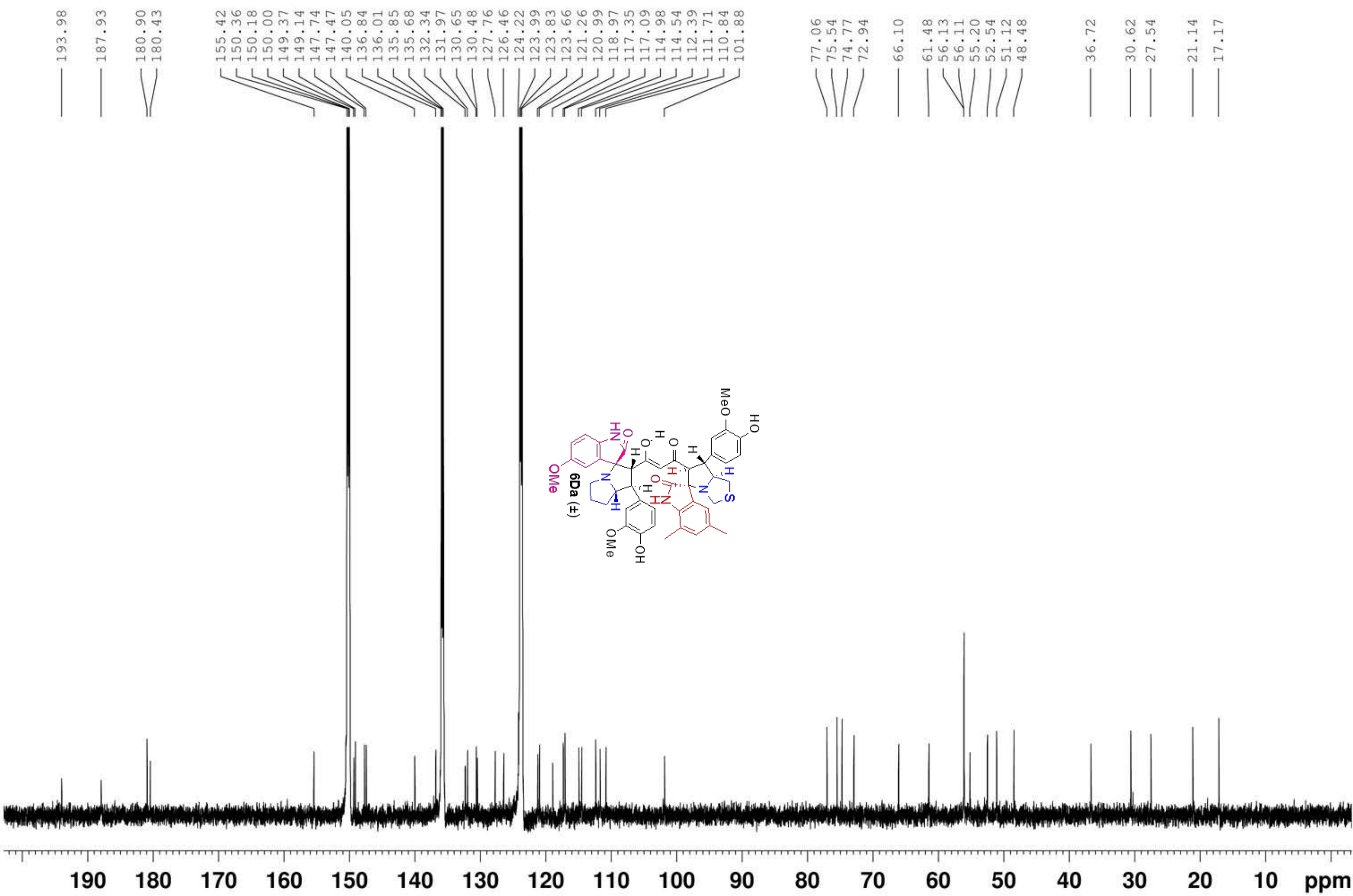

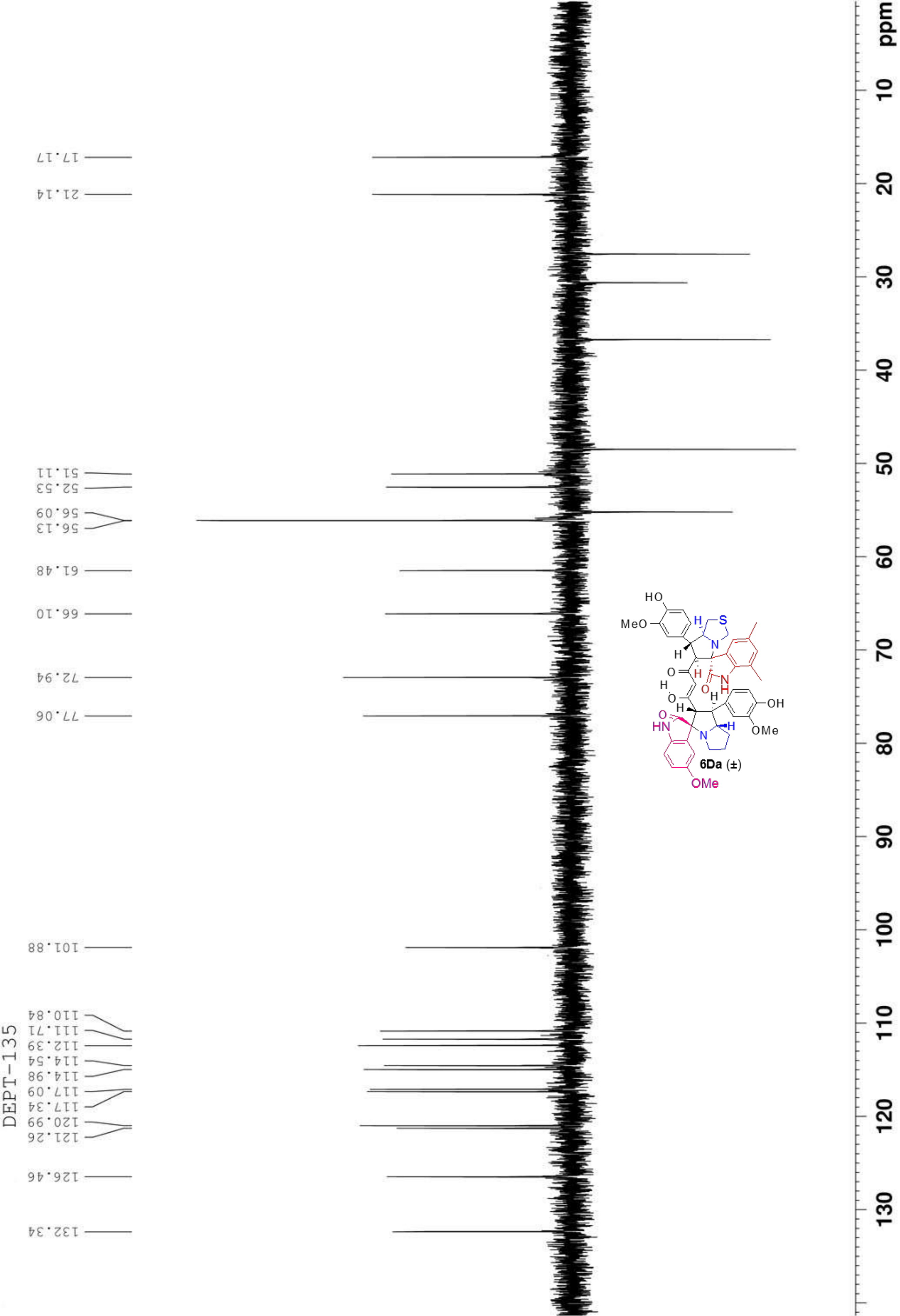

DEPT-135
17.17
21.14
51.11
52.53
56.09
56.13
61.48
66.10
72.94
77.06
101.88
110.84
111.71
112.39
114.54
114.98
117.09
117.34
120.99
121.26
126.46
132.34
ppm
HO
MeO
S
N
H
O
NH
OH
OMe
HN
OMe
6Da (±)

1H NMR in Py-d5

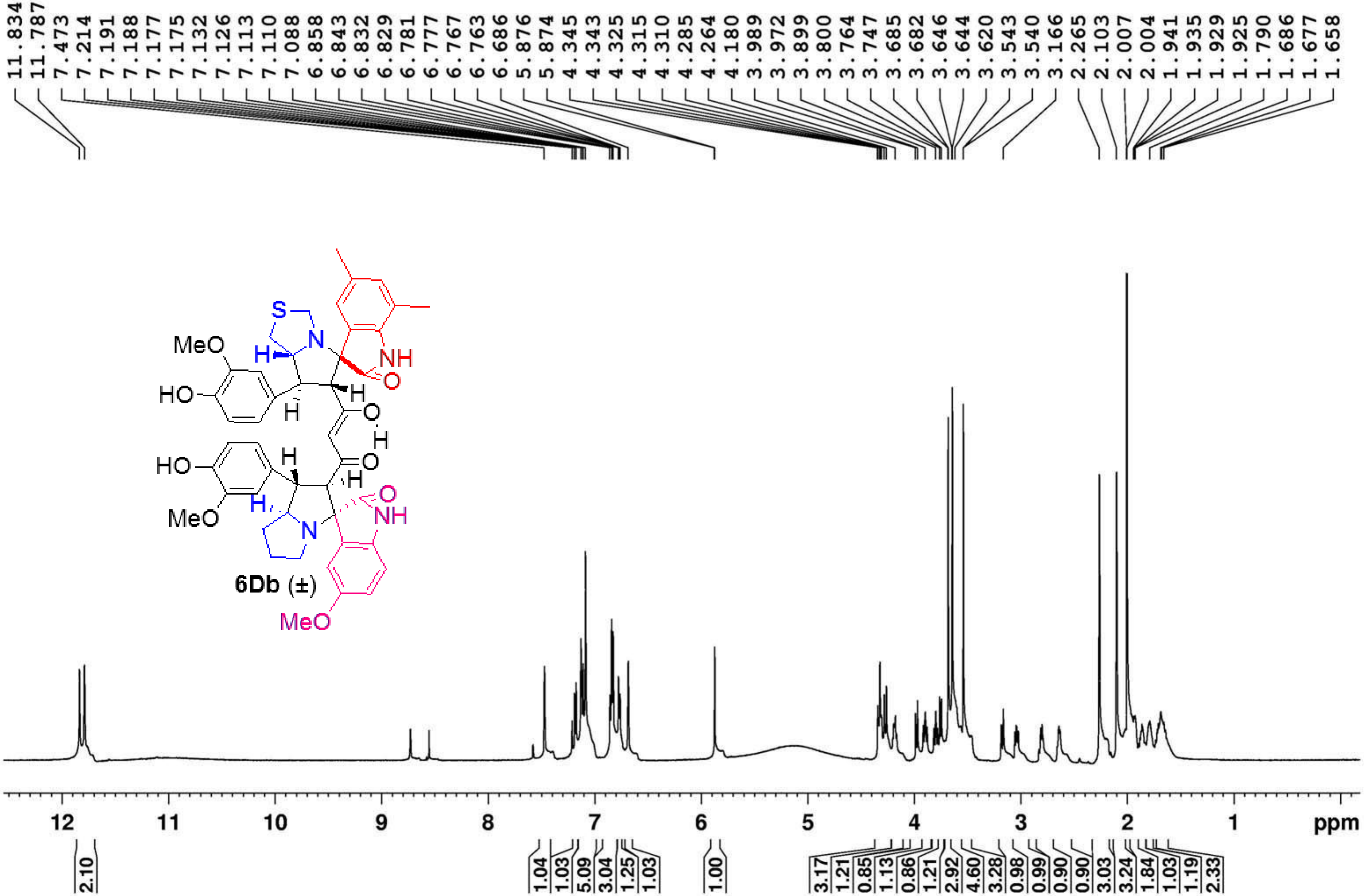

13C-NMR in Py-d5

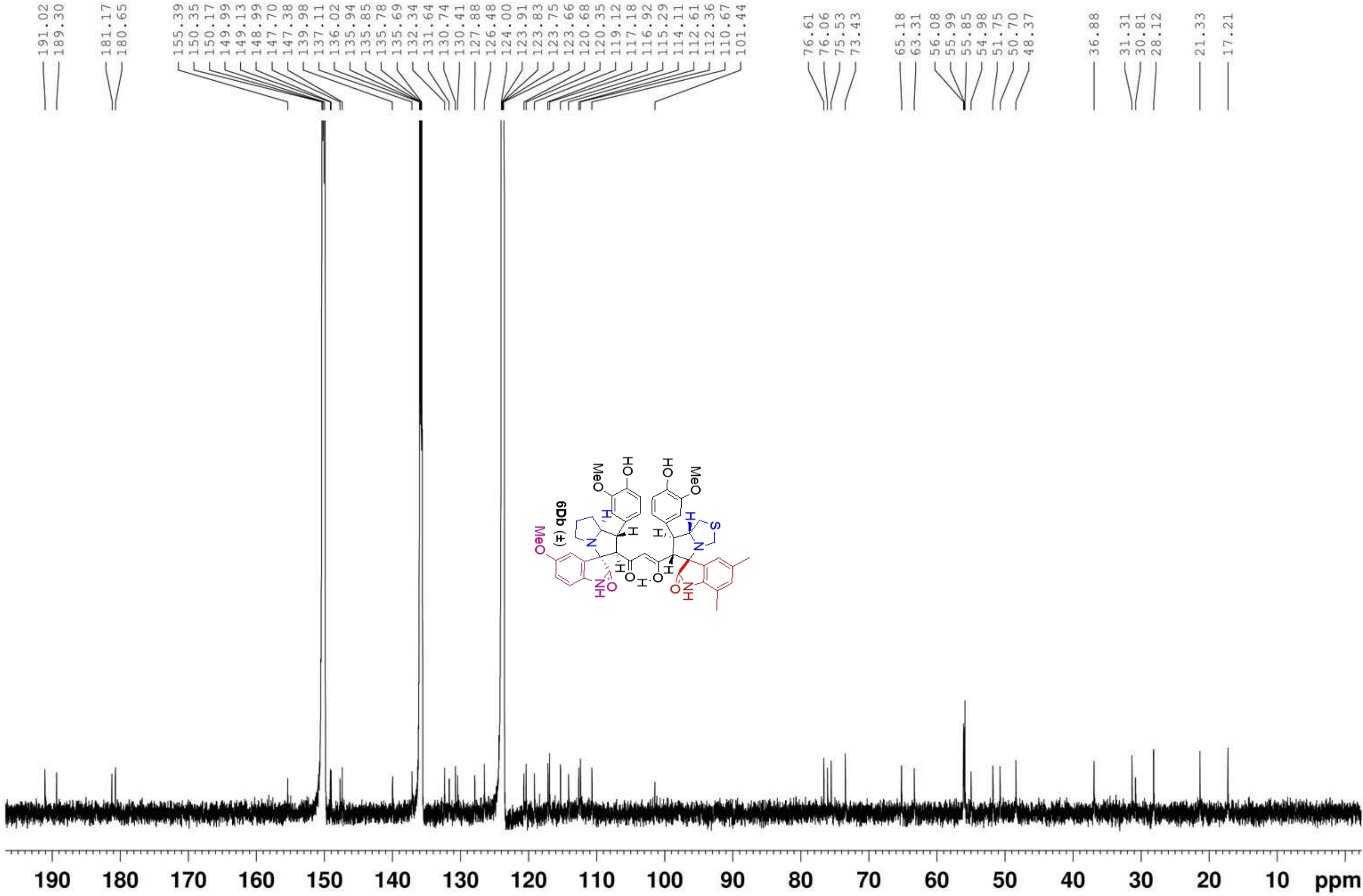

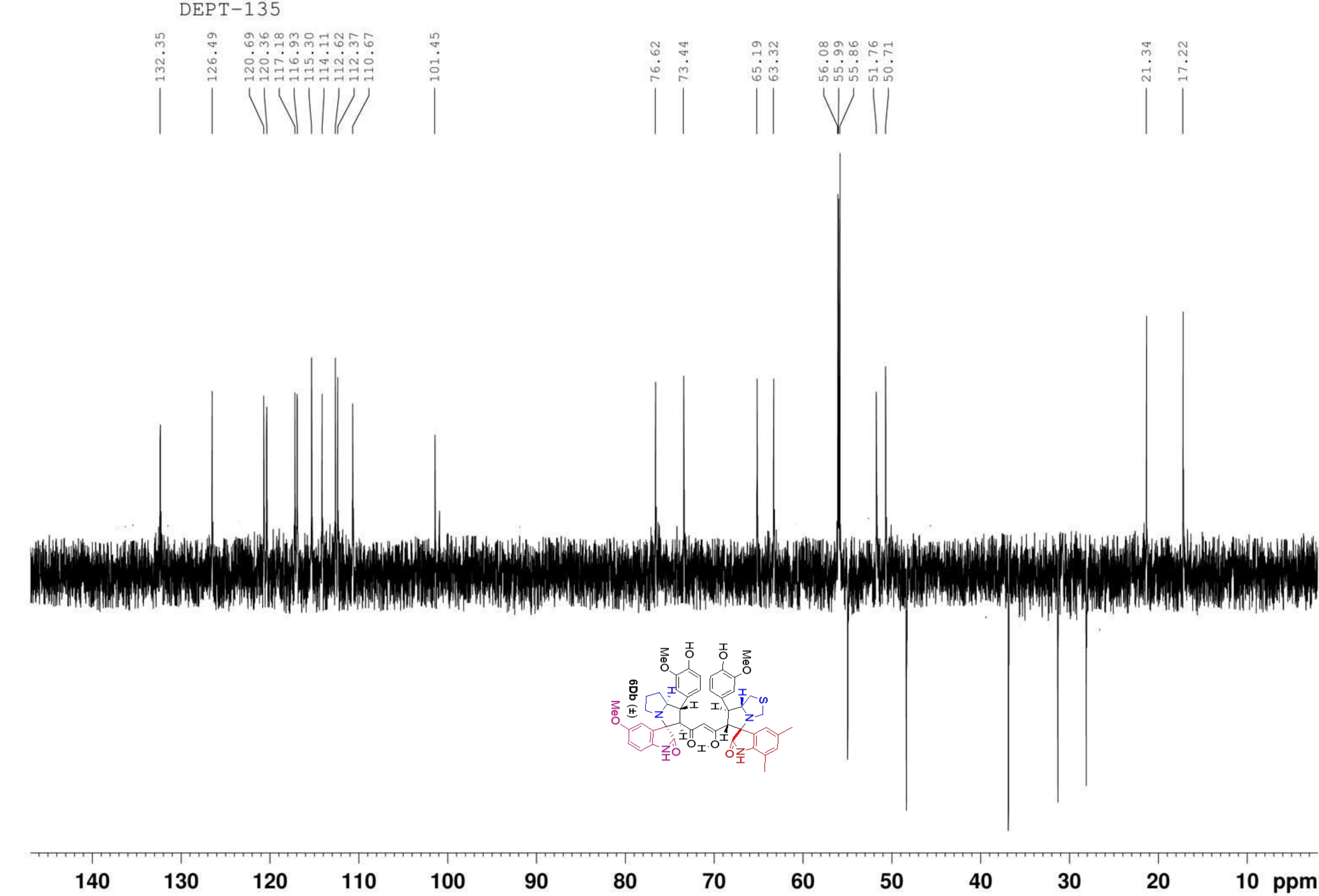
DEPT-135
132.35
126.49
120.69
120.36
117.18
116.93
115.30
114.11
112.62
112.37
110.67
101.45
76.62
73.44
65.19
63.32
56.08
55.99
55.86
51.76
50.71
21.34
17.22
MeO
HO
HO
MeO
S
N
H
NH
O
N
NH
O
MeO
6Db (±)
140
130
120
110
100
90
80
70
60
50
40
30
20
10 ppm

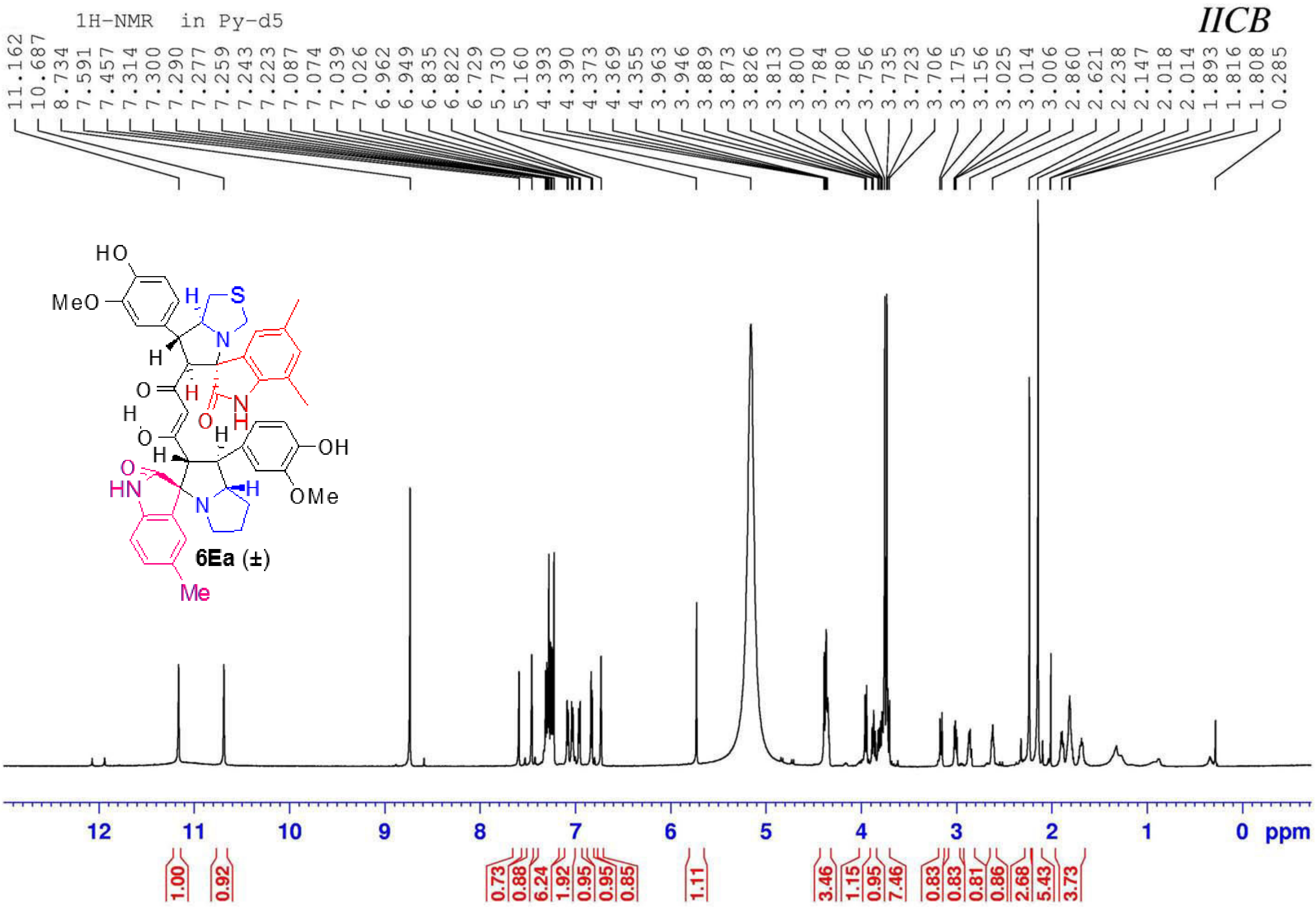
1H-NMR in Py-d5
IICB
11.162 10.687 8.734 7.591 7.457 7.314 7.300 7.290 7.277 7.259 7.243 7.223 7.087 7.074 7.039 7.026 6.962 6.949 6.835 6.822 6.729 5.730 5.160 4.393 4.390 4.373 4.369 4.355 3.963 3.946 3.889 3.873 3.826 3.813 3.800 3.784 3.780 3.756 3.735 3.723 3.706 3.175 3.156 3.025 3.014 3.006 2.860 2.621 2.238 2.147 2.018 2.014 1.893 1.816 1.808 0.285
HO
MeO
S
N
H
O
NH
OH
OMe
HN
Me
6Ea (±)
12 11 10 9 8 7 6 5 4 3 2 1 0 ppm
1.00 0.92 0.73 0.88 6.24 1.92 0.95 0.95 0.85 1.11 3.46 1.15 0.95 7.46 0.83 0.83 0.81 0.86 2.68 5.43 3.73

13C-NMR in Py-d5

IICB

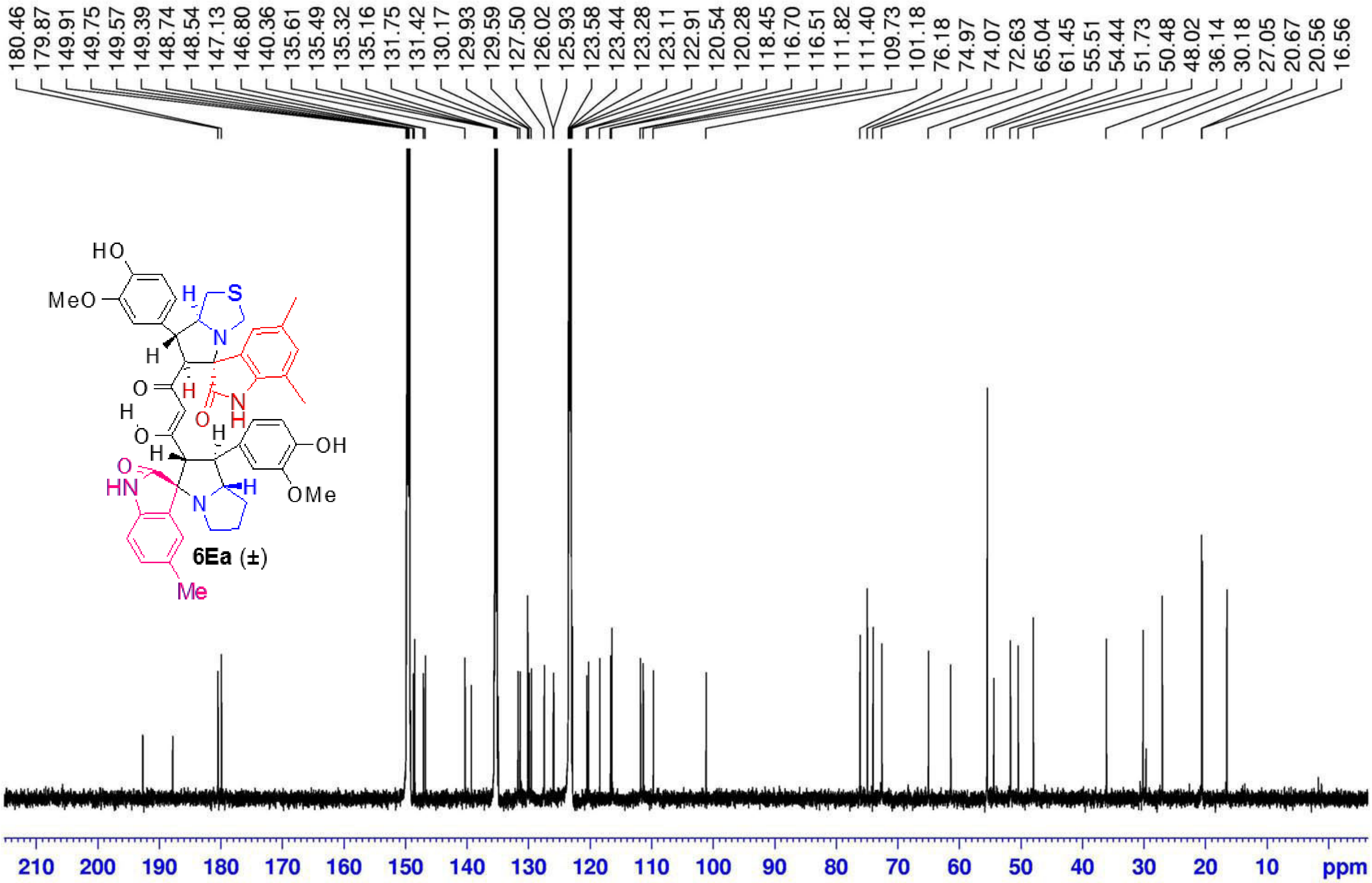

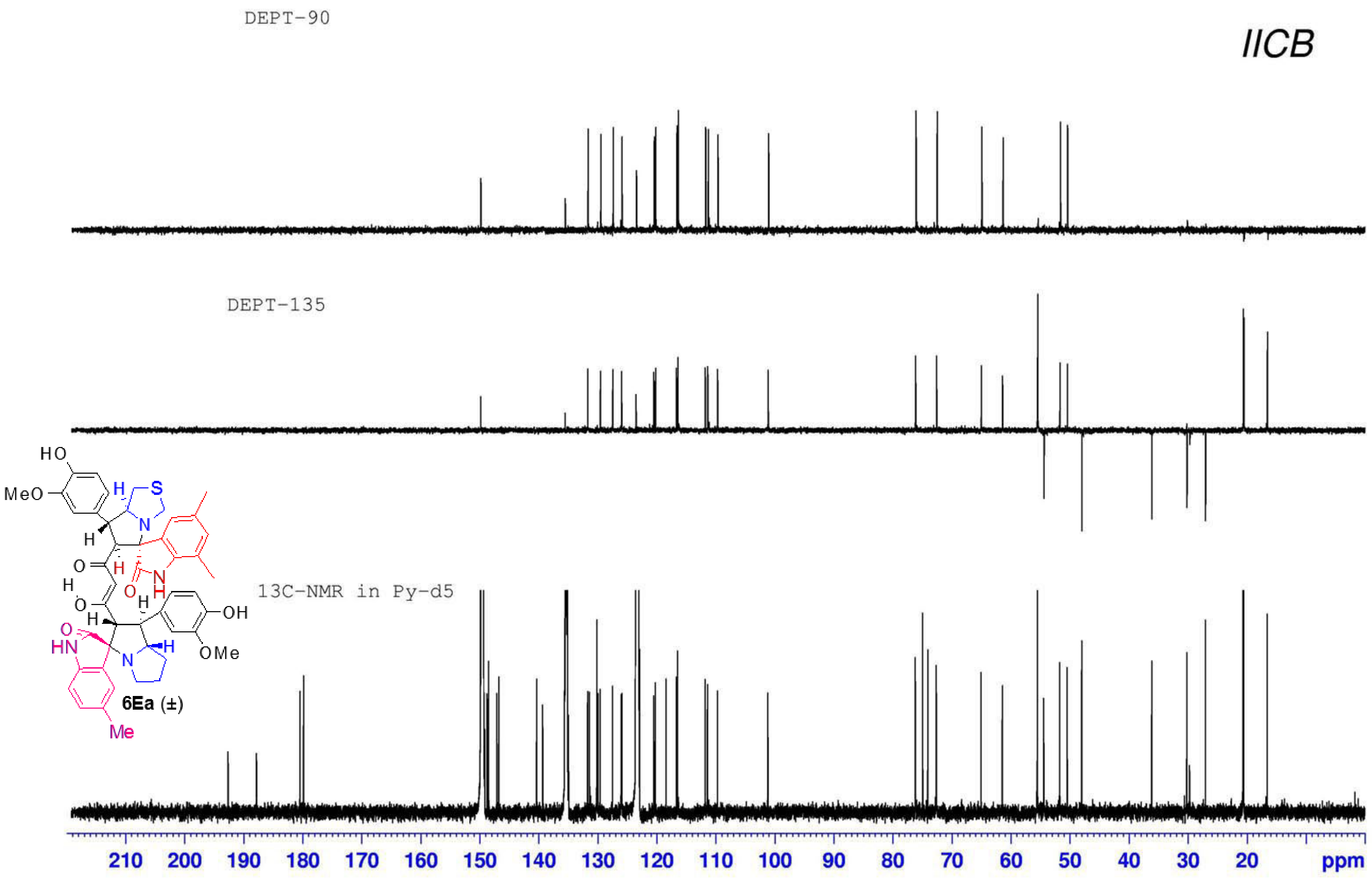
DEPT-90
IICB
DEPT-135
13C-NMR in Py-d5
HO
MeO
OH
OMe
HN
Me
6Ea (±)
210
200
190
180
170
160
150
140
130
120
110
100
90
80
70
60
50
40
30
20
ppm

1H-NMR in Py-d5

IICB

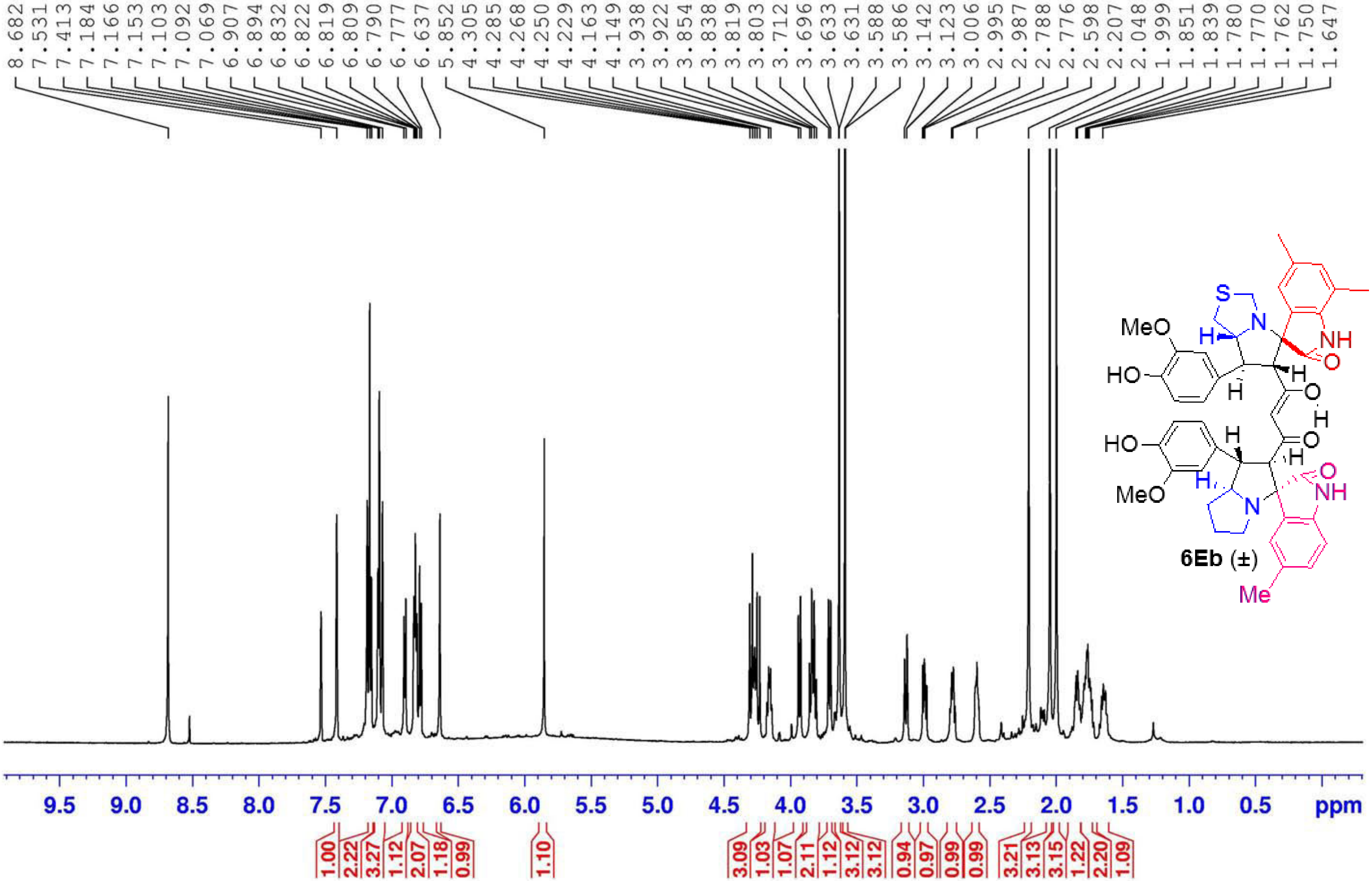

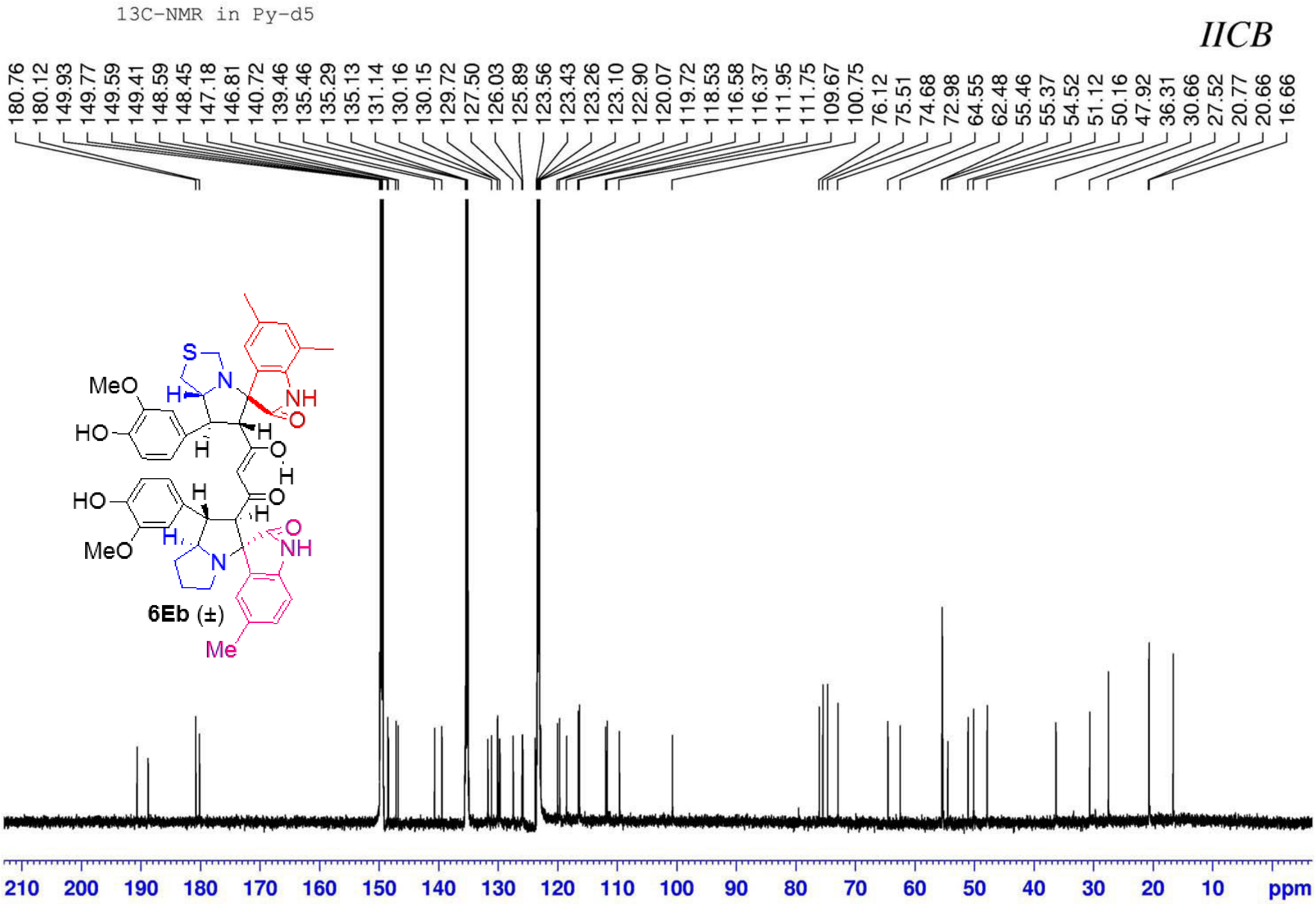
13C-NMR in Py-d5
IICB
180.76
180.12
149.93
149.77
149.59
149.41
148.59
148.45
147.18
146.81
140.72
139.46
135.46
135.29
135.13
131.14
130.16
130.15
129.72
127.50
126.03
125.89
123.56
123.43
123.26
123.10
122.90
120.07
119.72
118.53
116.58
116.37
111.95
111.75
109.67
100.75
76.12
75.51
74.68
72.98
64.55
62.48
55.46
55.37
54.52
51.12
50.16
47.92
36.31
30.66
27.52
20.77
20.66
16.66
MeO
HO
S
H
N
NH
O
H
H
O
H
HO
H
O
H
O
MeO
H
N
NH
6Eb (±)
Me
210
200
190
180
170
160
150
140
130
120
110
100
90
80
70
60
50
40
30
20
10
ppm

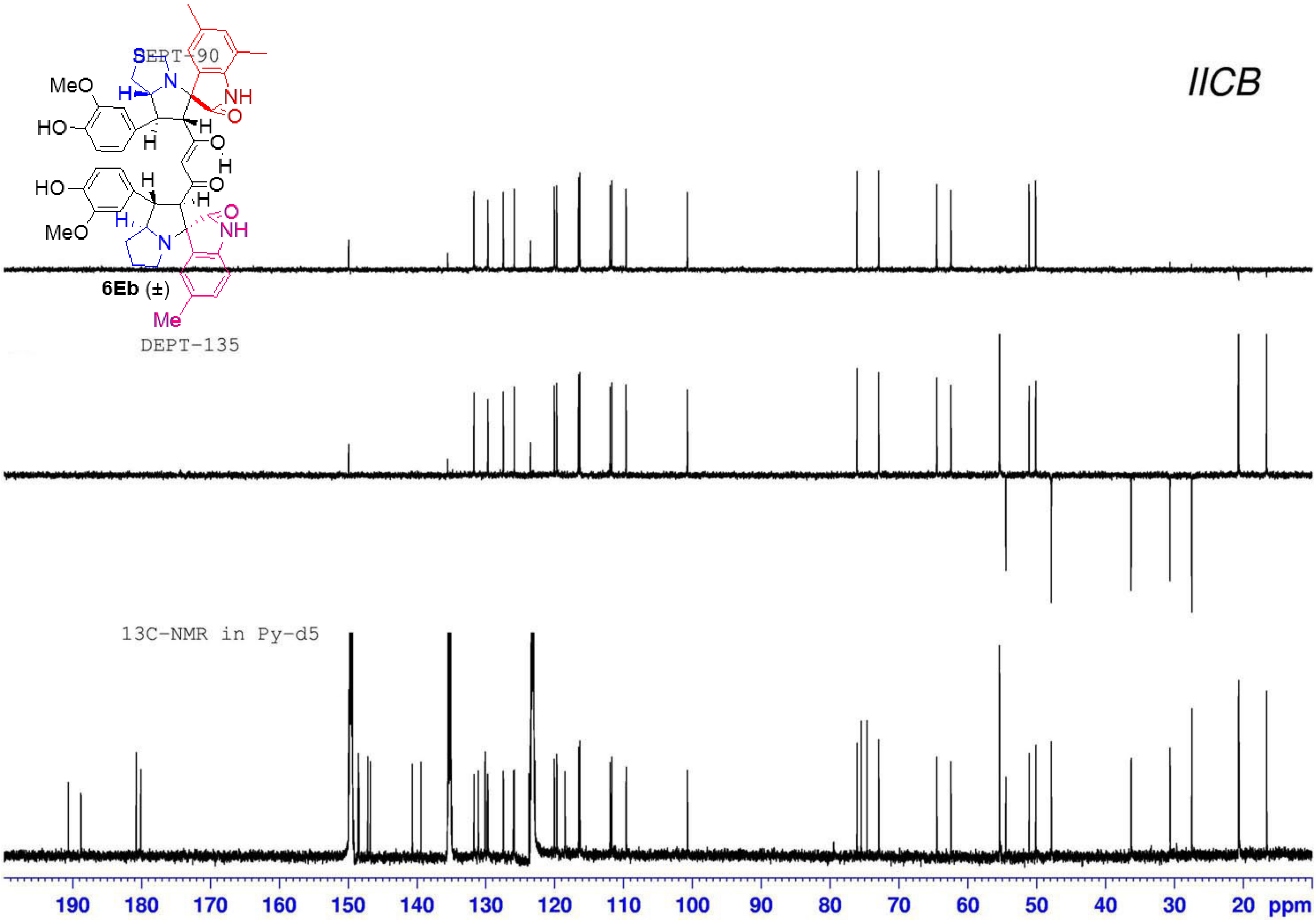

IICB
DEPT-90
DEPT-135
13C-NMR in Py-d5
MeO
HO
NH
O
H
N
6Eb (±)
Me
190
180
170
160
150
140
130
120
110
100
90
80
70
60
50
40
30
20
ppm

1H NMR in Py-d5

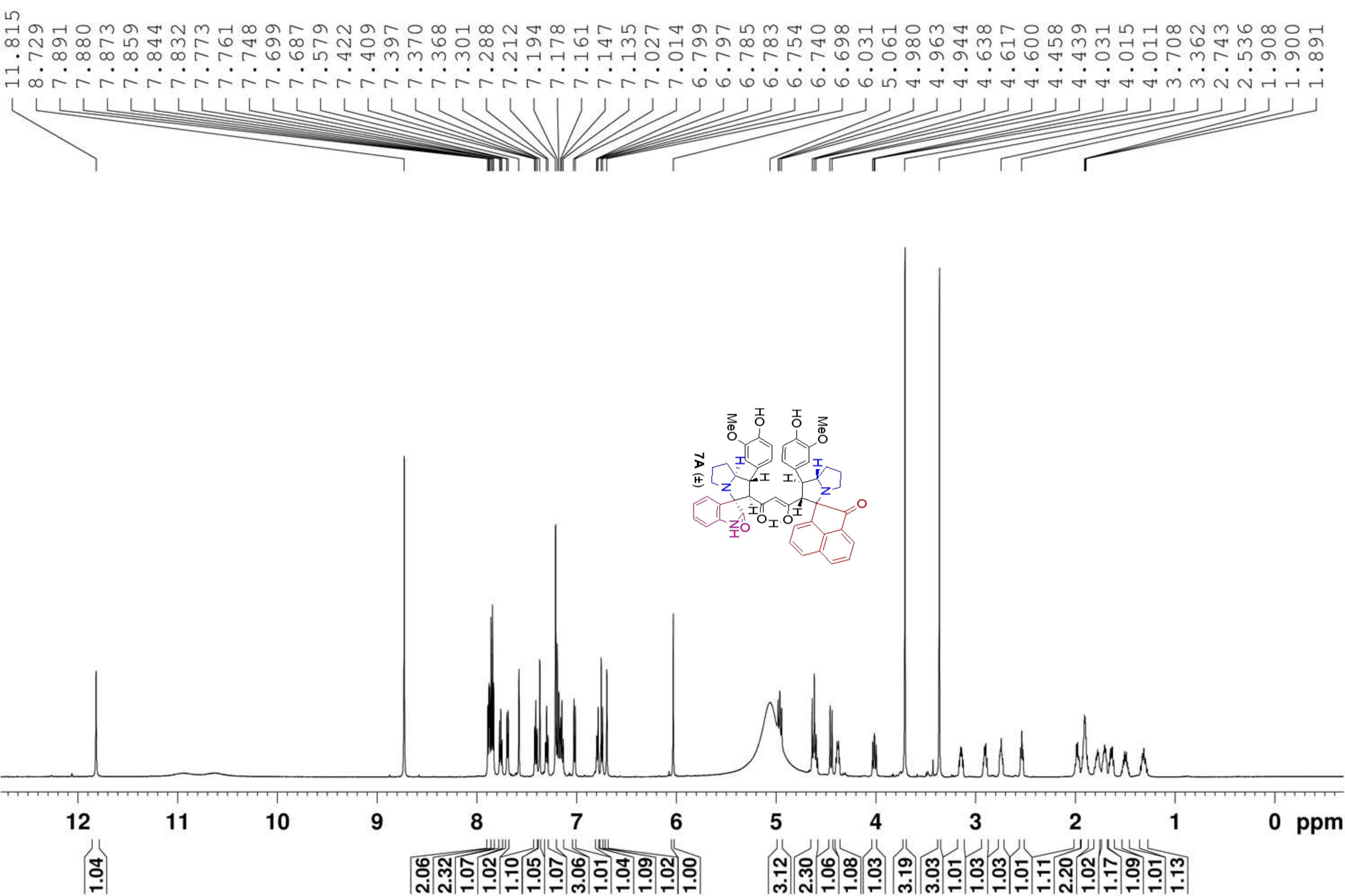

13C-NMR in Py-d5

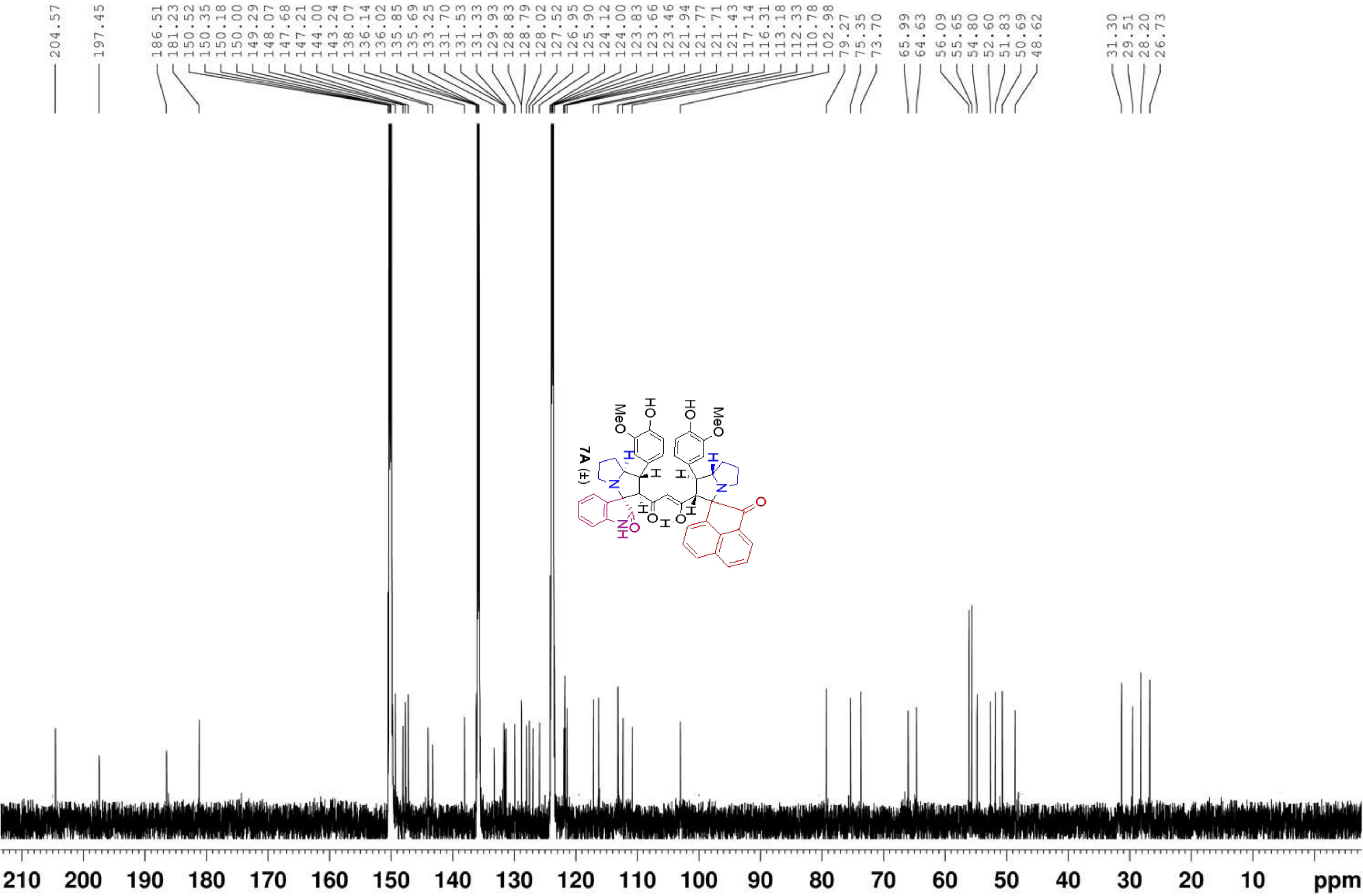

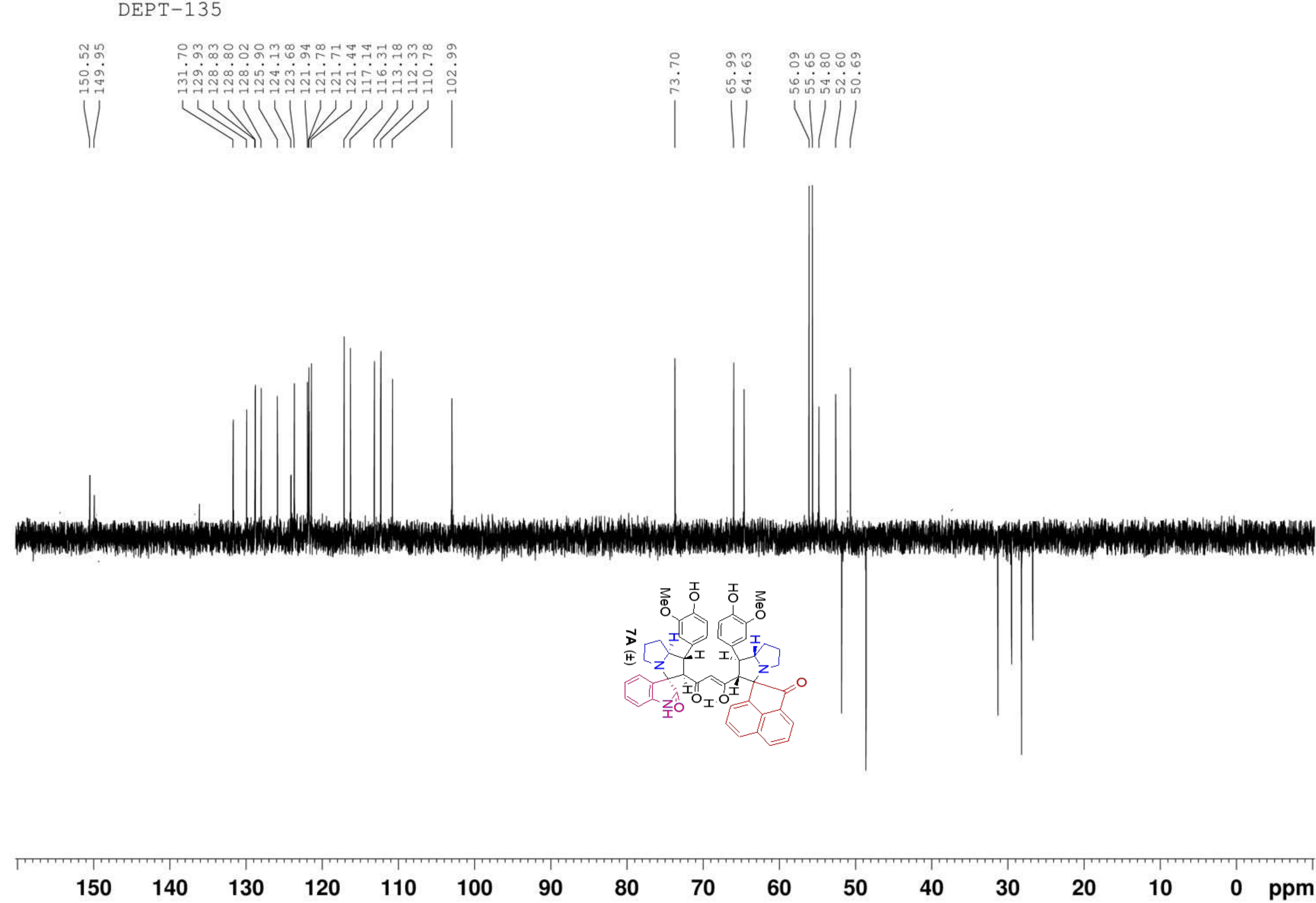

DEPT-135
150.52
149.95
131.70
129.93
128.83
128.80
128.02
125.90
124.13
123.68
121.94
121.78
121.71
121.44
117.14
116.31
113.18
112.33
110.78
102.99
73.70
65.99
64.63
56.09
55.65
54.80
52.60
50.69
MeO
HO
7A (±)
150
140
130
120
110
100
90
80
70
60
50
40
30
20
10
0
ppm

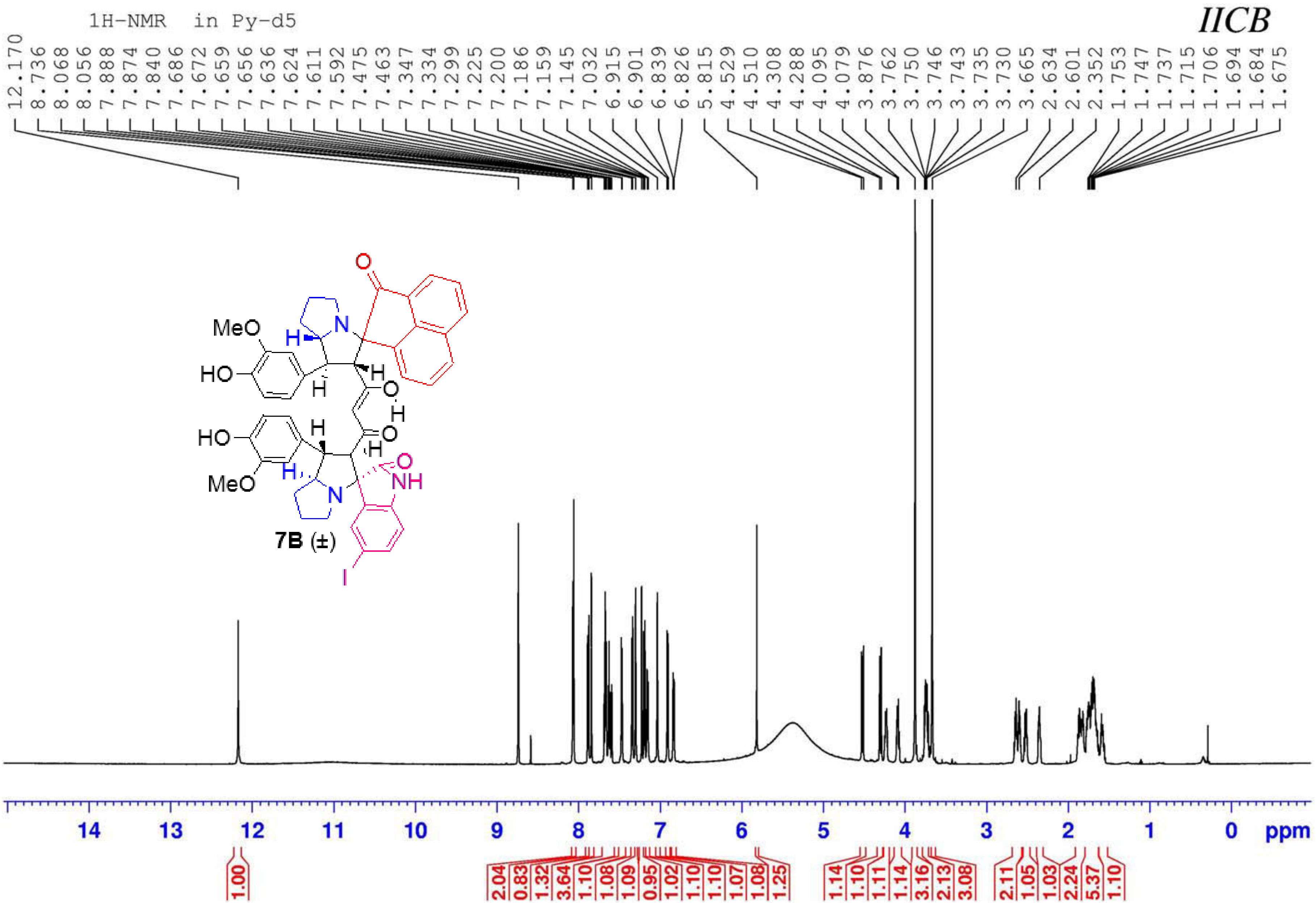
1H-NMR in Py-d5
IICB
12.170 8.736 8.068 8.056 7.888 7.874 7.840 7.686 7.672 7.659 7.656 7.636 7.624 7.611 7.592 7.475 7.463 7.347 7.334 7.299 7.225 7.200 7.186 7.159 7.145 7.032 6.915 6.901 6.839 6.826 5.815 4.529 4.510 4.308 4.288 4.095 4.079 3.876 3.762 3.750 3.746 3.743 3.735 3.730 3.665 2.634 2.601 2.352 1.753 1.747 1.737 1.715 1.706 1.694 1.684 1.675
MeO
HO
HO
MeO
NH
I
7B (±)
14 13 12 11 10 9 8 7 6 5 4 3 2 1 0 ppm
1.00 2.04 0.83 1.32 3.64 1.10 1.08 1.09 0.95 1.02 1.10 1.10 1.07 1.08 1.25 1.14 1.10 1.11 1.14 3.16 2.13 3.08 2.11 1.05 1.03 2.24 5.37 1.10

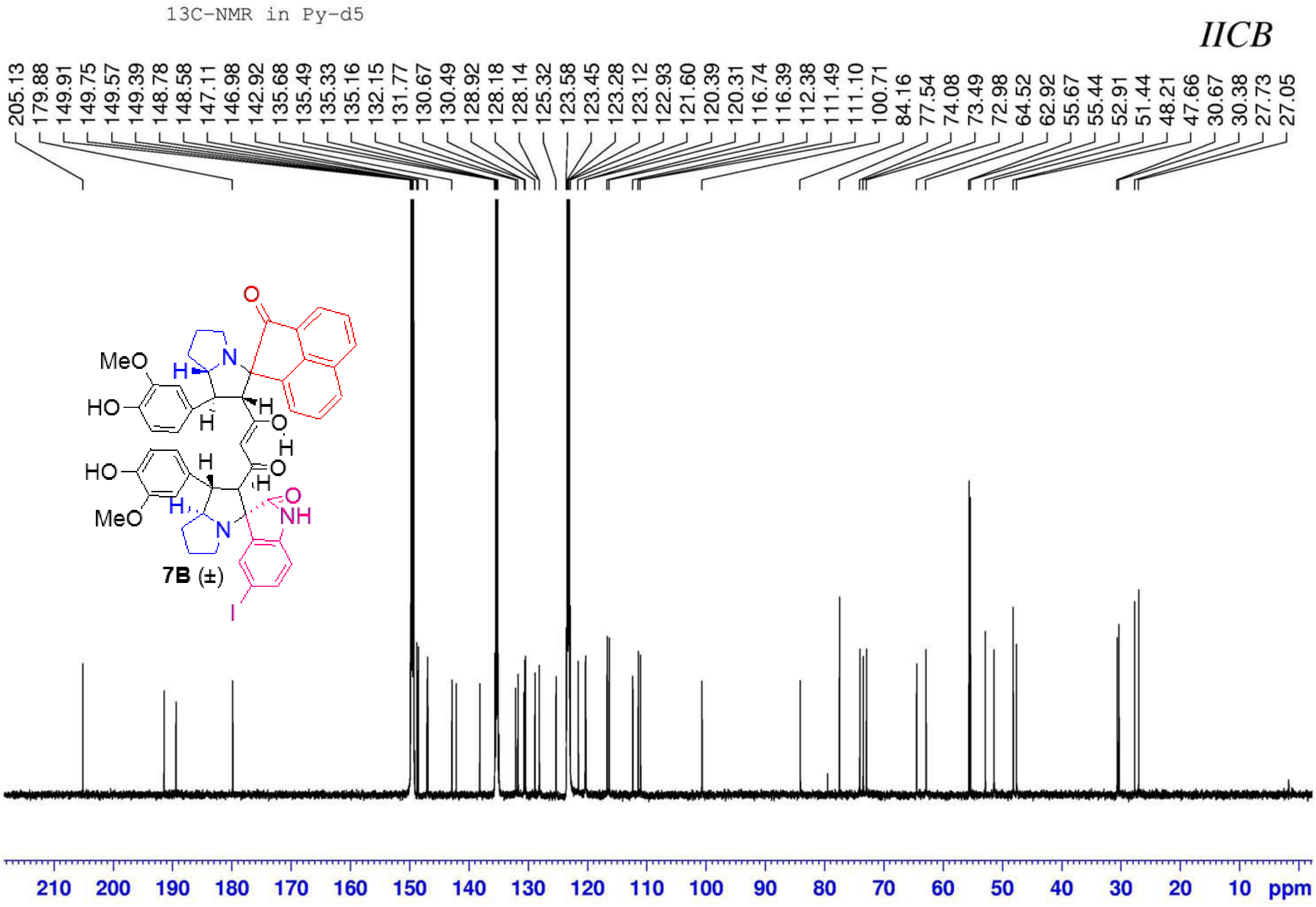
13C-NMR in Py-d5
IICB
205.13
179.88
149.91
149.75
149.57
149.39
148.78
148.58
147.11
146.98
142.92
135.68
135.49
135.33
135.16
132.15
131.77
130.67
130.49
128.92
128.18
128.14
125.32
123.58
123.45
123.28
123.12
122.93
121.60
120.39
120.31
116.74
116.39
112.38
111.49
111.10
100.71
84.16
77.54
74.08
73.49
72.98
64.52
62.92
55.67
55.44
52.91
51.44
48.21
47.66
30.67
30.38
27.73
27.05
MeO
HO
HO
MeO
N
N
O
O
O
O
NH
I
7B (±)
210
200
190
180
170
160
150
140
130
120
110
100
90
80
70
60
50
40
30
20
10
ppm

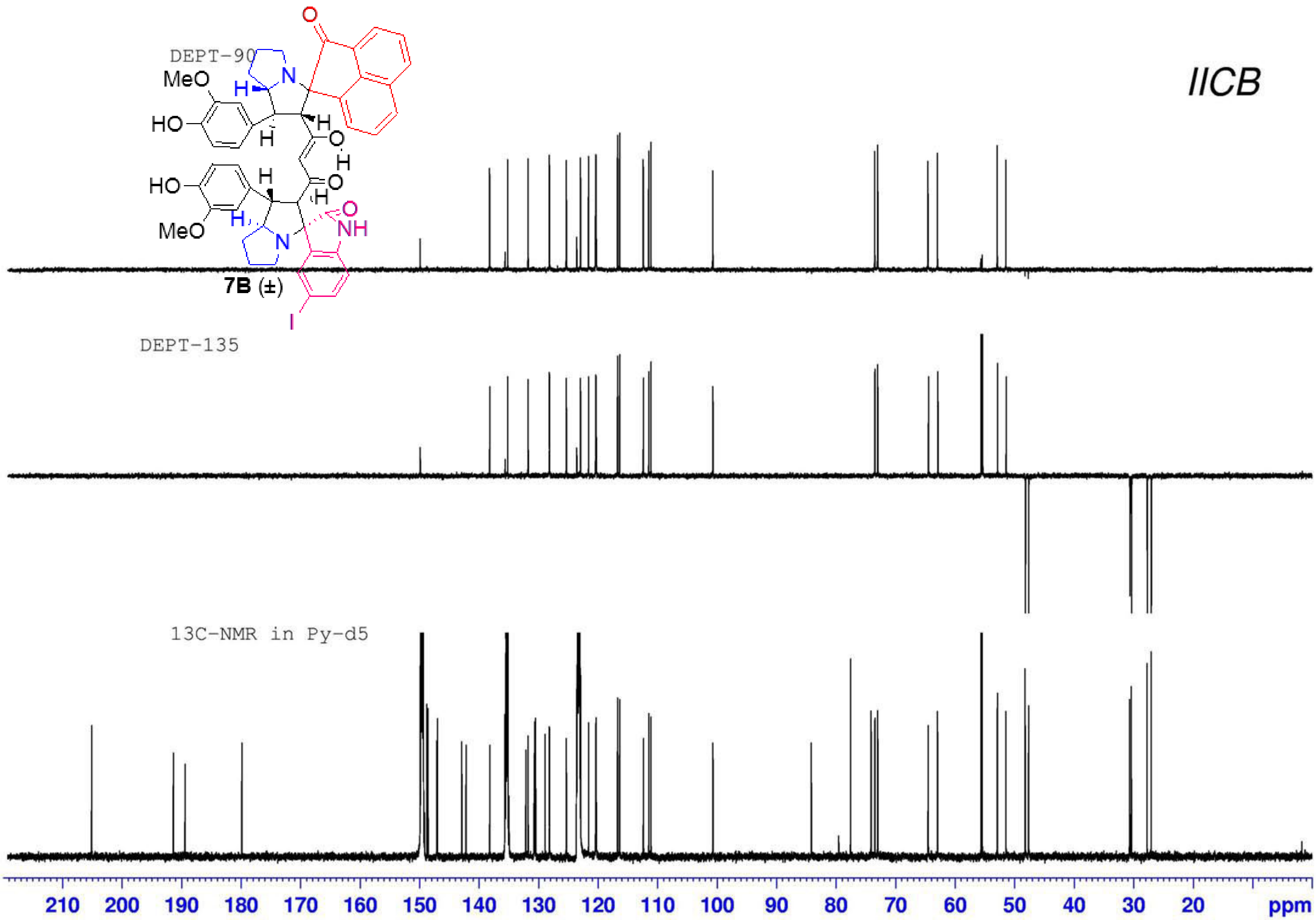

IICB
DEPT-90
MeO
HO
HO
MeO
H
N
O
NH
I
7B (±)
DEPT-135
13C-NMR in Py-d5
210
200
190
180
170
160
150
140
130
120
110
100
90
80
70
60
50
40
30
20
ppm

1H NMR in Py-d5

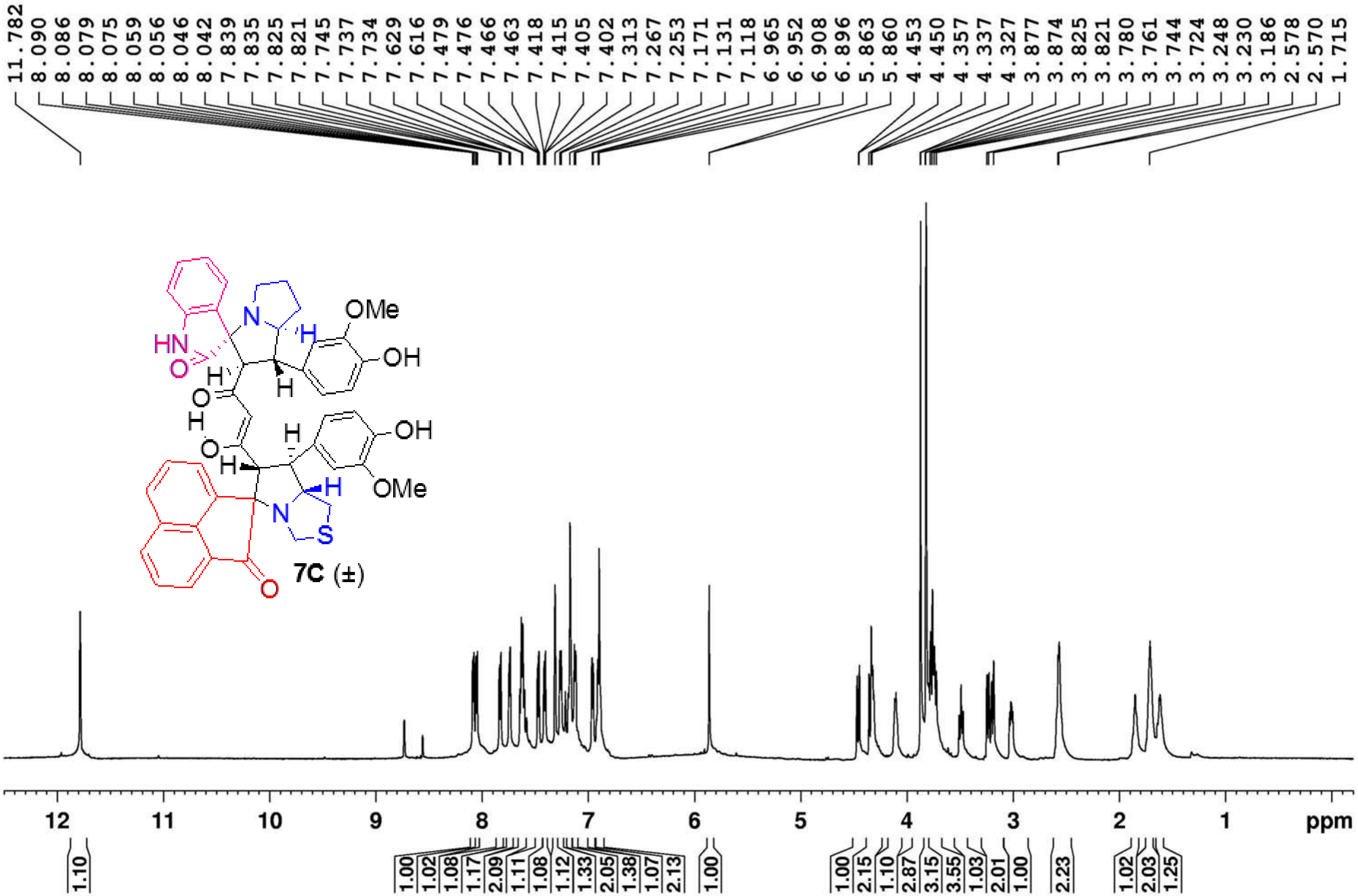

13C-NMR in Py-d5

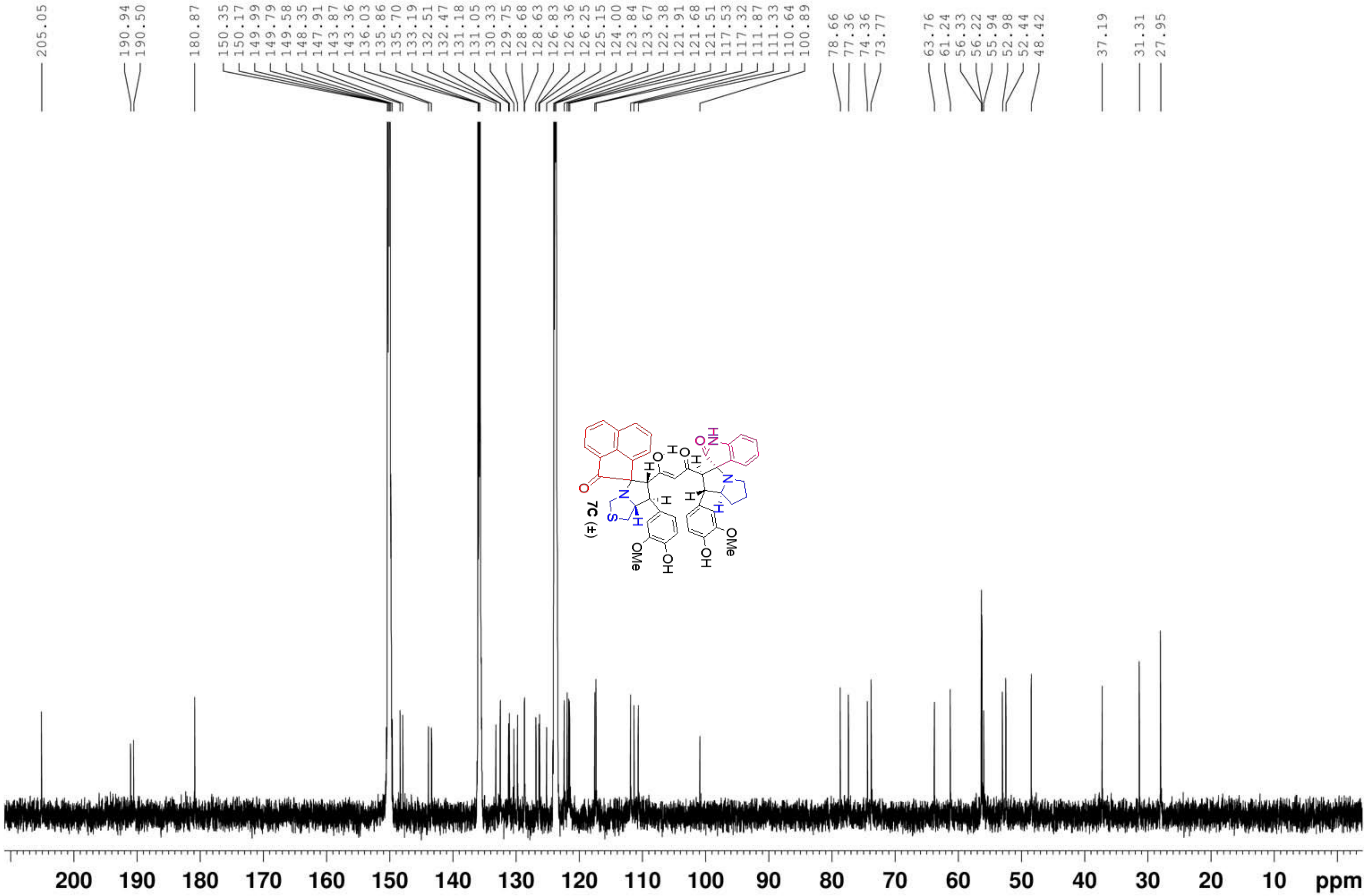

DEPT-135

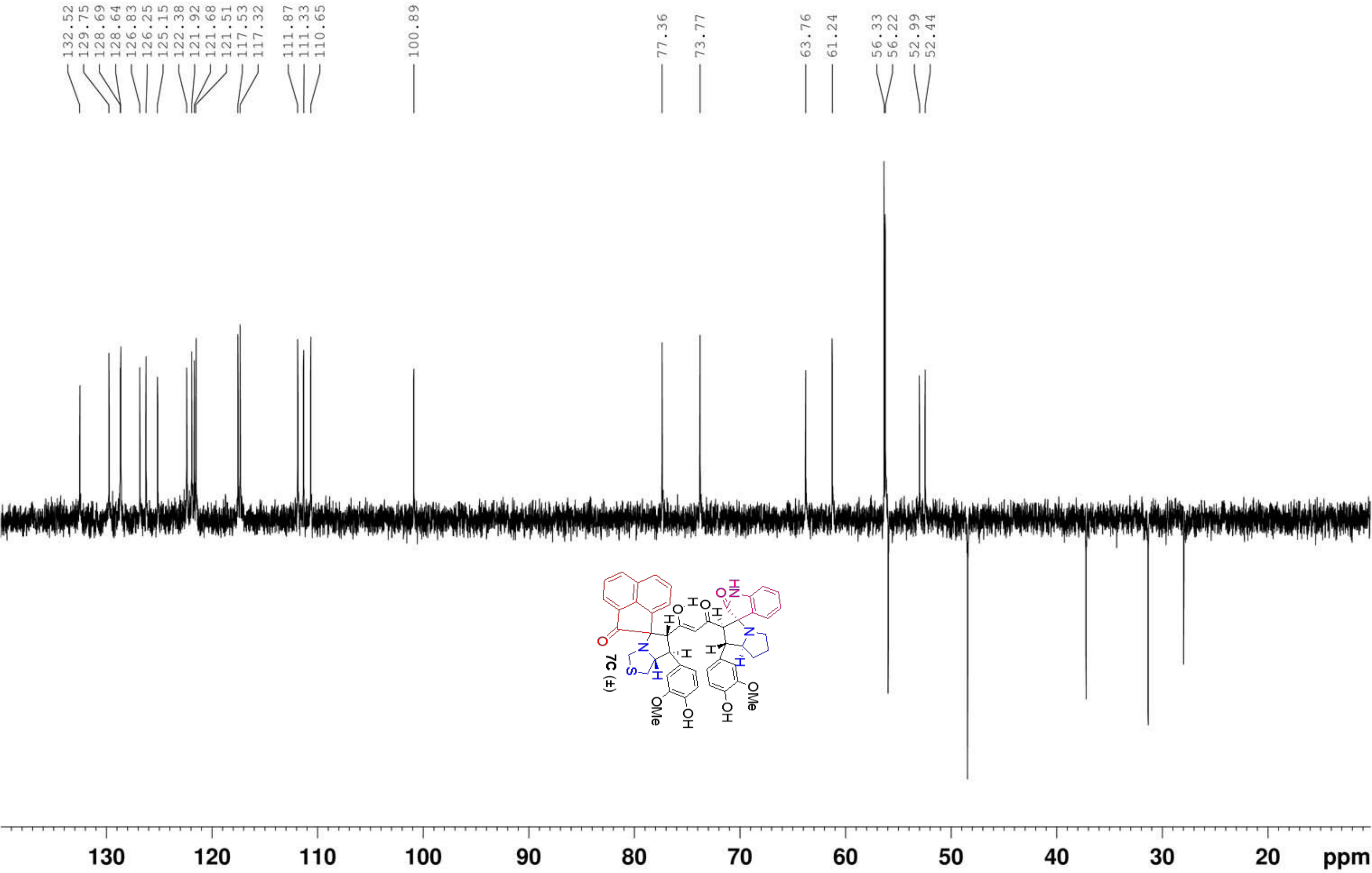

132.52
129.75
128.69
128.64
126.83
126.25
125.15
122.38
121.92
121.68
121.51
117.53
117.32
111.87
111.33
110.65
100.89
77.36
73.77
63.76
61.24
56.33
56.22
52.99
52.44
HN
O
H
OMe
OH
S
7C (±)
130
120
110
100
90
80
70
60
50
40
30
20
ppm

1H-NMR in Py-d5

IICB

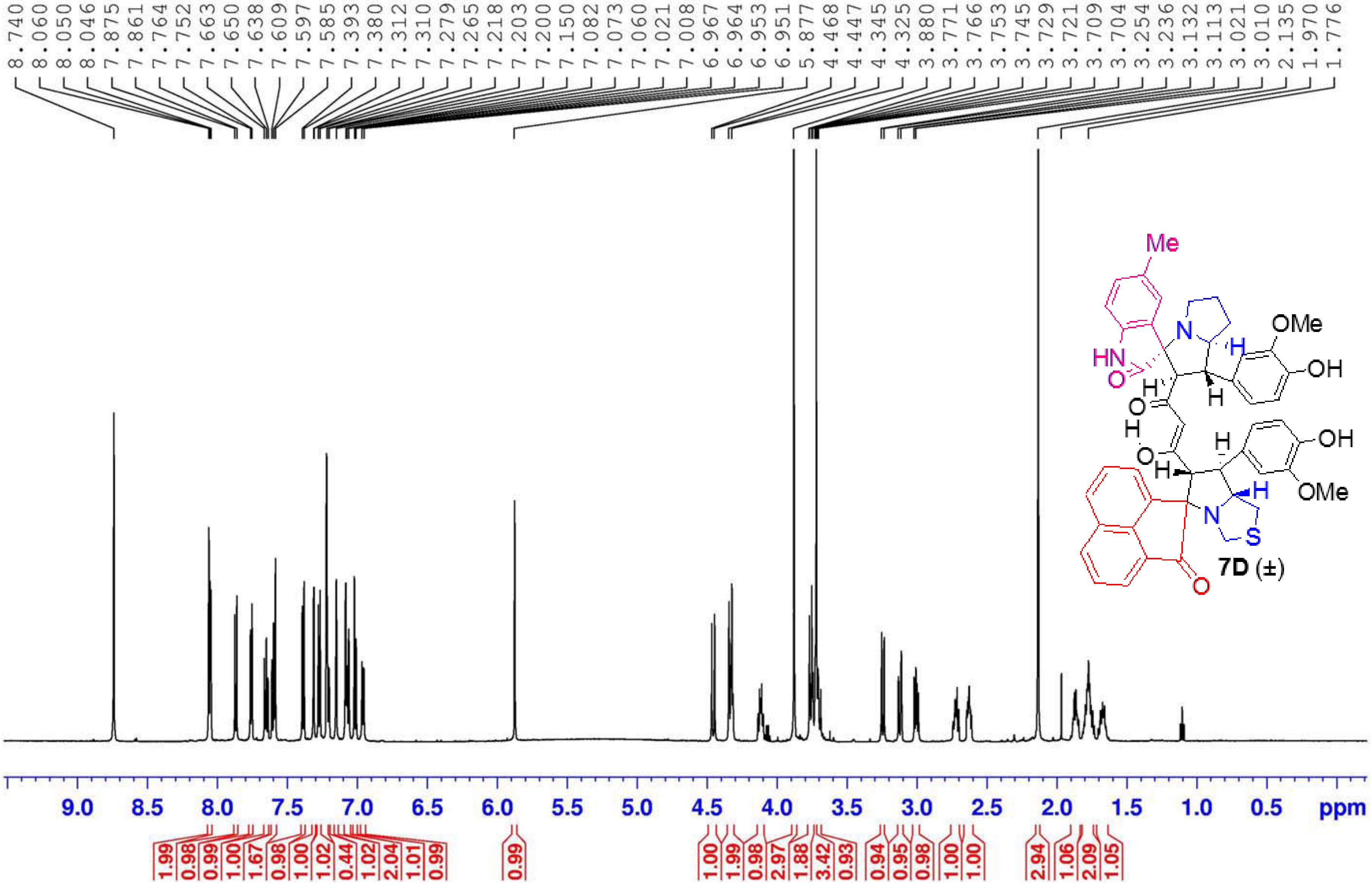

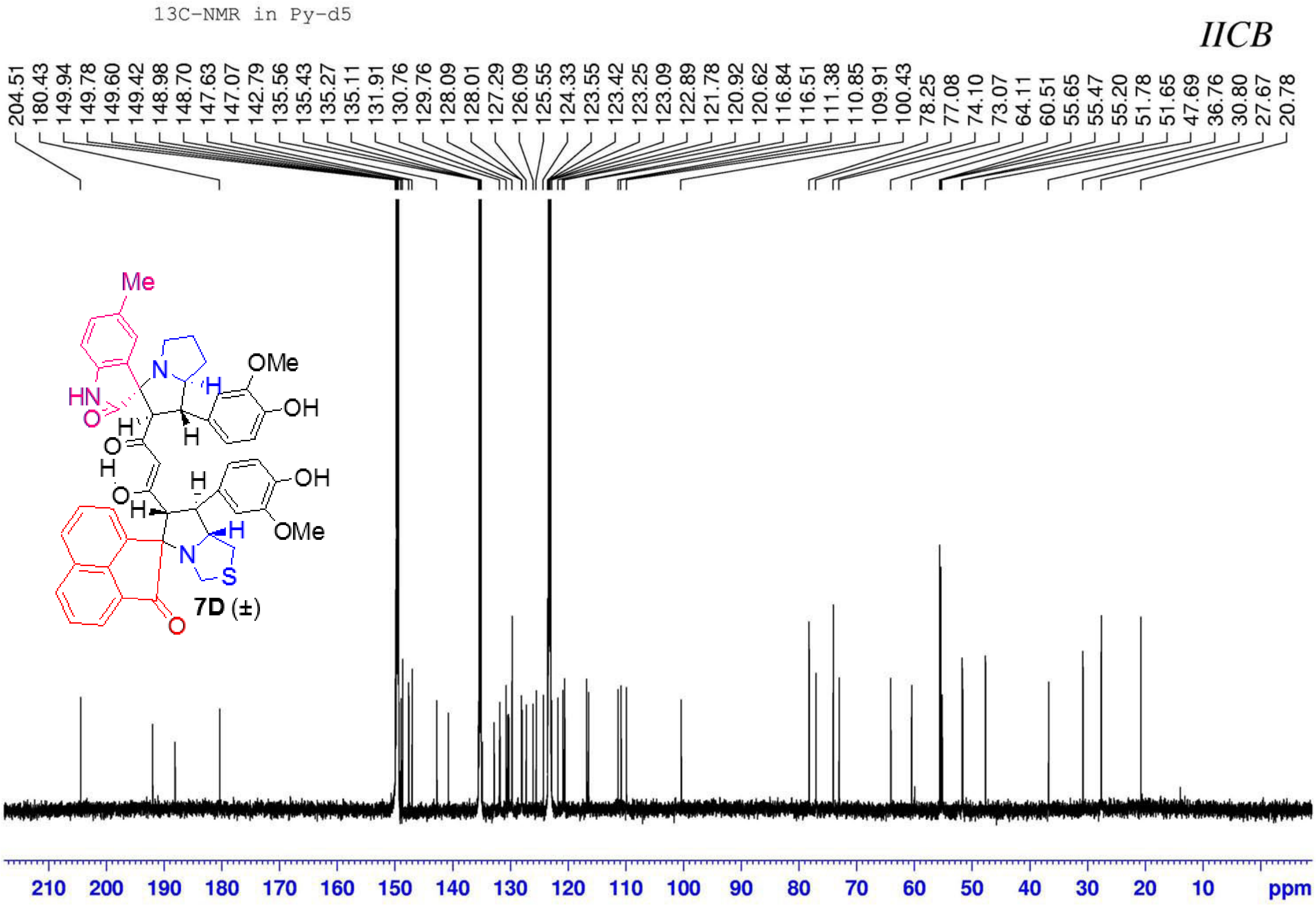
13C-NMR in Py-d5
IICB
204.51
180.43
149.94
149.78
149.60
149.42
148.98
148.70
147.63
147.07
142.79
135.56
135.43
135.27
135.11
131.91
130.76
129.76
128.09
128.01
127.29
126.09
125.55
124.33
123.55
123.42
123.25
123.09
122.89
121.78
120.92
120.62
116.84
116.51
111.38
110.85
109.91
100.43
78.25
77.08
74.10
73.07
64.11
60.51
55.65
55.47
55.20
51.78
51.65
47.69
36.76
30.80
27.67
20.78
Me
HN
O
N
H
OMe
OH
H
O
H
O
H
OH
OMe
N
S
O
7D (±)
210
200
190
180
170
160
150
140
130
120
110
100
90
80
70
60
50
40
30
20
10
ppm

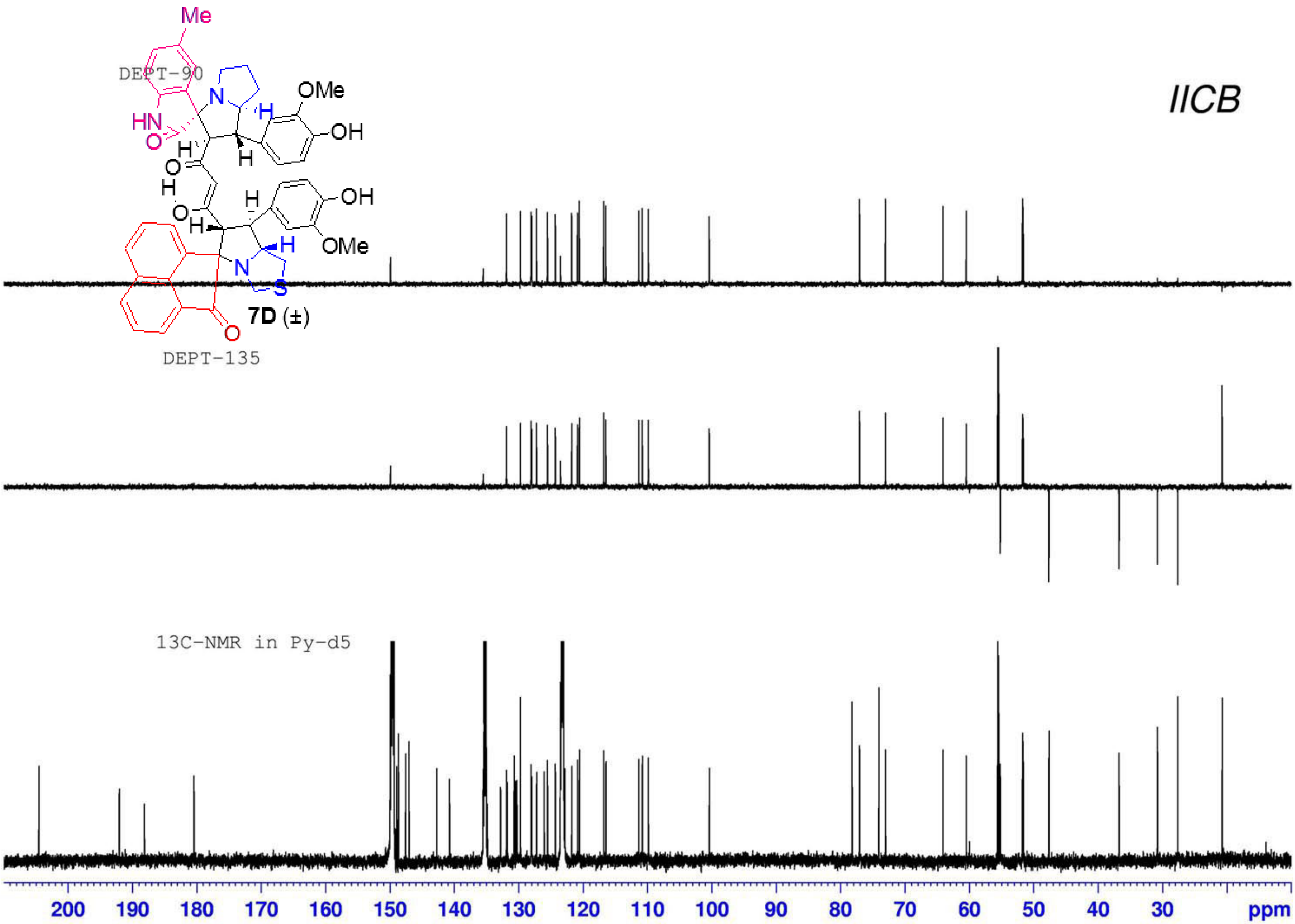

IICB
DEPT-90
DEPT-135
13C-NMR in Py-d5
7D (±)
Me
HN
O
N
H
OMe
OH
O
H
O
H
H
OH
OMe
N
S
O
200
190
180
170
160
150
140
130
120
110
100
90
80
70
60
50
40
30
ppm

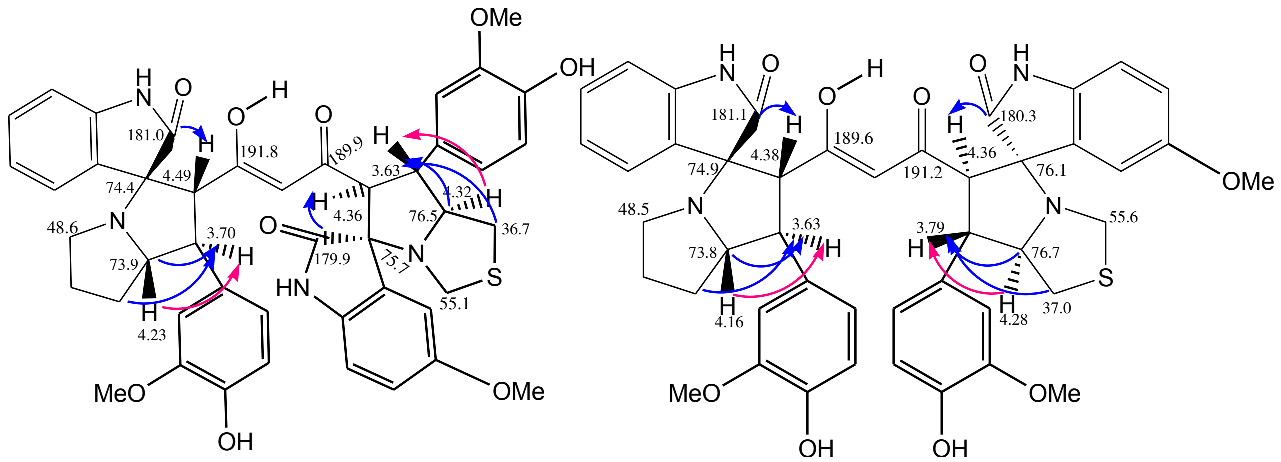

Figure 2. Important correlations of **6Aa** and **6Ab** [HMBC ( ), COSY ( )]

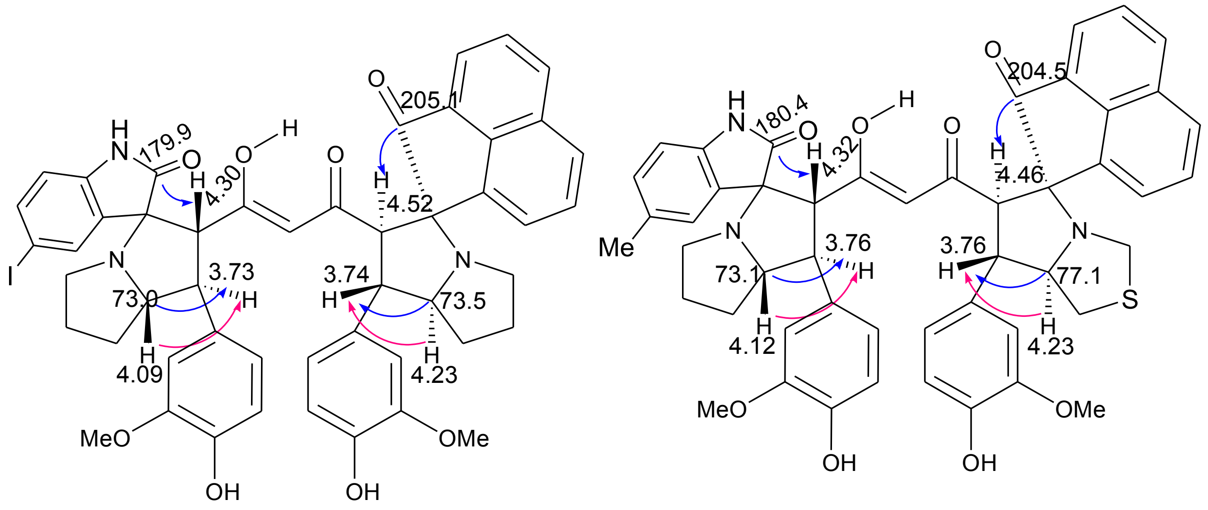

Figure 3. Important correlations of **7B** and **7D** [HMBC ( ), COSY ( )]

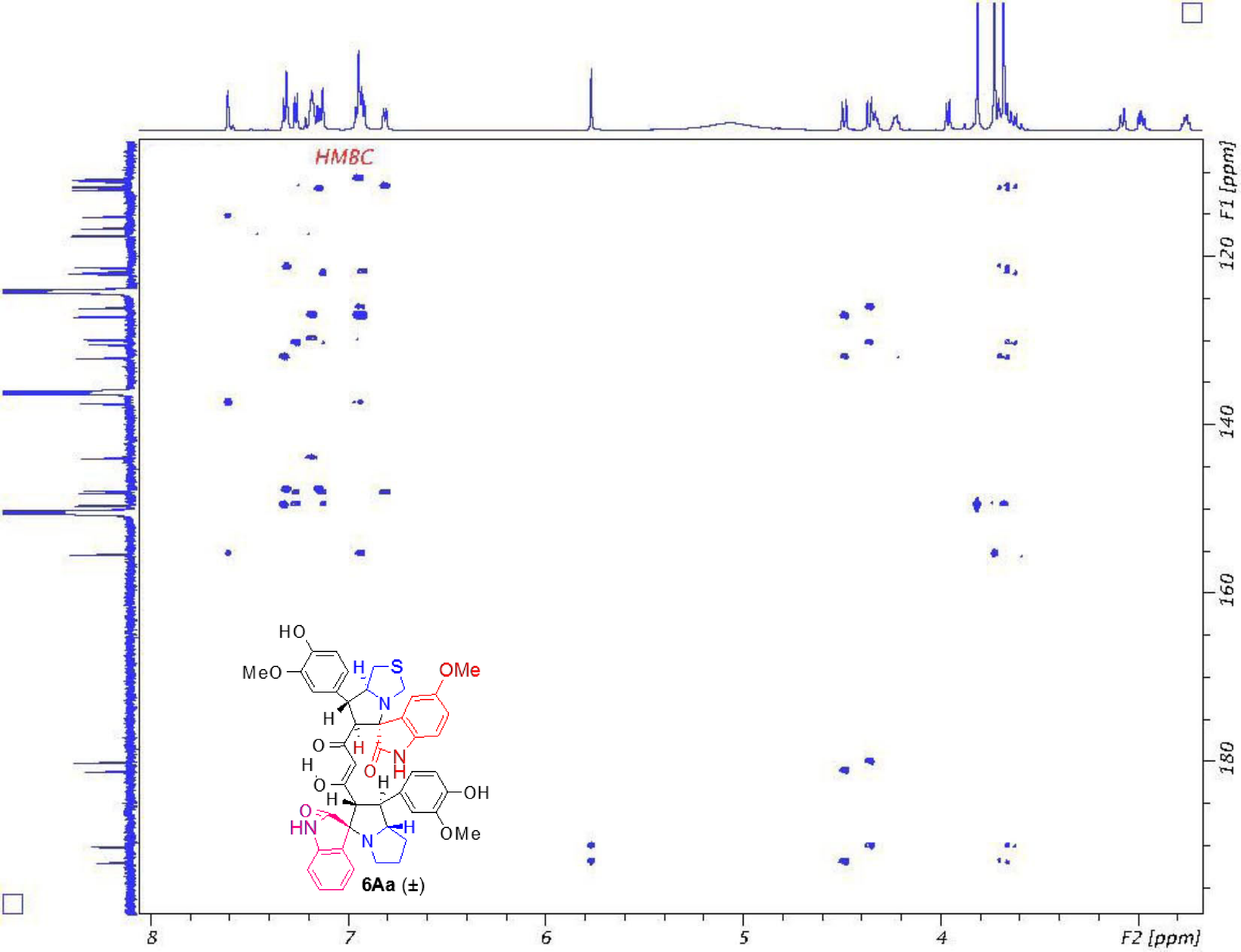

HMBC
F1 [ppm]
120
140
160
180
8
7
6
5
4
F2 [ppm]
HO
MeO
H
S
N
OMe
H
O
H
NH
O
H
O
H
H
OH
OMe
O
HN
N
H
6Aa (±)

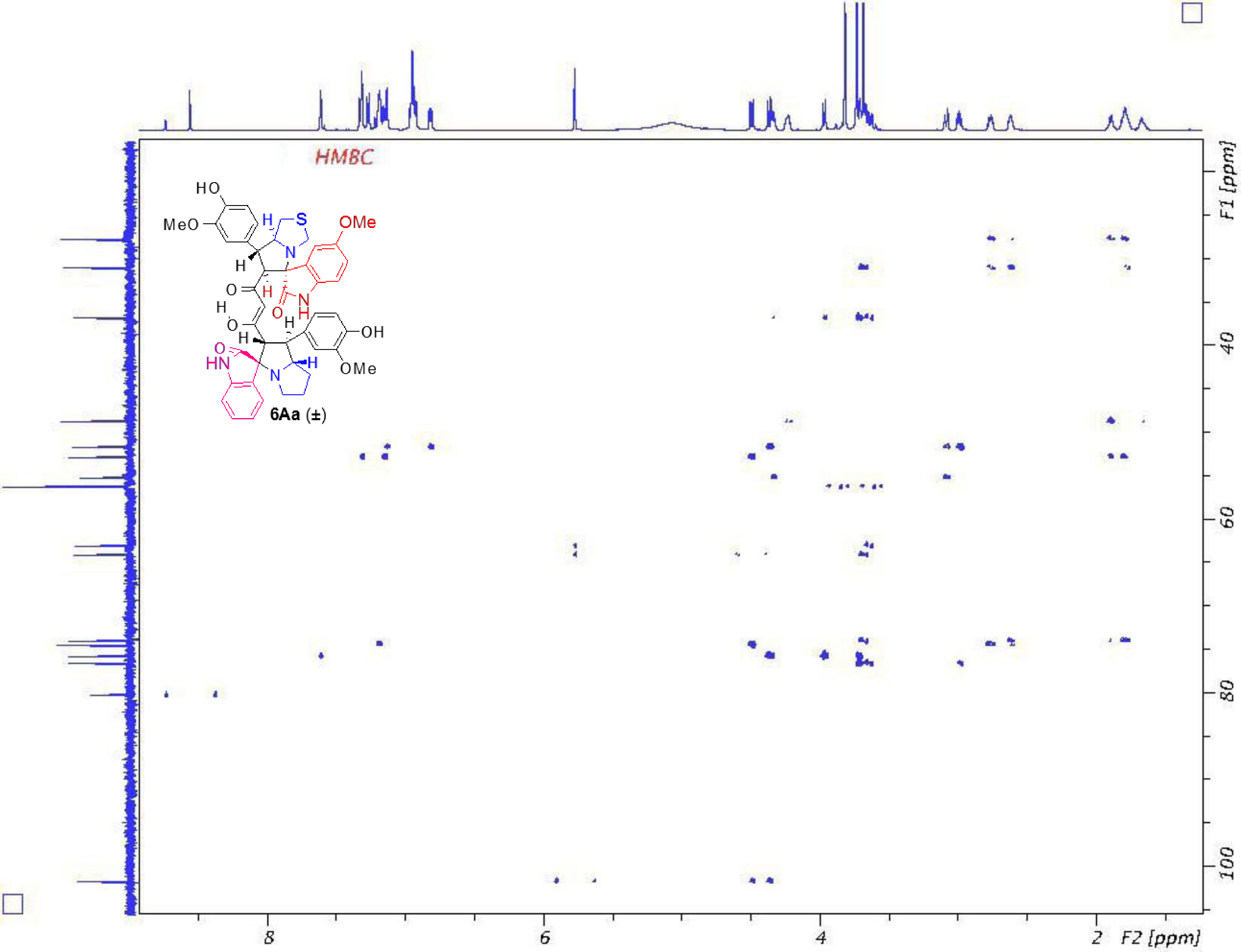

HMBC
HO
MeO
H
S
N
OMe
H
O
H
N
H
H
O
O
H
H
OH
O
HN
N
H
OMe
6Aa (±)
F1 [ppm]
20
40
60
80
100
8
6
4
2
F2 [ppm]

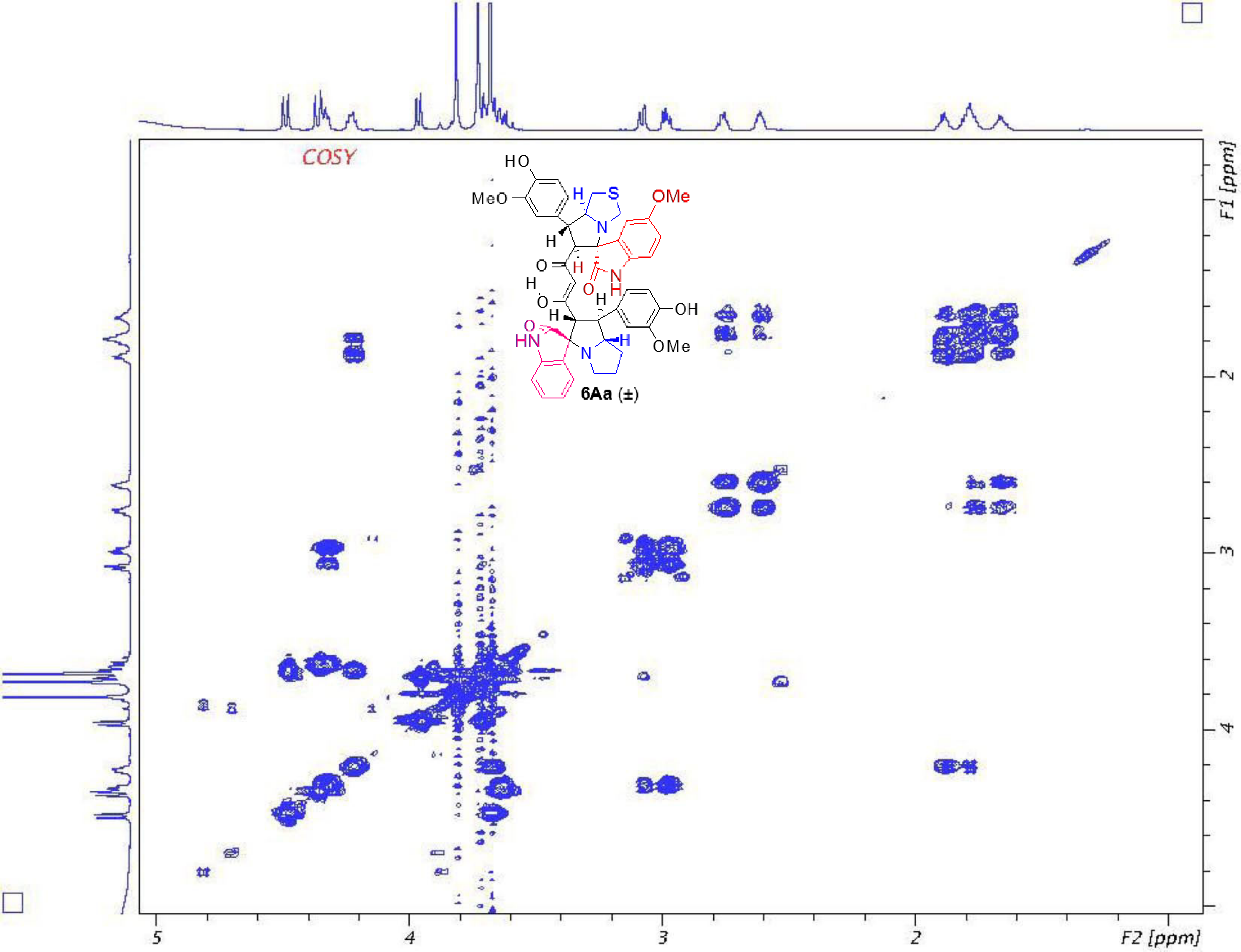
COSY
HO
MeO
H
S
OMe
N
H
O
H
N
H
H
O
H
O
H
OH
H
O
HN
N
H
OMe
6Aa (±)
F1 [ppm]
F2 [ppm]
5
4
3
2

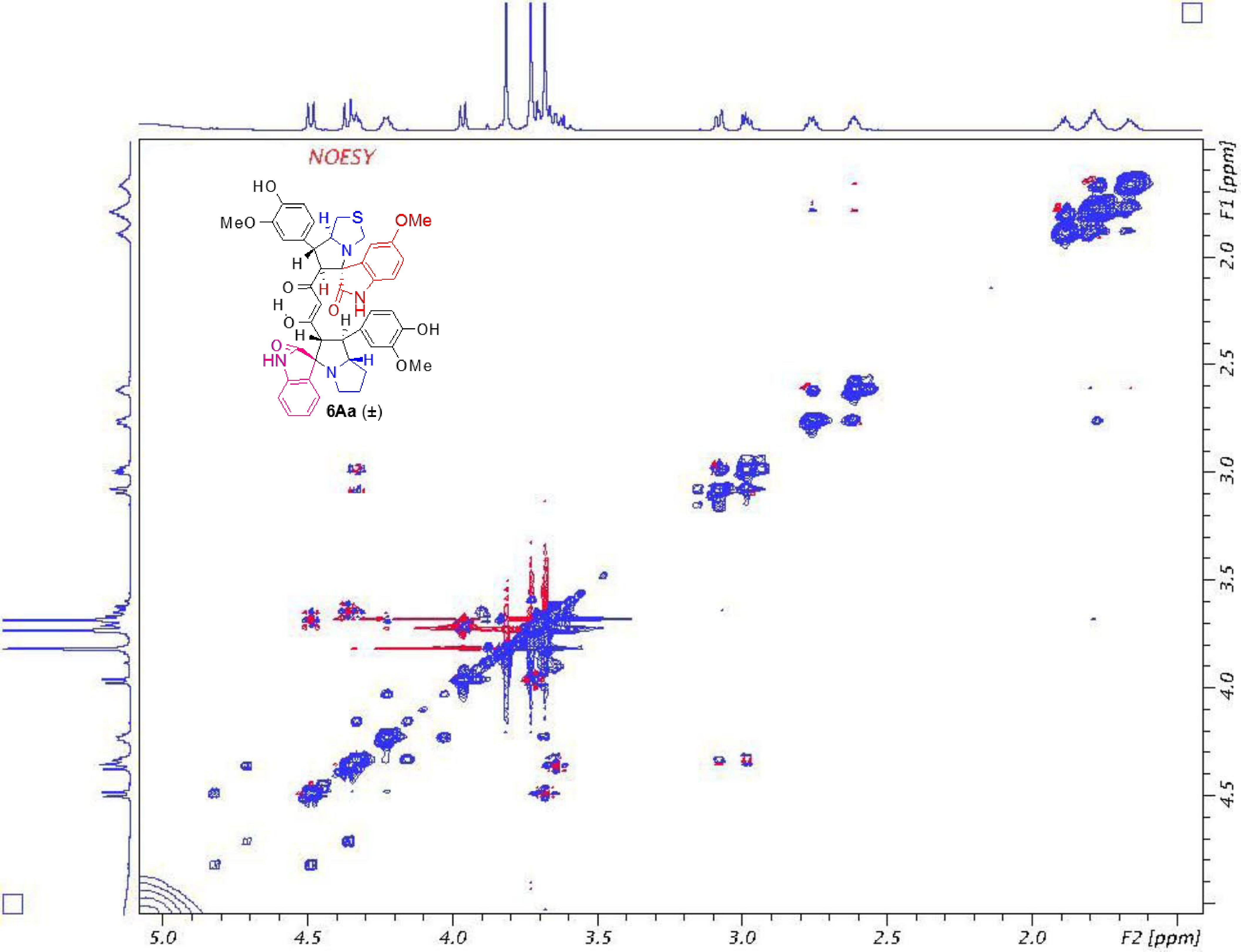
NOESY
HO
MeO
H
S
N
OMe
H
O
H
O
N
H
H
O
H
H
OH
O
HN
N
H
OMe
6Aa (±)
F1 [ppm]
F2 [ppm]
5.0
4.5
4.0
3.5
3.0
2.5
2.0

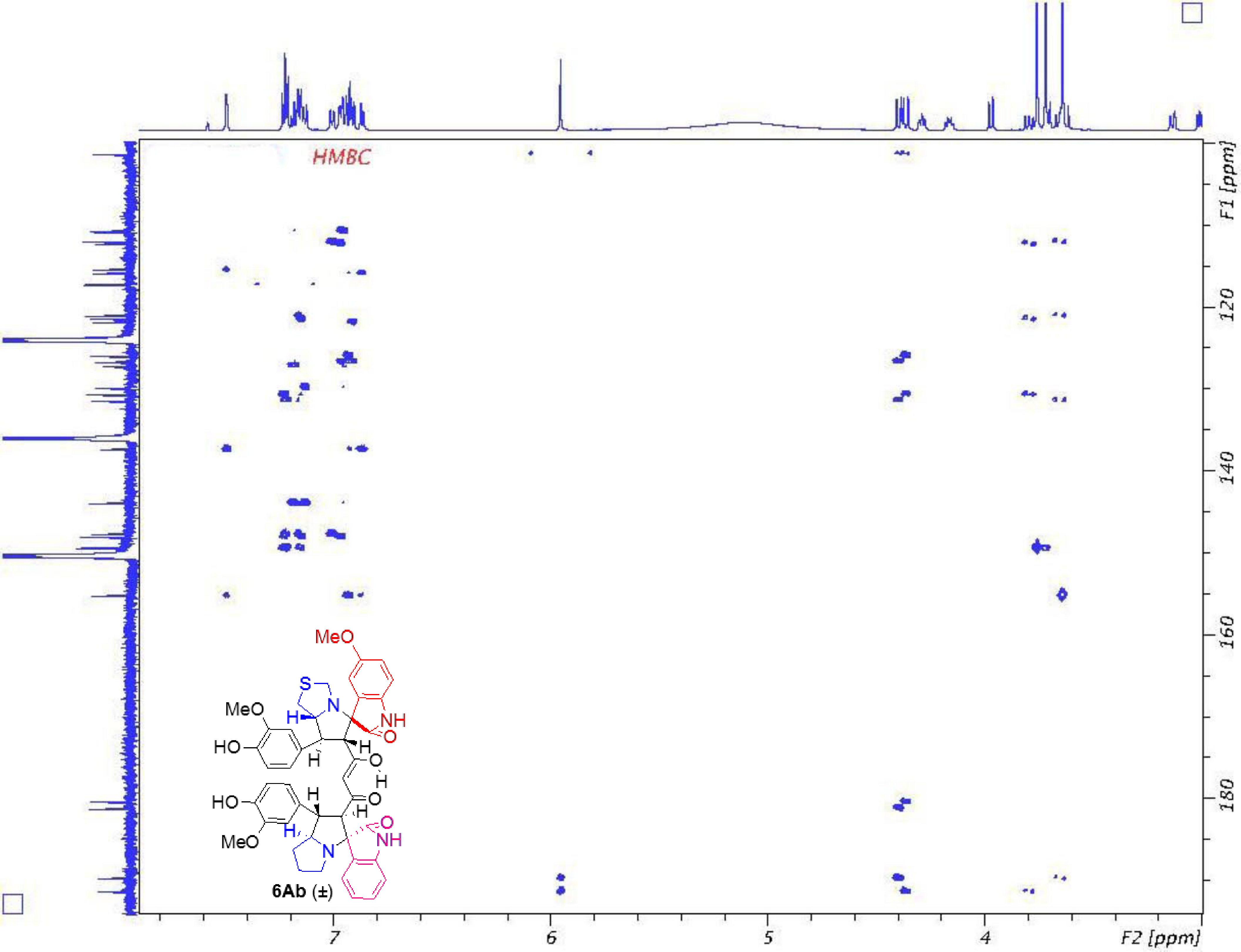

HMBC
F1 [ppm]
120
140
160
180
F2 [ppm]
7
6
5
4
MeO
S
N
H
NH
O
MeO
HO
H
H
O
H
HO
H
O
H
O
MeO
H
N
NH
6Ab (±)

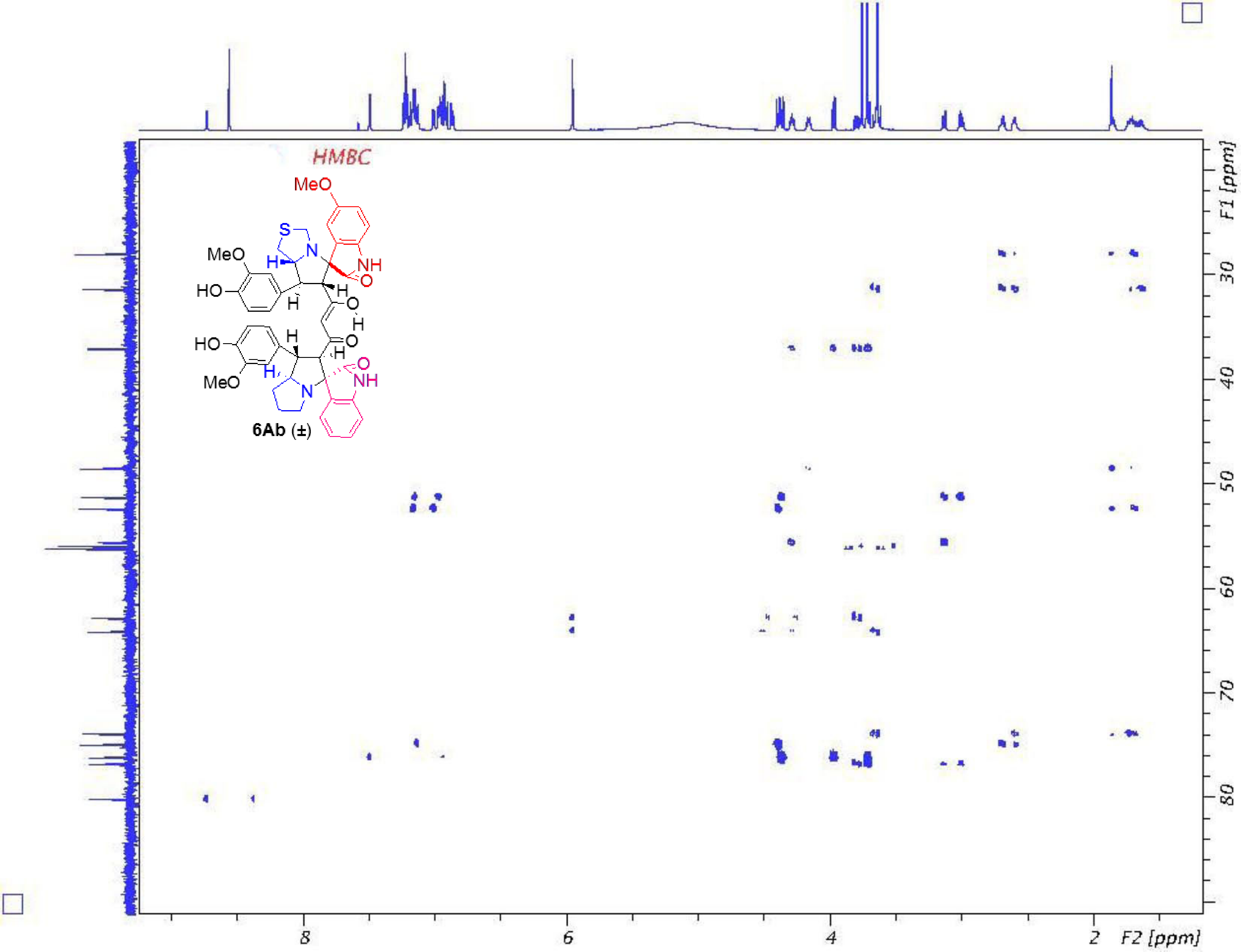
HMBC
MeO
S
N
H
NH
O
MeO
HO
H
H
O
H
HO
H
O
H
MeO
H
O
N
NH
6Ab (±)
F1 [ppm]
30
40
50
60
70
80
8
6
4
2
F2 [ppm]

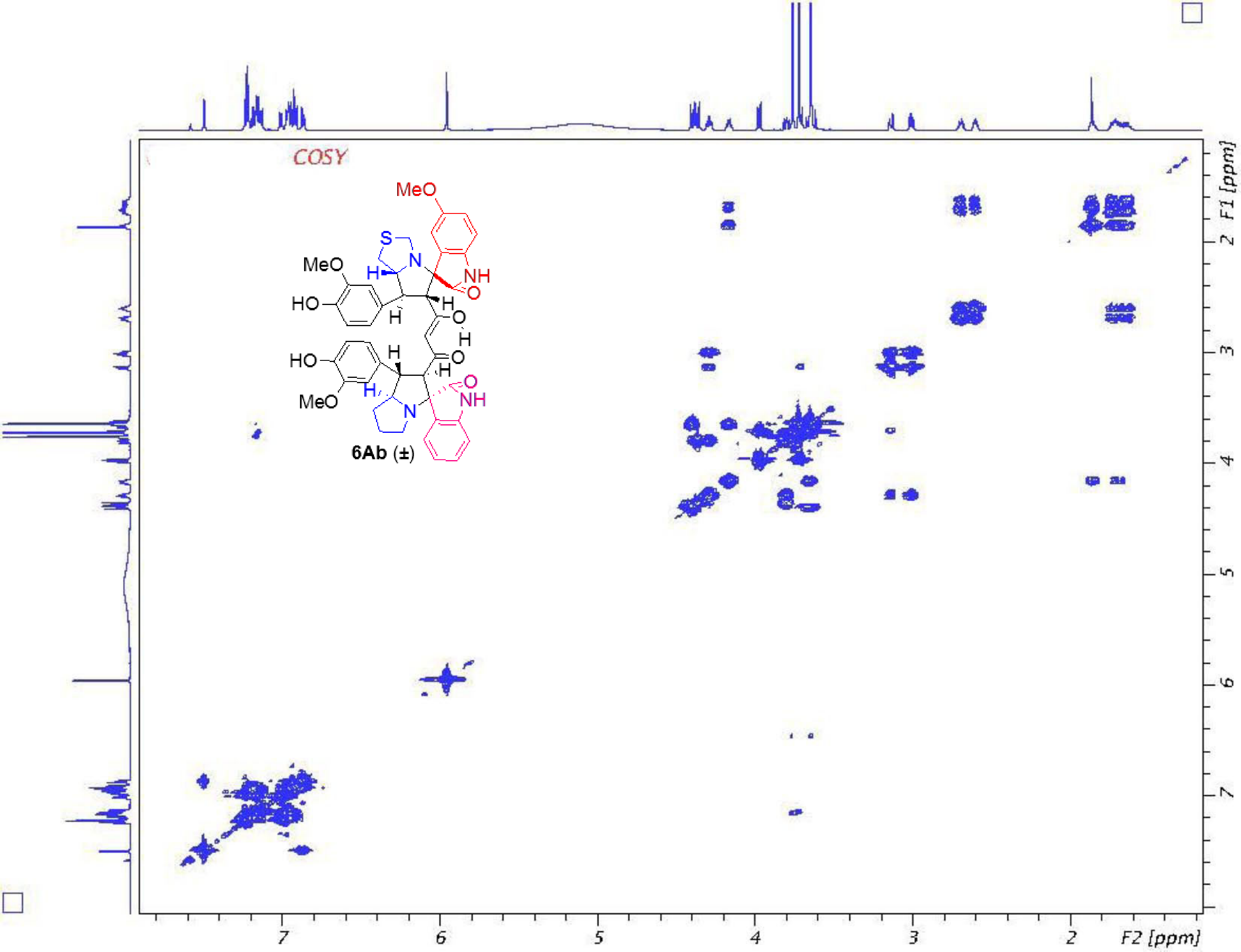
COSY
MeO
S
N
MeO
H
HO
NH
O
H
H
O
H
HO
H
O
H
O
MeO
H
NH
N
6Ab (±)
F1 [ppm]
F2 [ppm]
7
6
5
4
3
2

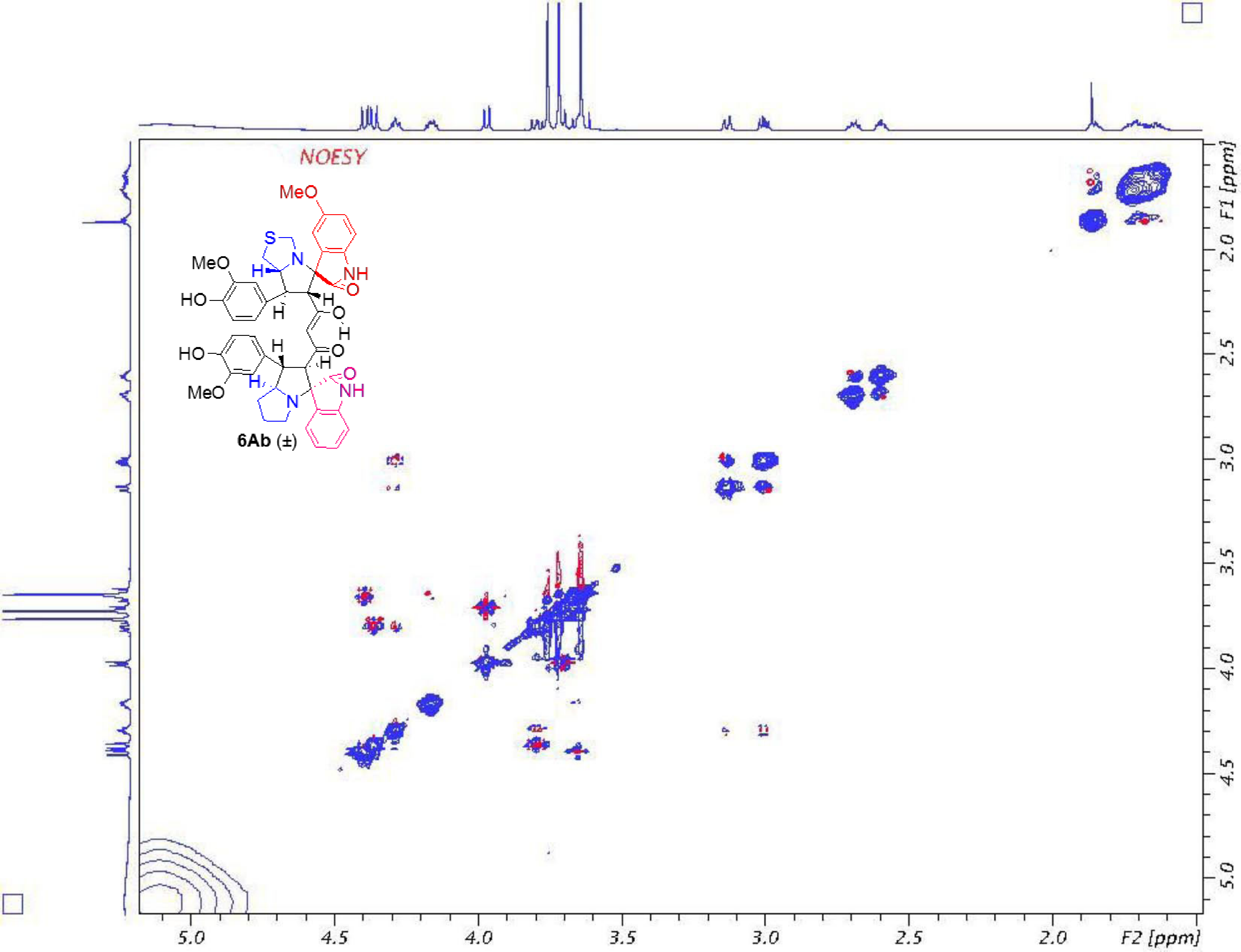
NOESY
MeO
S
N
H
NH
O
MeO
HO
H
H
O
H
H
O
HO
MeO
H
H
O
NH
N
6Ab (±)
F1 [ppm]
F2 [ppm]
5.0
4.5
4.0
3.5
3.0
2.5
2.0

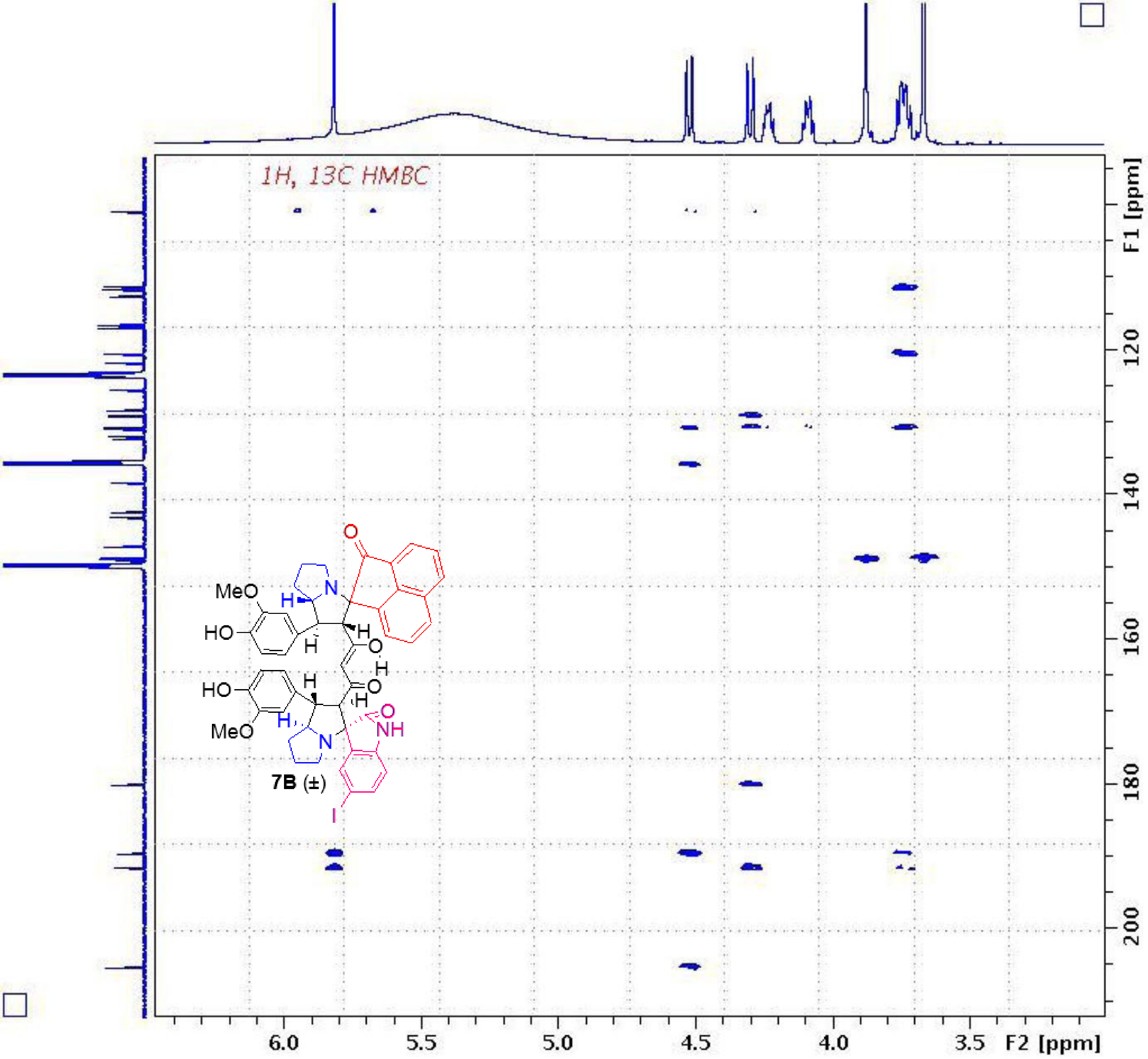
1H, 13C HMBC
F1 [ppm]
120
140
160
180
200
6.0
5.5
5.0
4.5
4.0
3.5
F2 [ppm]
MeO
HO
HO
MeO
NH
I
7B (±)

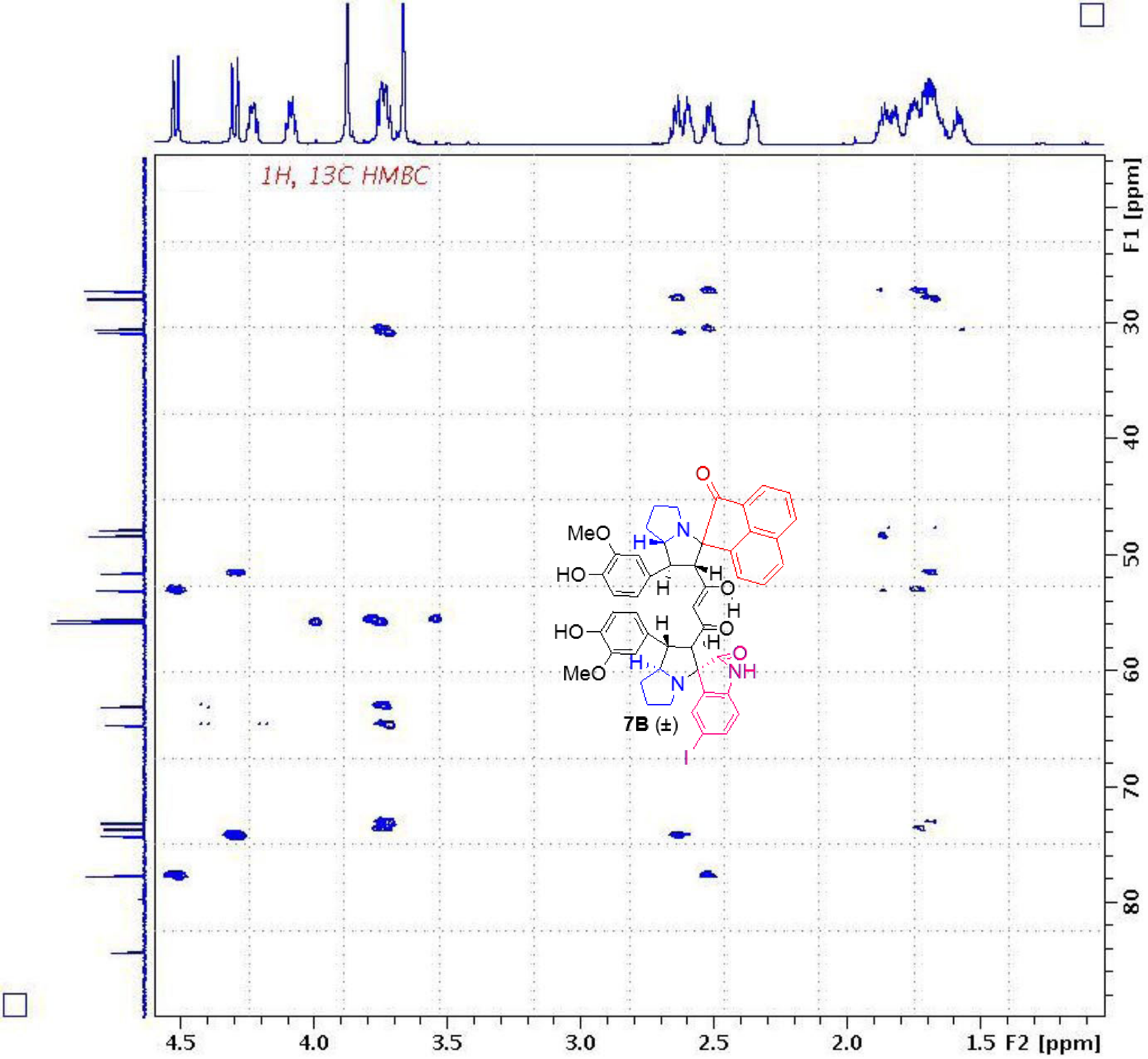
1H, 13C HMBC
F1 [ppm]
F2 [ppm]
4.5
4.0
3.5
3.0
2.5
2.0
1.5
30
40
50
60
70
80
MeO
HO
HO
MeO
N
N
H
H
H
H
H
H
O
O
O
O
NH
I
7B (±)

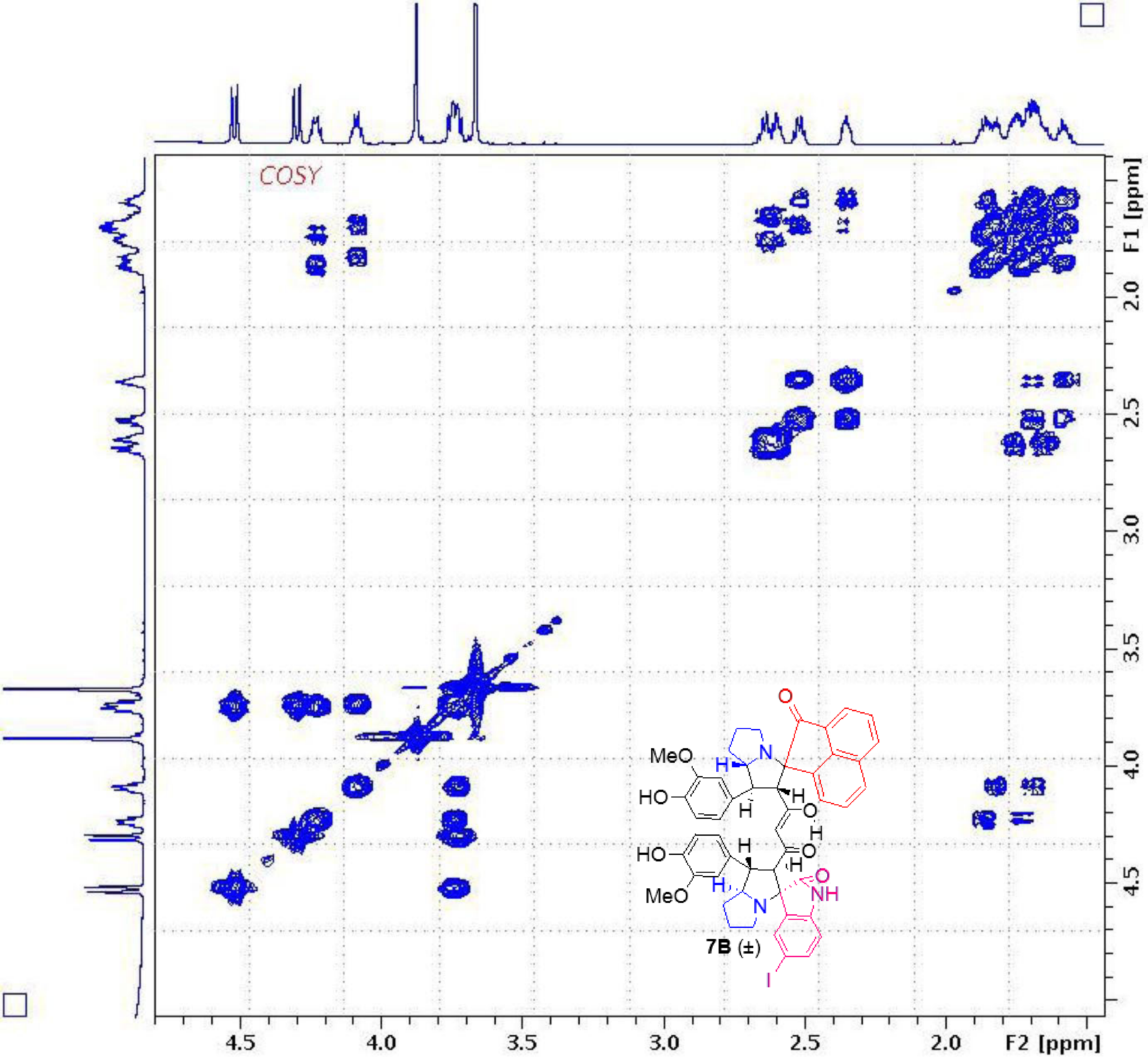
COSY
F1 [ppm]
F2 [ppm]
2.0
2.5
3.0
3.5
4.0
4.5
MeO
HO
HO
MeO
NH
I
7B (±)

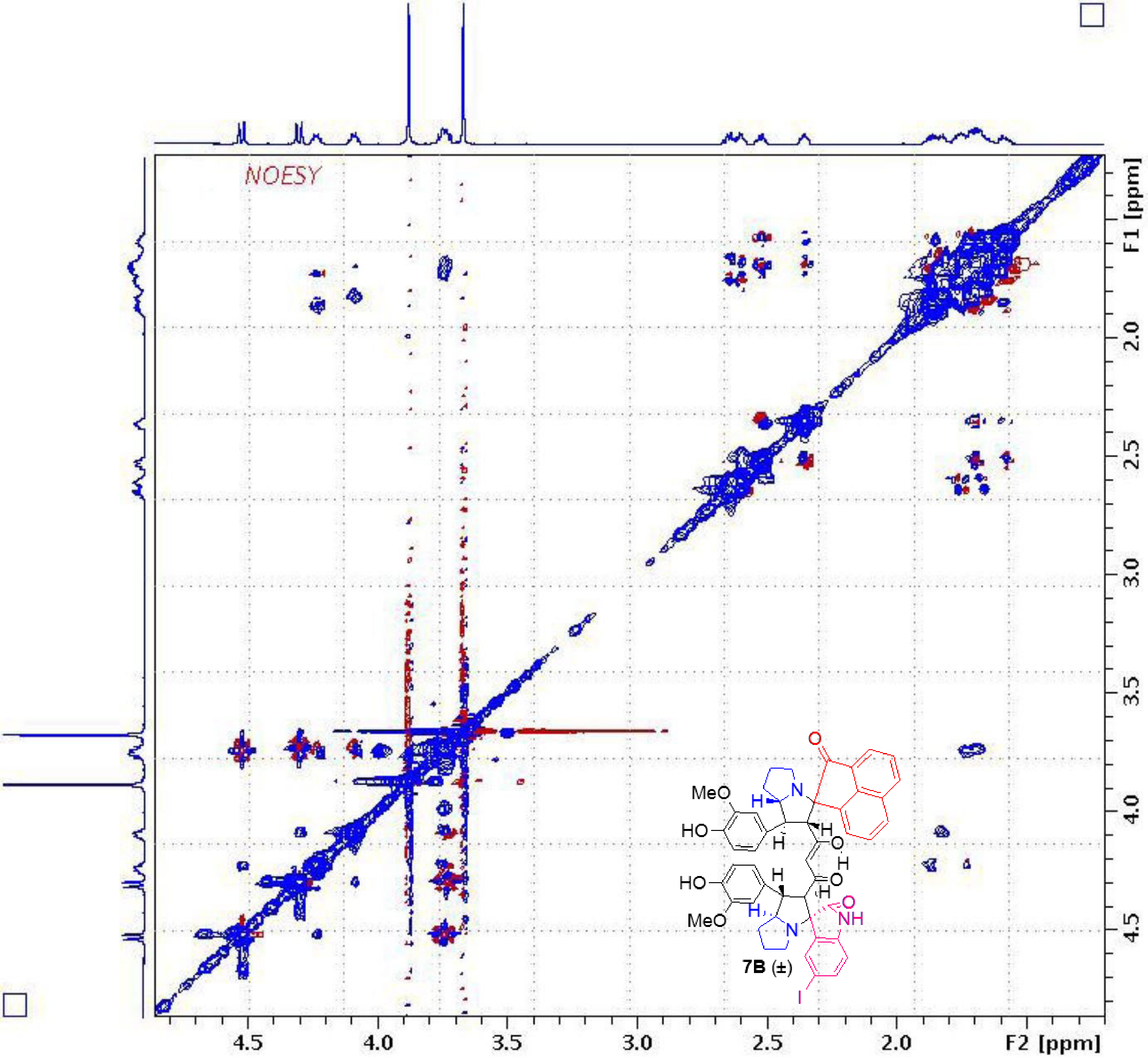
NOESY
F1 [ppm]
F2 [ppm]
2.0
2.5
3.0
3.5
4.0
4.5
MeO
HO
H
N
O
H
H
O
H
HO
H
O
H
H
MeO
N
O
NH
I
7B (±)

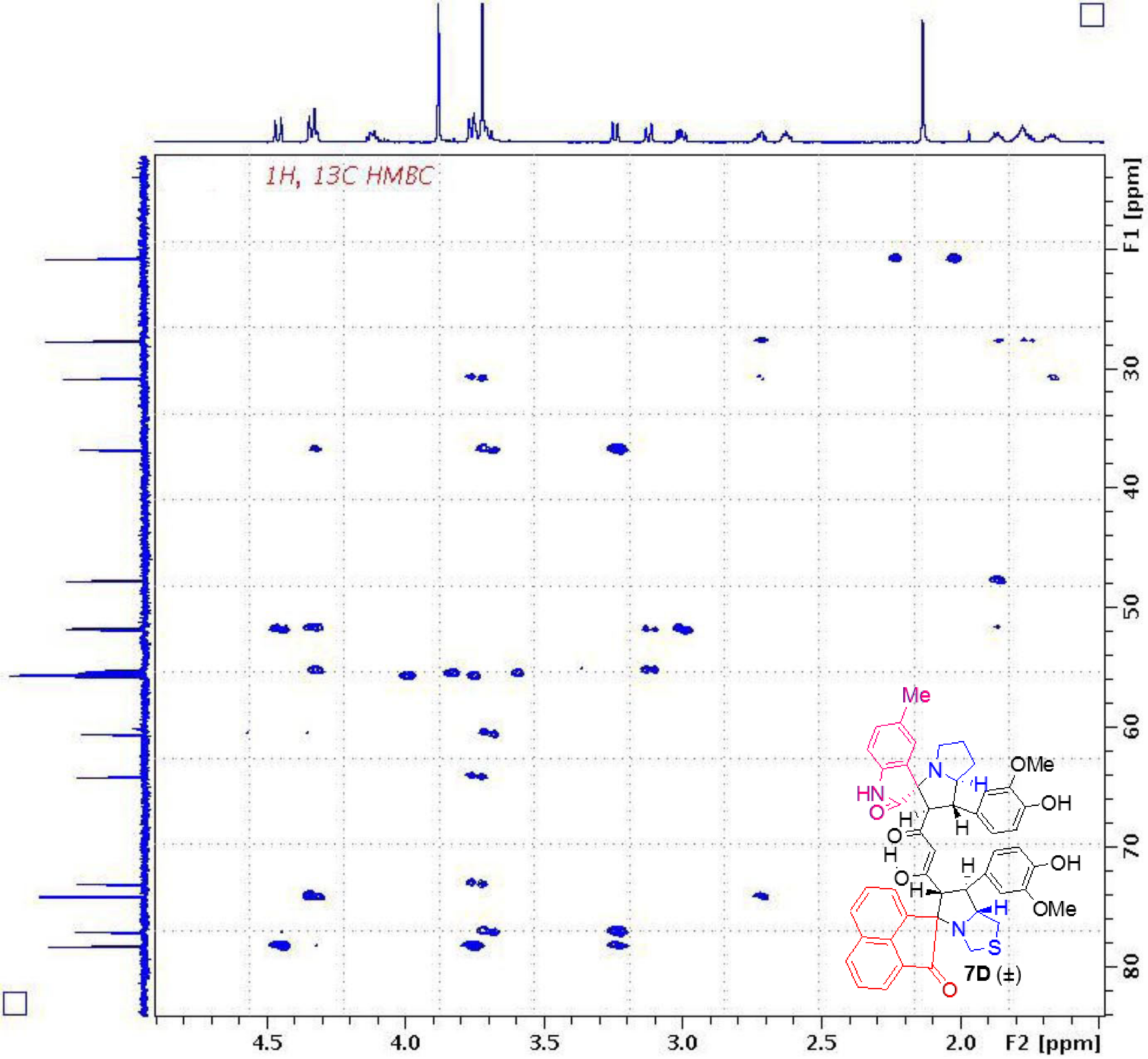

1H, 13C HMBC
F1 [ppm]
F2 [ppm]
4.5
4.0
3.5
3.0
2.5
2.0
30
40
50
60
70
80
Me
HN
O
N
H
OMe
OH
H
H
O
H
O
H
H
OH
H
OMe
N
S
O
7D (±)

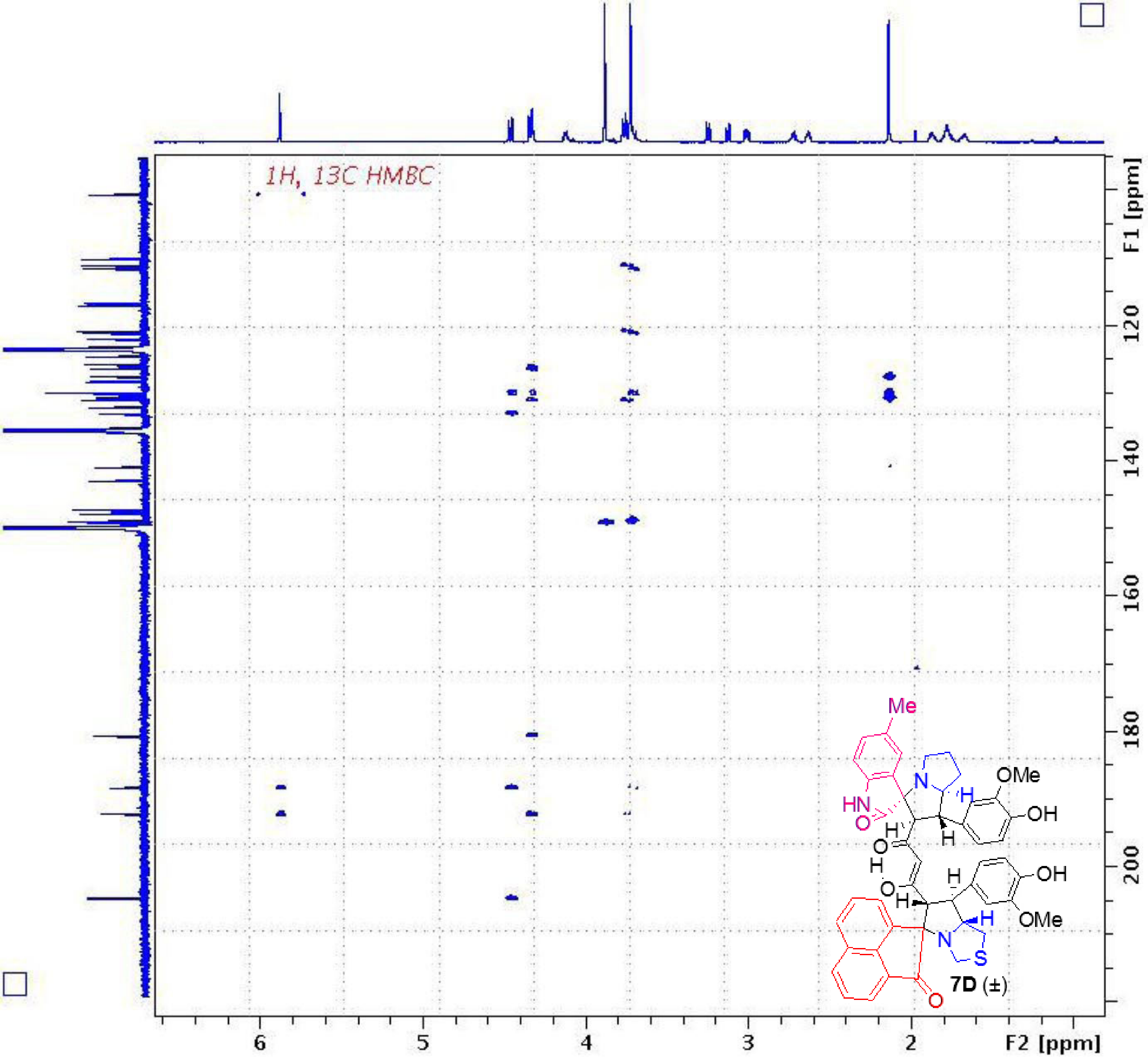
1H, 13C HMBC
F1 [ppm]
120
140
160
180
200
6
5
4
3
2
F2 [ppm]
Me
HN
O
N
H
OMe
OH
H
H
O
H
O
H
OH
H
H
OMe
N
S
O
7D (±)

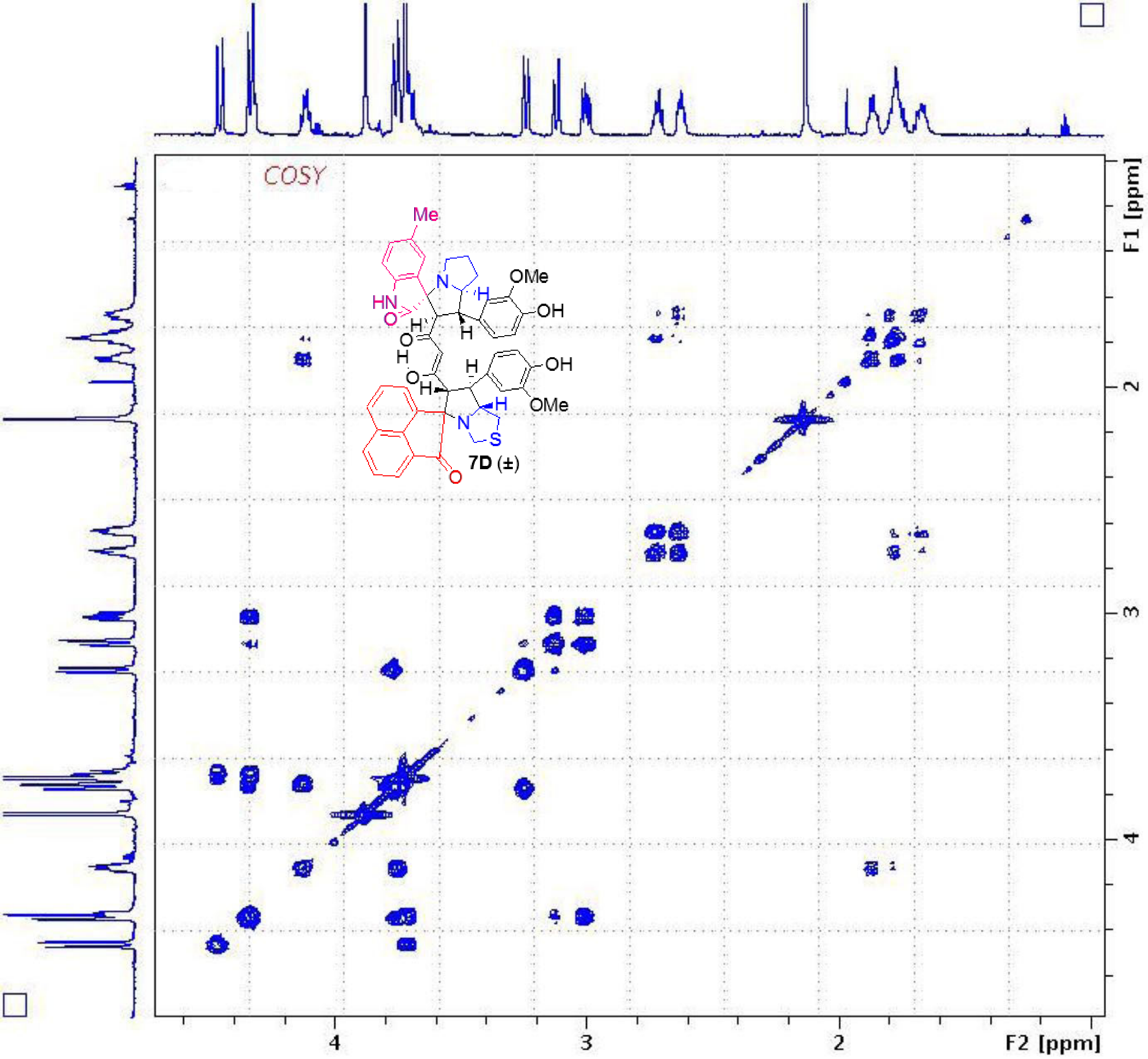
COSY
Me
N
H
OMe
OH
HN
O
H
H
O
H
O
H
OH
H
OMe
H
N
S
O
7D (±)
F1 [ppm]
2
3
4
F2 [ppm]
4
3
2

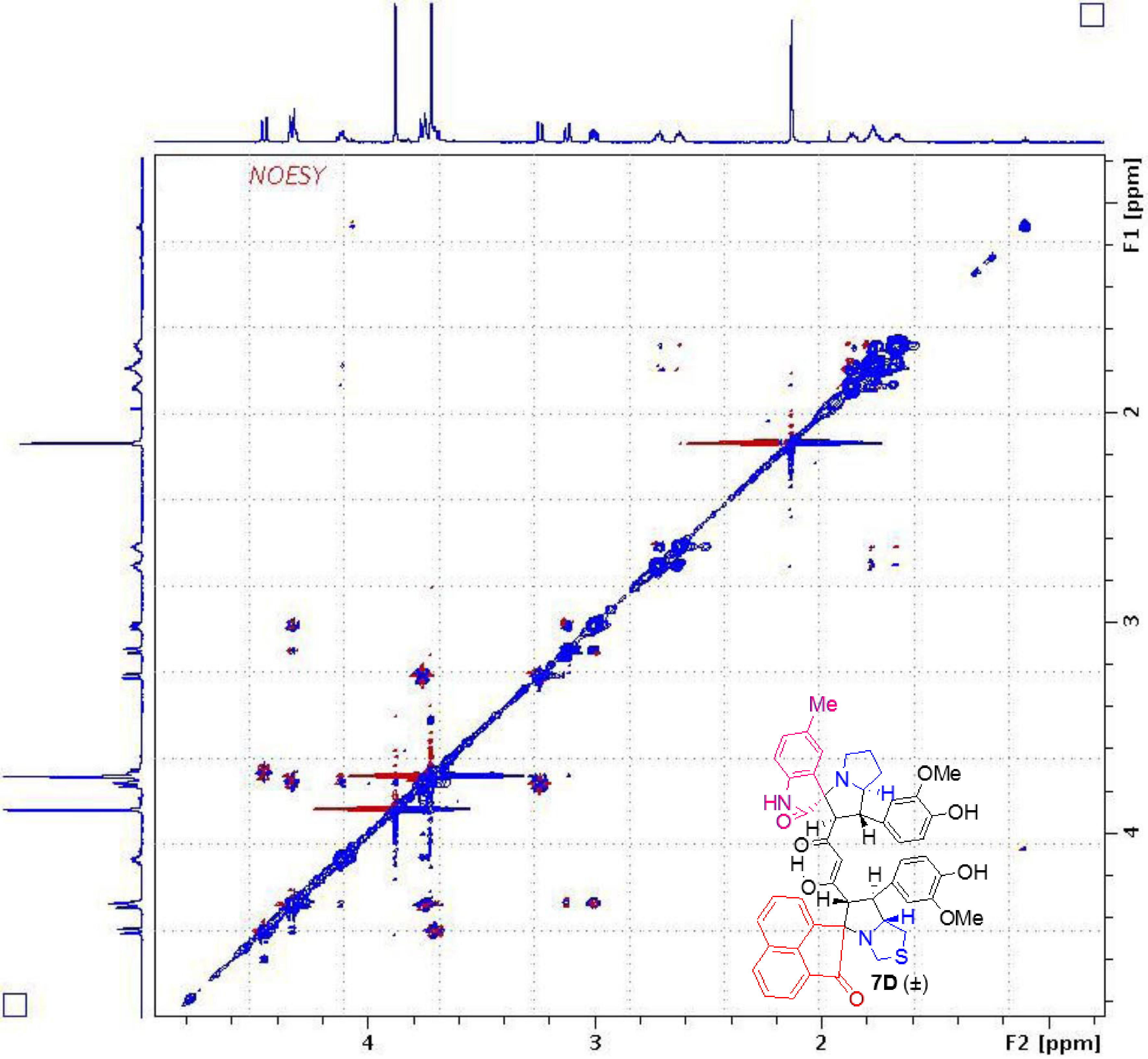
NOESY
F1 [ppm]
2
3
4
4
3
2
F2 [ppm]
Me
HN
O
N
H
OMe
OH
H
H
O
H
O
H
OH
H
H
OMe
N
S
O
7D (±)
